# Hydrogenation of Alkynes and Olefins Catalyzed by Quaternary Ammonium Salts

**DOI:** 10.1002/advs.202305271

**Published:** 2023-12-10

**Authors:** Qi Guo, Guoli Shen, Guangfu Lu, Jinyi Qian, Qitao Que, Jiuling Li, Yafei Guo, Baomin Fan

**Affiliations:** ^1^ Yunnan Key Laboratory of Chiral Functional Substance Research and Application School of Chemistry & Environment Yunnan Minzu University 2929 Yuehua road Kunming 650500 China

**Keywords:** alkynes, hydrogenation, olefins, quaternary ammonium salt

## Abstract

Catalytic hydrogenation of unsaturated hydrocarbons to alkenes and alkanes using molecular hydrogen is one of the most fundamental transformations in organic synthesis. While methodologies involving transition metals as catalysts in homogeneous and heterogeneous processes have been well developed, metal‐free catalytic hydrogenation offers an ideal approach for future chemistry. Herein, the common and inexpensive quaternary ammonium salts are first introduced as catalysts in the catalytic hydrogenation system for the transformations from alkynes or olefins into the corresponding olefins or alkanes. Interestingly, the hydrogenation process of alkynes can be controlled to selectively produce alkenes or alkanes under different conditions. Moreover, the possible mechanism is discussed in new insights into the catalytic behavior of quaternary ammonium salts.

## Graphical abstract

A new methodology on catalytic hydrogenation of alkynes and olefins is disclosed with good to moderate yields. Interestingly, the most common and commercial quaternary ammonium salts are first discovered to have the catalytic ability for the cleavage and addition of molecule hydrogen to unsaturated hydrocarbons.

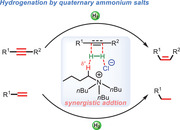

## 1 Introduction

**Scheme 1:**
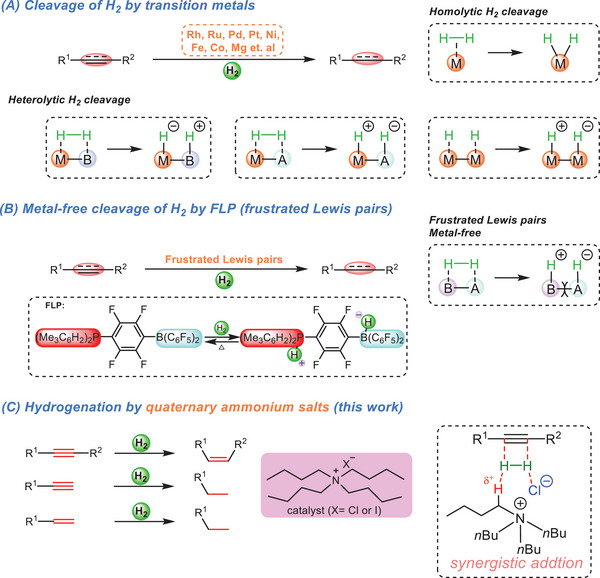
Hydrogenation of multiple C—C bonds with different methods. A) Transition metals catalyzed hydrogenation of alkynes and olefins. B) Frustrated Lewis pairs catalyzed hydrogenation of alkynes and olefins. (C) This work.

The hydrogenation of multiple C—C bonds through the cleaving and addition of H_2_ has been one of the most valuable and key technologies in the past 100 years, and finds a wide range of applications in petrochemical, food, agricultural and pharmaceutical industries.^[^ ^1^ ^]^ The precious metals, such as Ru, Rh, Pd, and Ir, played an irreplaceable role in the industrial hydrogenation of unsaturated hydrocarbons owing to their high reactivity in cleaving H_2_ molecule and delivering hydrogen to substrates, for example the famous Wilkinson, Schrock‐Osborn, Lindlar and Crabtree catalysts.^[^ ^2^ ^]^ Recently, the earth‐abundant and environmentally friendly transition metal catalysts, such as Fe, Mn, Co, Cu, Ni and Mg have successfully received much increasing interest.^[^ ^3^, ^4^ ^]^ In general, there are two strategies to cleavage H_2_ molecule using transition metals, namely single‐site and cooperative strategies (**Scheme** **1**A). Most of precious metals are employed as single‐site catalysts to cleavage H_2_ through homolytic oxidative addition (Scheme 1A). Furthermore, multiple cooperative catalysts, including metal‐base, metal‐acid and metal‐metal, have been well developed to cleavage H_2_ through heterolytic way (Scheme 1A). Despite the transition metal catalysts being irreplaceable in the hydrogenation transformations so far, the pursue of greener and low‐cost metal‐free catalysts is a growing fascination for scientists.

In 2006, a new concept known as “frustrated Lewis pairs (FLPs)” was introduced by Stephan to activate H_2_ without metal catalyst under mild conditions which was described as the most efficient metal‐free catalytic system for the hydrogenation of unsaturated bonds.^[^ ^5^ ^]^ Interestingly, it was first discovered that the H—H bond can be activated and split to generate stable hydrogen ion and cation by using a phosphine‐borane substrate and the H_2_ can be recovered above 100 °C (Scheme 1B).^[^ ^6^ ^]^ Based on this remarkable discovery, a wide range of unsaturated substrates, such as imines, enamines, olefins, alkynes and anilines, were successfully hydrogenated by H_2_ under metal‐free conditions.^[^ ^7^ ^]^ For example, in 2012, Grimme and Paradies disclosed a FLPs [(C_6_F_5_)_3_B‐Lewis base] catalytic olefin hydrogenation methodology under mild conditions.^[^ ^8^ ^]^ After that, Repo and Pápai discovered an interesting FLPs catalyst bearing Lewis base and acid which could be employed to produce a wide variety of internal alkynes into *cis*‐olefins.^[^ ^9^ ^]^ It was worth mentioning that only transition metal catalysts could lead to this transformations before this excellent report. However, the terminal alkynes were not tolerated with this method. Furthermore, Du developed an interesting strategy by using alkenes as promoters and HB(C_6_F_5_)_2_ as catalyst to selectively hydrogenation of alkynes to observe *Z*‐ and *E*‐olefins.^[^ ^10^ ^]^

Although the metal‐free FLPs chemistry has greatly advanced the hydrogenation of unsaturated hydrocarbons, there are still some challenges that need to addressed. Here, we disclose a new catalytic pathway by using the most common quaternary ammonium salt (R_4_NX) to activate H_2_. Remarkably, this new system showed a promising catalytic efficiency on the hydrogenation of H_2_ to unactivated alkynes and olefins (Scheme 1C).

## 2 Results and Discussion

We started off our investigations using the terminal alkyne (1a) as the starting substrate to explore the optimal reaction conditions. The preliminary experiment was carried out in toluene under the pressure of H_2_ (0.5 MPa) at 80 °C in the absence of quaternary ammonium salts (entry 1). As anticipated, no hydrogenation product was obtained. However, to our delight, when the most common and commercial Bu_4_NCl (tetrabutylammonium chloride) was introduced in this reaction as catalyst, the terminal olefin 2a was observed with 93% yield (entry 2). It should be noted that the corresponding hydrogenated alkane was not found in the products and this hydrogenation reaction was selective towards the formation of the alkene. Furthermore, when the hydrogen pressure was reduced to 0.5 MPa, it still could provide 2a up to 91% yield (entry 3). Nonetheless, this hydrogenation process could not completely be finished under the condition of hydrogen balloon and only generated 2a with 48% yield (entry 4). In addition, 39% yield was received by shortening reaction time from 72 to 48 h even under the pressure of 5.0 MPa (entry 5). Whereafter, several solvents were examined to compare with toluene, such as THF (tetrahydrofuran), DCE (dichloroethane), 1,4‐Dioxane and DMF (dimethyl formamide) (entries 6–9). The results suggested that THF and Toluene gave higher yields than other solvents, 92% and 91% respectively. Moreover, the lower temperature of 60 °C led to a slight decrease of yield (entry 10). In order to further explore the influence of catalysts, various quaternary ammonium salts were evaluated in the solvent of THF (entries 11–16). Interestingly, Bu_4_NBr (tetrabutylammonium bromide) and Bu_4_NCl could produce 2a with more than 90% yields and Bu_4_NI (tetrabutylammonium iodide) also gave 68% yield. However, only trace products were obtained for Me_4_NCl (tetramethylammonium chloride)and Me_4_NI (tetramethylammonium iodide). In addition, compared with Bu_4_NBF_4_ (tetrabutylammonium tetrafluoroborate) and Bu_4_NOTf (tetrabutylammonium trifluoromethanesulfonate), the results were quite different with 61% and trace yields respectively. Consequently, both anions and cations of catalysts are important for the yields. As a result, the optimization of hydrogenation conditions from alkynes to olefins were chosen as following: 1 (0.2 mmol) in 2 mL THF at 80 °C for 72 h under the pressure of 0.5 MPa.

**Scheme 2:**
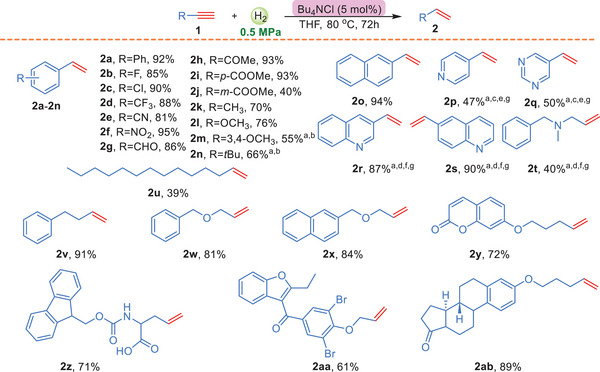
Hydrogenation of terminal alkynes to olefins. All reactions were performed with 1 (0.2 mmol), pressure of H_2_ (0.5 MPa), in 2 mL THF at 80 °C for 72 h. Yields are those for the isolated products. ^a^50 mol% *n*Bu_4_NCl was used. ^b^The reaction temperature was100 °C. ^c^the reaction temperature was 120 °C. ^d^the reaction temperature was150 °C. ^e^Pressure of H_2_ (5.0 MPa) ^f^Pressure of H_2_ (7.0 MPa). ^g^50 mol% *n*Bu_4_NI was used.

With the optimized conditions in hand, we proceeded to examine the scope of various terminal alkynes and the results were shown in **Scheme** **2**. Initially, the model reaction had been well explored in **Table** **1** and furnished product 2a in 92% yield. Afterward, various aromatic terminal alkynes bearing electro‐withdrawing groups at the para‐position of benzene rings, such as ‐F, ‐Cl, ‐CF_3_, ‐CN, ‐NO_2_, ‐CHO and ‐COOMe, were all tolerated with this reaction conditions and afforded the corresponding products (2b–2 h) with excellent yields (85–95%). Interestingly, aromatic terminal alkyne (1i) bearing ester substituent group at *para*‐position, introduced a much better yield (2i, 93%) than the *meta*‐alkyne (2j, 40%). Furthermore, the substrates with electro‐donating groups at the benzene rings were evaluated and led to the products (2k‐2n) with moderate yields (55–76%). Moreover, naphthyl and N‐heterocyclic terminal alkynes, such as pyridine, quinoline and pyrimidine substituents, were well accommodated under the standard conditions, providing 2o‐2t with 40–94% yields. In addition, in order to further expand the applicability of this method, various aliphatic terminal alkynes were examined. Gratifyingly, the long‐chain terminal alkynes 1u also could work to deliver the product (2u) with 39% yield. Encouraged by this result, several simple aliphatic terminal alkynes (1v‐1y) were employed with the optimized conditions and gave the olefins up to 91% yield. Furthermore, several bioactive derivatives containing terminal alkynes were tested with this method. To our delight, these molecules were still applicable with the present conditions, providing 2z‐2ab in 61–89% yield. In general, Bu_4_NCl as a catalyst was first introduced in the hydrogenation of terminal alkynes to olefins, and most of alkynes were tolerated with this methodology Figure S1.

**Table 1:** Optimization of hydrogenation conditions of alkynes to olefins.

Entry^a^ ^)^	R_4_NX	Pressure (MPa)	Solvent	Yield [%]
1	–	5.0	Toluene	ND
2	Bu_4_NCl	5.0	Toluene	93
3	Bu_4_NCl	0.5	Toluene	91
4	Bu_4_NCl	balloon	Toluene	48
5^b^ ^)^	Bu_4_NCl	5.0	Toluene	39
6	Bu_4_NCl	0.5	THF	92
7	Bu_4_NCl	0.5	DCE	89
8	Bu_4_NCl	0.5	1,4‐Dioxane	86
9	Bu_4_NCl	0.5	DMF	78
10^c^ ^)^	Bu_4_NCl	0.5	THF	87
11	Bu_4_NBr	0.5	THF	88
12	Bu_4_NI	0.5	THF	68
13	Bu_4_NBF_4_	0.5	THF	61
14	Bu_4_NOTf	0.5	THF	Trace
15	Me_4_NI	0.5	THF	Trace
16	Me_4_NCl	0.5	THF	Trace

^a)^ All reactions were performed with 1a (0.2 mmol) in 2 mL solvent at 80 °C for 72 h. Yields are those for the isolated products;

^b)^ Reaction ran 48 h under 5 MPa;

^c)^ Reaction was carried out at 60 °C.

**Scheme 3:**
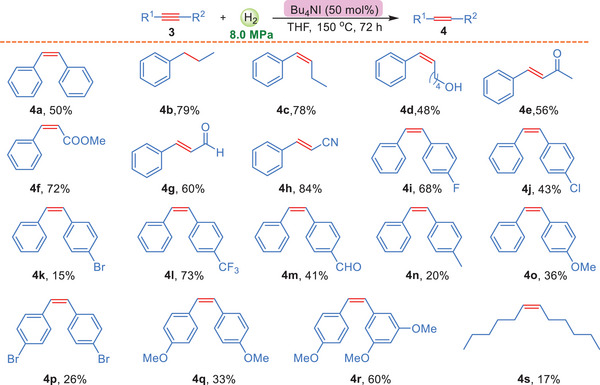
Hydrogenation of internal alkynes to olefins. All reactions were performed with 3 (0.2 mmol), pressure of H_2_ (8.0 MPa), in 2 mL THF at 150 °C for 72 h. Yields are those for the isolated products.

Although the terminal alkynes were successfully reduced by Bu_4_NCl, the hydrogenation of internal alkynes posed a greater challenge. The initial experiment was carried out by using 3a as substrate and the result showed that almost no corresponding internal olefin (4a) was observed under the optimized conditions in Table 1. Therefore, a new selection of reaction conditions for internal alkyne was performed. The results indicated that Bu_4_NI was a better catalyst and the reaction could work well in THF at 150 °C under a high pressure (8.0 MPa) (the detailed selections were shown in the supporting information). Consequently, the internal olefin (4a) was obtained in 50% yield under the conditions. Afterward, various internal alkynes were examined and the results were shown in **Scheme** **3**. Interestingly, the internal alkyne (3b) was directly transformed to alkane 4b with 79% yield and no olefin was detected. On the contrary, this strategy could lead to 4c in 78% yield for the similar substrate 3c. Furthermore, several internal alkynes bearing functional groups, such as ‐CH_2_OH, ‐COCH_3_, ‐CHO, ‐COOEt and ‐CN, were all tolerated, giving their hydrogenated olefins (4d‐4 h) with 48–84% yields. In order to further explore the influence of various substituent groups on the substrate 3a, a wide range of substrates 3i‐3r were tested. Consequently, substrates with strong electron‐withdrawing groups could furnish moderate yields, such as 4i and 4l. However, it is obvious to find that substrates with electron‐donating substituent groups usually gave low yields, besides 3r. In addition, the long‐chain internal alkynes 3s still could work, but with only 17% yield. Overall, these findings demonstrate that the hydrogenation of internal alkynes using Bu_4_NI as the catalyst can yield a range of products depending on the nature of the substituent groups. The results also highlight the importance of quaternary ammonium salts on the reactivity of the terminal and internal alkynes.

**Scheme 4:**
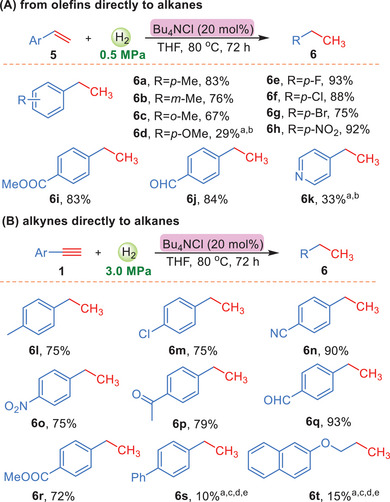
A) Hydrogenation from olefins directly to alkanes. B) Hydrogenation from alkynes directly to alkanes. ^a^50 mol% nBu_4_NCl was used. ^b^The reaction temperature was 100 °C. ^c^the reaction temperature was 150 °C. ^d^Pressure of H_2_ (7.0 MPa). ^e^50 mol% nBu_4_NI was used.

The hydrogenation processes catalyzed by quaternary ammonium salts from alkynes to olefines have been discussed. Moreover, it is also quite interesting to explore the catalytic hydrogenation ability of quaternary ammonium salts from olefins to alkanes. Notably, as the study develops in depth, we found that when the amount of Bu_4_NCl was increased to 20 mol%, the aromatic terminal olefins could be successfully reduced to alkanes (the detailed selections were shown in the supporting information). As shown in **Scheme** **4**A, different aromatic terminal olefins, no matter with electron‐withdrawing or electron‐donating substituent groups, could react smoothly to afford the alkanes with good yields (6a–6k). Furthermore, we turned our attention to the direct hydrogenation from alkynes to alkanes. However, the mixtures of alkynes and alkanes were obtained under the current conditions. Interestingly, when the pressure of H_2_ increased to 3.0 MPa, the terminal alkynes could be transferred to their corresponding alkanes (the detailed selections were shown in the supporting information). For example, several aromatic terminal alkynes were successfully hydrogenated and furnished the alkanes up to 93% yield (Scheme 4B). Unfortunately, this hydrogenation conditions described were not effective for internal alkynes and olefins.

**Scheme 5:**
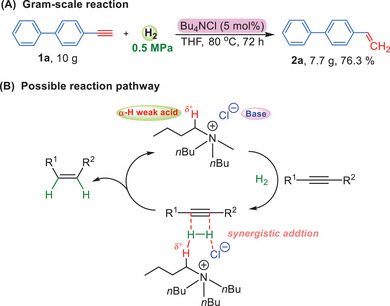
A) Gram‐scale reaction. B) Possible reaction pathway.

In order to further explore the application of this methodology, the gram‐scale reaction (10 g) was performed and generated 2a in 76.3% yield (**Scheme** **5**A). Encouraged by the unexpected catalytic ability of quaternary ammonium salts, what we are mostly interested in is the role of catalyst during the whole reaction. As we know that the quaternary nitrogen is a strong withdrawing group which can increase the acidity of α‐hydrogen. Moreover, it was reported that tetrabutylammonium cation and halide anion could work synergistically as a bifunctional catalyst to active substrates.^[^ ^11^ ^]^ Considering the successful experience of FLPs, we suggest a possible mechanism in Scheme 5B. The Cl^−^ serving as a good Lewis base together with the α‐H (weak acid) on the butyl chains probably form a frustrated Lewis pair which can activate the H—H bond. It was reported that the frustrated Lewis pair usually activated hydrogen and received H^+^ and H^−^ ions. However, considering that all the products obtained in this work were *cis*‐structures (4e, 4 g, and 4 h obtained from *Z* to *E*), the mechanism could be different with FLPs. Therefore, we suggest that the hydrogen is activated by quaternary ammonium salts, and the H‐H is added to the unsaturated bond via a synergistic addition process (Scheme 5B). After that, the formation of the *cis*‐olefin is observed and the catalyst continues to run in next cycle. This mechanism provides a plausible explanation for the observed results and suggests new insights into the catalytic behavior of quaternary ammonium salts in this transformation.

## 3 Conclusion

In conclusion, we have developed a new methodology for the hydrogenation of alkynes and olefins using molecular hydrogen. Importantly, the most common and inexpensive quaternary ammonium salts were first discovered to possess the catalytic ability to reduce multiple C—C bonds. In addition, this catalytic strategy only work on the C=C or C≡C and the high reactive C=N, C=O, C—Br bonds are not suitable for the catalyst. Interestingly, the reduction capacity of this new methodology can be controlled by changing the reaction conditions, such as the pressure of hydrogen and the amount of catalyst. The new applications and detailed mechanism of this new methodology are under exploration. In general, this new hydrogenation method is a significant and valuable discovery, with immense potential for various applications in the chemical industry. Its cost‐effectiveness, versatility, and ability to selectively target C—C bonds make it an attractive alternative to traditional metal‐based catalysts.

## Conflict of Interest

The authors declare no conflict of interest.

## Supporting information

Supporting Information

## Data Availability

The data that support the findings of this study are available from the corresponding author upon reasonable request.
