# Supplementary material for: Hydrogenation of Alkynes and Olefins Catalyzed by Quaternary Ammonium Salts
Source: Adv Sci (Weinh). 2023 Dec 10;11(7):2305271. doi: 10.1002/advs.202305271 (PMC10870019; doi:10.1002/advs.202305271)
Supplement: Supplementary file 1 — Supporting Information [file ADVS-11-2305271-s001.pdf]

ADVANCED SCIENCE
Open Access

# Supporting Information

for *Adv. Sci.*, DOI 10.1002/advs.202305271

Hydrogenation of Alkynes and Olefins Catalyzed by Quaternary Ammonium Salts

*Qi Guo, Guoli Shen, Guangfu Lu, Jinyi Qian, Qitao Que, Jiuling Li, Yafei Guo* and Baomin Fan**

Supporting Information

# Hydrogenation of alkynes and olefins catalyzed by quaternary ammonium salts

Qi Guo,[+] Guoli Shen,[+] Guangfu Lu,[+] Jinyi Qian, Qitao Que, Jiuling Li, Yafei Guo,* and Baomin Fan*

Yunnan Key Laboratory of Chiral Functional Substance Research and Application, School of Chemistry & Environment, Yunnan Minzu University, 2929 Yuehua road, Kunming 650500, China

Email: gyfwin@sina.com; FanBM@ynni.edu.cn

[+]These authors contributed equally to this work.

# *Table of Contents*

1. General Information ........................................................................................3

2. Optimization of reaction conditions from olefins to alkanes ......................................4

3. Optimization of reaction conditions from alkynes to alkanes .................................... 4

4. Optimization of reaction conditions from internal alkynes to internal olefins ............5

5. Preparation of alkynes ........................................................................................ 7

6. General procedures for the hydrogenation process ................................................. 11

7.The gram-scale reaction .......................................................................................12

8. Characterization data for the products ...................................................................13

9. ICp-Ms Test Report ............................................................................................33

10. NMR spectra for the products ...........................................................................34

11. References ........................................................................................................96

## 1. General Information

All commercially available reagents were obtained from commercial suppliers and used without further purification. Chromatography was carried out over silica gel (Innochem 200-300 mesh) with petroleum ether and ethyl acetate as eluents, and TLC was performed using silica gel 60 F254 (Merck) plates. $^{1}H$ NMR (400 MHz) and $^{13}C$ NMR (100 MHz) spectra were recorded on a Bruker NMR spectrometer in $CDCl_3$ using TMS as an internal reference with chemical shift values reported in ppm. Abbreviations used in the NMR follow-up experiments: s, singlet; d, doublet; t, triplet; q, quartet; m, multiplet. High-resolution mass spectra (HRMS) were obtained by fast atom bombardment (FAB) using a double focusing magnetic sector mass spectrometer and electron impact (EI) ionization technique. Inductively Coupled Plasma-Mass Spectrometry (ICP-MS) spectra were recorded on Plasma Quant PQ9000.

## 2. Optimization of reaction conditions from olefins to alkanes[a]

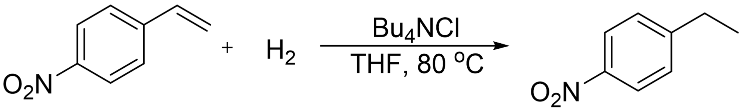


**5h** **6h**

| Entry | $Bu_4NCl$ (mol%) | Time (h) | Yield (%)[b] |
|---|---|---|---|
| 1 | 10 | 72 | / |
| 2 | 15 | 72 | 43 |
| 3 | 20 | 72 | 92 |

[a]Reaction conditions: **5h** (0.2 mmol) in THF (2mL) at 80 °C for 72 h. The reaction mixture was stirred in hydrogen atmosphere of 0.5 MPa. [b] Isolated yield.

## 3. Optimization of reaction conditions from alkynes to alkanes[a]

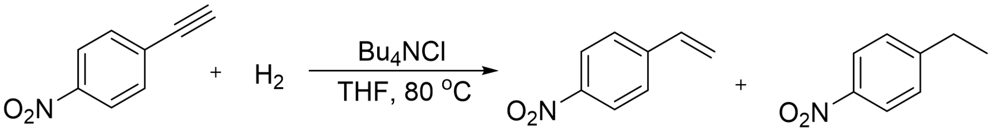


**1f** **2f** **6h**

| Entry | $Bu_4NCl$ (mol%) | Pressure (MPa) | Time (h) | **2f** Yield (%)[b] | **6h** Yield (%)[b] |
|---|---|---|---|---|---|
| 1 | 10 | 0.5 | 72 | 92 | / |
| 2 | 15 | 0.5 | 72 | 93 | / |
| 3 | 20 | 0.5 | 72 | 52 | 38 |
| 4 | 25 | 0.5 | 72 | 14 | 78 |
| 5 | 20 | 1.0 | 72 | 54 | 43 |
| 6 | 20 | 2.0 | 72 | 27 | 69 |
| 7 | 20 | 3.0 | 72 | 5 | 86 |

[a]Reaction conditions: **1f** (0.2 mmol) in THF(2mL) at 80 °C for 72 h. The reaction mixture was stirred in hydrogen atmosphere of 0.5-3.0 MPa [b] Isolated yield.

## 4. Optimization of reaction conditions from internal alkynes to internal olefins[a]

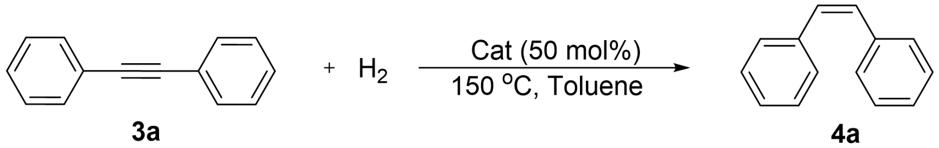


| Entry | Cat. (50%) | Time (h) | Yield (%) |
|---|---|---|---|
| 1 | **Cat 1** | 12 | NR |
| 2 | **Cat 2** | 12 | NR |
| 3 | **Cat 3** | 12 | 8 |
| **4** | **Cat 4** | **12** | **30** |
| 5 | **Cat 5** | 12 | 3 |
| 6 | **Cat 6** | 12 | < 1 |
| 7 | **Cat 7** | 12 | NR |
| 8 | **Cat 8** | 12 | NR |
| 9 | **Cat 9** | 12 | NR |
| 10 | **Cat 10** | 12 | NR |
| 11 | **Cat 11** | 12 | NR |
| 12 | **Cat 12** | 12 | NR |
| 13 | **Cat 13** | 12 | NR |
| 14 | **Cat 14** | 12 | 27 |
| 15 | **Cat 15** | 12 | 8 |
| 16 | **Cat 16** | 12 | NR |
| 17 | **Cat 17** | 12 | NR |
| 18 | **Cat 18** | 12 | 27 |
| 19 | **Cat 19** | 12 | NR |
| 20 | **Cat 20** | 12 | 6 |
| 21 | **Cat 21** | 12 | NR |
| 22 | **Cat 22** | 12 | NR |
| 23 | **Cat 23** | 12 | NR |
| 24 | **Cat 24** | 12 | 26 |
| 25 | **Cat 25** | 12 | 14 |
| 26 | **Cat 26** | 12 | 18 |
| 27 | **Cat 27** | 12 | NR |

[a]Reaction conditions: **3a** (0.2 mmol) in Toluene (2mL) at 150 °C for 12 h. The reaction mixture was stirred in hydrogen atmosphere of 8.0 MPa. [b] Isolated yield.

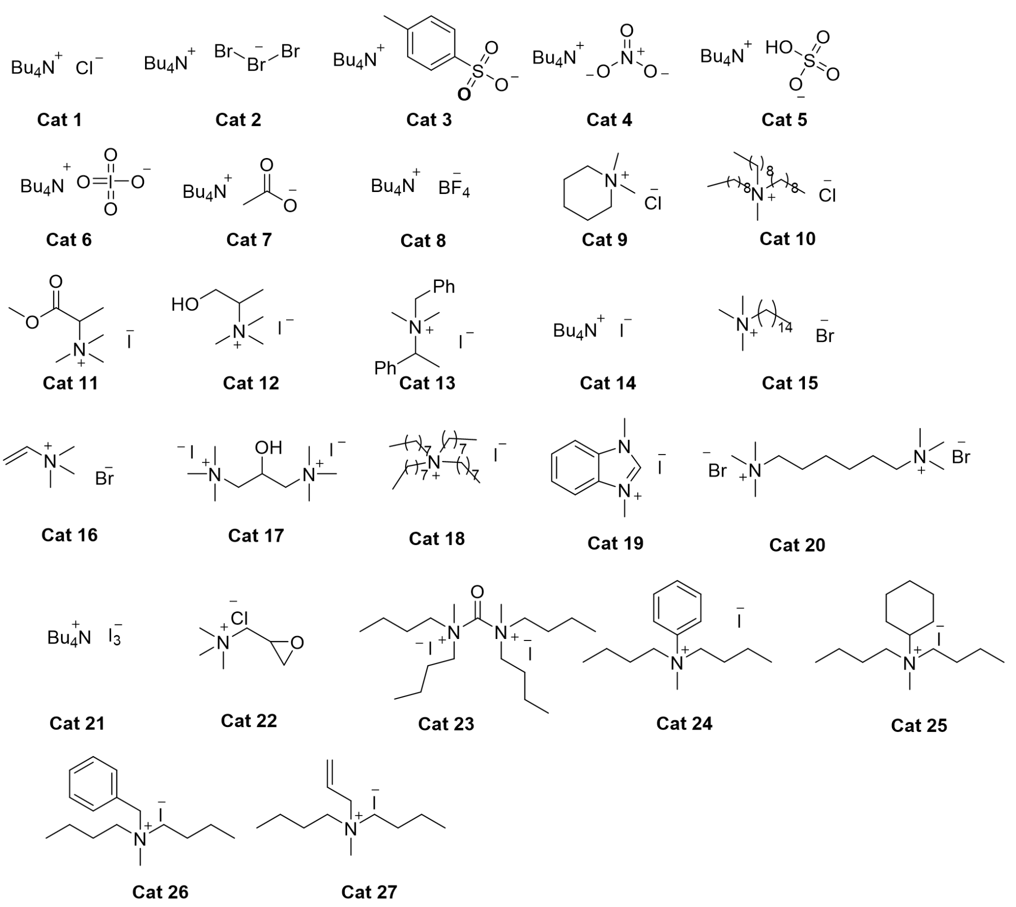


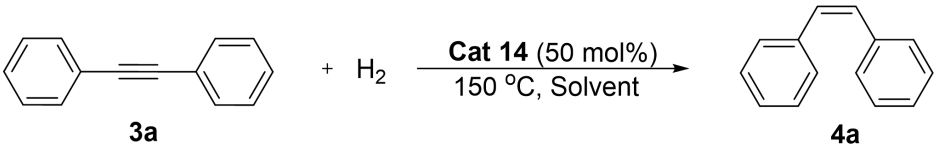


| Entry | Solvent | Time (h) | Yield (%) |
|---|---|---|---|
| 1 | Toluene | 12 | 27 |
| 2 | THF | 12 | 47 |
| 3 | 1,4-Dioxane | 12 | < 1 |
| 4 | DMF | 12 | 6 |
| 5 | EtOH | 12 | NR |
| 6 | ACN | 12 | < 1 |
| 7 | DCE | 12 | NR |
| 8 | Octane | 12 | < 1 |
| 9 | THF | 48 | 47 |

$^a$Reaction conditions: **3a** (0.2 mmol) in Toluene (2mL) at 150 °C for 12 h. The reaction mixture was stirred in hydrogen atmosphere of 8.0 MPa. $^b$ Isolated yield.

# 5. Preparation of Alkynes

Procedure A:

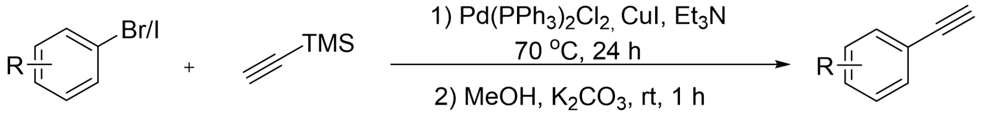


To a mixture of aryl bromide or aryl iodide (3 mmol, 1.0 equiv.), $Pd(PPh_3)_2Cl_2$ (3 mol%), and CuI (8 mol%) in 30 mL of triethylamine. (trimethylsilyl) acetylene (1.5 equiv.) was added and the mixture was stirred at 70 °C for 24 h. After that, the reaction mixture was cooled to room temperature, diluted with $Et_2O$, quenched with saturated $NH_4Cl$, and the aqueous layer was extracted with $Et_2O$ (3 times). The combined organic layer was washed with brine, dried over anhydrous $Na_2SO_4$, and concentrated to afford the crude product. To the crude product was added MeOH (20 mL) and $K_2CO_3$ (3.0 equiv.), the resulting mixture was stirred at rt for 1 h, and neutralized with a HCl (1.0 M) solution. The organic layer was washed with water and brine, dried over anhydrous $Na_2SO_4$, and concentrated. The resulting residue was purified by flash column chromatography on silica gel.

Procedure B:

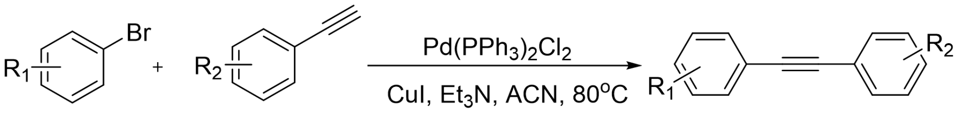


The corresponding aryl bromide (1 equiv.), $Pd(PPh_3)_2Cl_2$ (3 mol%), CuI (2 mol%) and phenylacetylene (1.1 equiv.) were added to a 50 mL Schlenk flask with a stir bar under nitrogen atmosphere. Then

tetrahydrofuran (5 mL) and triethylamine (5 mL) were added sequentially. The reaction mixture was then stirred at 80 °C overnight. Afterwards, 10 mL of water was added and the reaction mixture was extracted with EtOAc (3 × 10 mL). The combined organic fractions were washed with brine and dried over $Na_2SO_4$. After filtration, the solvent was removed under reduced pressure. The residue was purified by chromatography on silica gel, eluting with the mixture of petroleum ether or ethyl acetate/petroleum ether to give internal alkyne substrates. The yields were not optimized for the synthesis of alkynes.

**(8S,14S)-3-(pent-4-yn-1-yloxy)-6,7,8,9,11,12,13,14,15,16-decahydro-17H-cyclopenta[a]phenanthren-17-one (1ab)**

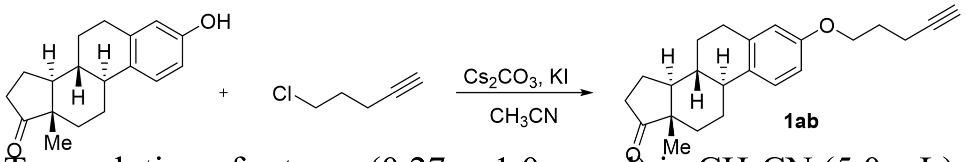


To a solution of estrone (0.27 g, 1.0 mmol) in $CH_3CN$ (5.0 mL) were added $Cs_2CO_3$ (0.49 g, 1.5 mmol) and KI (17 mg, 0.10 mmol) at room temperature, and 5-Cl-1-pentyne (0.12 g, 1.2 mmol) was added. The reaction was warmed to 85 °C and stirred until full consumption of the starting materials. The reaction was quenched with water and extracted with ethyl acetate (3×20 mL). The combined organic layers were washed with brine and dried over anhydrous $Na_2SO_4$. The solution was concentrated in vacuo, and the residue was purified by flash chromatography on silica gel (petroleum ether/ethyl acetate = 4:1) to afford **1ab** 315 mg, as a white solid, 87% yield.

**$^1$H NMR** (400 MHz, Chloroform-d) δ 7.20 (d, $J$ = 8.6 Hz, 1H), 6.72 (dd, $J$ = 8.6, 2.9 Hz, 1H), 6.66 (d, $J$ = 2.7 Hz, 1H), 4.07 – 4.01 (m, 2H), 2.95 – 2.85 (m, 2H), 2.51 (dd, $J$ = 18.9, 8.6 Hz, 1H), 2.44 – 2.35 (m, 3H), 2.30 –

2.20 (m, 1H), 2.19 – 1.91 (m, 8H), 1.67 – 1.38 (m, 7H), 0.91 (d, *J* = 1.4 Hz, 3H).

**$^{13}$CNMR** (101 MHz, Chloroform-d) δ 156.8, 137.7, 132.1, 126.4, 114.53, 112.1, 83.6, 68.8, 66.0, 50.4, 48.0, 43.9, 38.4, 35.9, 31.6, 29.7, 28.2, 26.5, 25.9, 21.6, 15.2, 13.9.

**HRMS** calcd for $C_{24}H_{30}O_2[M+H]^+$:351.2319. Found:350.2315.

## coumarin alkyne (1y)

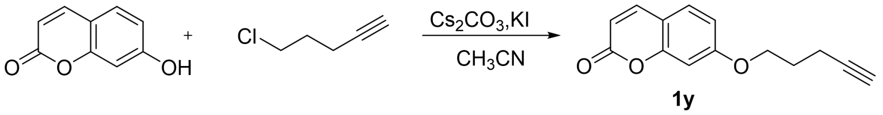


To a solution of 7-Hydroxycoumarin (0.16 g, 1.0 mmol) in $CH_3CN$ (5.0 mL) were added $Cs_2CO_3$ (0.49 g, 1.5 mmol) and KI (17 mg, 0.10 mmol) at room temperature, and 5-Chloro-1-pentyne (0.12 g, 1.2 mmol) was added. The reaction was warmed to 85°C and stirred until full consumption of the starting materials. The reaction was quenched with water and extracted with ethyl acetate (3×20 mL). The combined organic layers were washed with brine and dried over anhydrous $Na_2SO_4$. The solution was concentrated in vacuo, and the residue was purified by flash chromatography on silica gel (petroleum ether/ethyl acetate = 4:1) to afford **1y** 180.18mg, as a white solid, 79% yield;

**$^1$H NMR** (400 MHz, Chloroform-*d*) δ 7.64 (d, *J* = 9.5 Hz, 1H), 7.37 (d, *J* = 8.5 Hz, 1H), 6.88 – 6.79 (m, 2H), 6.25 (d, *J* = 9.4 Hz, 1H), 4.13 (t, *J* = 6.1 Hz, 2H), 2.45-2.39 (m, 2H), 2.06 – 2.01 (m, 2H), 1.99 (t, *J* = 2.7 Hz, 1H).

**$^{13}$C NMR** (101 MHz, Chloroform-*d*) δ 162.1, 161.2, 155.8, 143.4, 128.7, 113.0, 112.8, 112.5, 101.3, 82.9, 69.2, 66.6, 27.7, 15.0.

**HRMS** calcd for $C_{14}H_{12}O_3[M+H]^+$:229.0859. Found:229.0860.

**(3,5-dibromo-4-(prop-2-yn-1-yloxy)phenyl)(2-ethylbenzofuran-3-yl)methanone (1aa)**

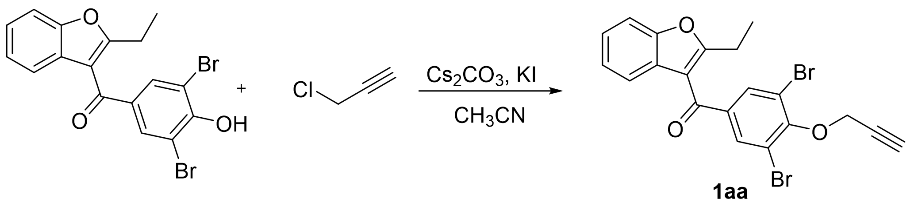

1aa

To a solution of benzbromarone (0.42 g, 1.0 mmol) in $CH_3CN$ (5.0 mL) were added $Cs_2CO_3$ (0.49 g, 1.5 mmol) and KI (17 mg, 0.10 mmol) at room temperature, and 3-Bromopropyne (0.14g, 1.2 mmol) was added. The reaction was warmed to 85 °C and stirred until full consumption of the starting materials. The reaction was quenched with water and extracted with ethyl acetate (3×20 mL). The combined organic layers were washed with brine and dried over anhydrous $Na_2SO_4$. The solution was concentrated in vacuo, and the residue was purified by flash chromatography on silica gel (petroleum ether/ethyl acetate = 5:1) to afford **1aa** 213.8mg, as a white solid, 45% yield;

**$^1$H NMR** (400 MHz, Chloroform-*d*) δ 7.99 (s, 2H), 7.51 (m, *J* = 8.2, 0.9 Hz, 1H), 7.41 (m, *J* = 7.7, 1.4, 0.7 Hz, 1H), 7.32 (m, *J* = 8.2, 7.3, 1.4 Hz, 1H), 7.27 – 7.23 (m, 1H), 4.89 (d, *J* = 2.5 Hz, 2H), 2.90 (m, *J* = 7.5 Hz, 2H), 2.60 (t, *J* = 2.5 Hz, 1H), 1.36 (t, *J* = 7.5 Hz, 3H).

**$^{13}$C NMR** (101 MHz, Chloroform-*d*) δ 188.1, 167.1, 155.3, 153.6, 137.7, 133.5, 126.3, 124.8, 123.9, 121.0, 119.1, 115.3, 111.2, 60.7, 22.0, 12.2.

**HRMS** calcd for $C_{20}H_{14}Br_2O_3[M+H]^+$: 460.9382. Found:460.9378.

# 6. General procedures for hydrogenation process

## General experiment equipment

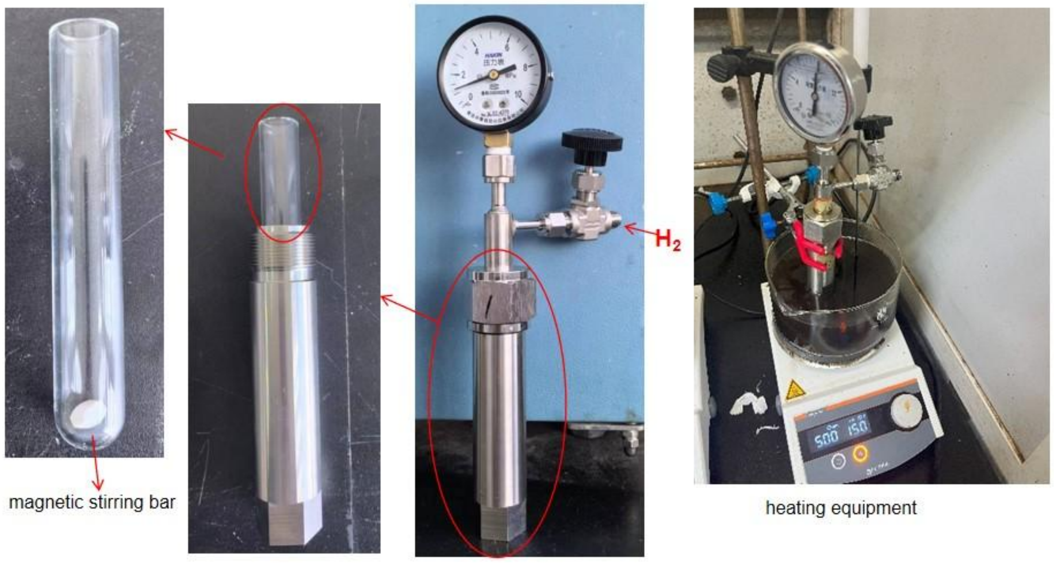


experiment equipment

### From alkynes to olefins

Alkynes (0.2 mmol), $Bu_4NCl$ (0.01 mmol, 5 mol%) in THF (2 mL) were added to a pressure resistant vessel equipped with a magnetic stir bar and the resulting solution was stirred at 80 °C for 72 h under $H_2$ (0.5 MPa). After that, the desired product was purified by chromatography on silica gel column to obtain the desired product olefins.

### From alkyne to alkanes

Alkynes (0.2 mmol), $Bu_4NCl$ (0.04 mmol, 20 mol%) in THF (2 mL) were added to a pressure resistant vessel equipped with a magnetic stir bar and the resulting solution was stirred at 80 °C for 72 h under $H_2$ (3.0

MPa). After that, the desired product was purified by chromatography on silica gel column to obtain the desired product alkanes.

**From olefins to alkanes**

Olefins (0.2 mmol), $Bu_4NCl$ (0.04 mmol, 20 mol%) in THF (2 mL) were added to a pressure resistant vessel equipped with a magnetic stir bar and the resulting solution was stirred at 80 °C for 72 h under $H_2$ (0.5 MPa). After that, the desired product was purified by chromatography on silica gel column to obtain the desired product alkanes.

**Hydrogenation of Internal Alkynes**

Internal alkynes (0.2 mmol), $Bu_4NI$ (0.1 mmol, 50 mol%) in THF (2 mL) were added to a pressure resistant vessel equipped with a magnetic stir bar and the resulting solution was stirred at 150 °C for 72 h under $H_2$ (8.0 MPa). After that, the desired product was purified by chromatography on silica gel column to obtain the desired product internal olefins.

## 7. The gram-scale reaction

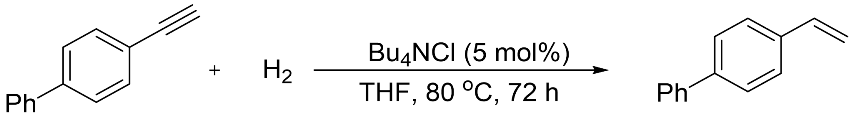


**1a**, 10g　　**2a**

**1a** (10g, 56 mmol), $Bu_4NCl$ (778mg, 2.8 mmol) in THF (50 mL) were added to a reaction vial equipped with a magnetic stir bar, the resulting solution was stirred at 80 °C for 72 h under under $H_2$ (0.5 MPa). After that, the reaction mixture was purified by chromatography on silica gel column to obtain the desired product **2a** 7.7g, 78% yield.

## 8. Characterization data for the products

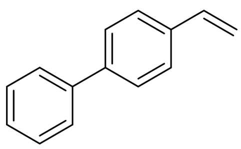

**4-vinyl-1,1'-biphenyl (2a)[1]**

White solid, 33.4 mg, 93% yield.

**$^{1}$H NMR** (400 MHz, Chloroform-*d*) δ 7.63 – 7.54 (m, 4H), 7.51 – 7.40 (m, 4H), 7.38 – 7.31 (m, 1H), 6.76 (dd, *J* = 17.6, 10.9 Hz, 1H), 5.80 (dd, *J* = 17.6, 0.9 Hz, 1H), 5.28 (dd, *J* = 10.9, 0.9 Hz, 1H).

**$^{13}$C NMR** (101 MHz, Chloroform-*d*) δ 140.7, 140.5, 136.6, 136.4, 128.7, 127.3, 127.2, 126.9, 126.6, 113.8.

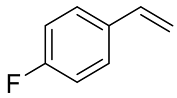


**1-fluoro-4-vinylbenzene (2b)[2]**

Yellow liquid, 20.7mg, 85% yield.

**$^{1}$H NMR** (400 MHz, Chloroform-*d*) δ 7.64 – 7.56 (m, 2H), 7.31 – 7.21 (m, 2H), 6.92 (dd, *J* = 17.6, 10.9 Hz, 1H), 5.92 (d, *J* = 17.6 Hz, 1H), 5.47 (dd, *J* = 10.9, 0.9 Hz, 1H).

**$^{13}$C NMR** (101 MHz, Chloroform-*d*) δ 163.8, 161.4, 135.8, 133.9, 133.9, 127.9, 127.8, 115.6, 115.3, 113.5, 113.4.

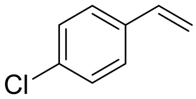


**1-chloro-4-vinylbenzene (2c)[3]**

Brown liquid, 24.88mg, 85% yield.

**$^{1}$H NMR** (400 MHz, Chloroform-*d*) δ 7.34 – 7.14 (m, 4H), 6.62 (dd, *J* = 17.6, 10.9 Hz, 1H), 5.68 (d, *J* = 17.6 Hz, 1H), 5.23 (d, *J* = 10.9 Hz, 1H)

**$^{13}$C NMR** (101 MHz, Chloroform-d) δ 142.5, 131.2, 129.2, 128.3, 28.2, 15.5.

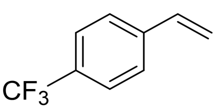

**1-(trifluoromethyl)-4-vinylbenzene (2d)[2]**

Yellow liquid, 20.7mg, 85% yield.

**$^{1}$H NMR** (400 MHz, Chloroform-d) δ 7.72 (d, *J* = 8.3 Hz, 2H), 7.56 (d, *J* = 8.2 Hz, 2H), 6.84 (dd, J = 17.7, 10.9 Hz, 1H), 5.97 (d, *J* = 17.6 Hz, 1H), 5.52 (d, *J* = 10.9 Hz, 1H).

**$^{13}$C NMR** (101 MHz, Chloroform-d) δ 140.9, 140.9, 135.6, 129.8, 129.4, 126.3, 125.6, 125.5, 125.5, 125.4, 125.4, 122.9, 116.3.

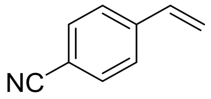


**4-vinylbenzonitrile (2e)[3]**

Yellow liquid, 20.8 mg, 81% yield.

**$^{1}$H NMR** (400 MHz, Chloroform-*d*) δ 7.62 – 7.56 (m, 2H), 7.48 – 7.44 (m, 2H), 6.70 (dd, *J* = 17.6, 10.9 Hz, 1H), 5.88 (s, 1H), 5.43 (d, *J* = 10.9 Hz, 1H).

**$^{13}$C NMR** (101 MHz, Chloroform-*d*) δ 141.8, 135.3, 132.3, 126.7, 118.9, 117.7, 111.0.

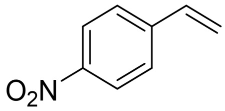


**1-nitro-4-vinylbenzene (2f)[3]**

Brown solid, 28.3 mg, 95% yield.

**$^{1}$H NMR** (400 MHz, Chloroform-*d*) δ 8.22 – 8.14 (m, 2H), 7.56 – 7.51 (m, 2H), 6.77 (dd, *J* = 17.6, 10.9 Hz, 1H), 5.92 (d, *J* = 17.6 Hz, 1H), 5.49 (d, *J* = 10.9 Hz, 1H).

**$^{13}$C NMR** (101 MHz, Chloroform-*d*) δ 147.1, 143.8, 134.9, 126.8, 123.9, 118.6.

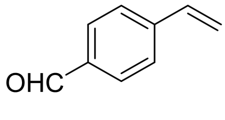

**4-vinylbenzaldehyde (2g) [4]**

Yellow liquid, 22.7mg, 86% yield.

**$^1$H NMR** (400 MHz, Chloroform-*d*) δ 9.99 (d, *J* = 1.2 Hz, 1H), 7.88 – 7.80 (m, 2H), 7.55 (dd, *J* = 8.3, 1.5 Hz, 2H), 6.77 (dd, *J* = 17.6, 11.0 Hz, 1H), 5.91 (dd, *J* = 17.4, 1.3 Hz, 1H), 5.44 (dd, *J* = 10.9, 1.3 Hz, 1H).

**$^{13}$C NMR** (101 MHz, Chloroform-*d*) δ 191.7, 143.4, 135.8, 135.6, 130.1, 126.7, 117.4.

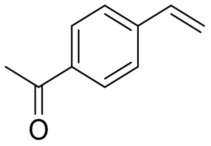


**1-(4-vinylphenyl)ethan-1-one (2h)[5]**

Yellow liquid, 27.1 mg, 93% yield.

**$^1$H NMR** (400 MHz, Chloroform-*d*) δ 7.92 (d, *J* = 8.4 Hz, 2H), 7.48 (d, *J* = 8.3 Hz, 2H), 6.75 (dd, *J* = 17.6, 10.9 Hz, 1H), 5.88 (dd, *J* = 17.6, 0.8 Hz, 1H), 5.40 (dd, *J* = 10.9, 0.7 Hz, 1H), 2.60 (s, 3H).

**$^{13}$C NMR** (101 MHz, Chloroform-*d*) δ 196.6, 141.0, 135.1, 134.8, 127.6, 125.2, 115.7, 25.6.

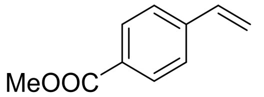


**methyl 4-vinylbenzoate(2i)[4]**

White solid, 31.4 mg, 93% yield.

**$^1$H NMR** (400 MHz, Chloroform-*d*) δ 8.02 – 7.97 (m, 2H), 7.49 – 7.44 (m, 2H), 6.75 (dd, *J* = 17.6, 10.9 Hz, 1H), 5.86 (dd, *J* = 17.6, 0.7 Hz, 1H), 5.38 (dd, *J* = 10.9, 0.8 Hz, 1H), 3.91 (s, 3H).

**$^{13}$C NMR** (101 MHz, Chloroform-*d*) δ 166.8, 141.8, 135.9, 129.8, 129.2, 126.0, 116.5, 52.1.

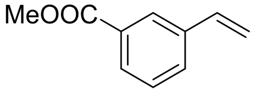


**methyl 3-vinylbenzoate(2j)[6]**

Colorless, 14.5 mg, 40% yield.

**$^1$H NMR** (400 MHz, Chloroform-*d*) δ 7.98 (dd, *J* = 1.6 Hz, 1H), 7.82 (dd, *J* = 7.7, 1.3 Hz, 1H), 7.49-7.42 (m, 1H), 7.29 (t, *J* = 7.7 Hz, 1H), 6.64 (dd, *J* = 17.6, 10.9 Hz, 1H), 5.73 (d, *J* = 17.6 Hz, 1H), 5.22 (d, *J* = 10.9 Hz, 1H), 3.82 (s, 3H).

**$^{13}$C NMR** (101 MHz, Chloroform-*d*) δ 165.8, 136.7, 134.8, 129.4, 129.3, 127.7, 127.5, 126.2, 114.0, 51.1.

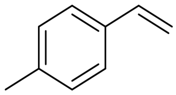

**1-methyl-4-vinylbenzene (2k)[4]**

Colorless liquid, 16.5 mg, 70% yield.

**$^1$H NMR** (400 MHz, Chloroform-*d*) δ 7.99 – 7.92 (m, 2H), 7.72 (t, *J* = 6.5 Hz, 2H), 7.35 (dd, *J* = 17.6, 10.8 Hz, 1H), 6.42 – 6.35 (m, 1H), 5.87 (t, *J* = 9.8 Hz, 1H), 2.94 (d, *J* = 3.9 Hz, 3H).

**$^{13}$C NMR** (101 MHz, Chloroform-*d*) δ 137.6, 136.7, 134.8, 129.2, 126.1, 112.8, 21.3.

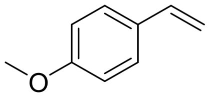

**1-methoxy-4-vinylbenzene(2i)[2]**

Yellow liquid, 20.3mg, 76% yield.

**$^1$H NMR** (400 MHz, Chloroform-*d*) δ 7.37 – 7.30 (m, 2H), 6.89 – 6.81 (m, 2H), 6.65 (dd, *J* = 17.6, 10.9 Hz, 1H), 5.60 (dd, *J* = 17.6, 0.9 Hz, 1H), 5.16 – 5.08 (m, 1H), 3.79 (s, 3H).

**$^{13}$C NMR** (101 MHz, Chloroform-*d*) δ 159.3, 136.2, 130.4, 127.3, 113.8, 111.5, 55.2.

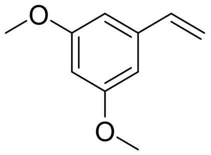


**1,3-dimethoxy-5-vinylbenzene (2m)[7]**

Colorless liquid, 18.0 mg, 55% yield.

**$^1$H NMR** (400 MHz, Chloroform-*d*) δ 6.65 (dd, *J* = 17.5, 10.8 Hz, 1H), 6.57 (d, *J* = 2.3 Hz, 2H), 6.39 (t, *J* = 2.3 Hz, 1H), 5.73 (d, *J* = 17.5 Hz, 1H), 5.25 (d, *J* = 10.8 Hz, 1H), 3.81 (s, 6H).

**$^{13}$C NMR** (101 MHz, Chloroform-*d*) δ 160.9, 139.6, 136.8, 114.3, 104.3, 100.1, 55.3.

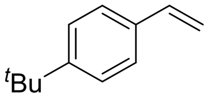


**1-(tert-butyl)-4-vinylbenzene (2n)[8]**

Yellow liquid, 21.1mg, 66% yield.

**$^1$H NMR** (400 MHz, Chloroform-*d*) δ 7.38 (s, 4H), 6.72 (dd, *J* = 17.6, 10.9 Hz, 1H), 5.73 (dd, *J* = 17.6, 1.1 Hz, 1H), 5.21 (dd, *J* = 10.8, 1.0 Hz, 1H), 1.34 (s, 9H).

**$^{13}$C NMR** (101 MHz, Chloroform-*d*) δ 150.8, 136.6, 134.8, 125.9, 125.4, 112.9, 34.5, 31.3.

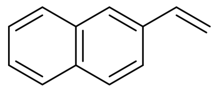

**2-vinylnaphthalene (2o)[5]**

White solid, 29.0 mg, 94% yield.

**$^1$H NMR** (400 MHz, Chloroform-*d*) δ 7.86 – 7.73 (m, 4H), 7.66 (dd, *J* = 8.6, 1.8 Hz, 1H), 7.52 – 7.41 (m, 2H), 6.90 (dd, *J* = 17.6, 10.9 Hz, 1H), 5.89 (dd, *J* = 17.6, 0.8 Hz, 1H), 5.35 (dd, *J* = 10.9, 0.8 Hz, 1H).

**$^{13}$C NMR** (101 MHz, Chloroform-*d*) δ 136.8, 134.9, 133.4, 133.0, 128.1, 128.0, 127.6, 126.3, 126.2, 125.8, 123.0, 114.1.

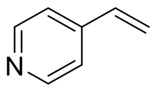


**2-vinylpyridine (2p)[2]**

Brown liquid, 9.9mg, 47% yield.

**¹H NMR** (400 MHz, Chloroform-d) δ 8.58 – 8.49 (m, 2H), 7.26 – 7.17 (m, 2H), 6.75 – 6.42 (m, 1H), 5.93 (dd, *J* = 17.6, 5.2 Hz, 1H), 5.45 (dd, *J* = 10.8, 5.2 Hz, 1H).
**¹³C NMR** (101 MHz, Chloroform-d) δ149.9, 144.3, 134.4, 120.5, 118.4.

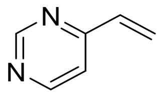

**4-vinylpyrimidine (2q)[4]**

Yellow liquid, 27.9 mg, 90% yield.
**¹H NMR** (400 MHz, Chloroform-d) δ 9.08 (s, 1H), 8.75 (s, 2H), 6.64 (dd, *J* = 17.8, 11.1 Hz, 1H), 5.92 (d, *J* = 17.7 Hz, 1H), 5.50 (d, *J* = 11.1 Hz, 1H).
**¹³C NMR** (101 MHz, Chloroform-d) δ 157.5, 154.2, 154.1, 130.9, 130.0, 118.6.

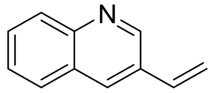

**3-vinylquinoline (2r)[8]**

Colorless liquid, 26.9 mg, 87% yield.
**¹H NMR** 1H NMR (400 MHz, Chloroform-d) δ 9.02 (d, *J* = 2.2 Hz, 1H), 8.13 – 8.04 (m, 2H), 7.79 (dd, *J* = 8.1, 1.4 Hz, 1H), 7.72 – 7.63 (m, 1H), 7.53 (td, *J* = 8.1, 6.9, 1.2 Hz, 1H), 6.87 (dd, *J* = 17.7, 11.0 Hz, 1H), 5.98 (d, *J* = 17.6 Hz, 1H), 5.46 (d, *J* = 11.0 Hz, 1H).
**¹³C NMR** (101 MHz, Chloroform-d) δ 149.0, 147.6, 133.7, 132.5, 130.3, 129.3, 129.2, 127.9, 127.9, 126.9, 116.3.

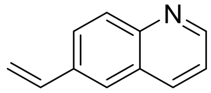

**6-vinylquinoline (2s)[9]**

Colorless liquid, 27.9 mg, 90% yield.
**¹H NMR** (400 MHz, Chloroform-d) δ 8.97 – 8.79(m, 1H), 8.11 (d, *J* = 8.5, 1.7 Hz, 1H), 8.06 (d, *J* = 8.9 Hz, 1H), 7.87 (dd, *J* = 8.8, 2.0 Hz, 1H),

7.71 (d, $J$ = 1.9 Hz, 1H), 7.38 (dd, $J$ = 8.3, 4.2 Hz, 1H), 6.89 (dd, $J$ = 17.6, 10.9 Hz, 1H), 5.90 (d, $J$ = 17.6 Hz, 1H), 5.40 (d, $J$ = 11.0 Hz, 1H).
**$^{13}$C NMR** (101 MHz, Chloroform-d) δ 150.2, 148.1, 136.1, 136.0, 135.7, 129.6, 128.4, 126.9, 125.8, 121.4, 115.4.

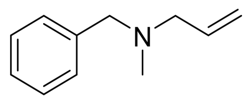

***N*-benzyl-N-methylprop-2-en-1-amine (2t)[10]**

Colorless liquid, 12.8 mg, 40% yield.
**$^{1}$H NMR** (400 MHz, Chloroform-d) δ 7.34 – 7.20 (m, 5H), 5.87 – 5.81 (m, 1H), 5.20 – 5.04 (m, 2H), 3.42 (s, 2H), 2.96 (dd, $J$ = 6.5, 1.4 Hz, 2H), 2.12 (s, 3H).
**$^{13}$C NMR** (101 MHz, Chloroform-d) δ 137.8, 134.7, 128.0, 127.1, 125.9, 116.5, 60.6, 59.4, 41.0.

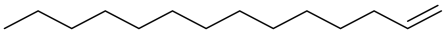

**tetradec-1-ene (2u)[11]**

Colorless liquid, 15.2 mg, 39% yield.
**$^{1}$H NMR** (400 MHz, Chloroform-d) δ 5.86 – 5.80 (m, 1H), 5.06 – 4.90 (m, 2H), 2.06 (q, $J$ = 7.0 Hz, 2H), 1.29 (d, $J$ = 6.7 Hz, 20H), 0.90 (s, 3H).
**$^{13}$C NMR** (101 MHz, Chloroform-d) δ 138.9, 114.0, 33.9, 32.0, 29.8, 29.8, 29.7, 29.6, 29.5, 29.3, 29.0, 22.7, 14.0.

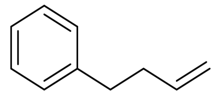

**but-3-en-1-ylbenzene (2v)[12]**

Brown liquid, 16.6 mg, 63% yield.
**$^{1}$H NMR** (400 MHz, Chloroform-d) δ 7.30 – 7.24 (m, 2H), 7.21 – 7.13 (m, 3H), 5.87 – 5.82 (m, 1H), 5.08 – 4.94 (m, 2H), 2.70 (dd, $J$ = 9.0, 6.7 Hz, 2H), 2.41 – 2.33 (m, 2H).

**$^{13}$C NMR** (101 MHz, Chloroform-d) δ 142.1, 138.3, 128.8, 128.6, 126.2, 115.2, 36.0, 35.8.

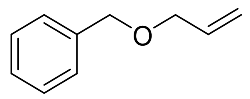

**((allyloxy)methyl)benzene (2w)[13]**

Colorless liquid, 24.0 mg, 81% yield.
**$^{1}$H NMR** (400 MHz, Chloroform-d) δ 7.36 (d, *J* = 4.3 Hz, 4H), 7.33 – 7.27 (m, 1H), 5.62 – 5.96 (m, 1H), 5.32 (dd, *J* = 17.2, 1.7 Hz, 1H), 5.22 (dd, *J* = 10.4, 1.4 Hz, 1H), 4.54 (s, 2H), 4.05 (d, *J* = 5.6, 1.5 Hz, 2H).
**$^{13}$C NMR** (101 MHz, Chloroform-d) δ 138.3, 134.7, 128.3, 127.7, 127.6, 117.1, 72.1, 71.1.

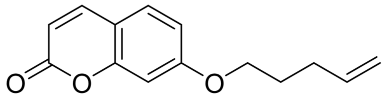

**7-(pent-4-en-1-yloxy)-2H-chromen-2-one (2y)**

White solid, 33.1 mg, 72% yield.
**$^{1}$H NMR** (400 MHz, Chloroform-d) δ 7.65 – 7.62 (m, 1H), 7.36 (d, *J* = 8.5 Hz, 1H), 6.85 – 6.73 (m, 2H), 6.24 (d, *J* = 9.5 Hz, 1H), 5.84 (td, *J* = 16.9, 10.2, 6.7 Hz, 1H), 5.09 – 4.93 (m, 2H), 4.01 (t, *J* = 6.4 Hz, 2H), 2.28 – 2.21 (m, 2H), 1.93 – 1.84 (m, 2H).
**$^{13}$C NMR** (101 MHz, Chloroform-d) δ 162.3, 161.3, 155.8, 143.5, 137.4, 128.7, 115.5, 112.9, 112.9, 112.4, 101.3, 67.7, 29.9, 28.0.
**HRMS** calcd for C14H14O3 [M+H]+: 253.0835. Found: 253.0834.

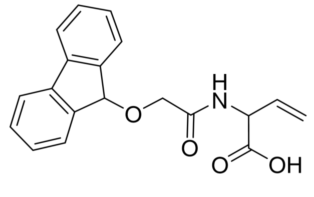

**2-((((9H-fluoren-9-yl)methoxy)carbonyl)amino)pent-4-enoic acid (2z)[14]**

White solid,47.9 mg, 71% yield.

**$^1$H NMR** (400 MHz, Chloroform-d) δ 7.77 (d, *J* = 7.5 Hz, 2H), 7.59 (dd, *J* = 7.7, 2.7 Hz, 2H), 7.40 (t, *J* = 7.4 Hz, 2H), 7.36 – 7.28 (m, 2H), 5.76 – 5.70 (m, 1H), 5.30 (d, *J* = 8.1 Hz, 1H), 5.21 – 5.17 (m, 1H), 4.54 – 4.47 (m, 1H), 4.41 (d, *J* = 7.1 Hz, 2H), 4.23 (t, *J* = 7.1 Hz, 1H), 2.67 – 2.54 (m, 2H).

**$^{13}$C NMR** (101 MHz, Chloroform-d) δ 176.3, 155.8, 143.7, 143.6, 141.2, 131.6, 127.7, 127.0, 125.0, 120.0, 119.8, 67.1, 53.0, 47.0, 36.3.

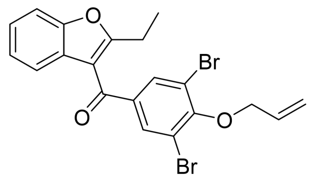


**(4-(allyloxy)-3,5-dibromophenyl)(2-ethylbenzofuran-3-yl)methanone (2aa)**

Yellow liquid, 56.4 mg, 61% yield.

**$^1$H NMR** (400 MHz, Chloroform-d) δ 7.89 (d, *J* = 2.2 Hz, 2H), 7.43 – 7.36 (m, 1H), 7.35 – 7.29 (m, 1H), 7.24 – 7.18 (m, 1H), 7.16 – 7.11 (m, 1H), 6.14 – 6.05 (m, 1H), 5.42 – 5.37 (m, 1H), 5.23 (d, *J* = 10.4, 1.2 Hz, 1H), 4.56 (dd, *J* = 6.0, 1.3 Hz, 2H), 2.80 (q, *J* = 7.5 Hz, 2H), 1.29 – 1.24 (m, 3H).

**$^{13}$C NMR** (101 MHz, Chloroform-d) δ 186.9, 186.9, 167.2, 165.8, 155.5, 155.5, 152.6, 152.6, 136.1, 134.4, 132.5, 132.4, 131.4, 125.3, 125.2, 123.6, 123.6, 122.8, 122.7, 119.9, 118.1, 117.7, 116.0, 114.4, 114.3, 110.1, 110.0, 73.3, 38.5, 32.1, 20.9, 17.6, 11.1.

**HRMS** calcd for $C_{20}H_{16}Br_2O_3$ $[M+H]^+$: 462.9539. Found:462.5943.

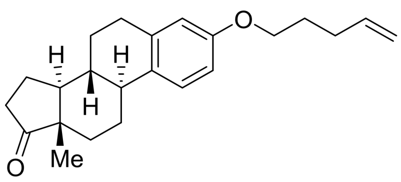

**(8R,9S,13S,14S)-13-methyl-3-(pent-4-en-1-yloxy)-6,7,8,9,11,12,13,14,15,16-decahydro-17H-cyclopenta[a]phenanthren-17-one (2ab)**

White solid, 60.1 mg, 89% yield.
$^{1}$**H NMR** (400 MHz, Chloroform-*d*) δ 7.20 (d, *J* = 8.5 Hz, 1H), 6.72 (dd, *J* = 8.6, 2.9 Hz, 1H), 6.65 (d, *J* = 2.8 Hz, 1H), 5.89 – 5.83 (m, 1H), 5.10 – 4.98 (m, 2H), 3.95 (t, *J* = 6.5 Hz, 2H), 2.93 – 2.87 (m, 2H), 2.51 (dd, *J* = 18.8, 8.6 Hz, 1H), 2.43 – 2.37 (m, 1H), 2.27 – 2.21 (m, 3H), 2.17 – 2.11 (m, 1H), 2.11 – 1.92 (m, 5H), 1.88 (dd, *J* = 8.2, 6.5 Hz, 2H), 1.70 – 1.39 (m, 9H), 0.91 (s, 3H).
$^{13}$**C NMR** (101 MHz, Chloroform-*d*) δ 157.0, 137.9, 137.7, 131.9, 126.3, 115.1, 114.5, 112.1, 112.1, 67.0, 50.4, 48.0, 44.0, 38.4, 35.9, 31.6, 30.1, 29.6, 28.5, 26.5, 25.9, 21.6, 13.8.
**HRMS** calcd for $C_{23}H_{30}O_2$ $[M+H]^+$:339.2315.Found:339.2319

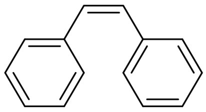

**(*Z*)-1,2-diphenylethene (4a)[15]**

Colorless liquid, 18.0mg, 50% yield.
$^{1}$**H NMR** (400 MHz, Chloroform-d) δ 7.30 – 7.11 (m, 10H), 6.59 (s, 2H).
$^{13}$**C NMR** (101 MHz, Chloroform-d) δ 137.2, 130.2, 128.8, 128.2, 127.1.

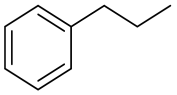

**Propylbenzene (4b)[16]**

Yellow liquid, 19.0 mg, 79% yield.

**$^1$H NMR** (400 MHz, Chloroform-d) δ 7.61 – 7.51 (m, 2H), 7.50 – 7.41 (m, 3H), 2.93 – 2.83 (m, 2H), 2.02-1.92 (m, 2H), 1.25 (t, *J* = 7.4, 1.8 Hz, 3H).

**$^{13}$C NMR** (101 MHz, Chloroform-d) δ 142.8, 128.7, 128.4, 125.9, 38.4, 24.9, 14.1.

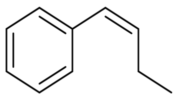

**(*Z*)-but-1-en-1-ylbenzene (4c)[17]**

Colorless liquid, 19.0 mg, 79% yield.

**$^1$H NMR** (400 MHz, Chloroform-d) δ 7.28 – 7.18 (m, 5H), 6.31 (d, *J* = 11.7 Hz, 1H), 5.69 – 5.62 (m, 1H), 2.28 (dd, *J* = 7.5, 1.8 Hz, 2H), 0.99 (t, *J* = 7.5 Hz, 3H).

**$^{13}$C NMR** (101 MHz, Chloroform-d)δ 137.7, 134.7, 128.7, 128.2, 128.0, 126.4, 21.9, 14.4.

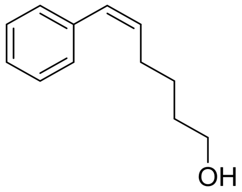


**(*Z*)-trimethyl(3-phenylallyl)-l5-oxidane (4d)[18]**

Yellow liquid, 17.0 mg, 48% yield.

**$^1$H NMR** (400 MHz, Chloroform-d) δ 7.42 – 7.19 (m, 6H), 6.43 (dd, *J* = 11.7, 2.0 Hz, 1H), 5.69 – 5.63 (m, 1H), 3.64 (t, *J* = 6.3 Hz, 2H), 2.65-2.37 (m, 2H), 1.65-1.51 (m, 4H).

**$^{13}$C NMR** (101 MHz, Chloroform-d) δ 137.6, 132.6, 129.1, 128.7, 128.1, 126.5, 62.8, 32.3, 28.2, 26.0.

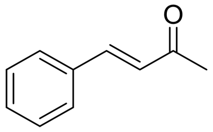

**(*E*)-4-phenylbut-3-en-2-one(4e)[19]**

Yellow liquid, 16.4 mg, 56% yield.
**$^1$H NMR** (400 MHz, Chloroform-*d*) δ 7.58 – 7.52 (m, 2H), 7.50 (s, 1H), 7.43 – 7.36 (m, 3H), 6.72 (d, *J* = 16.3 Hz, 1H), 2.39 (s, 3H).
**$^{13}$C NMR** (101 MHz, Chloroform-*d*) δ 198.44, 143.46, 134.43, 130.53, 128.98, 128.27, 127.18, 27.54.

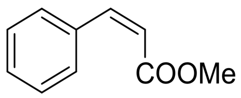


**methyl (*Z*)-3-phenylacrylate (4f)[15]**

Yellow liquid, 23.3 mg, 72% yield.
**$^1$H NMR** (400 MHz, Chloroform-d) δ 7.65 – 7.51 (m, 2H), 7.43 – 7.31 (m, 3H), 6.96 (d, *J* = 12.6 Hz, 1H), 5.96 (d, *J* = 12.6 Hz, 1H), 3.71 (s, 3H).
**$^{13}$C NMR** (101 MHz, Chloroform-d) δ 166.6, 143.4, 129.7, 129.1, 128.1, 128.0, 119.2, 51.4.

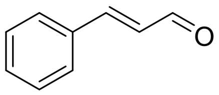


**Cinnamaldehyde (4g)[19]**

Yellow liquid, 15.8 mg, 60% yield.
**$^1$H NMR** (400 MHz, Chloroform-d) δ 9.67 (d, *J* = 7.7 Hz, 1H), 7.57 – 7.52 (m, 2H), 7.48 (s, 1H), 7.42 – 7.38 (m, 3H), 6.71 (dd, *J* = 15.9, 7.8 Hz, 1H).
**$^{13}$C NMR** (101 MHz, Chloroform-d) δ 194.0, 153.1, 133.9, 131.3, 129.1, 129.0, 128.9, 128.5, 128.4, 128.3.

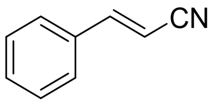


**Cinnamonitrile (4h)[20]**

Brown liquid, 21.7 mg, 84% yield.
**$^1$H NMR** (400 MHz, Chloroform-d) δ 7.43 – 7.39 (m, 5H), 6.94 (d, *J* = 16.7 Hz, 1H), 5.47 (d, *J* = 16.7 Hz, 1H).
**$^{13}$C NMR** (101 MHz, Chloroform-d) δ 150.5, 133.5, 131.2, 129.1, 127.4, 118.3, 96.3.

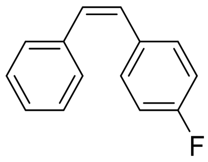


**(*Z*)-1-fluoro-4-styrylbenzene (4i)**[15]

Colorless liquid, 26.9 mg, 68% yield.
**$^1$H NMR** (400 MHz, Chloroform-d) δ 7.21 – 7.10 (m, 7H), 6.83 (t, *J* = 8.7 Hz, 2H), 6.60 – 6.40 (m, 2H).
**$^{13}$C NMR** (101 MHz, Chloroform-d) δ 161.9, 159.5, 135.9, 132.1, 132.0, 129.5, 129.4, 129.1, 129.1, 128.0, 127.7, 127.2, 126.1, 114.2, 113.9.

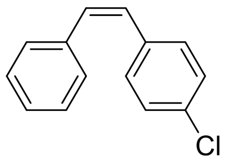


**(*Z*)-1-chloro-4-styrylbenzene (4j)**[15]

Colorless liquid, 26.9 mg, 43% yield.
**$^1$H NMR** (400 MHz, Chloroform-d) δ 7.24 – 7.20 (m, 5H), 7.19 – 7.14 (m, 4H), 6.62 (d, J = 12.2 Hz, 1H), 6.52 (d, J = 12.2 Hz, 1H).
**$^{13}$C NMR** (101 MHz, Chloroform-d) δ 136.8, 135.6, 132.8, 132.7, 131.6, 130.9, 130.2, 128.9, 128.8, 128.7, 128.5, 128.4, 128.4, 127.3.

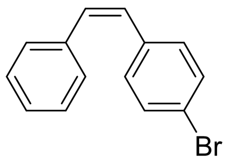


**(*Z*)-1-bromo-4-styrylbenzene (4k)**[15]

Colorless liquid, 7.8 mg, 15% yield.

**$^1$H NMR** (400 MHz, Chloroform-d) δ 7.37 – 7.30 (m, 2H), 7.25 – 7.17 (m, 5H), 7.11 (d, *J* = 8.5 Hz, 2H), 6.63 (d, *J* = 12.2 Hz, 1H), 6.50 (d, *J* = 12.2 Hz, 1H).

**$^{13}$C NMR** (101 MHz, Chloroform-d) δ 136.8, 136.1, 131.4, 131.0, 130.5, 128.9, 128.8, 128.4, 127.4, 120.9.

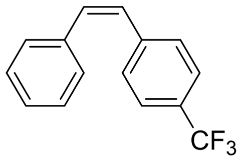


**(*Z*)-1-styryl-4-(trifluoromethyl)benzene (4l)**[15]

Yellow liquid, 36.2 mg, 73% yield.

**$^1$H NMR** (400 MHz, Chloroform-d) δ 7.38 (d, *J* = 8.1 Hz, 2H), 7.25 (d, *J* = 8.1 Hz, 2H), 7.16-7.12 (m, 5H), 6.69 – 6.47 (m, 2H).

**$^{13}$C NMR** (101 MHz, Chloroform-d) δ 140.92, 136.56, 132.34, 129.15, 128.83, 128.75, 128.43, 127.58, 125.22, 125.18, 125.14, 125.11.

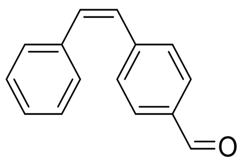


**(*Z*)-4-styrylbenzaldehyde (4m)**[15]

Yellow liquid, 17.0 mg, 41% yield.

**$^1$H NMR** (400 MHz, Chloroform-d) δ 9.95 (s, 1H), 7.77 – 7.70 (m, 2H), 7.40 (d, *J* = 8.0 Hz, 2H), 7.28 – 7.20 (m, 5H), 6.76 (d, *J* = 12.2 Hz, 1H), 6.62 (d, *J* = 12.3 Hz, 1H).

**$^{13}$C NMR** (101 MHz, Chloroform-d) δ 190.8, 142.7, 135.4, 133.9, 131.9, 128.7, 128.5, 127.9, 127.8, 127.4, 126.6, 125.9.

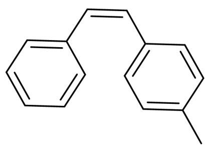

## (*Z*)-1-methyl-4-styrylbenzene (4n)[15]

Colorless liquid, 8.0 mg, 20% yield.

**$^1$H NMR** (400 MHz, Chloroform-d) δ 7.33 – 7.08 (m, 7H), 7.02 (d, *J* = 7.9 Hz, 2H), 6.55 (s, 2H), 2.30 (s, 3H).

**$^{13}$C NMR** (101 MHz, Chloroform-d) δ 137.5, 136.8, 134.3, 130.2, 129.6, 128.9, 128.8, 128.8, 128.2, 126.9, 21.3.

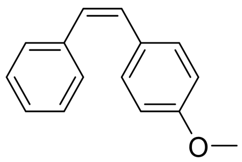


## (*Z*)-1-methoxy-4-styrylbenzene (4o)[15]

Yellow liquid, 15.0 mg, 36% yield.

**$^1$H NMR** (400 MHz, Chloroform-d) δ 7.23 – 7.07 (m, 7H), 6.71 – 6.60 (m, 2H), 6.45 (d, *J* = 1.9 Hz, 2H), 3.71 (s, 3H).

**$^{13}$C NMR** (101 MHz, Chloroform-d) δ 157.6, 136.5, 129.1, 128.7, 128.6, 127.7, 127.7, 127.2, 125.8, 112.5, 54.2.

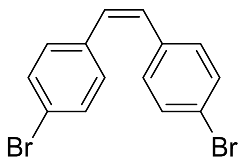


## (*Z*)-1,2-bis(4-bromophenyl)ethene (4p)[21]

Yellow liquid, 17.0 mg, 26% yield.

**$^1$H NMR** (400 MHz, Chloroform-d) δ 7.40 – 7.32 (m, 4H), 7.13 – 7.04 (m, 4H), 6.54 (s, 2H).

**$^{13}$C NMR** (101 MHz, Chloroform-d) δ 134.6, 130.5, 129.4, 128.6, 120.2.

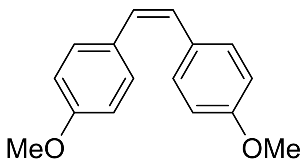

**(*Z*)-1,2-bis(4-methoxyphenyl)ethene (4q) [21]**

Yellow liquid, 15.8 mg, 33% yield.

**$^1$H NMR** (400 MHz, Chloroform-d) δ 7.20 (d, *J* = 8.8 Hz, 4H), 6.77 (d, *J* = 8.8 Hz, 4H), 6.45 (s, 2H), 3.79 (s, 6H).

**$^{13}$C NMR** (101 MHz, Chloroform-d) δ 159.0, 130.1, 127.4, 126.2, 114.1, 55.3.

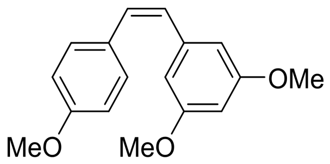


**(Z)-1,3-dimethoxy-5-(4-methoxystyryl)benzene (4r) [15]**

Yellow liquid, 32.4 mg, 60% yield.

**$^1$H NMR** (400 MHz, Chloroform-d) δ 7.21 (d, *J* = 8.6 Hz, 2H), 6.81 – 6.73 (m, 2H), 6.53 (d, *J* = 12.2 Hz, 1H), 6.47 – 6.41 (m, 3H), 6.32 (t, *J* = 2.3 Hz, 1H), 3.78 (s, 3H), 3.67 (s, 6H).

**$^{13}$C NMR** (101 MHz, Chloroform-d) δ 160.6, 158.7, 139.5, 130.3, 130.2, 128.7, 127.8, 113.5, 106.6, 99.7, 55.2, 29.7.

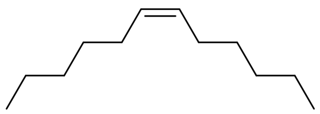

**(*Z*)-dodec-6-ene (4s)[15]**

Colorless liquid, 5.7 mg, 17% yield.

**$^1$H NMR** (400 MHz, Chloroform-d) δ 5.44 – 5.29 (m, 2H), 2.01 (m, *J* = 6.7 Hz, 4H), 1.29 (m *J* = 10.2, 6.8, 3.2 Hz, 12H), 0.89 (d, *J* = 6.5 Hz, 6H).

**$^{13}$C NMR** (101 MHz, Chloroform-d) δ 129.9, 31.5, 29.5, 27.2, 22.6, 14.1.

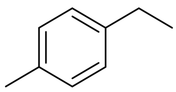

**1- ethyl-4-methylbenzene (6a/6l)[22]**

Yellow liquid, 6a:19.9 mg, 83% yield 6l:18.0 mg, 75% yield.

**$^1$H NMR** (400 MHz, Chloroform-d) δ 7.11 – 7.05 (m, 4H), 2.59 (d, *J* = 7.6 Hz, 2H), 2.30 (t, *J* = 3.7 Hz, 3H), 1.25 – 1.17 (m, 3H).

**$^{13}$C NMR** (101 MHz, Chloroform-d) δ 141.4, 135.1, 129.2, 127.9, 28.6, 21.1, 16.0.

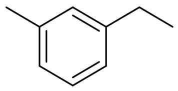

**1-ethyl-3-methylbenzene (6b)[22]**

Yellow liquid, 18.2 mg, 76% yield.

**$^1$H NMR** (400 MHz, Chloroform-d) δ 7.18-7.12 (m, 1H), 7.10 – 6.89 (m, 3H), 2.60 (dd, *J* = 7.6 Hz, 2H), 2.39 – 2.30 (m, 3H), 1.22 (t, *J* = 7.6 Hz, 3H).

**$^{13}$C NMR** (101 MHz, Chloroform-d) δ 144.3, 137.8, 128.7, 128.3, 126.4, 124.9, 28.8, 21.4, 15.7.

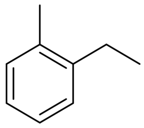

**1-ethyl-2-methylbenzene (6c) [22]**

Yellow liquid, 16.0 mg, 67% yield.

**$^1$H NMR** (400 MHz, Chloroform-d) δ 7.51-7.43 (m, 4H), 3.08 – 2.90 (m, 2H), 2.65 (d, *J* = 4.6, 2.2 Hz, 3H), 1.57 (t, *J* = 7.3, 4.9, 2.1 Hz, 3H).

**$^{13}$C NMR** (101 MHz, Chloroform-d) δ 142.5, 135.9, 130.3, 128.2, 126.3, 126.1, 26.5, 19.4, 14.7.

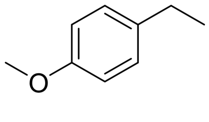


**1-ethyl-4-methoxybenzene (6d)[22]**

Yellow liquid, 7.9 mg,29% yield.

**$^1$H NMR** (400 MHz, Chloroform-d) δ 7.18 – 7.10 (m, 2H), 6.90 – 6.82 (m, 2H), 3.81 (d, $J$ = 0.9 Hz, 3H), 2.61 (q, $J$ = 7.6 Hz, 2H), 1.23 (t, $J$ = 7.6, 1.2 Hz, 3H).
**$^{13}$C NMR** (101 MHz, Chloroform-d) δ 157.6, 136.4, 128.7, 113.7, 55.3, 27.9, 15.9.

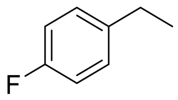


**1-ethyl-4-fluorobenzene (6e)[23]**

Colorless liquid, 23.0 mg,93% yield.
**$^1$H NMR** (400 MHz, Chloroform-d) δ 7.20 – 7.12 (m, 2H), 7.01 – 6.93 (m, 2H), 2.63 (q, $J$ = 7.6 Hz, 2H), 1.23 (t, $J$ = 7.6 Hz, 3H).
**$^{13}$C NMR** (101 MHz, Chloroform-d) δ 162.3, 159.9, 139.8, 139.7, 129.1, 129.1, 115.1, 114.8, 28.1, 15.8.

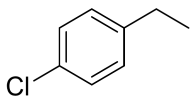


**1-chloro-4-ethylbenzene(6f/6m)**[22]

Colorless liquid, 6f: 22.6 mg, 81% yield. 6m: 21.0 mg, 75% yield.
**$^1$H NMR** (400 MHz, Chloroform-d) δ 7.35 – 7.27 (m, 2H), 7.22 – 7.14 (m, 2H), 2.67 (q, $J$ = 7.6 Hz, 2H), 1.28 (t, $J$ = 7.6, 1.1 Hz, 3H).
**$^{13}$C NMR** (101 MHz, Chloroform-d) δ 142.6, 131.2, 129.2, 128.3, 28.3, 15.5.

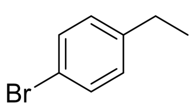


**1-bromo-4-ethylbenzene (6g)[24]**

Yellow liquid, 22.6 mg, 81% yield.

**$^{1}$H NMR** (400 MHz, Chloroform-d) δ 7.41 – 7.31 (m, 2H), 7.08 – 6.99 (m, 2H), 2.55 (q, *J* = 7.6 Hz, 2H), 1.18 (t, *J* = 7.6 Hz, 3H).
**$^{13}$C NMR** (101 MHz, Chloroform-d) δ 143.2, 131.3, 129.6, 119.3, 28.3, 15.5.

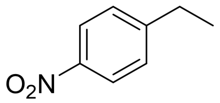


**1-ethyl-4-nitrobenzene (6h/6o)[25]**

Yellow liquid, **6h**: 29.0 mg, 95% yield. **6o**: 22.7 mg,75%.
**$^{1}$H NMR** (400 MHz, Chloroform-d) δ 8.17 – 8.00 (m, 2H), 7.34-7.31 (m, 2H), 2.77-2.71 (m, 2H), 1.27 (t, *J* = 7.7, 1.1 Hz, 3H).
**$^{13}$C NMR** (101 MHz, Chloroform-d) δ 152.0, 146.0, 128.5, 123.4, 28.7, 14.8.

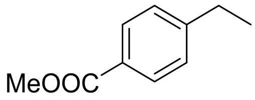


**methyl 4-ethylbenzoate (6i/6r)[25]**

Yellow liquid, 6i: 27.2 mg, 83% yield. 6r: 23.6 mg, 72% yield.
**$^{1}$H NMR** (400 MHz, Chloroform-d) δ 7.99 – 7.92 (m, 2H), 7.24 (d, *J* = 8.0 Hz, 2H), 3.89 (s, 3H), 2.68 (q, *J* = 7.6 Hz, 2H), 1.24 (t, *J* = 7.6 Hz, 3H).
**$^{13}$C NMR** (101 MHz, Chloroform-d) δ 167.1, 149.7, 129.7, 127.8, 127.6, 51.9, 28.9, 15.2.

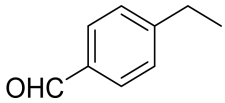


**4-ethylbenzaldehyde (6j/6q)[26]**

Yellow liquid, 6j: 22.5 mg, 84% yield. 6q: 24.9 mg, 93% yield.
**$^{1}$H NMR** (400 MHz, Chloroform-d) δ 9.95 (d, *J* = 1.7 Hz, 1H), 7.78 (dd, *J* = 8.2, 1.8 Hz, 2H), 7.40 – 7.26 (m, 2H), 2.71 (d, *J* = 7.6 Hz, 2H), 1.25 (t, *J* = 7.7, 1.8 Hz, 3H).
**$^{13}$C NMR** (101 MHz, Chloroform-d) δ 191.9, 151.6, 134.4, 129.9, 128.5, 29.1, 15.1.

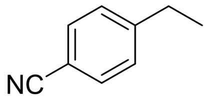


**4-ethylbenzonitrile (6n)[27]**

Colorless liquid, 23.6mg, 79% yield.

**$^{1}$H NMR** (400 MHz, Chloroform-d) δ 7.54 (dd, *J* = 8.2, 1.9 Hz, 2H), 7.29 (dd, *J* = 8.3, 1.9 Hz, 2H), 2.70 (q, *J* = 7.6, 1.8 Hz, 2H), 1.24 (t, *J* = 7.6, 1.9 Hz, 3H).

**$^{13}$C NMR** (101 MHz, Chloroform-d) δ 149.8, 132.1, 132.1, 128.7, 119.2, 109.4, 29.0, 15.0.

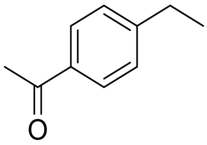


**1-(4-ethylphenyl)ethan-1-one (6p)[28]**

Yellow liquid, 23.3mg, 79% yield.

**$^{1}$H NMR** (400 MHz, Chloroform-d) δ 7.91 – 7.85 (m, 2H), 7.28 (d, *J* = 8.2 Hz, 2H), 2.70 (q, *J* = 7.7 Hz, 2H), 2.58 (s, 3H), 1.25 (t, *J* = 7.6 Hz, 3H).

**$^{13}$C NMR** (101 MHz, Chloroform-d) δ 197.1, 149.7, 134.8, 128.4, 127.8, 28.7, 26.2, 15.1.

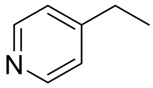


**4-ethylpyridine (6k) [2]**

Colorless liquid, 7.0 mg, 33% yield.

**$^{1}$H NMR** (400 MHz, Chloroform-d) δ 8.27 – 7.94 (m, 2H), 6.85 – 6.58 (m, 2H), 2.22 (q, *J* = 7.6 Hz, 2H), 0.83 (t, *J* = 7.7 Hz, 3H).

**$^{13}$C NMR** (101 MHz, Chloroform-d) δ 152.4, 149.2, 122.9, 27.7, 13.8.

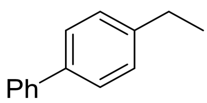


**4-ethyl-1,1'-biphenyl (6s)[29]**

Yellow liquid, 3.6mg, 10% yield.

**$^1$H NMR** (400 MHz, Chloroform-d) δ 7.64 – 7.57 (m, 2H), 7.57 – 7.51 (m, 2H), 7.49 – 7.39 (m, 2H), 7.38 – 7.27 (m, 3H), 2.71 (q, *J* = 7.6 Hz, 2H), 1.30 (t, *J* = 7.6 Hz, 3H).

**$^{13}$C NMR** (101 MHz, Chloroform-d) δ 143.4, 141.2, 138.6, 128.7, 128.3, 127.1, 127.0, 126.9, 28.5, 15.6.

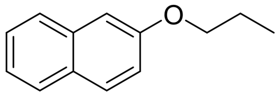

**2-propoxynaphthalene (6t)[30]**

**$^1$H NMR** (400 MHz, Chloroform-d) δ 7.81 – 7.68 (m, 4H), 7.43-7.30 (m, 1H), 7.19 – 7.11 (m, 2H), 4.05 (t, *J* = 6.6 Hz, 2H), 1.88 (q, *J* = 7.0 Hz, 2H), 1.09 (t, *J* = 7.4 Hz, 3H).

**$^{13}$C NMR** (101 MHz, Chloroform-d) δ 157.1, 129.3, 127.6, 126.7, 126.3, 123.4, 119.0, 106.6, 69.5, 22.6, 10.6.

## 9. ICp-Ms Test Report

Sample: *n*$Bu_4NCl$ (from TCI)

| Sample number | Sample name | Element | Value reporting(mg/kg) |
|---|---|---|---|
| KYHX20223328 | $Bu_4NCl$ | Rh | ＜0.5 |
| KYHX20223328 | $Bu_4NCl$ | Ir | ＜0.5 |
| KYHX20223328 | $Bu_4NCl$ | Pd | ＜0.5 |
| KYHX20223328 | $Bu_4NCl$ | Ru | ＜0.5 |
| KYHX20223328 | $Bu_4NCl$ | Fe | ＜0.5 |

Sample: **1a**

| Sample number | Sample name | Element | Value reporting(mg/kg) |
|---|---|---|---|
| KYHX20235280 | **1a** | Rh | ＜0.5 |
| KYHX20235280 | **1a** | Ir | ＜0.5 |
| KYHX20235280 | **1a** | Pd | ＜0.5 |
| KYHX20235280 | **1a** | Ru | ＜0.5 |
| KYHX20235280 | **1a** | Fe | ＜0.5 |

# 10. NMR spectra for the products

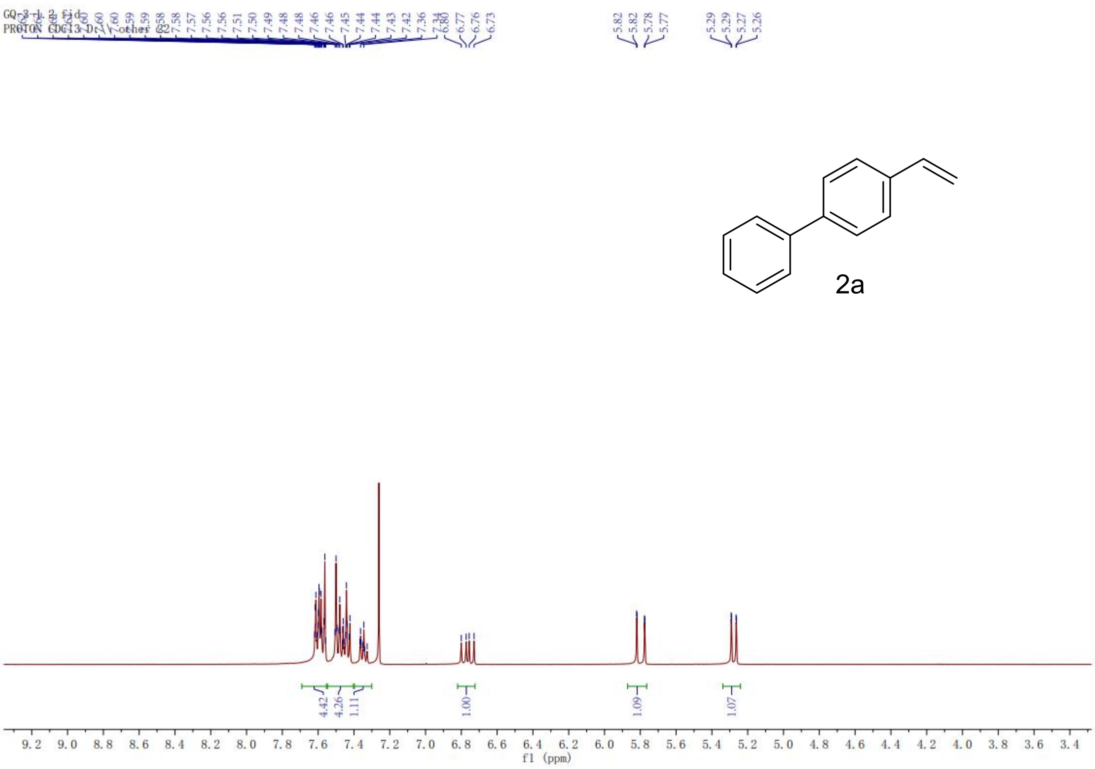


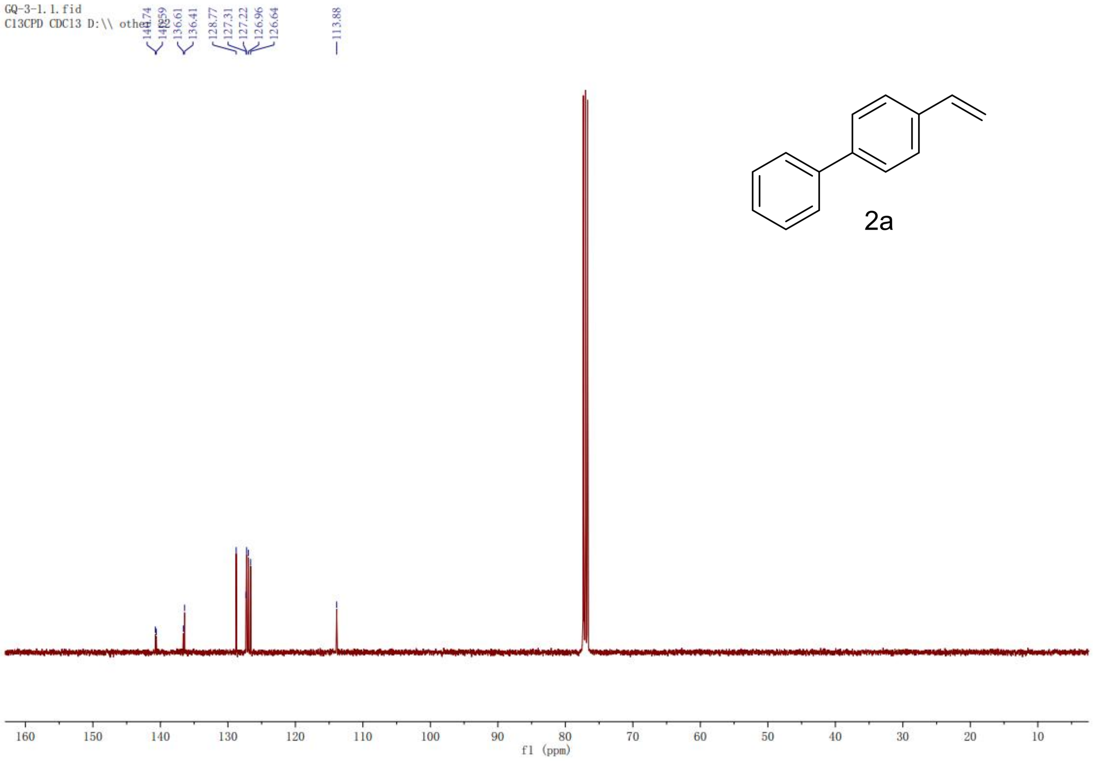

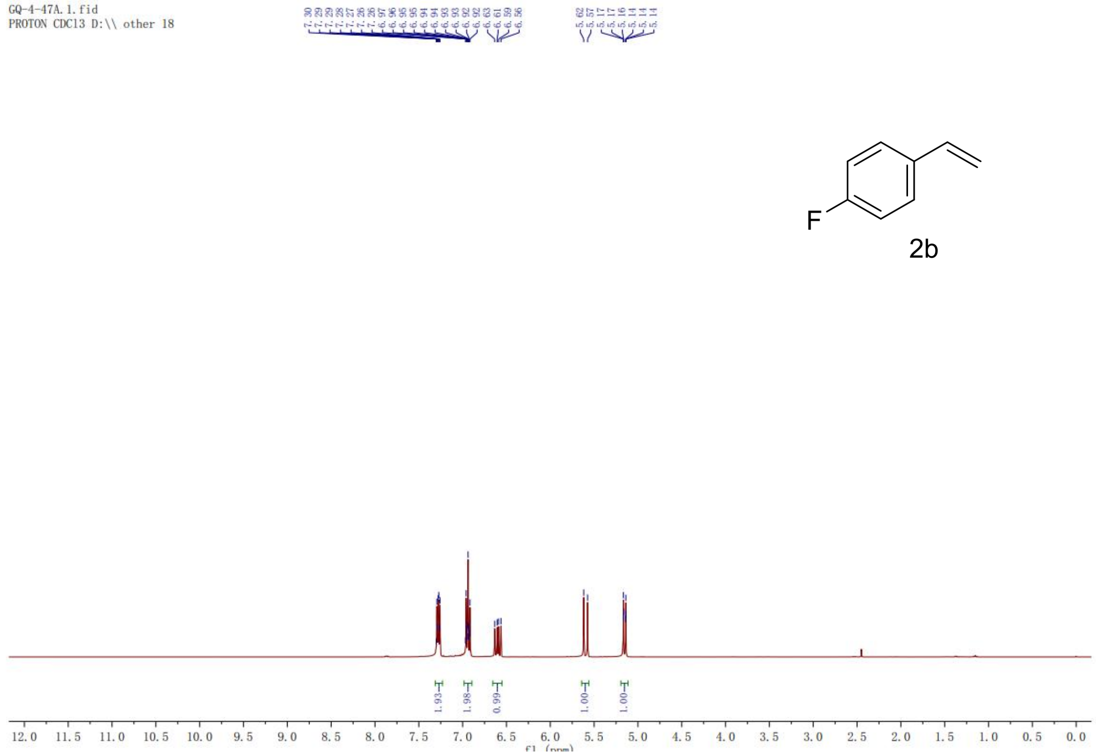
GQ-4-47A.1.fid
PROTON CDCl3 D:\\ other 18
7.30 7.29 7.29 7.28 7.27 7.26 7.26 6.97 6.96 6.95 6.95 6.94 6.94 6.93 6.93 6.92 6.92 6.63 6.61 6.59 6.56
5.62 5.57 5.17 5.17 5.16 5.14 5.14 5.14
F
2b
1.93
1.98
0.99
1.00
1.00
f1 (ppm)

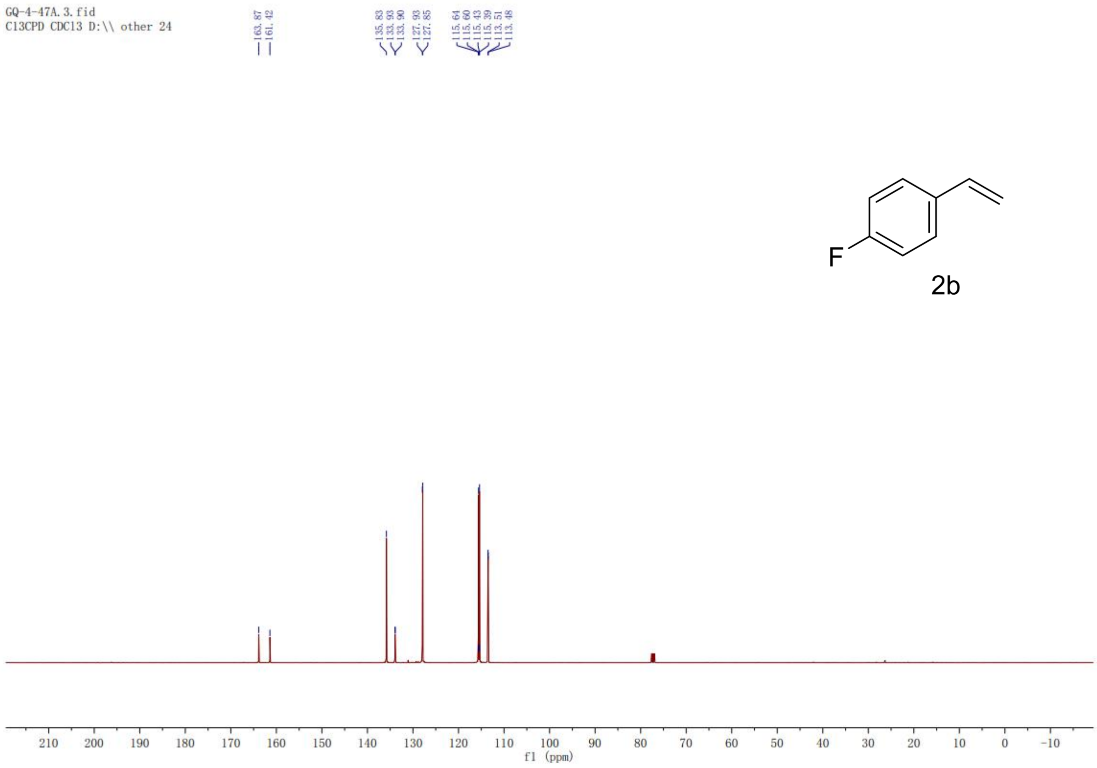
GQ-4-47A.3.fid
C13CPD CDCl3 D:\\ other 24
163.87 161.42
135.83 133.93 133.90 127.93 127.85
115.64 115.60 115.43 115.39 113.51 113.48
F
2b
f1 (ppm)

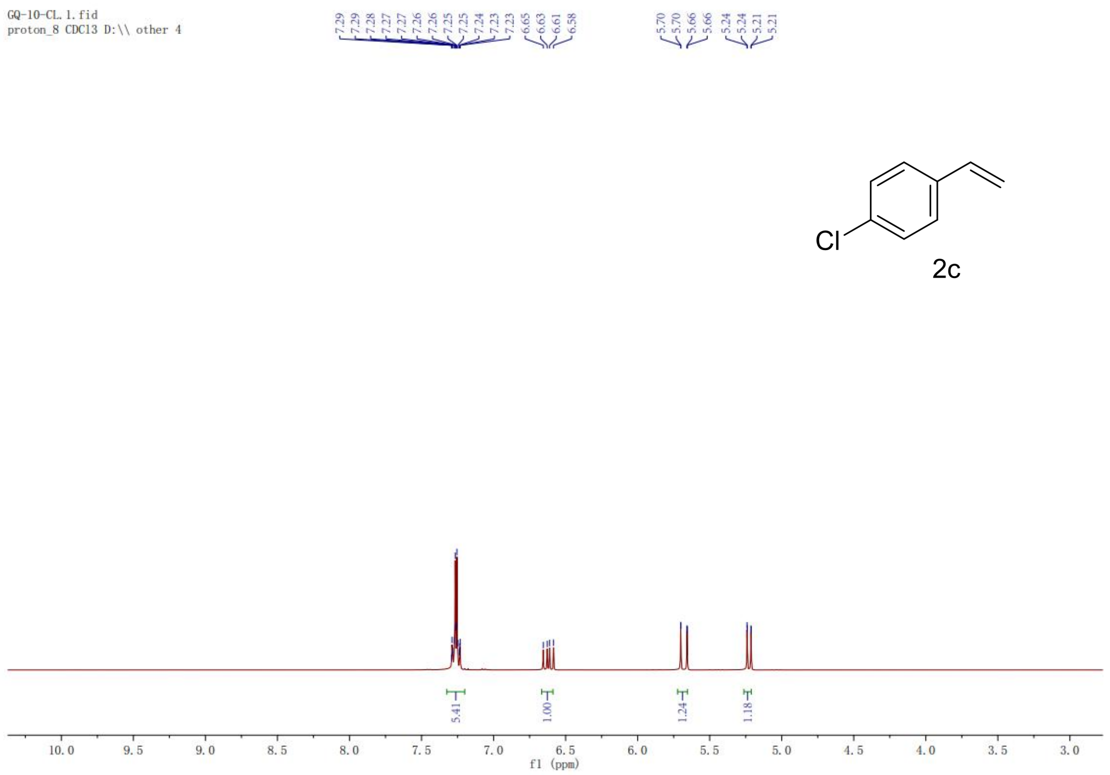
GQ-10-CL.1.fid
proton_8 CDCl3 D:\\ other 4
7.29
7.29
7.28
7.27
7.27
7.26
7.26
7.25
7.25
7.24
7.23
7.23
6.65
6.63
6.61
6.58
5.70
5.70
5.66
5.66
5.24
5.24
5.21
5.21
Cl
2c
5.41
1.00
1.24
1.18
10.0
9.5
9.0
8.5
8.0
7.5
7.0
6.5
6.0
5.5
5.0
4.5
4.0
3.5
3.0
f1 (ppm)

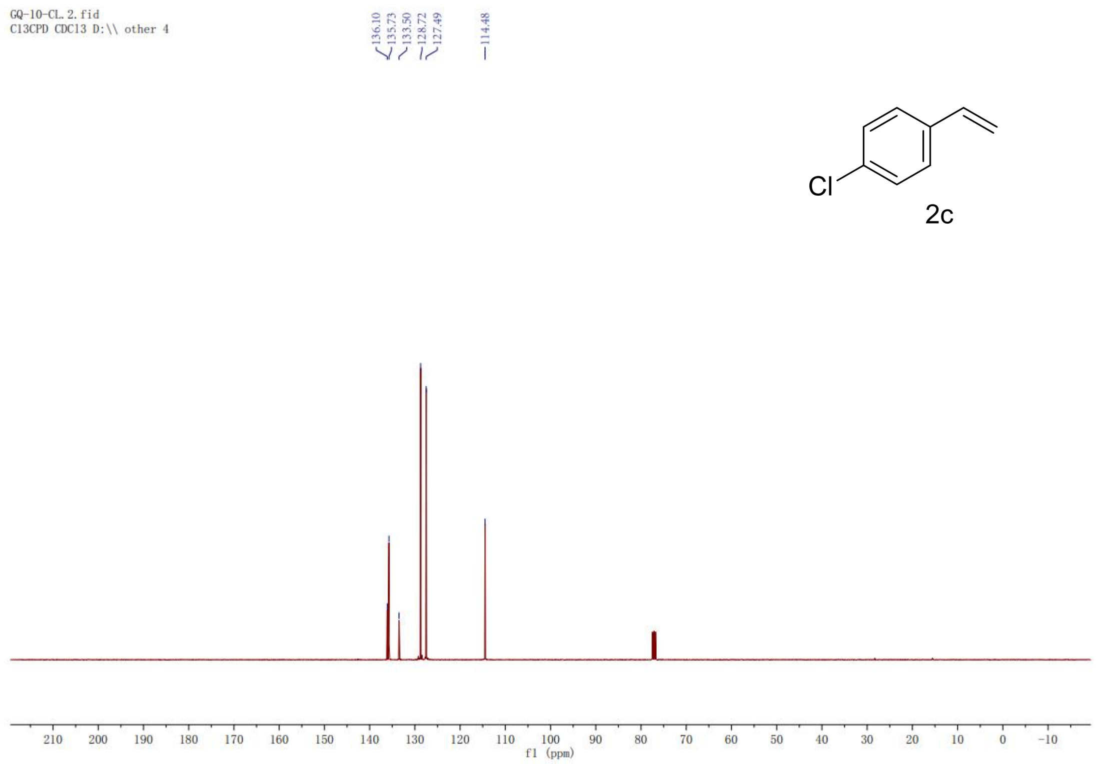
GQ-10-CL.2.fid
C13CPD CDCl3 D:\\ other 4
136.10
135.73
133.50
128.72
127.49
114.48
Cl
2c
210
200
190
180
170
160
150
140
130
120
110
100
90
80
70
60
50
40
30
20
10
0
-10
f1 (ppm)

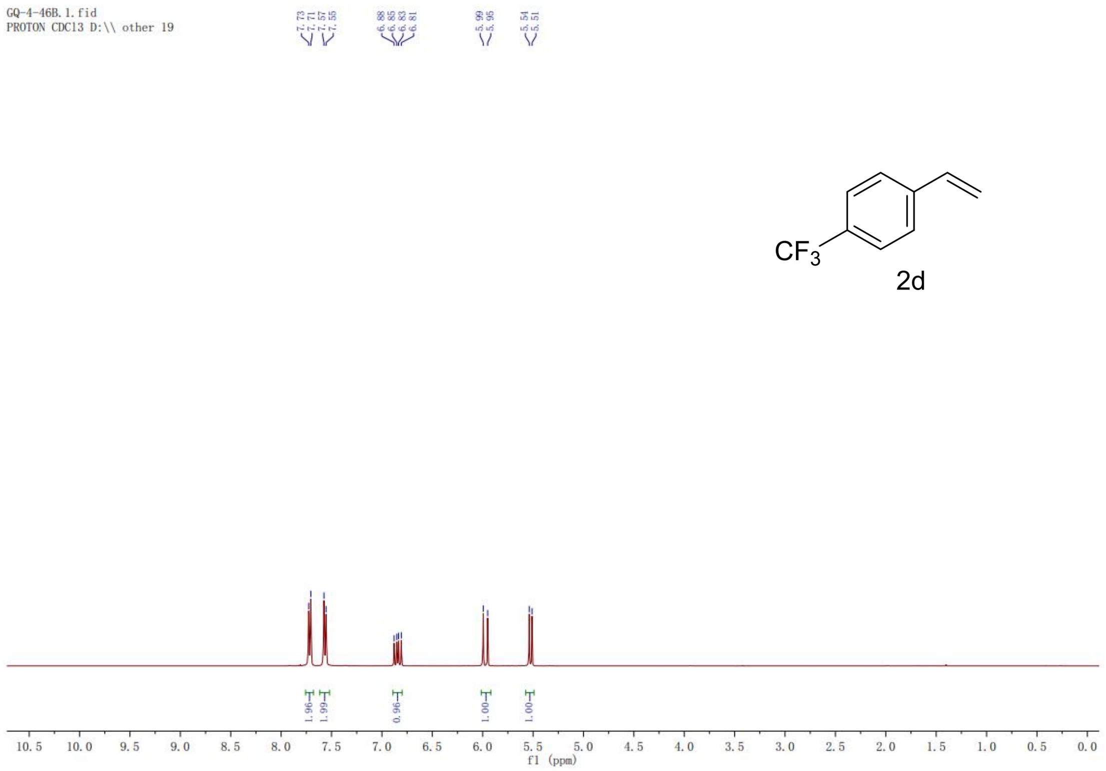
GQ-4-46B.1.fid
PROTON CDCl3 D:\\ other 19
7.73
7.71
7.57
7.55
6.88
6.85
6.83
6.81
5.99
5.95
5.54
5.51
CF3
2d
1.96
1.99
0.96
1.00
1.00
10.5 10.0 9.5 9.0 8.5 8.0 7.5 7.0 6.5 6.0 5.5 5.0 4.5 4.0 3.5 3.0 2.5 2.0 1.5 1.0 0.5 0.0
f1 (ppm)

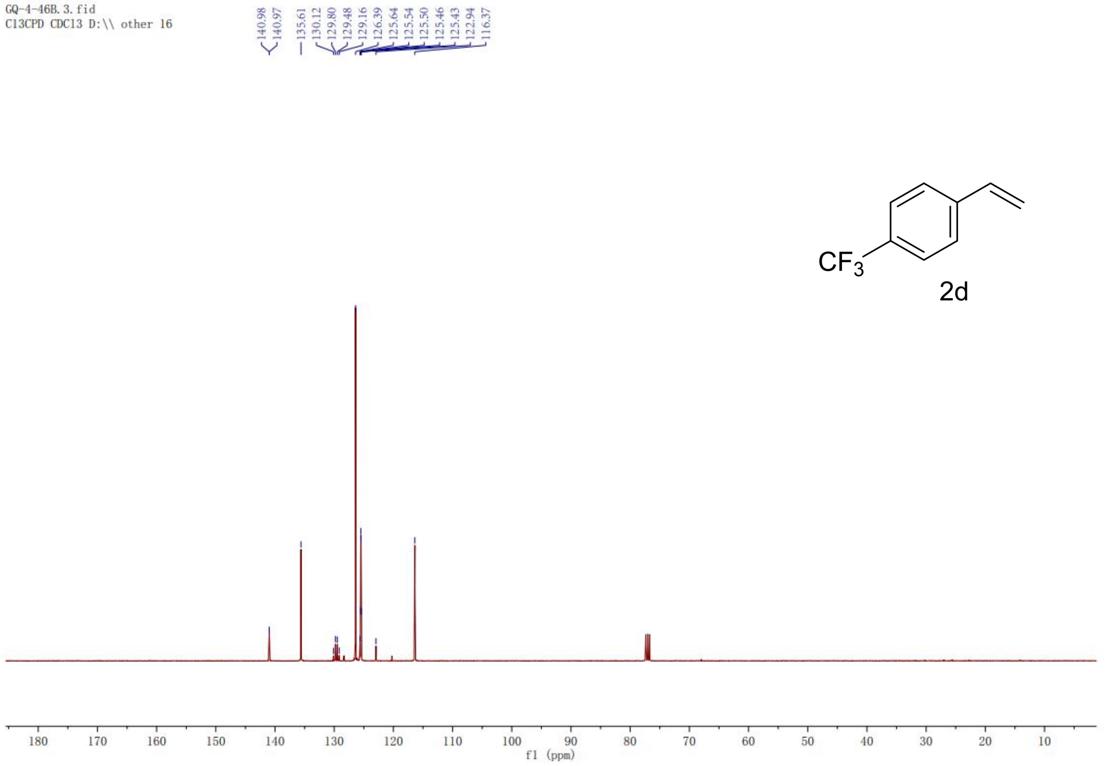
GQ-4-46B.3.fid
C13CPD CDCl3 D:\\ other 16
140.98
140.97
135.61
130.12
129.80
129.48
129.16
126.39
125.64
125.54
125.50
125.46
125.43
122.94
116.37
CF3
2d
180 170 160 150 140 130 120 110 100 90 80 70 60 50 40 30 20 10
f1 (ppm)

GQ-6-59A.3.fid
PROTON CDCl3 D:\\ other 9

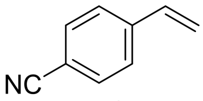

2e

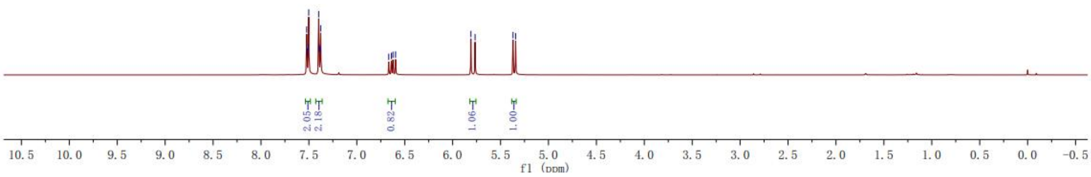


GQ-6-59A.5.fid
C13CPD CDCl3 D:\\ other 24

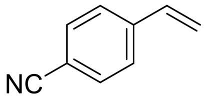

2e

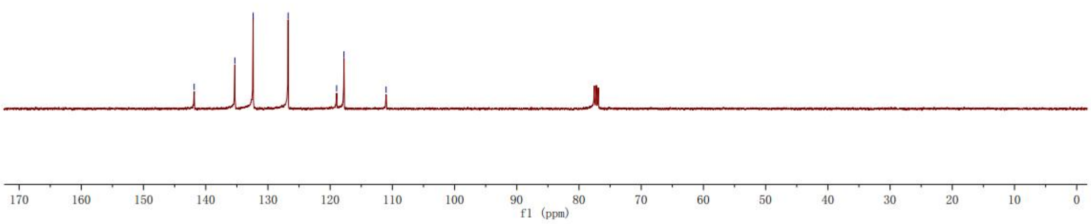

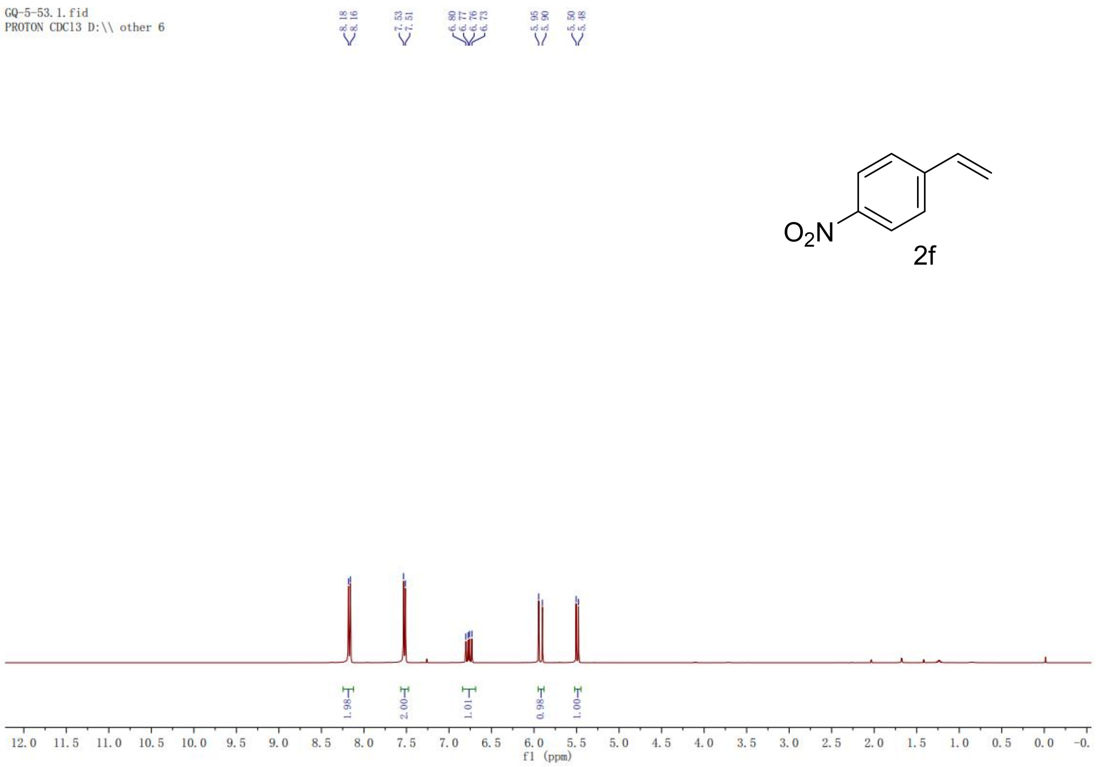
GQ-5-53.1.fid
PROTON CDCl3 D:\\ other 6
8.18
8.16
7.53
7.51
6.80
6.77
6.76
6.73
5.95
5.90
5.50
5.48
O2N
2f
1.98
2.00
1.01
0.98
1.00
f1 (ppm)

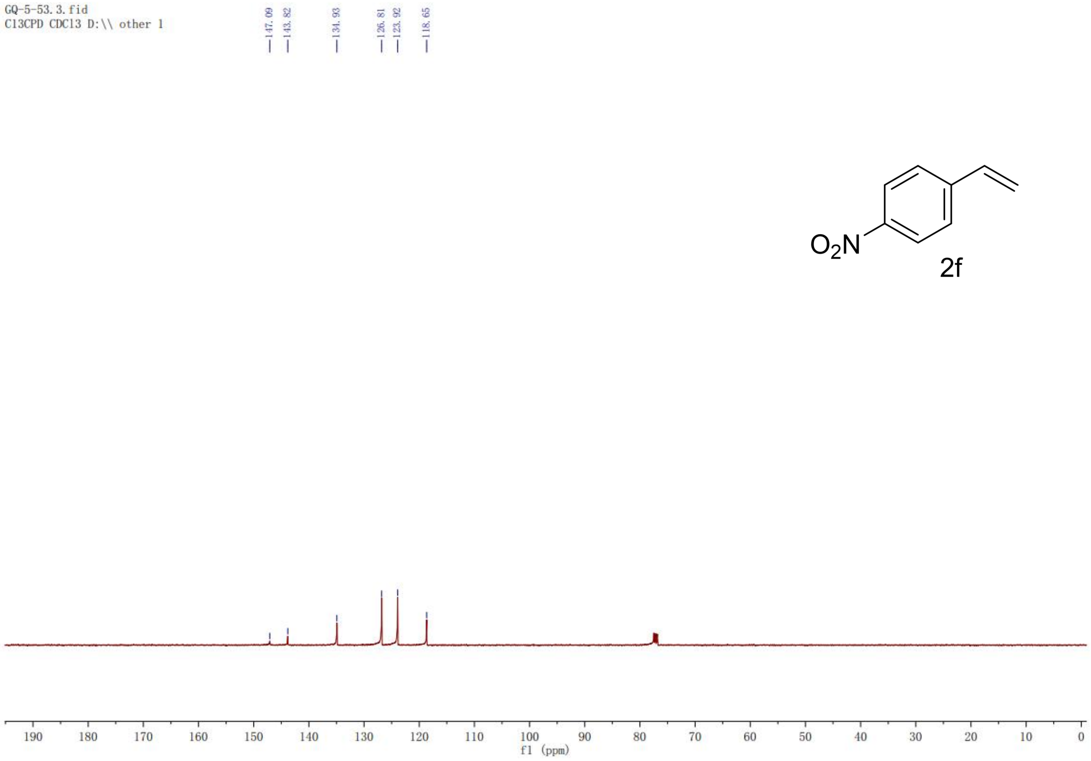
GQ-5-53.3.fid
C13CPD CDCl3 D:\\ other 1
147.09
143.82
134.93
126.81
123.92
118.65
O2N
2f
f1 (ppm)

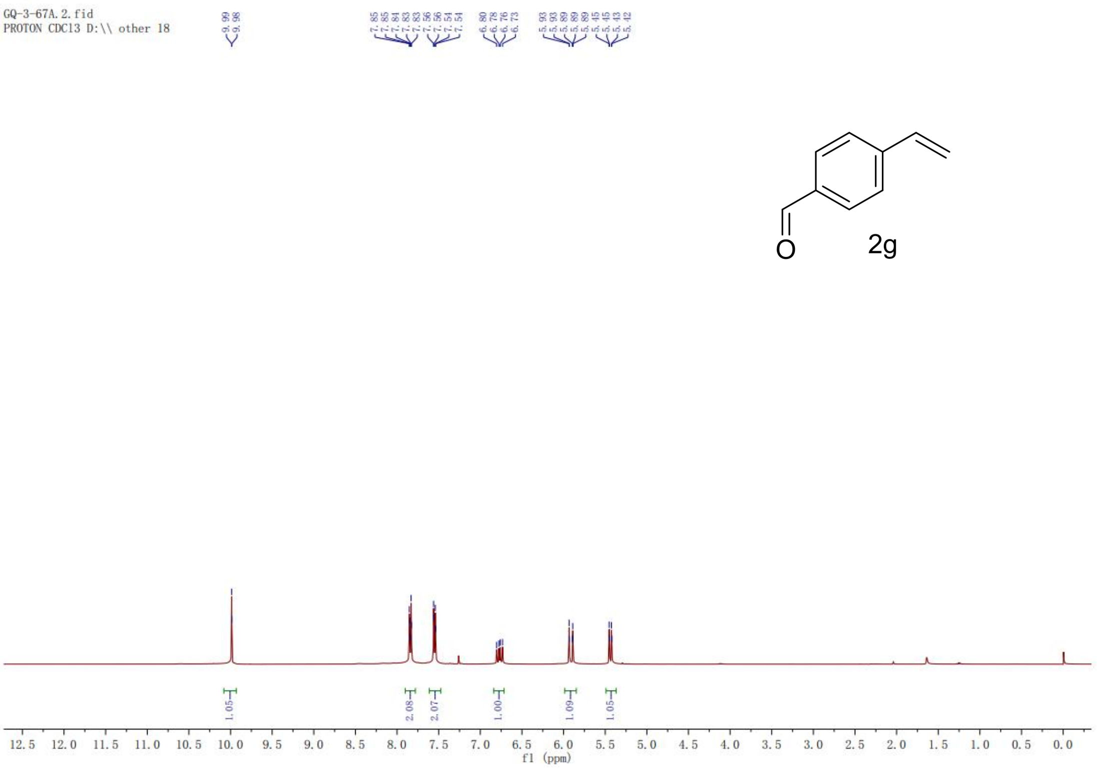
GQ-3-67A.2.fid
PROTON CDCl3 D:\\ other 18
9.99
9.98
7.85
7.85
7.84
7.83
7.83
7.56
7.56
7.54
7.54
6.80
6.78
6.76
6.73
5.93
5.93
5.89
5.89
5.89
5.45
5.45
5.43
5.42
O
2g
1.05
2.08
2.07
1.00
1.09
1.05
12.5
12.0
11.5
11.0
10.5
10.0
9.5
9.0
8.5
8.0
7.5
7.0
6.5
6.0
5.5
5.0
4.5
4.0
3.5
3.0
2.5
2.0
1.5
1.0
0.5
0.0
f1 (ppm)

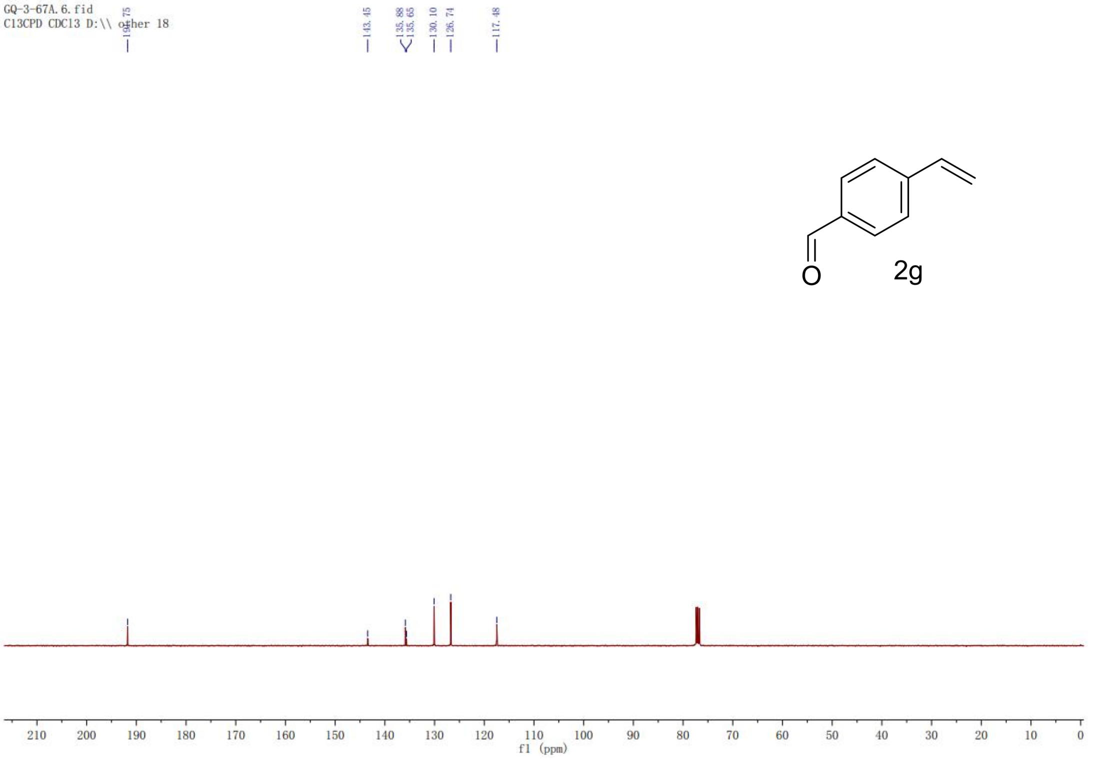
GQ-3-67A.6.fid
C13CPD CDCl3 D:\\ other 18
143.45
135.88
135.65
130.10
126.74
117.48
O
2g
210
200
190
180
170
160
150
140
130
120
110
100
90
80
70
60
50
40
30
20
10
0
f1 (ppm)

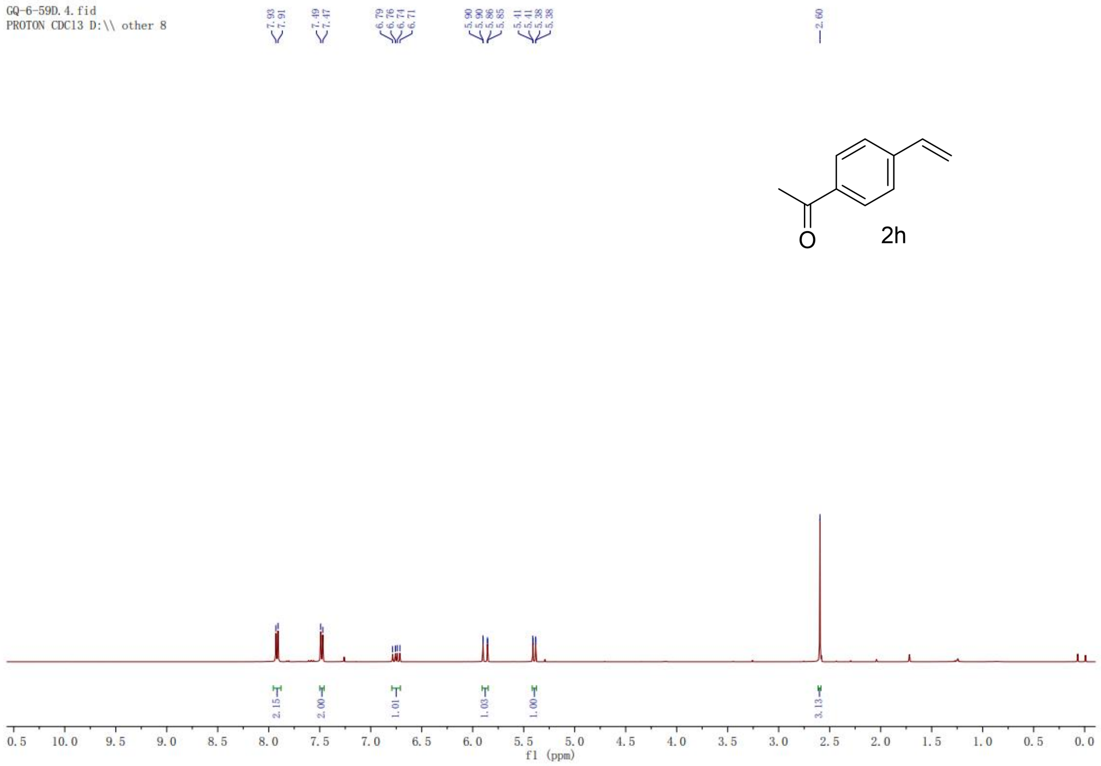
GQ-6-59D.4.fid
PROTON CDCl3 D:\\ other 8
7.93
7.91
7.49
7.47
6.79
6.76
6.74
6.71
5.90
5.90
5.86
5.85
5.41
5.41
5.38
5.38
2.60
2h
2.15
2.00
1.01
1.03
1.00
3.13
f1 (ppm)

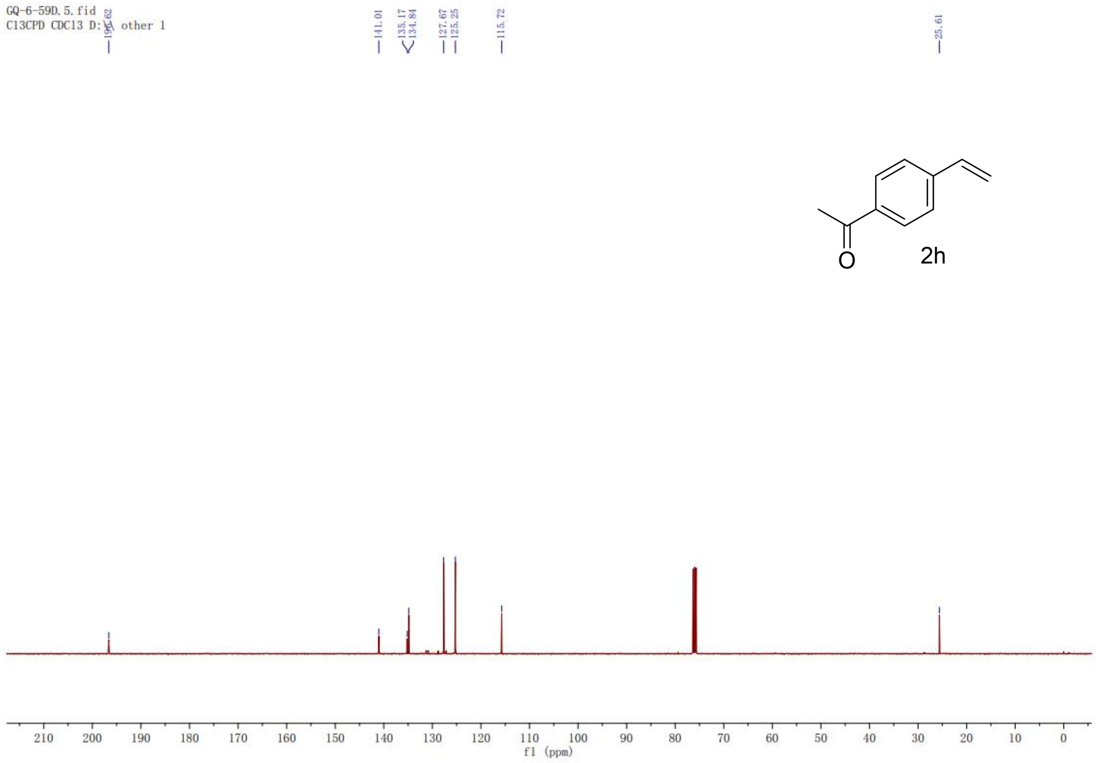
GQ-6-59D.5.fid
C13CPD CDCl3 D:\\ other 1
141.01
135.17
134.84
127.67
125.25
115.72
25.61
2h
f1 (ppm)

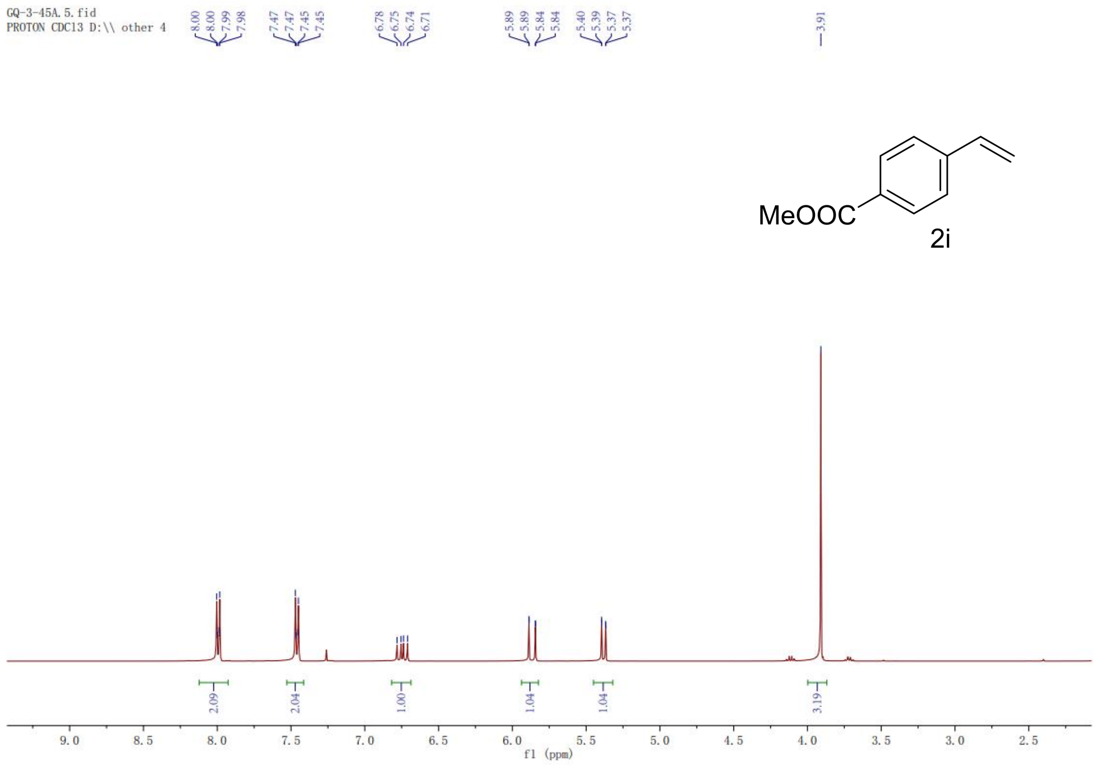
GQ-3-45A.5.fid
PROTON CDCl3 D:\\ other 4
8.00
8.00
7.99
7.98
7.47
7.47
7.45
7.45
6.78
6.75
6.74
6.71
5.89
5.89
5.84
5.84
5.40
5.39
5.37
5.37
3.91
MeOOC
2i
2.09
2.04
1.00
1.04
1.04
3.19
9.0
8.5
8.0
7.5
7.0
6.5
6.0
5.5
5.0
4.5
4.0
3.5
3.0
2.5
f1 (ppm)

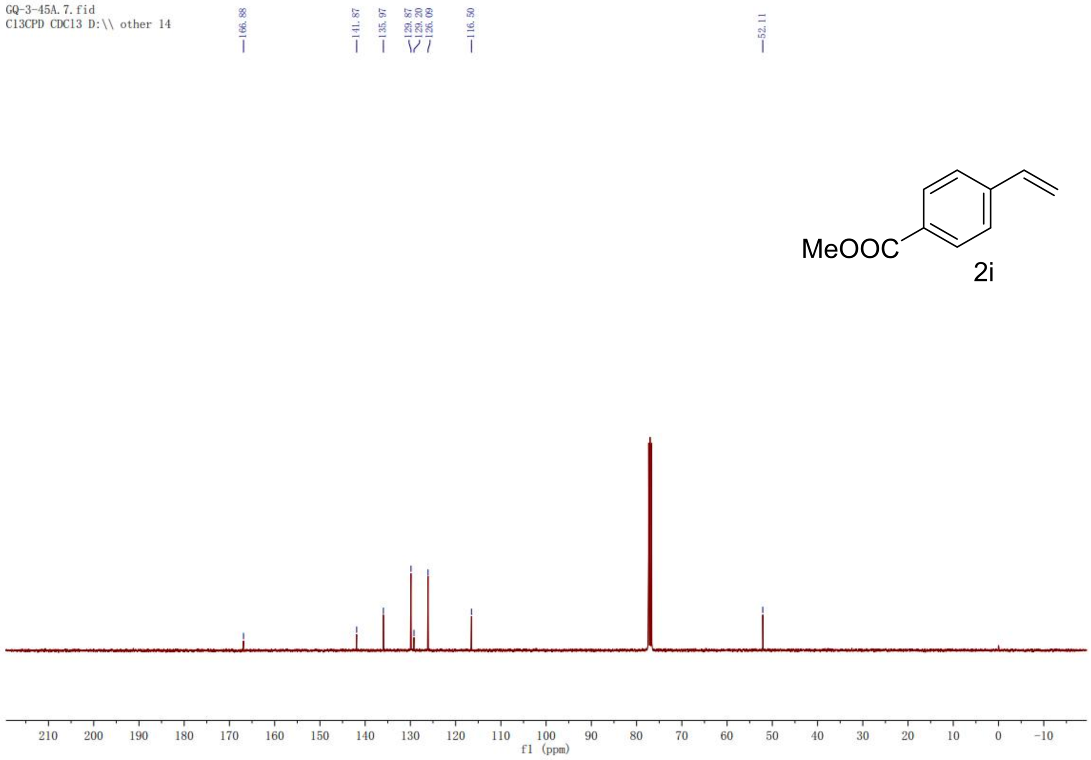
GQ-3-45A.7.fid
C13CPD CDCl3 D:\\ other 14
166.88
141.87
135.97
129.87
129.20
126.09
116.50
52.11
MeOOC
2i
210
200
190
180
170
160
150
140
130
120
110
100
90
80
70
60
50
40
30
20
10
0
-10
f1 (ppm)

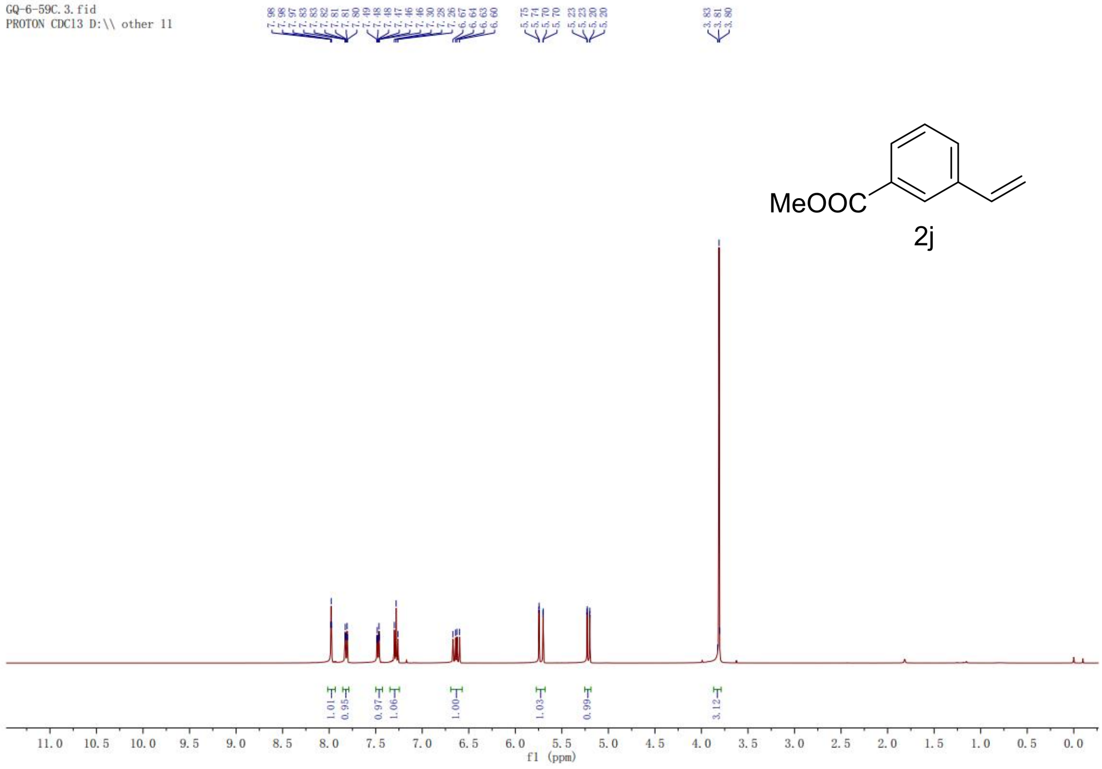
GQ-6-59C.3.fid
PROTON CDCl3 D:\\ other 11
7.98 7.98 7.97 7.83 7.83 7.82 7.81 7.81 7.80 7.49 7.48 7.48 7.47 7.46 7.46 7.30 7.28 7.26 6.67 6.64 6.63 6.60
5.75 5.74 5.70 5.70 5.23 5.23 5.20 5.20
3.83 3.81 3.80
MeOOC
2j
1.01 0.95 0.97 1.06 1.00 1.03 0.99 3.12
f1 (ppm)

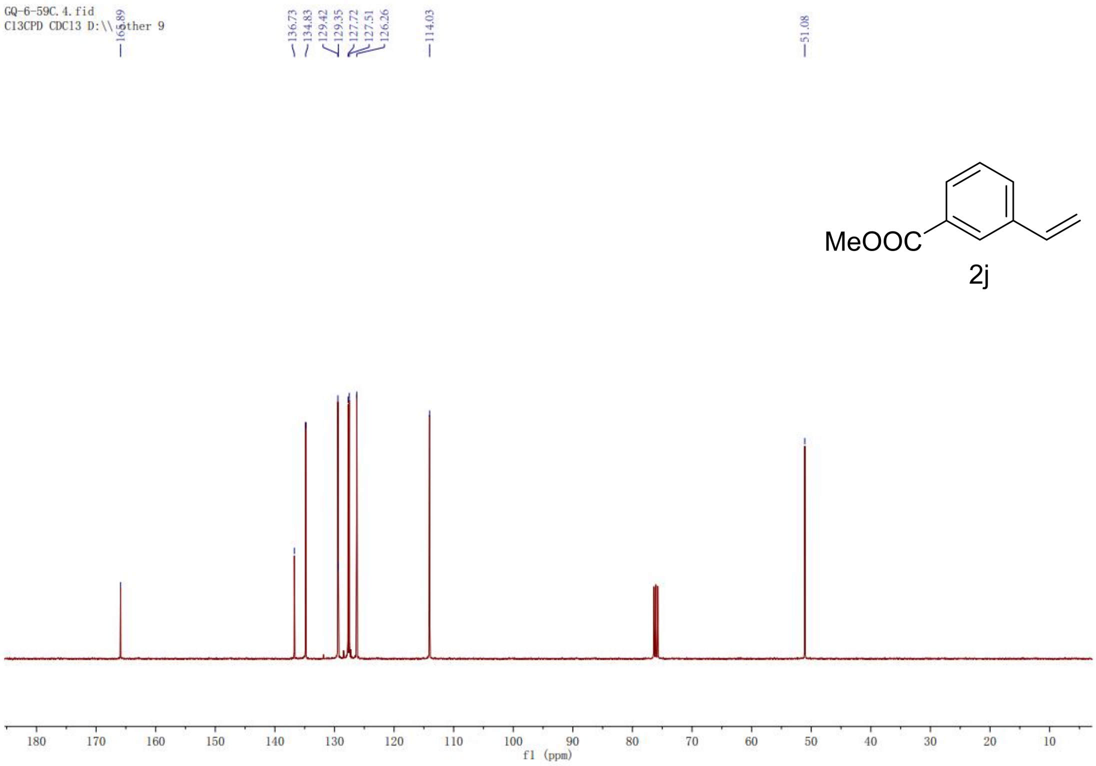
GQ-6-59C.4.fid
C13CPD CDCl3 D:\\ other 9
165.89
136.73 134.83 129.42 129.35 127.72 127.51 126.26
114.03
51.08
MeOOC
2j
f1 (ppm)

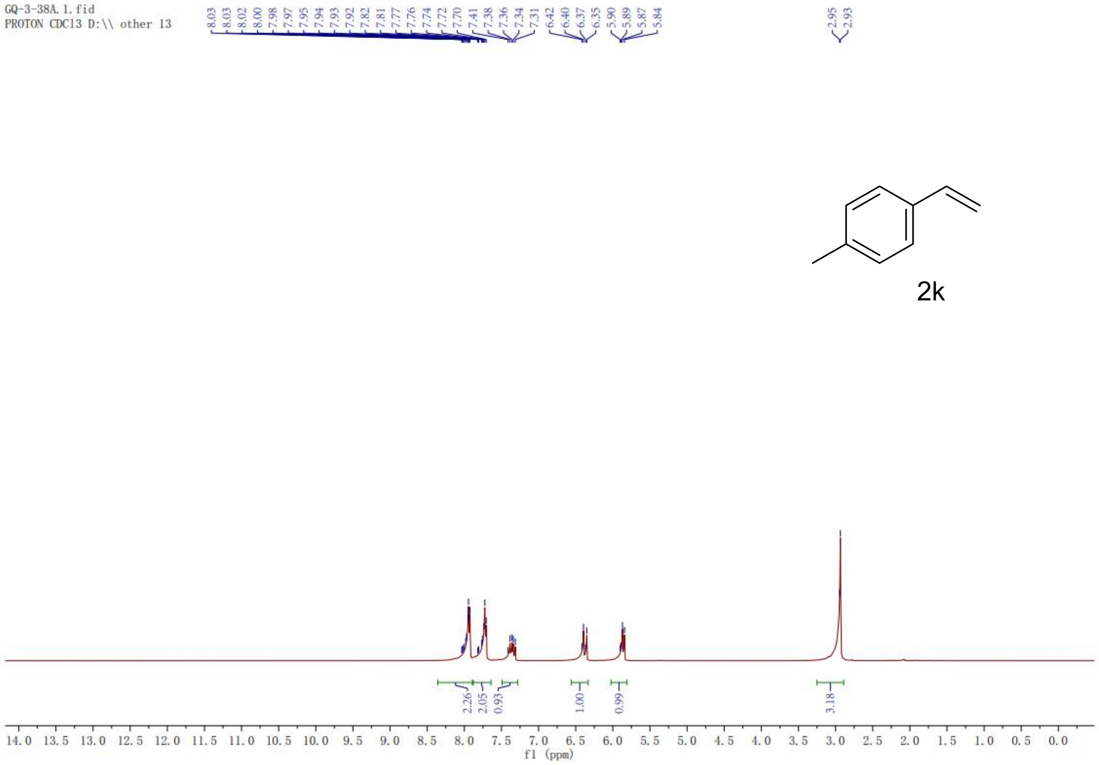
GQ-3-38A.1.fid
PROTON CDCl3 D:\\ other 13
8.03
8.03
8.02
8.00
7.98
7.97
7.95
7.94
7.93
7.92
7.82
7.81
7.77
7.76
7.74
7.72
7.70
7.41
7.38
7.36
7.34
7.31
6.42
6.40
6.37
6.35
5.90
5.89
5.87
5.84
2.95
2.93
2.26
2.05
0.93
1.00
0.99
3.18
f1 (ppm)

2k

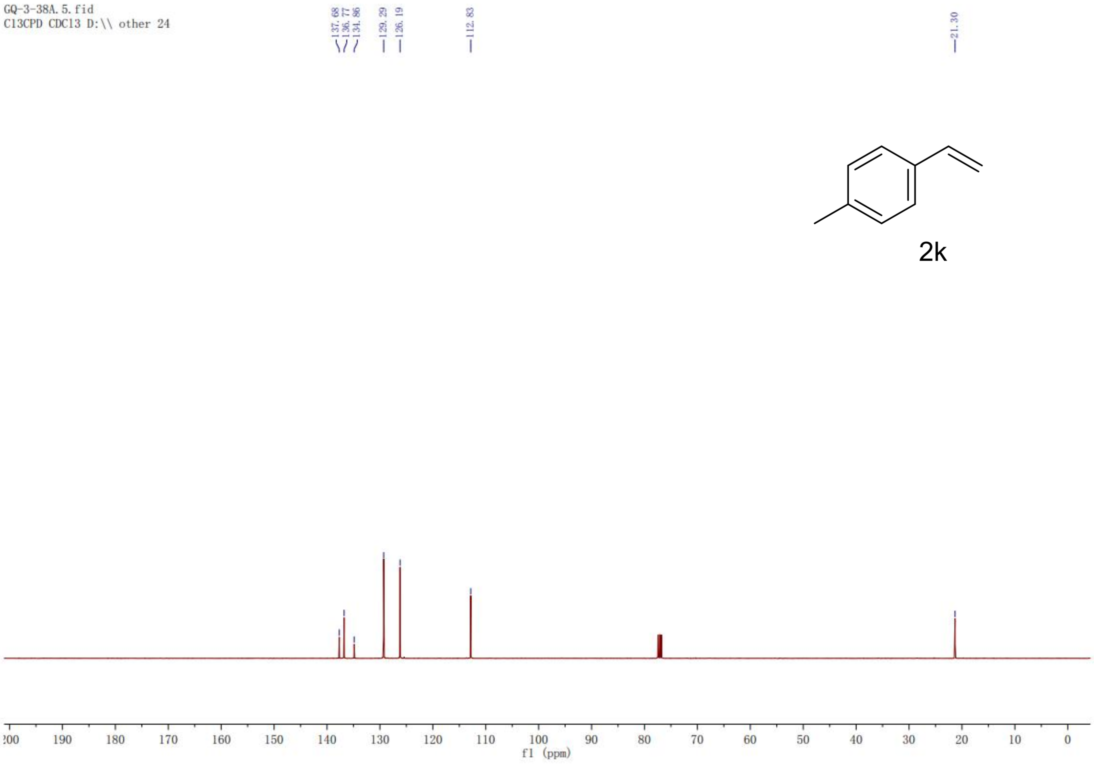
GQ-3-38A.5.fid
C13CPD CDCl3 D:\\ other 24
137.68
136.77
134.86
129.29
126.19
112.83
21.30
f1 (ppm)

2k

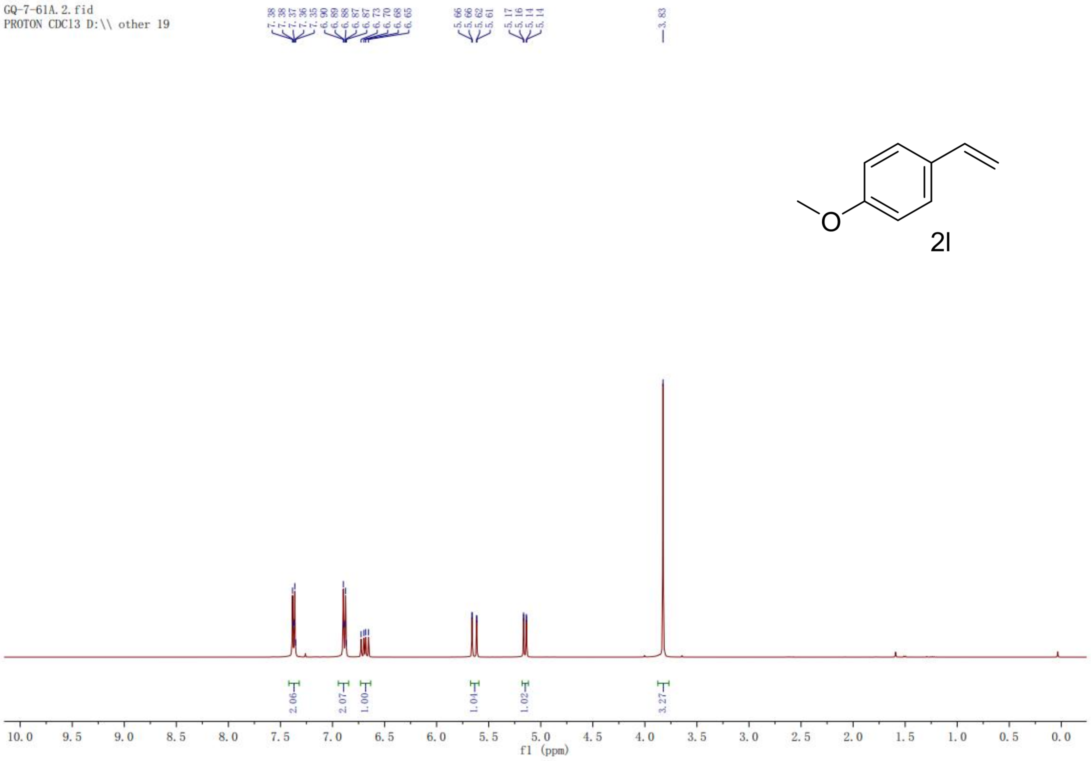
GQ-7-61A.2.fid
PROTON CDCl3 D:\\ other 19
7.38 7.38 7.37 7.36 7.35 6.90 6.89 6.88 6.87 6.87 6.73 6.70 6.68 6.65
5.66 5.66 5.62 5.61 5.17 5.16 5.14 5.14
3.83
O
2.06 2.07 1.00 1.04 1.02 3.27
f1 (ppm)

2l

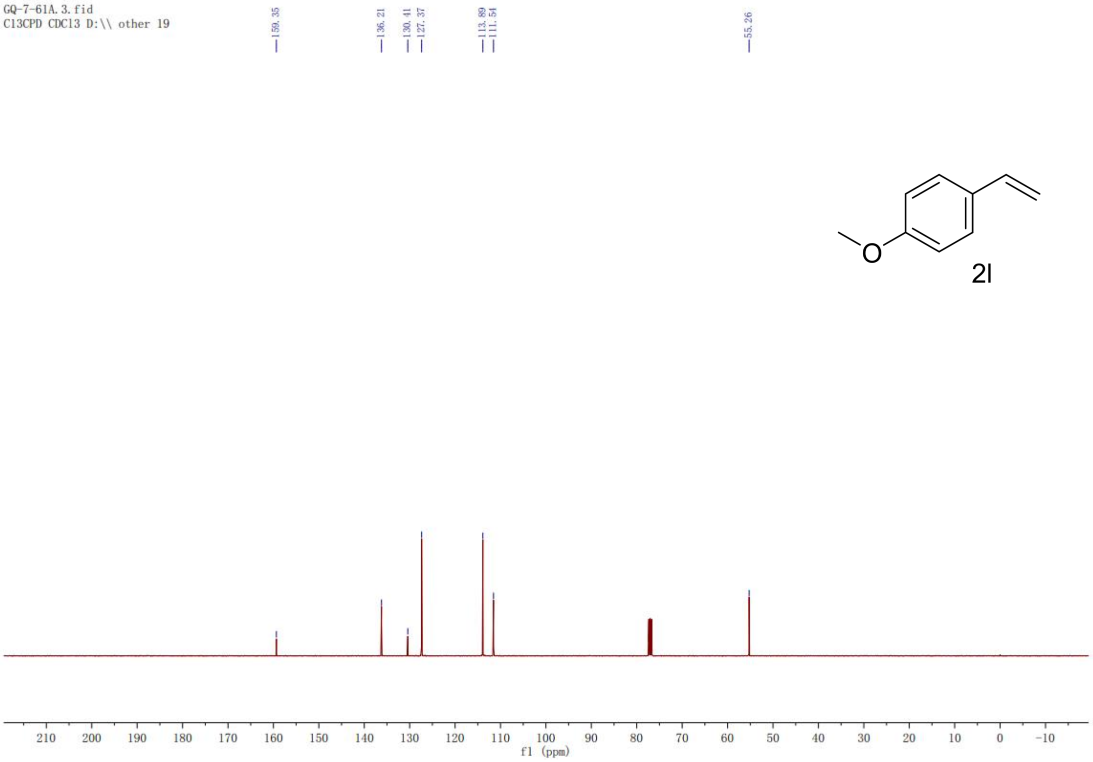
GQ-7-61A.3.fid
C13CPD CDCl3 D:\\ other 19
159.35
136.21 130.41 127.37
113.89 111.54
55.26
O
f1 (ppm)

2l

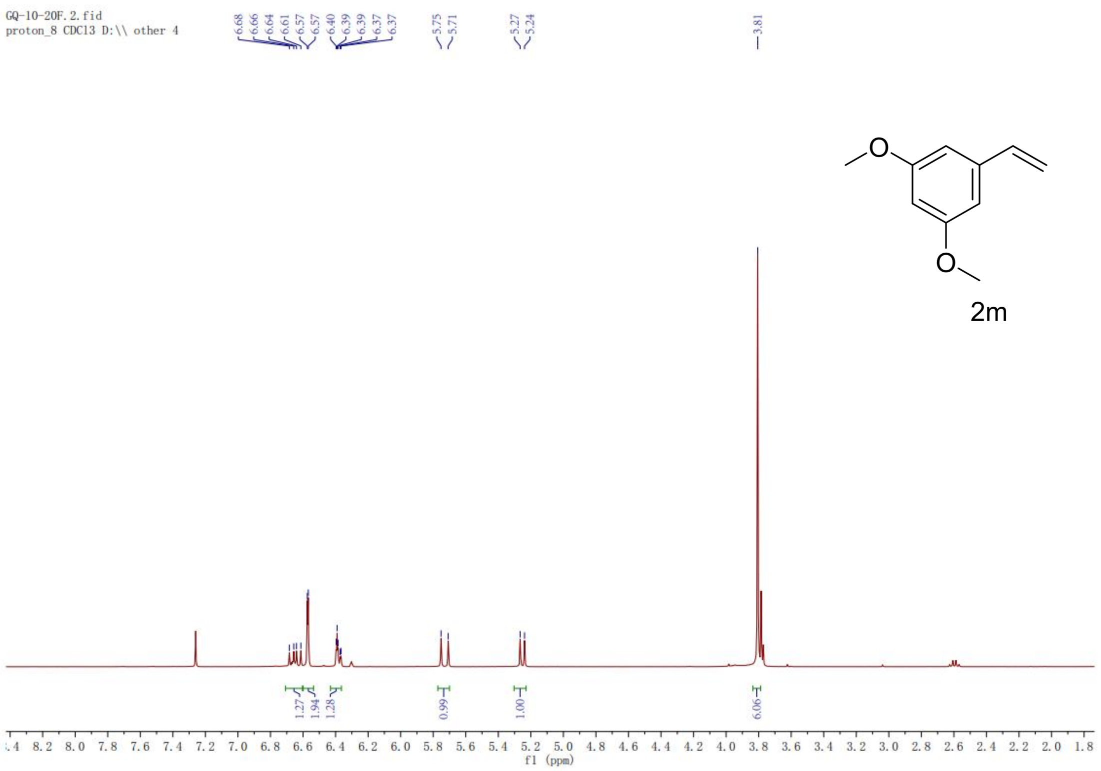
GQ-10-20F.2.fid
proton_8 CDCl3 D:\\ other 4
6.68
6.66
6.64
6.61
6.57
6.57
6.40
6.39
6.39
6.37
6.37
5.75
5.71
5.27
5.24
3.81
2m
1.27
1.94
1.28
0.99
1.00
6.06
f1 (ppm)

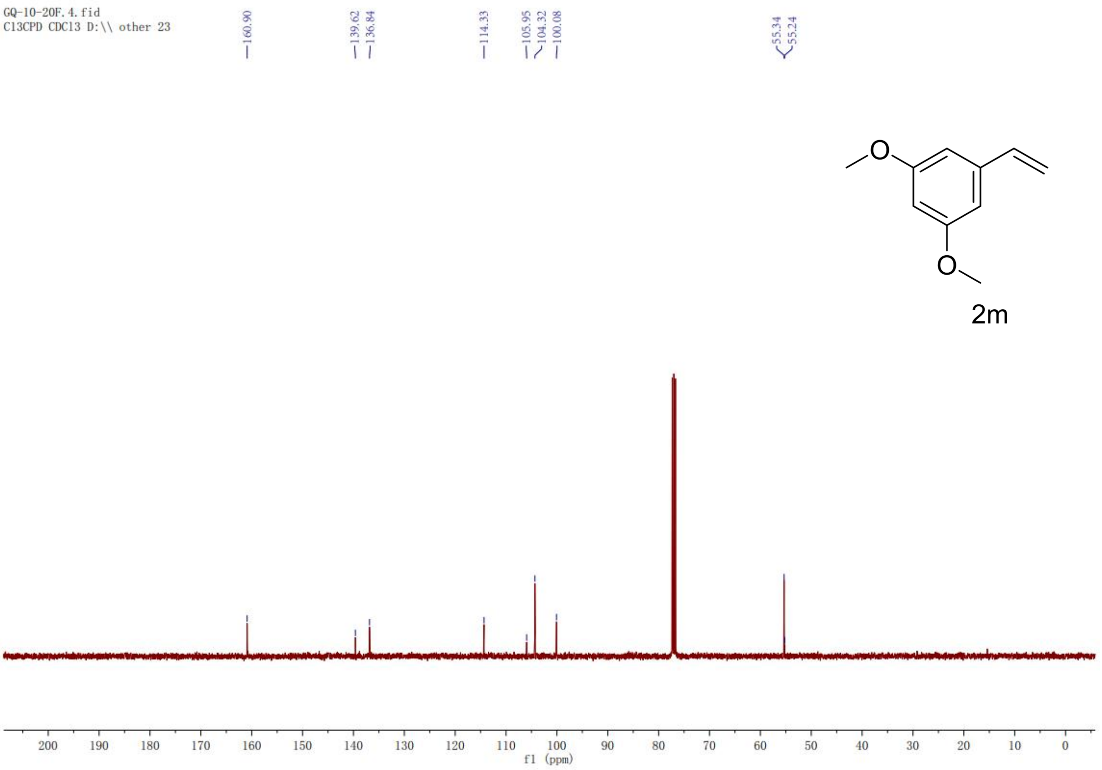
GQ-10-20F.4.fid
C13CPD CDCl3 D:\\ other 23
160.90
139.62
136.84
114.33
105.95
104.32
100.08
55.34
55.24
2m
f1 (ppm)

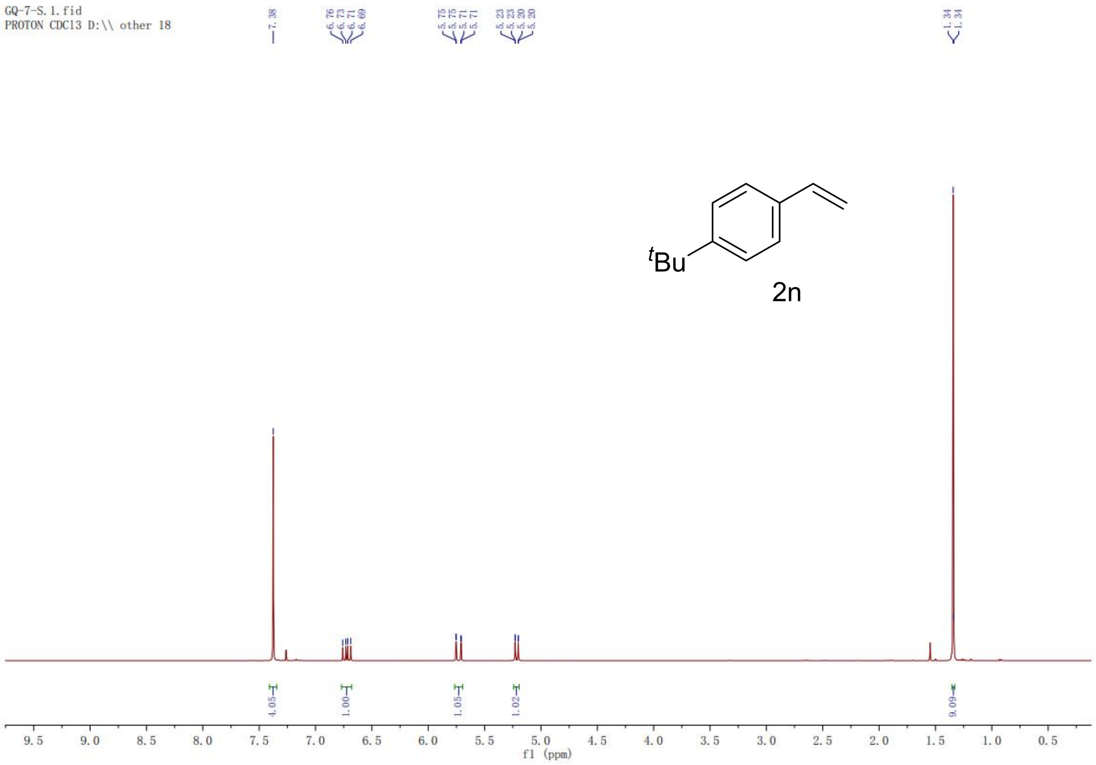
GQ-7-S.1.fid
PROTON CDCl3 D:\\ other 18
7.38
6.76
6.73
6.71
6.69
5.75
5.75
5.71
5.71
5.23
5.23
5.20
5.20
1.34
1.34
tBu
2n
4.05
1.00
1.05
1.02
9.09
9.5 9.0 8.5 8.0 7.5 7.0 6.5 6.0 5.5 5.0 4.5 4.0 3.5 3.0 2.5 2.0 1.5 1.0 0.5
f1 (ppm)

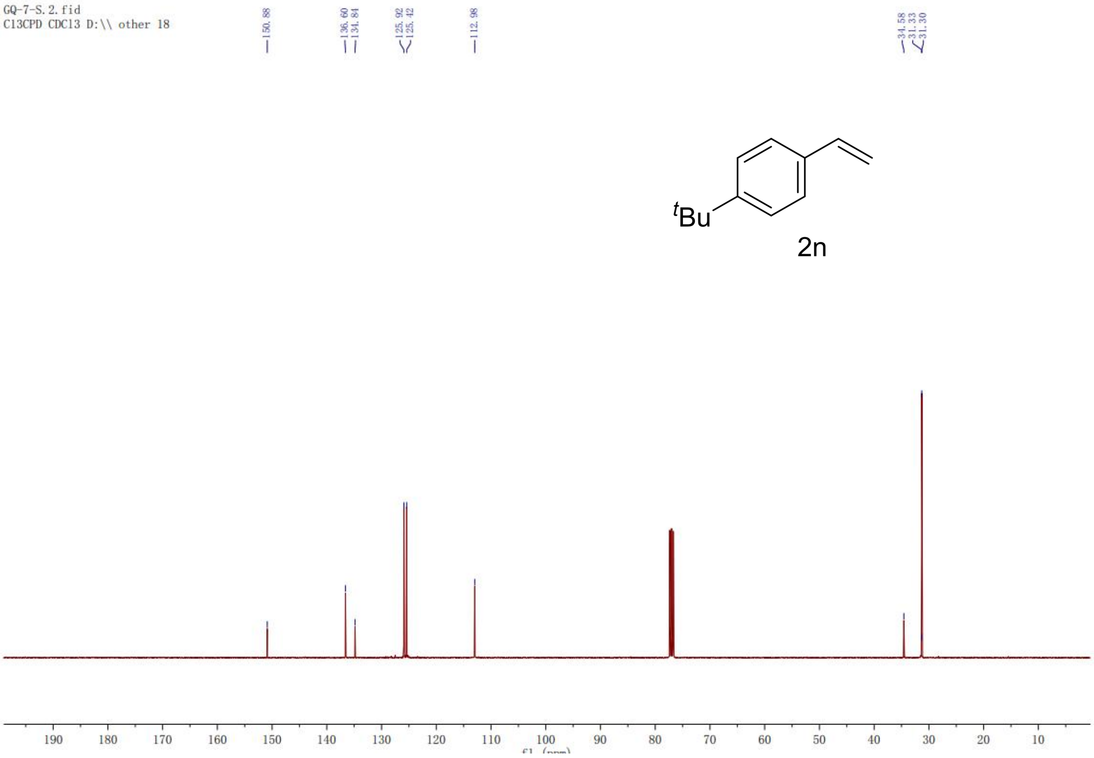
GQ-7-S.2.fid
C13CPD CDCl3 D:\\ other 18
150.88
136.60
134.84
125.92
125.42
112.98
34.58
31.33
31.30
tBu
2n
190 180 170 160 150 140 130 120 110 100 90 80 70 60 50 40 30 20 10

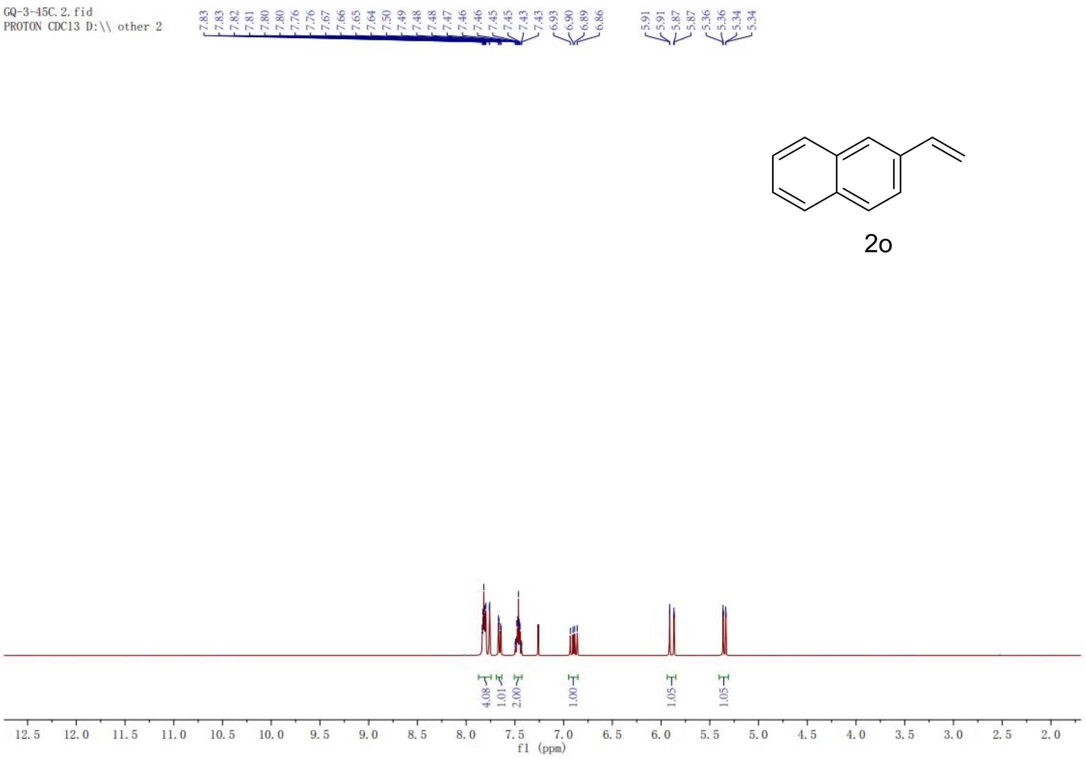
GQ-3-45C.2.fid
PROTON CDCl3 D:\\ other 2
7.83 7.83 7.82 7.81 7.80 7.80 7.76 7.76 7.67 7.66 7.65 7.64 7.50 7.49 7.48 7.48 7.47 7.46 7.46 7.45 7.45 7.43 7.43 6.93 6.90 6.89 6.86 5.91 5.91 5.87 5.87 5.36 5.36 5.34 5.34
4.08 1.01 2.00 1.00 1.05 1.05
12.5 12.0 11.5 11.0 10.5 10.0 9.5 9.0 8.5 8.0 7.5 7.0 6.5 6.0 5.5 5.0 4.5 4.0 3.5 3.0 2.5 2.0
f1 (ppm)

2o

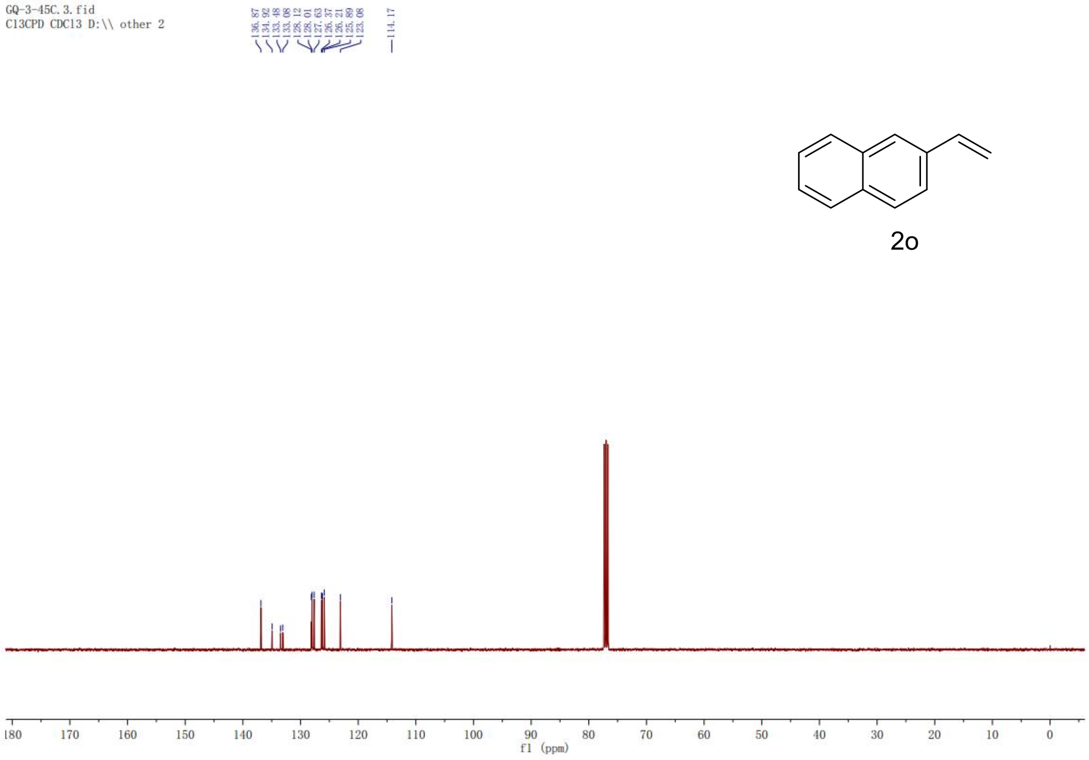
GQ-3-45C.3.fid
C13CPD CDCl3 D:\\ other 2
136.87 134.92 133.48 133.08 128.12 128.01 127.63 126.37 126.21 125.89 123.08
114.17
180 170 160 150 140 130 120 110 100 90 80 70 60 50 40 30 20 10 0
f1 (ppm)

2o

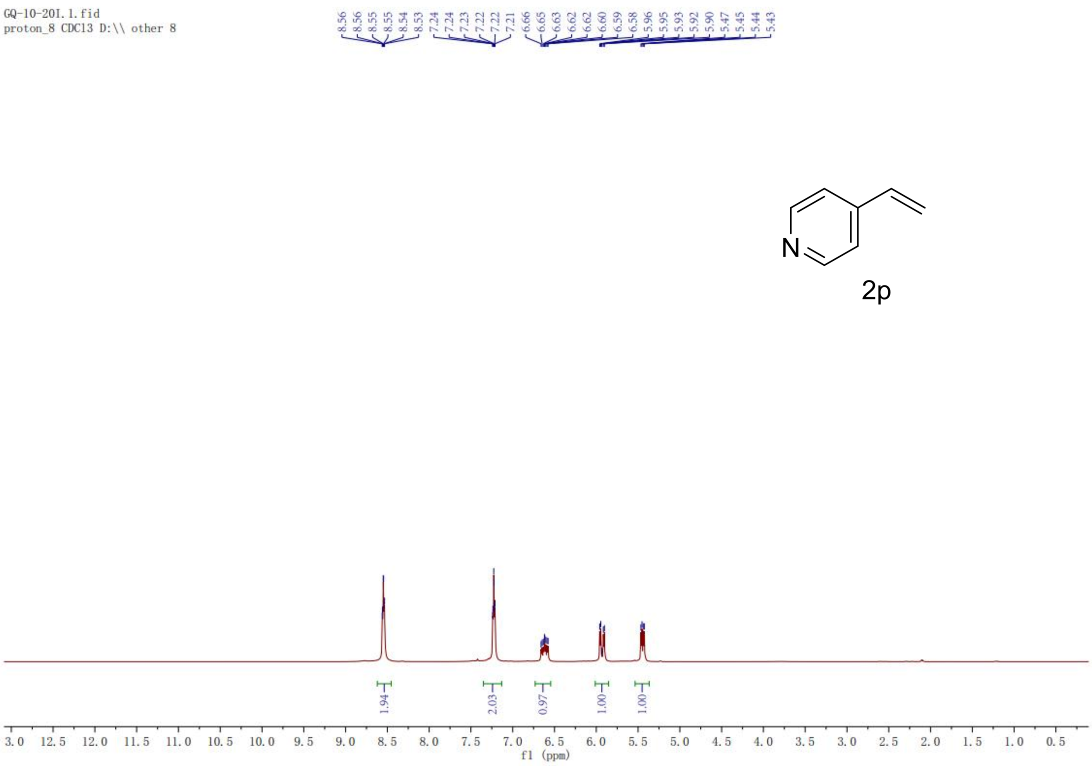
GQ-10-20I.1.fid
proton_8 CDCl3 D:\\ other 8
8.56 8.56 8.55 8.55 8.54 8.53 7.24 7.24 7.23 7.23 7.22 7.22 7.21 6.66 6.65 6.63 6.62 6.62 6.60 6.59 6.58 5.96 5.95 5.93 5.92 5.90 5.47 5.45 5.44 5.43
N
2p
1.94 2.03 0.97 1.00 1.00
f1 (ppm)

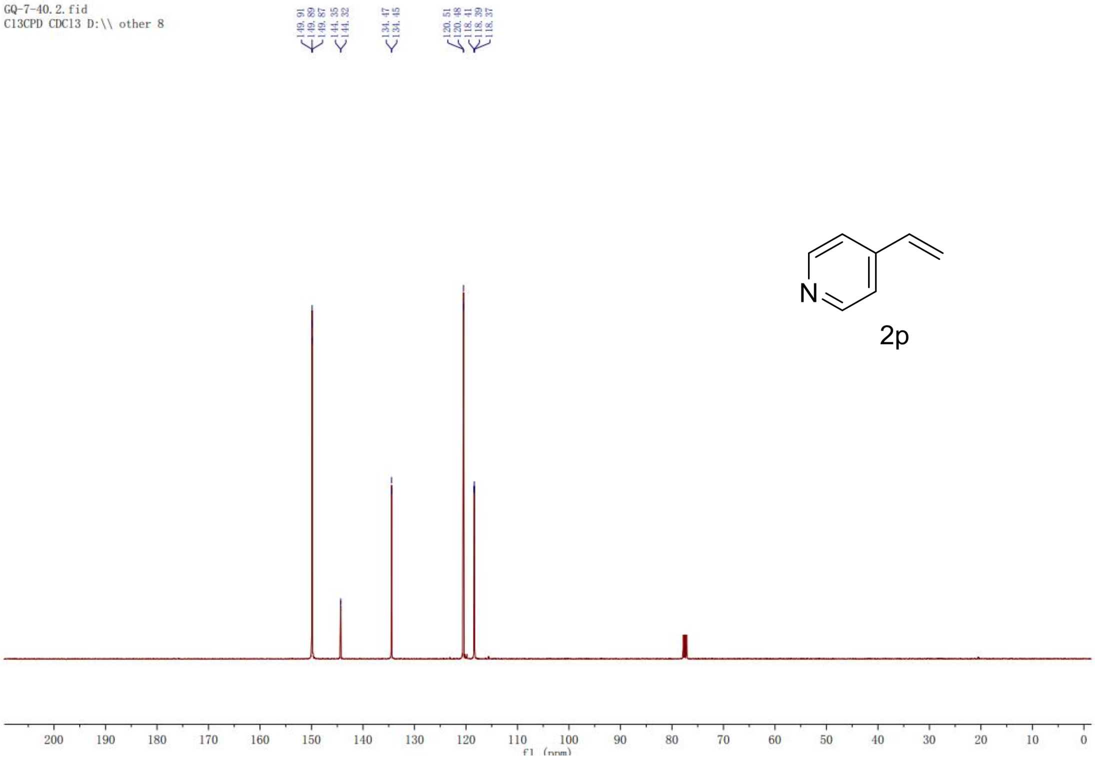
GQ-7-40.2.fid
C13CPD CDCl3 D:\\ other 8
149.91 149.89 149.87 144.35 144.32 134.47 134.45 120.51 120.48 118.41 118.39 118.37
N
2p
f1 (ppm)

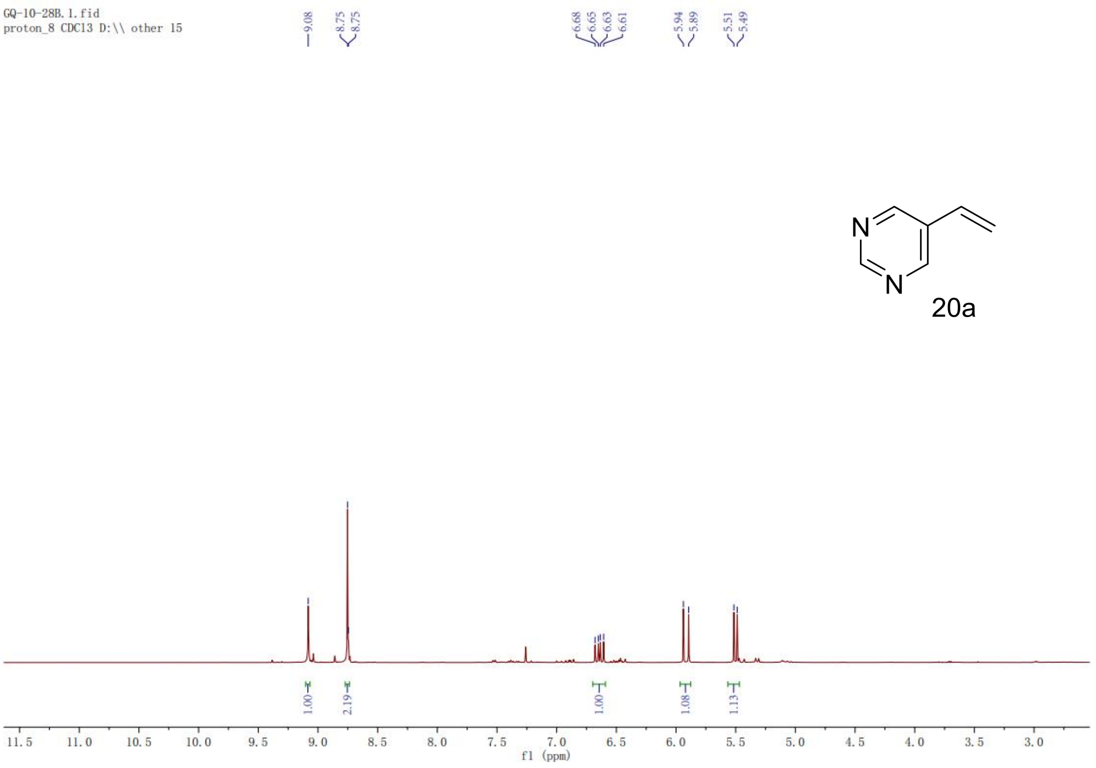
GQ-10-28B.1.fid
proton_8 CDCl3 D:\\ other 15
9.08
8.75
8.75
6.68
6.65
6.63
6.61
5.94
5.89
5.51
5.49
N
N
20a
1.00
2.19
1.00
1.08
1.13
11.5 11.0 10.5 10.0 9.5 9.0 8.5 8.0 7.5 7.0 6.5 6.0 5.5 5.0 4.5 4.0 3.5 3.0
f1 (ppm)

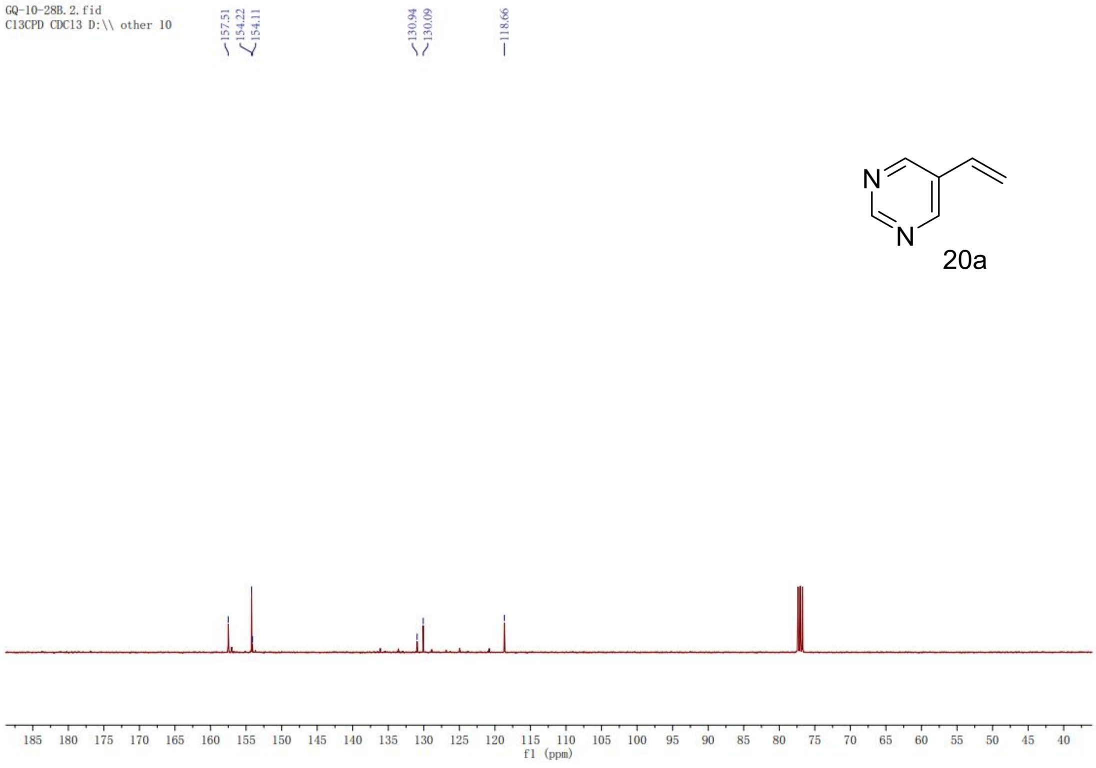
GQ-10-28B.2.fid
C13CPD CDCl3 D:\\ other 10
157.51
154.22
154.11
130.94
130.09
118.66
N
N
20a
185 180 175 170 165 160 155 150 145 140 135 130 125 120 115 110 105 100 95 90 85 80 75 70 65 60 55 50 45 40
f1 (ppm)

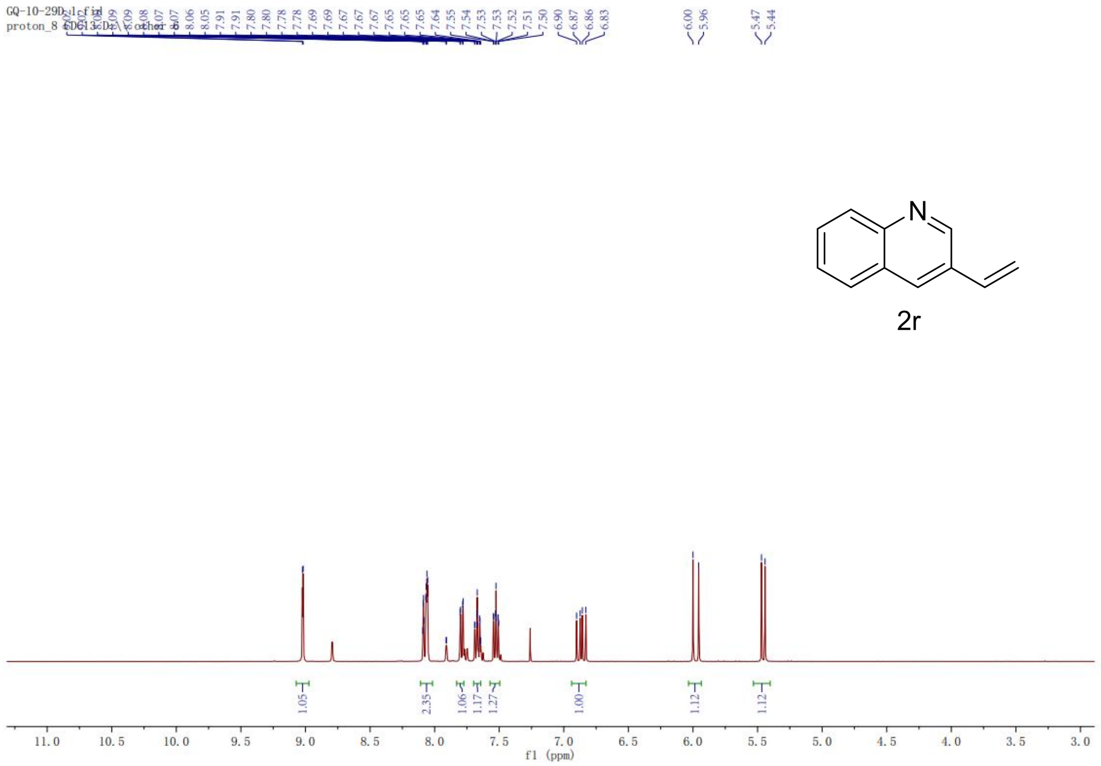

GQ-10-29D.1.fid
proton_8 CDCl3
8.09
8.08
8.07
8.07
8.06
8.05
7.91
7.91
7.80
7.80
7.78
7.78
7.69
7.69
7.67
7.67
7.67
7.65
7.65
7.65
7.64
7.55
7.54
7.53
7.53
7.52
7.51
7.50
6.90
6.87
6.86
6.83
6.00
5.96
5.47
5.44
N
2r
1.05
2.35
1.06
1.17
1.27
1.00
1.12
1.12
11.0 10.5 10.0 9.5 9.0 8.5 8.0 7.5 7.0 6.5 6.0 5.5 5.0 4.5 4.0 3.5 3.0
f1 (ppm)


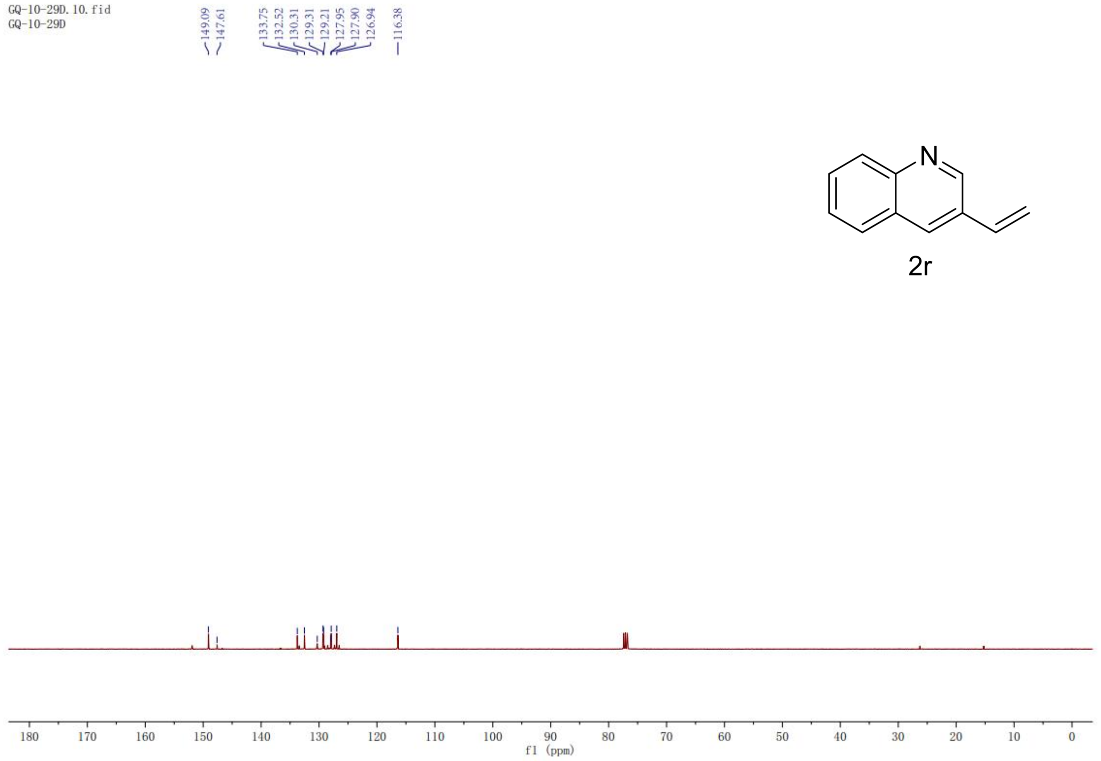

GQ-10-29D.10.fid
GQ-10-29D
149.09
147.61
133.75
132.52
130.31
129.31
129.21
127.95
127.90
126.94
116.38
N
2r
180 170 160 150 140 130 120 110 100 90 80 70 60 50 40 30 20 10 0
f1 (ppm)

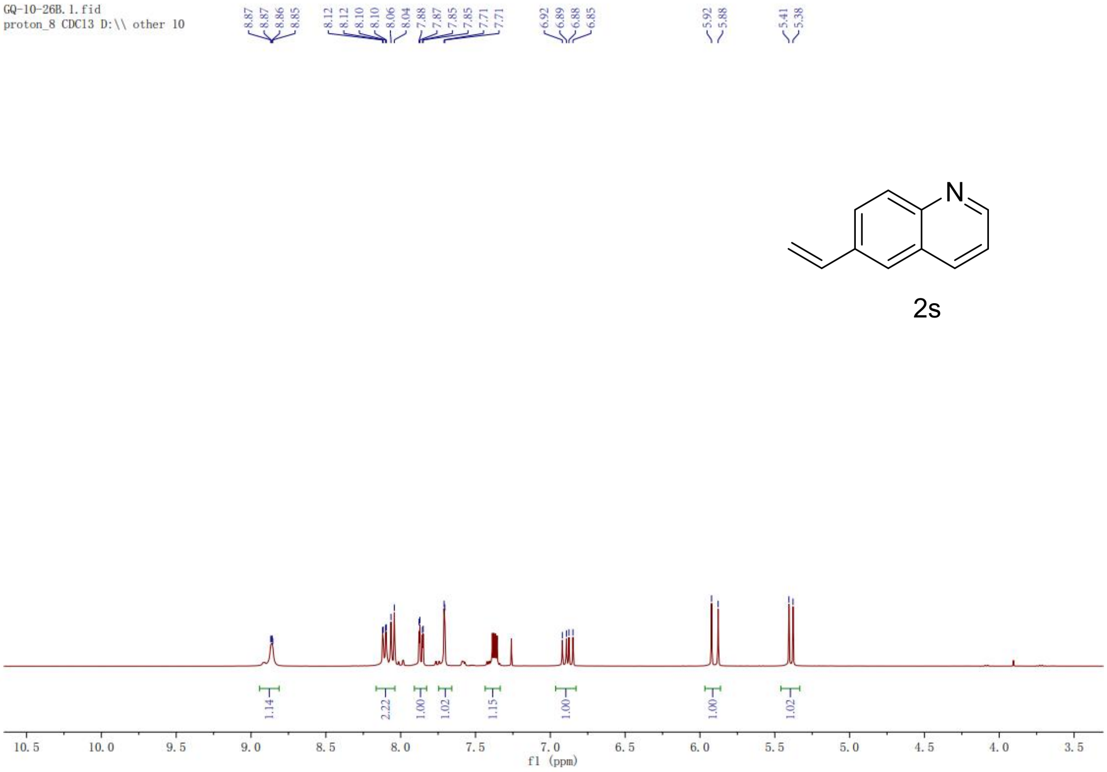
GQ-10-26B.1.fid
proton_8 CDCl3 D:\\ other 10
8.87
8.87
8.86
8.85
8.12
8.12
8.10
8.10
8.06
8.04
7.88
7.87
7.85
7.85
7.71
7.71
6.92
6.89
6.88
6.85
5.92
5.88
5.41
5.38
N
2s
1.14
2.22
1.00
1.02
1.15
1.00
1.00
1.02
10.5
10.0
9.5
9.0
8.5
8.0
7.5
7.0
6.5
6.0
5.5
5.0
4.5
4.0
3.5
f1 (ppm)

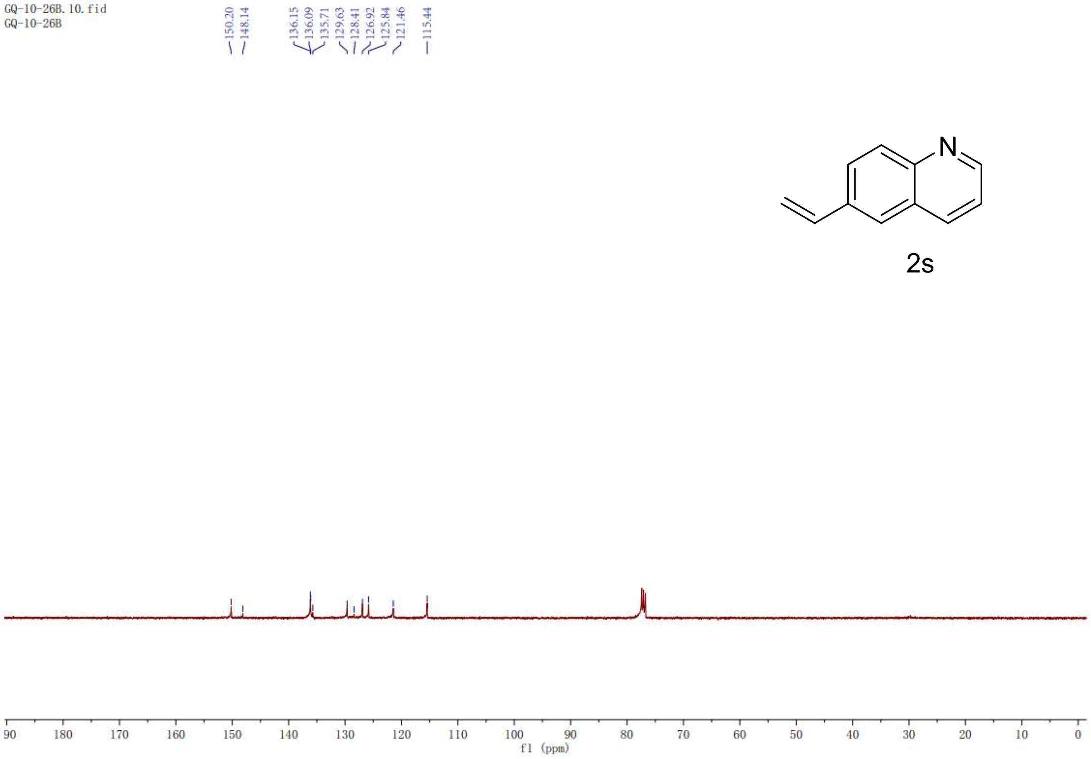
GQ-10-26B.10.fid
GQ-10-26B
150.20
148.14
136.15
136.09
135.71
129.63
128.41
126.92
125.84
121.46
115.44
N
2s
90
180
170
160
150
140
130
120
110
100
90
80
70
60
50
40
30
20
10
0
f1 (ppm)

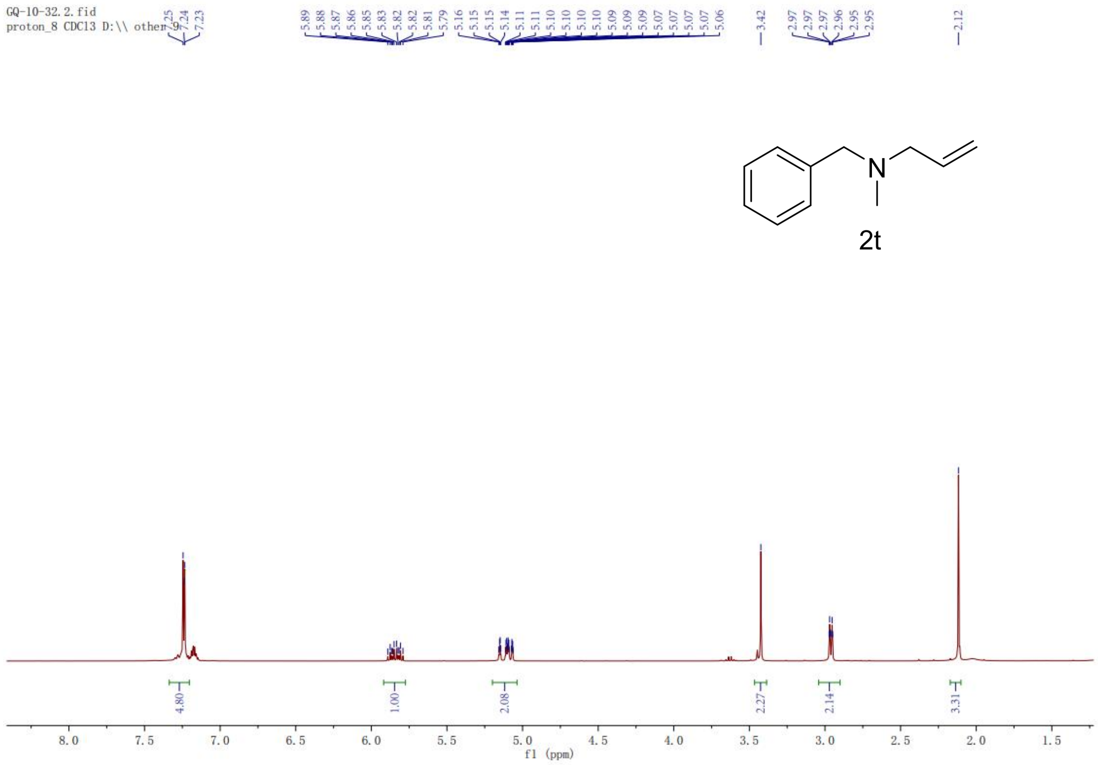
GQ-10-32.2.fid
proton_8 CDCl3 D:\\ other 9
7.25
7.24
7.23
5.89
5.88
5.87
5.86
5.85
5.83
5.82
5.82
5.81
5.79
5.16
5.15
5.15
5.14
5.11
5.11
5.10
5.10
5.10
5.10
5.09
5.09
5.09
5.07
5.07
5.07
5.07
5.06
3.42
2.97
2.97
2.97
2.96
2.95
2.95
2.12
N
2t
4.80
1.00
2.08
2.27
2.14
3.31
8.0
7.5
7.0
6.5
6.0
5.5
5.0
4.5
4.0
3.5
3.0
2.5
2.0
1.5
f1 (ppm)

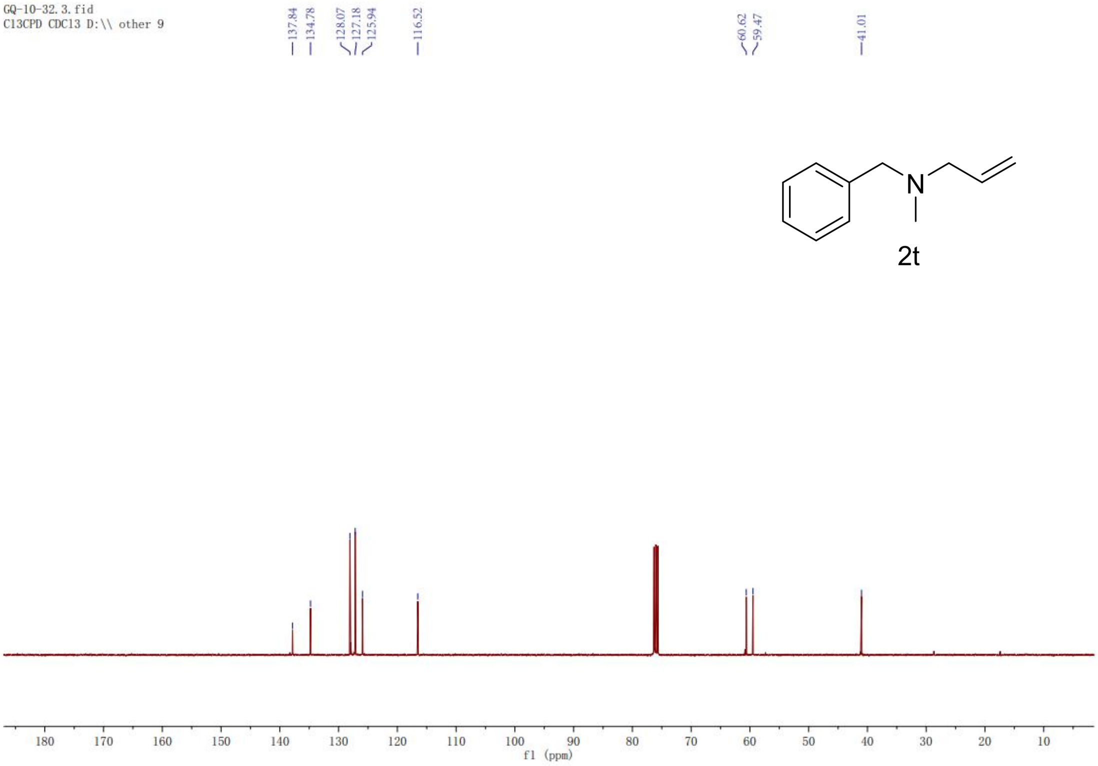
GQ-10-32.3.fid
C13CPD CDCl3 D:\\ other 9
137.84
134.78
128.07
127.18
125.94
116.52
60.62
59.47
41.01
N
2t
180
170
160
150
140
130
120
110
100
90
80
70
60
50
40
30
20
10
f1 (ppm)

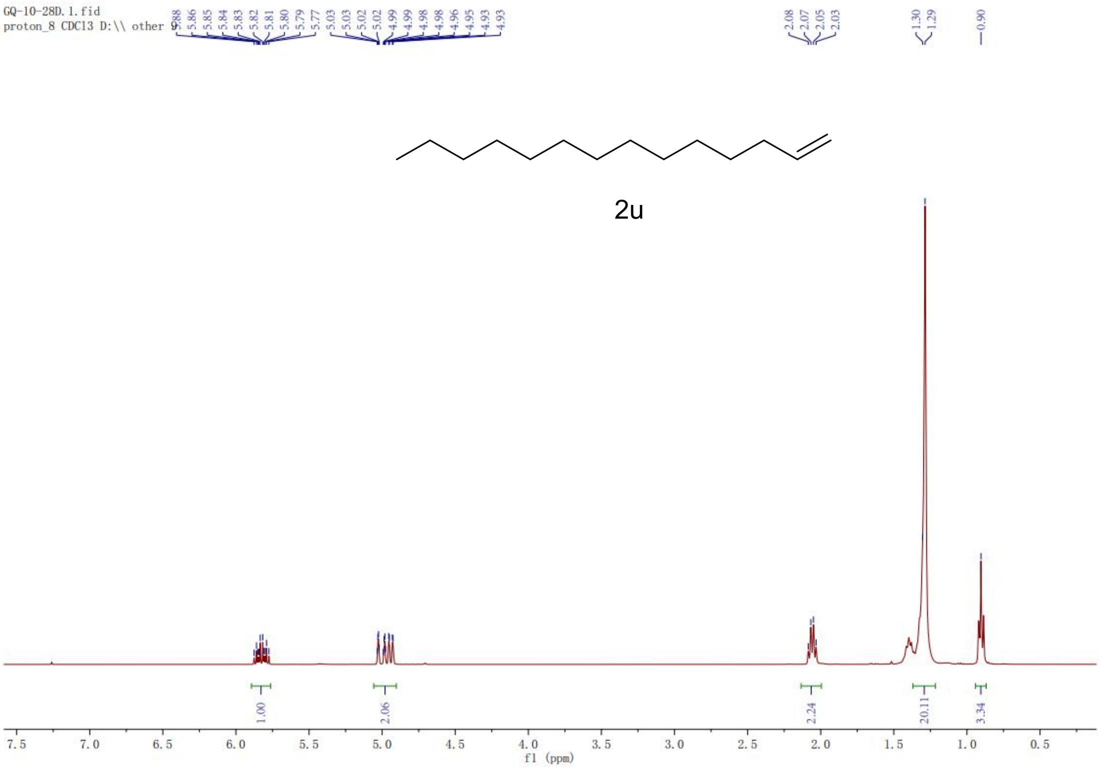
GQ-10-28D.1.fid
proton_8 CDCl3 D:\\ other
5.88 5.86 5.85 5.84 5.83 5.82 5.81 5.80 5.79 5.77 5.03 5.03 5.02 5.02 4.99 4.99 4.98 4.98 4.96 4.95 4.93 4.93
2.08 2.07 2.05 2.03
1.30 1.29
0.90
2u
1.00
2.06
2.24
20.11
3.34
7.5 7.0 6.5 6.0 5.5 5.0 4.5 4.0 3.5 3.0 2.5 2.0 1.5 1.0 0.5
f1 (ppm)

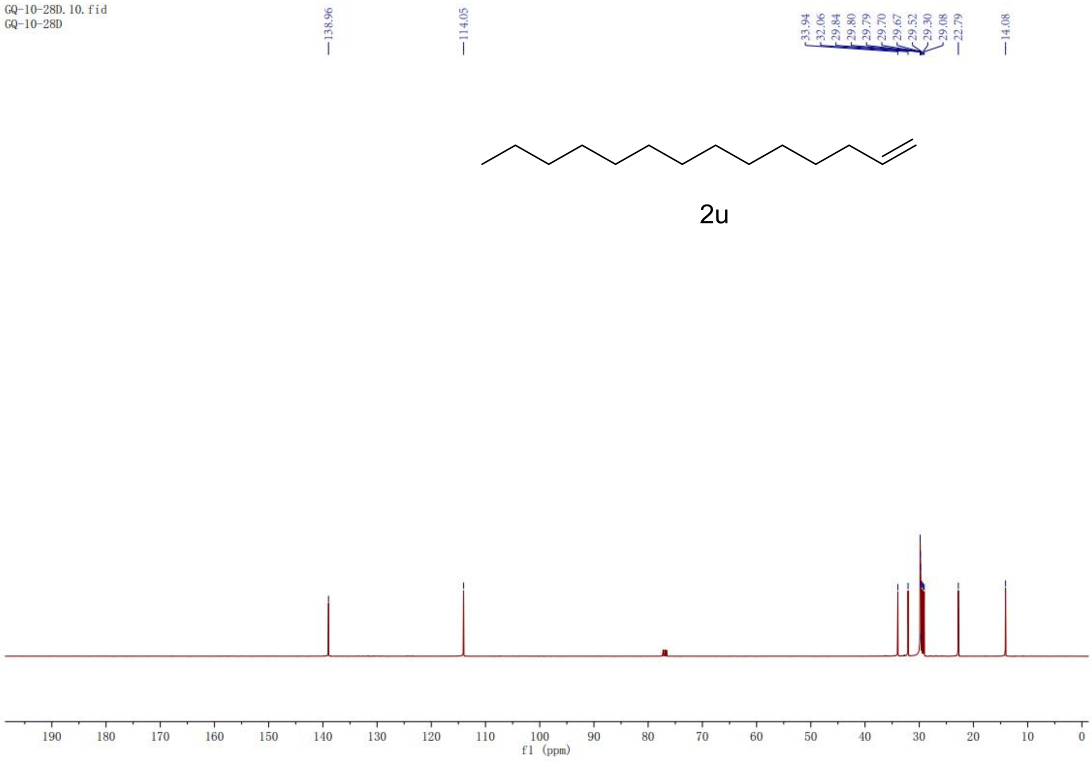
GQ-10-28D.10.fid
GQ-10-28D
138.96
114.05
33.94 32.06 29.84 29.80 29.79 29.70 29.67 29.52 29.30 29.08
22.79
14.08
2u
190 180 170 160 150 140 130 120 110 100 90 80 70 60 50 40 30 20 10 0
f1 (ppm)

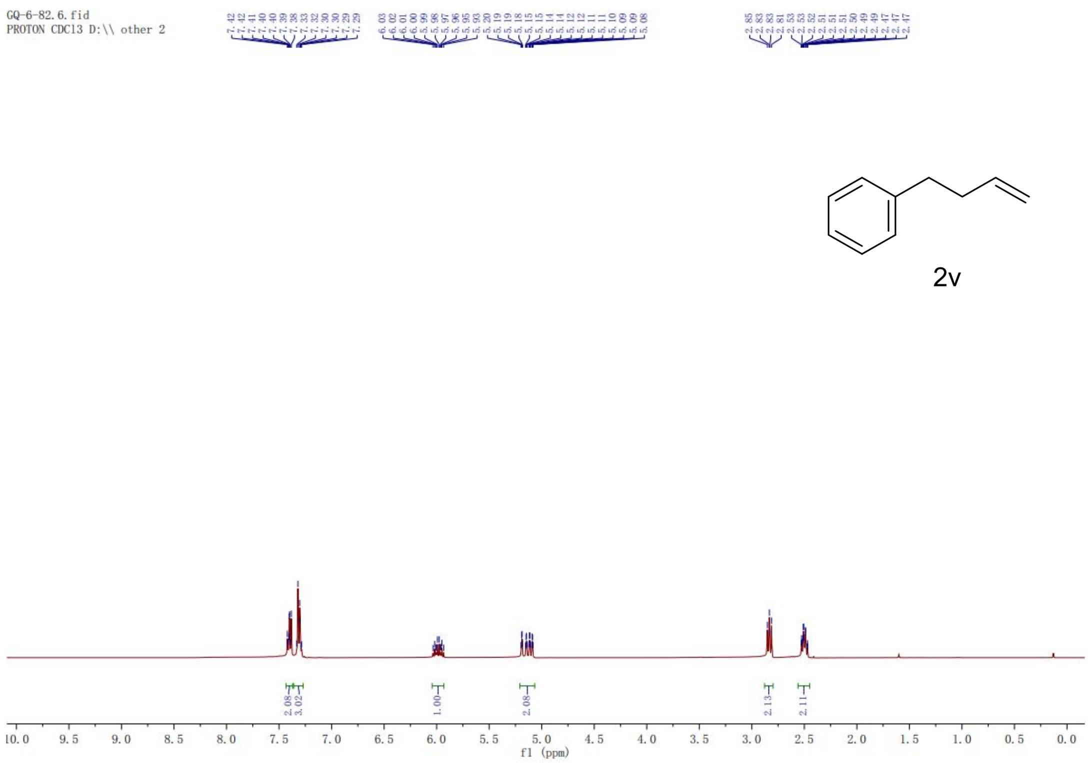
GQ-6-82.6.fid
PROTON CDCl3 D:\\ other 2
2v
2.08 3.02 1.00 2.08 2.13 2.11
10.0 9.5 9.0 8.5 8.0 7.5 7.0 6.5 6.0 5.5 5.0 4.5 4.0 3.5 3.0 2.5 2.0 1.5 1.0 0.5 0.0
f1 (ppm)

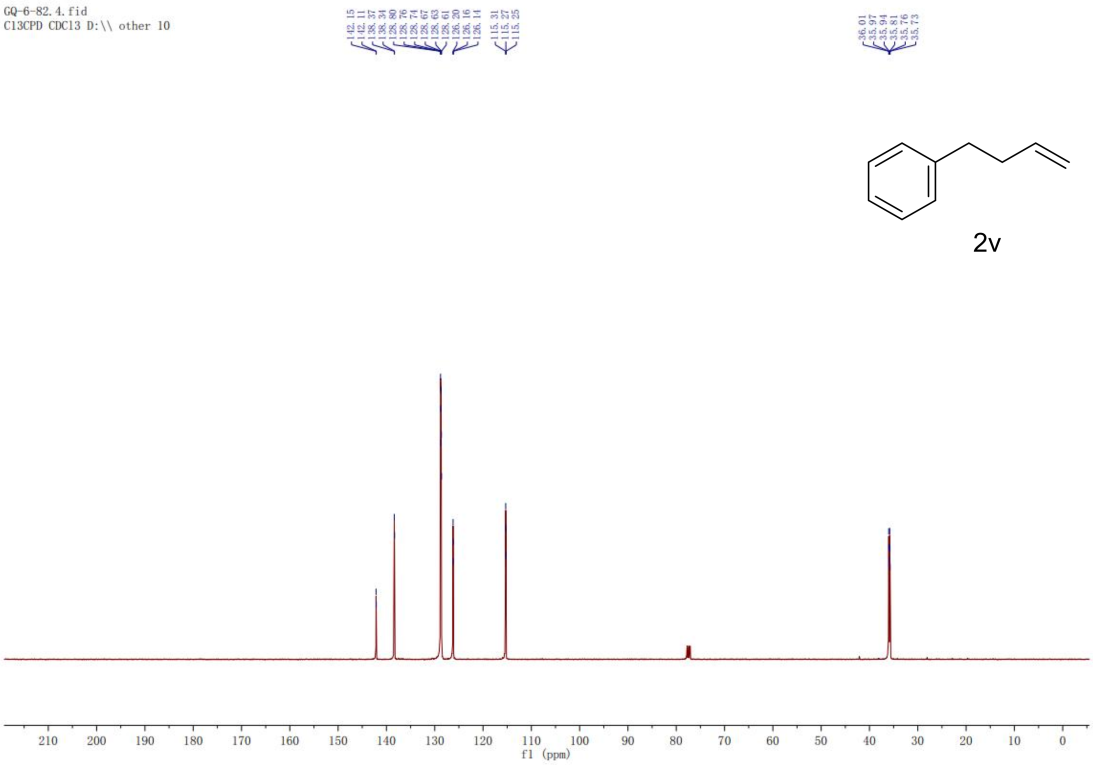
GQ-6-82.4.fid
C13CPD CDCl3 D:\\ other 10
142.15 142.11 138.37 138.34 128.80 128.76 128.74 128.67 128.63 128.61 126.20 126.16 126.14 115.31 115.27 115.25
36.01 35.97 35.94 35.81 35.76 35.73
2v
210 200 190 180 170 160 150 140 130 120 110 100 90 80 70 60 50 40 30 20 10 0
f1 (ppm)

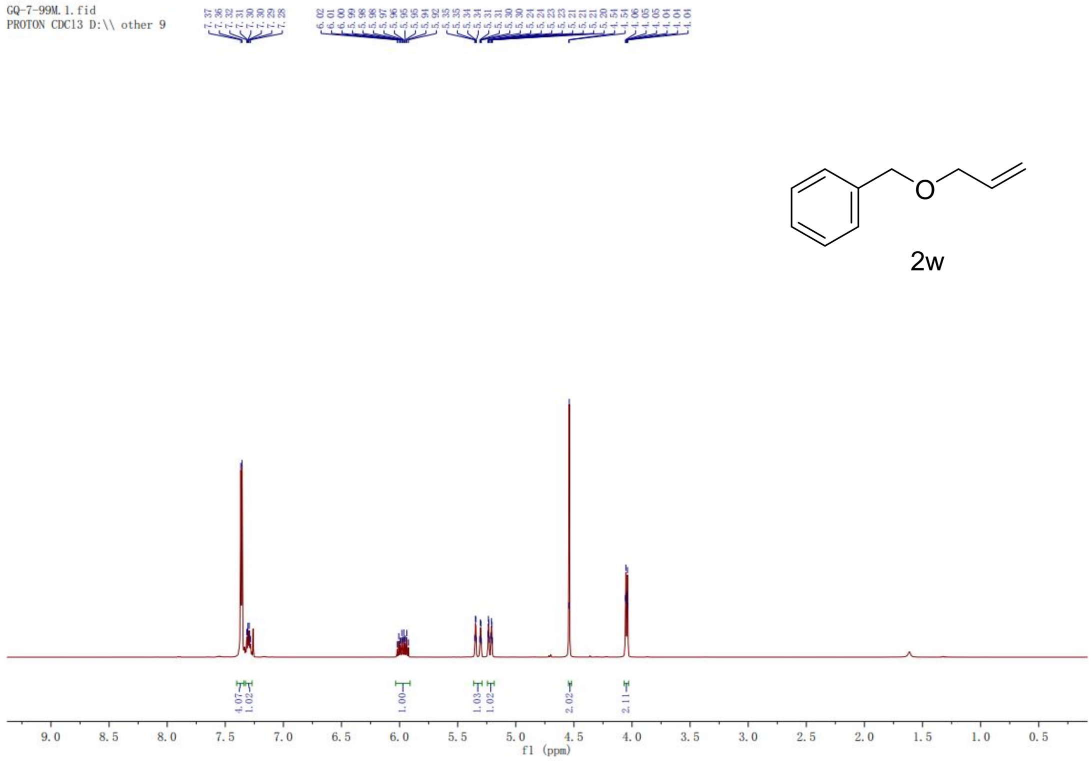
GQ-7-99M.1.fid
PROTON CDCl3 D:\\ other 9
4.07
1.02
1.00
1.03
1.02
2.02
2.11
f1 (ppm)

2w

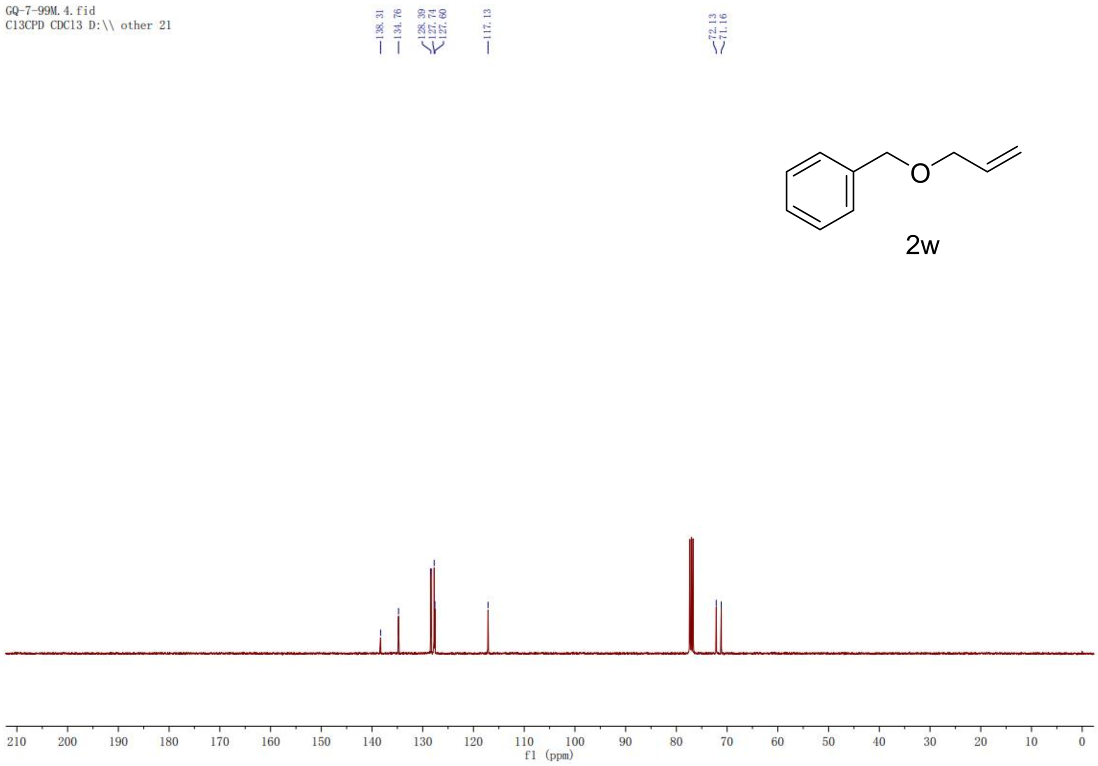
GQ-7-99M.4.fid
C13CPD CDCl3 D:\\ other 21
138.31
134.76
128.39
127.74
127.60
117.13
72.13
71.16
f1 (ppm)

2w

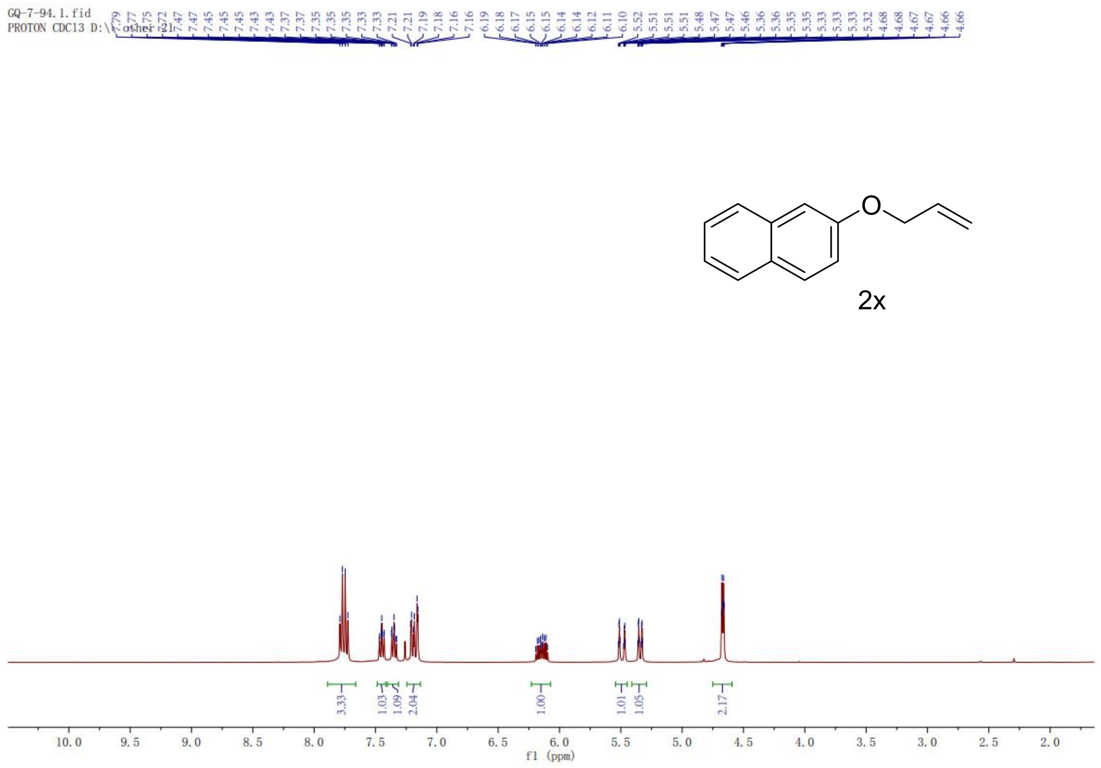
GQ-7-94.1.fid
PROTON CDCl3 D:\\ other 21
7.79 7.77 7.75 7.72 7.47 7.47 7.45 7.45 7.43 7.43 7.37 7.37 7.35 7.35 7.35 7.33 7.33 7.21 7.21 7.19 7.18 7.16 7.16 6.19 6.18 6.17 6.15 6.15 6.14 6.14 6.12 6.11 6.10 5.52 5.51 5.51 5.51 5.48 5.47 5.46 5.36 5.36 5.35 5.35 5.33 5.33 5.33 5.32 4.68 4.68 4.67 4.67 4.66 4.66
2x
3.33 1.03 1.09 2.04 1.00 1.01 1.05 2.17
10.0 9.5 9.0 8.5 8.0 7.5 7.0 6.5 6.0 5.5 5.0 4.5 4.0 3.5 3.0 2.5 2.0
f1 (ppm)

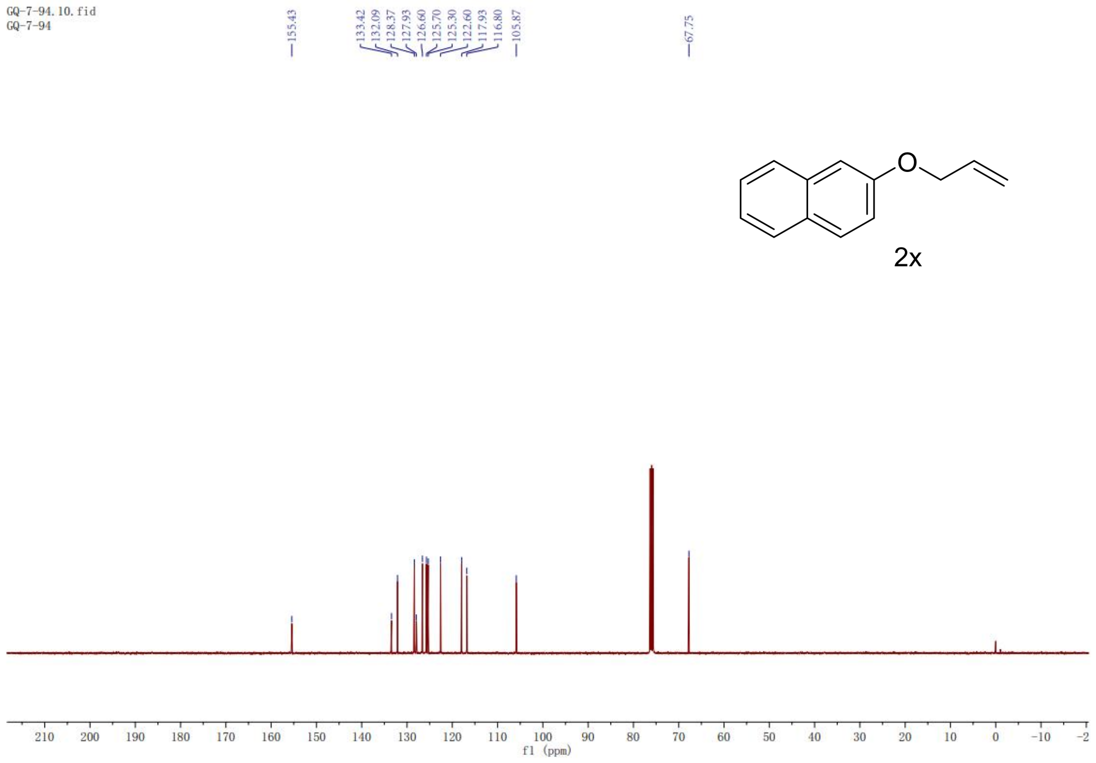
GQ-7-94.10.fid
GQ-7-94
155.43
133.42 132.09 128.37 127.93 126.60 125.70 125.30 122.60 117.93 116.80 105.87
67.75
2x
210 200 190 180 170 160 150 140 130 120 110 100 90 80 70 60 50 40 30 20 10 0 -10 -2
f1 (ppm)

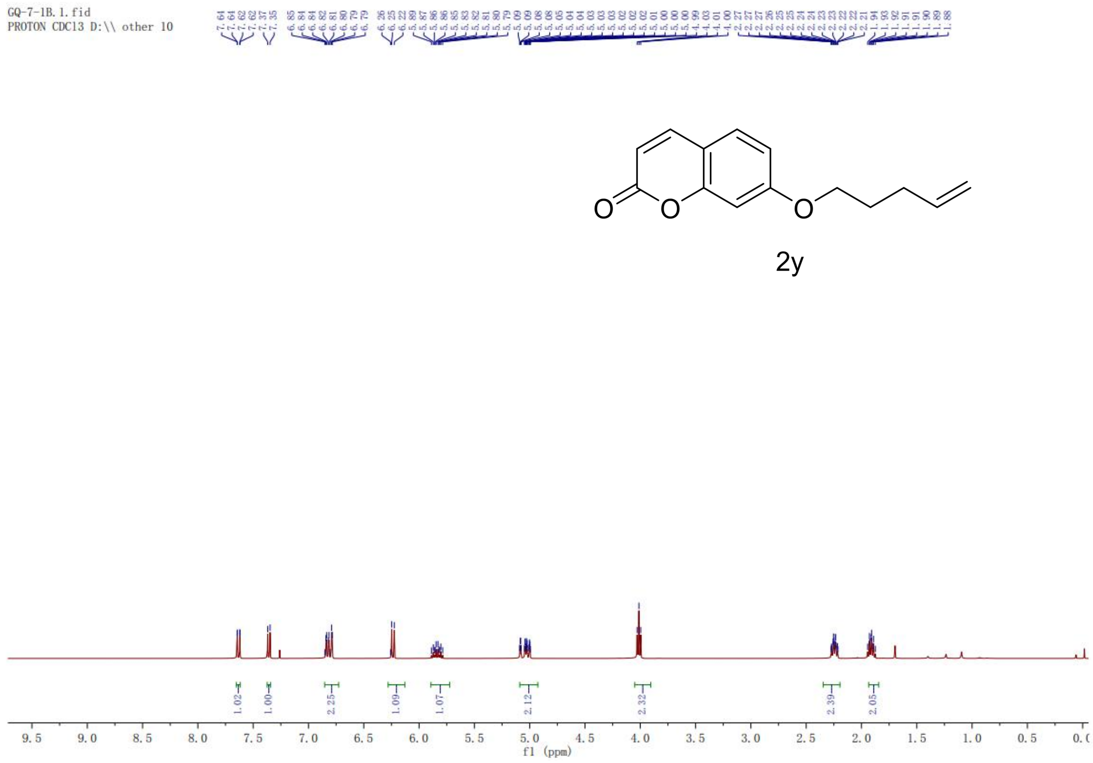
GQ-7-1B.1.fid
PROTON CDCl3 D:\\ other 10
2y
f1 (ppm)

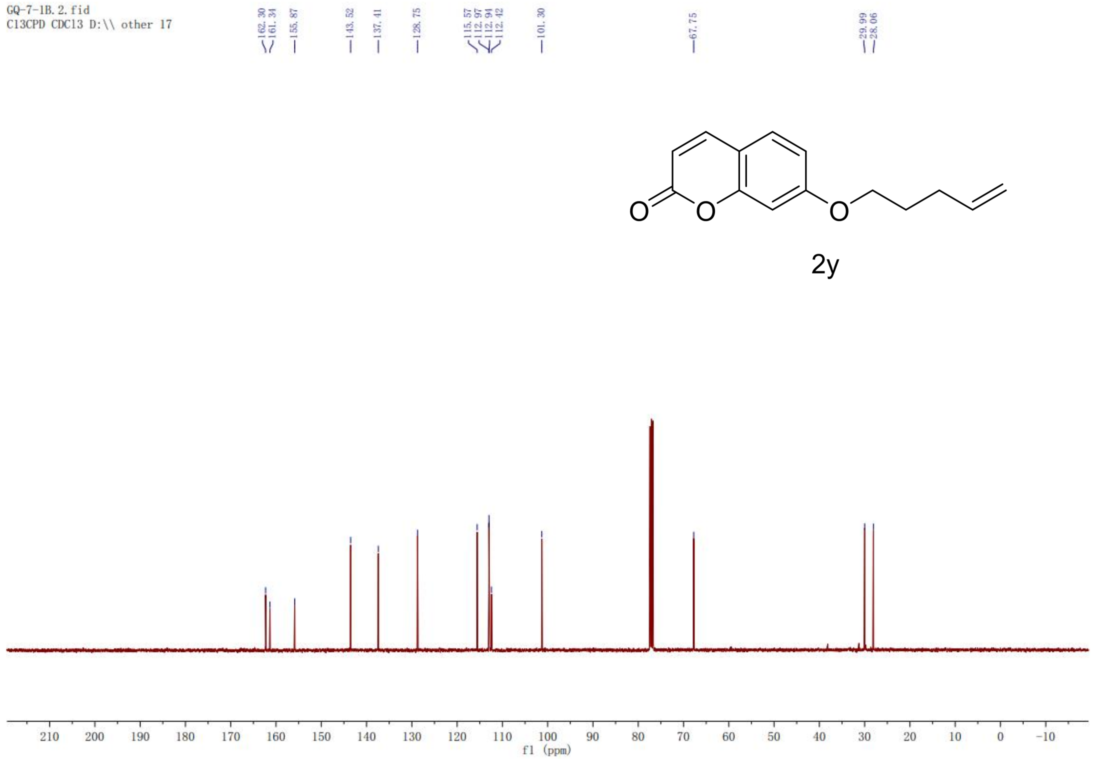
GQ-7-1B.2.fid
C13CPD CDCl3 D:\\ other 17
162.30
161.34
155.87
143.52
137.41
128.75
115.57
112.97
112.91
112.42
101.30
67.75
29.99
28.06
2y
f1 (ppm)

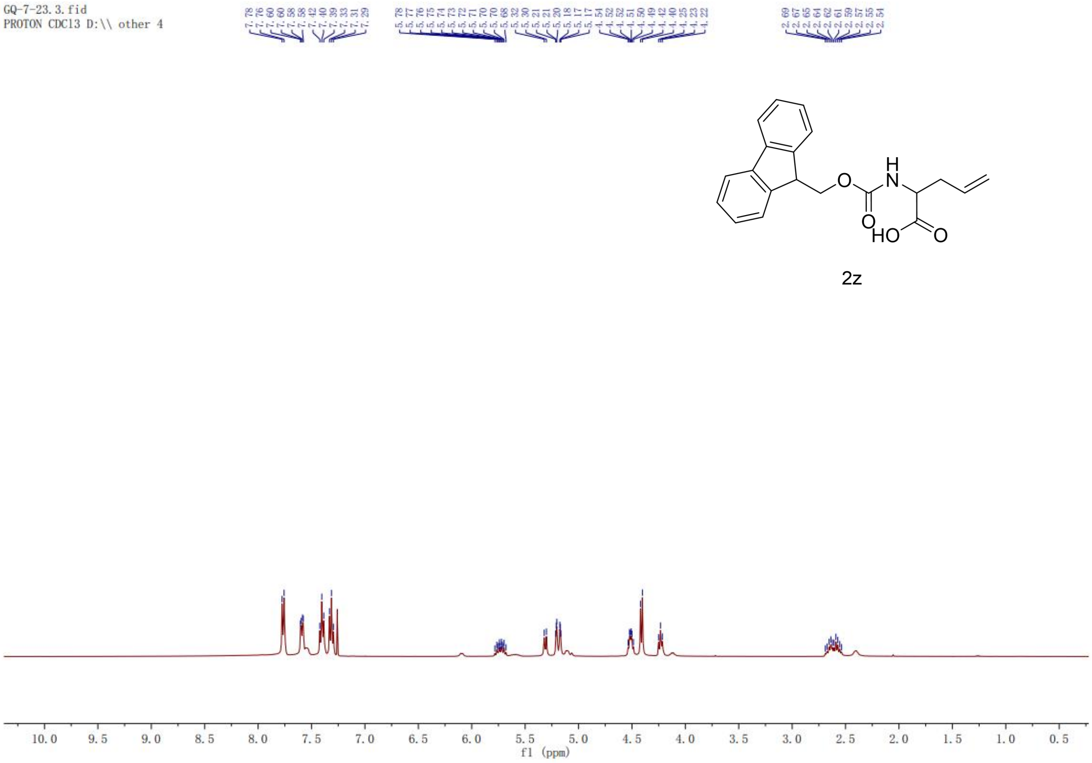
GQ-7-23.3.fid
PROTON CDCl3 D:\\ other 4
7.78 7.76 7.60 7.58 7.42 7.40 7.39 7.33 7.31 7.29
5.78 5.77 5.76 5.75 5.74 5.73 5.72 5.71 5.70 5.70 5.68 5.32 5.30 5.21 5.20 5.18 5.17 4.54 4.52 4.51 4.50 4.49 4.42 4.40 4.25 4.23 4.22
2.69 2.67 2.65 2.64 2.62 2.61 2.59 2.57 2.55 2.54
H
N
O
O
HO
O
2z
f1 (ppm)

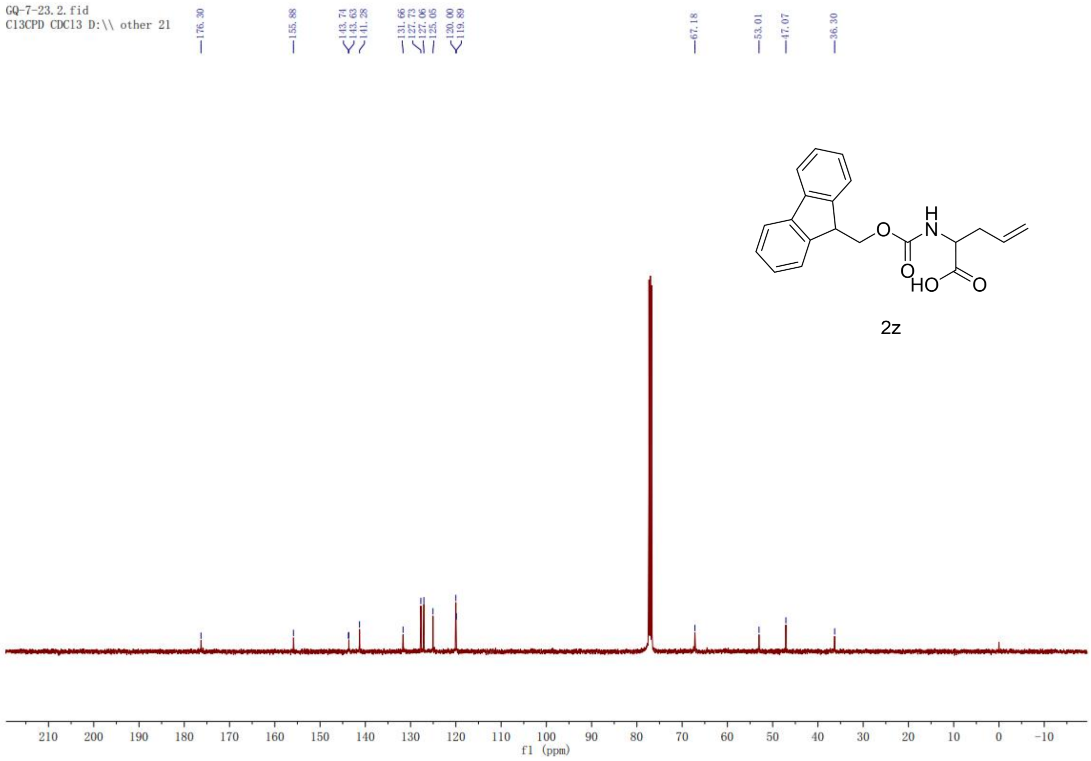
GQ-7-23.2.fid
C13CPD CDCl3 D:\\ other 21
176.30
155.88
143.71 143.63 141.28
131.66 127.73 127.06 125.05 120.00 119.89
67.18
53.01
47.07
36.30
H
N
O
O
HO
O
2z
f1 (ppm)

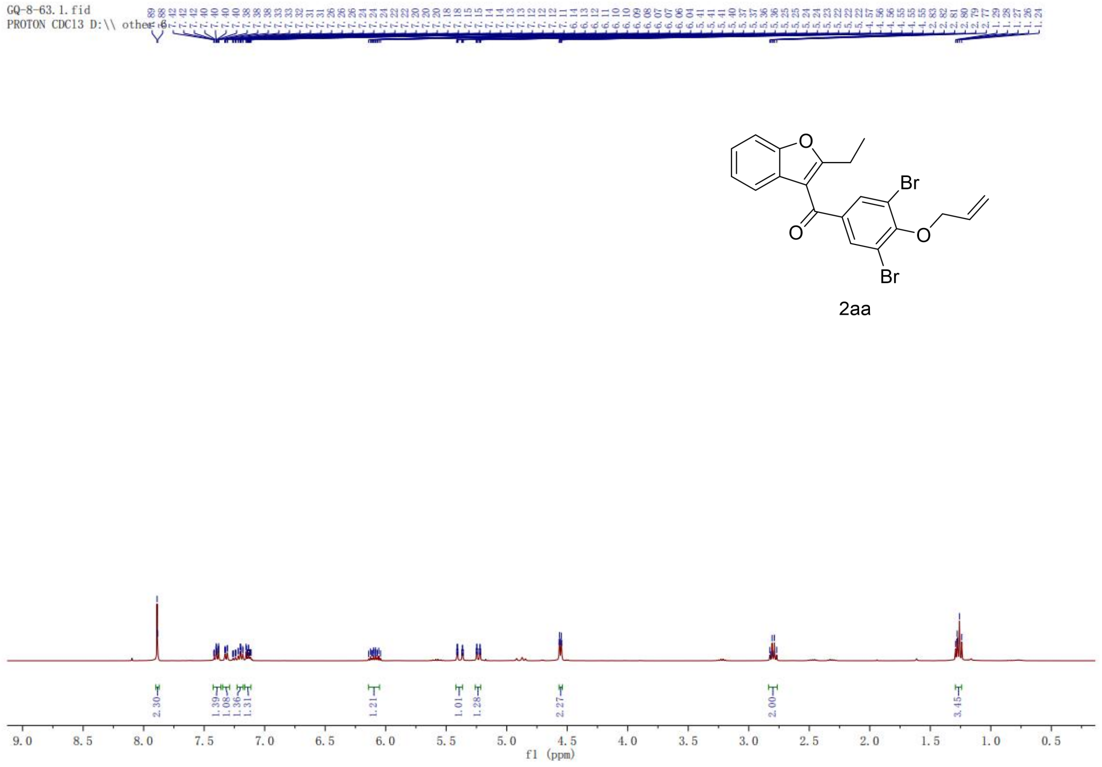
GQ-8-63.1.fid
PROTON CDCl3 D:\\ other 6
Br
O
Br
O
O
2aa
f1 (ppm)

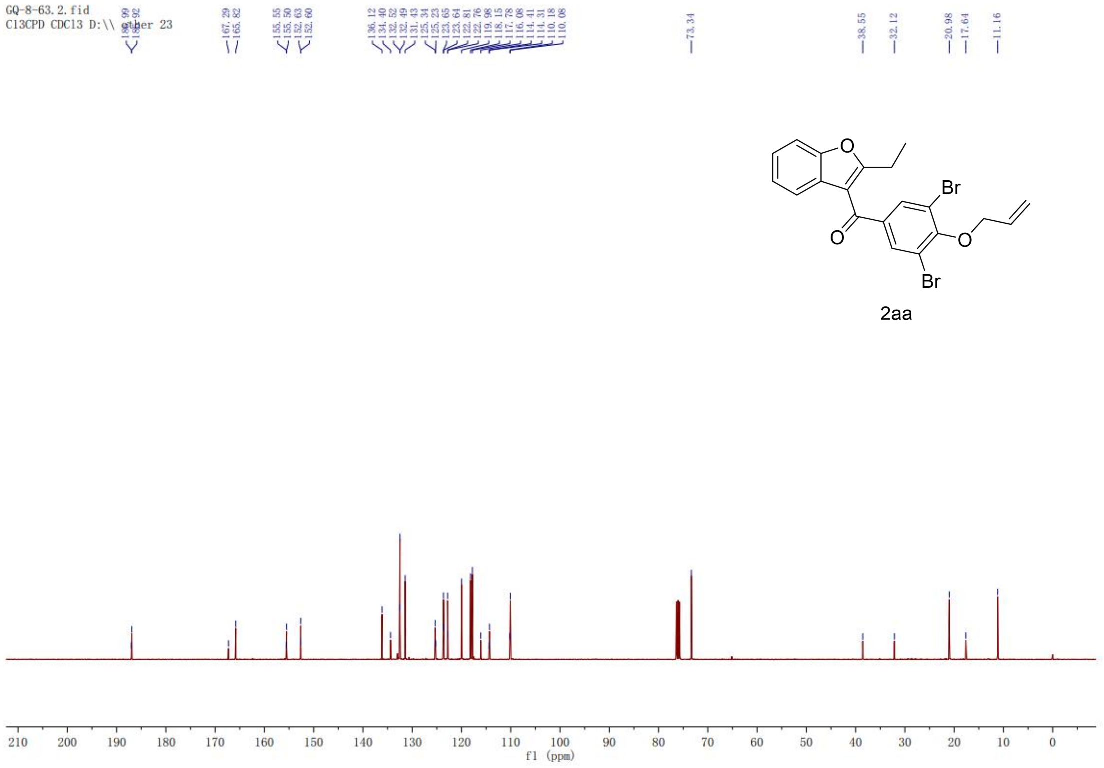
GQ-8-63.2.fid
C13CPD CDCl3 D:\\ other 23
Br
O
Br
O
O
2aa
f1 (ppm)

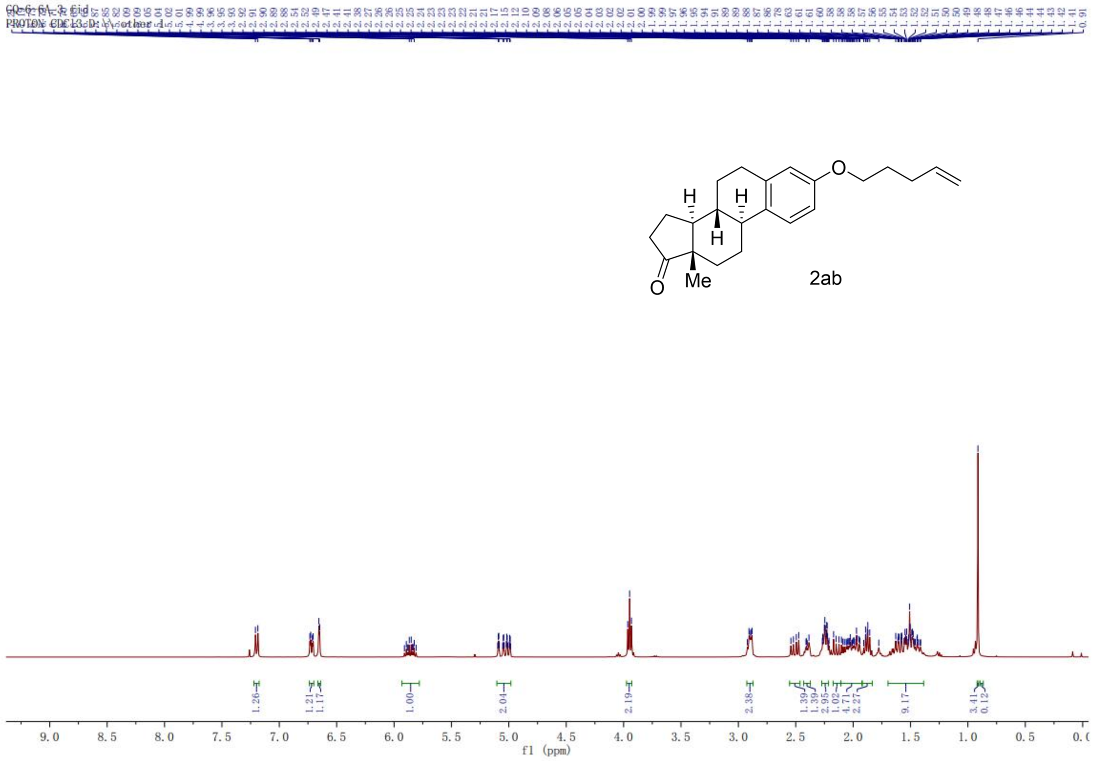
2ab
1.26
1.21
1.17
1.00
2.04
2.19
2.38
1.39
1.39
2.95
1.02
4.71
2.27
9.17
3.41
0.12
f1 (ppm)

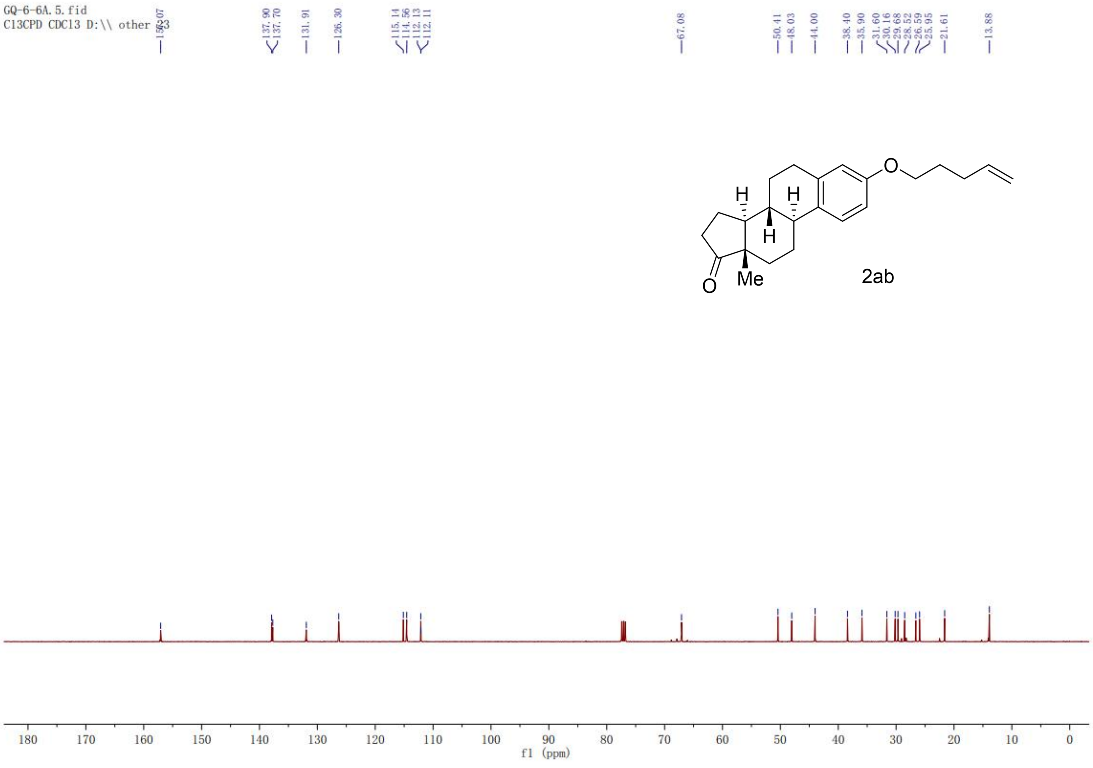
GQ-6-6A.5.fid
C13CPD CDCl3 D:\\ other 13
137.90
137.70
131.91
126.30
115.14
114.56
112.13
112.11
67.08
50.41
48.03
44.00
38.40
35.90
31.60
30.16
29.68
28.52
26.59
25.95
21.61
13.88
2ab
f1 (ppm)

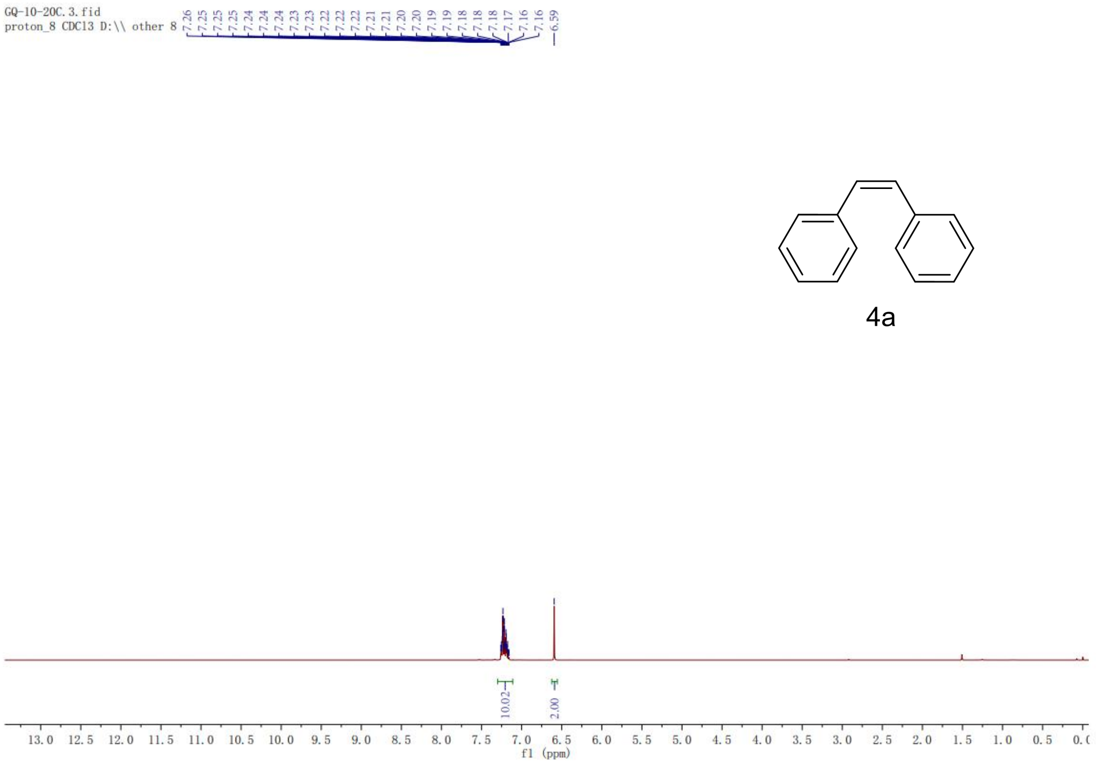
GQ-10-20C.3.fid
proton_8 CDCl3 D:\\ other 8
7.26 7.25 7.25 7.25 7.24 7.24 7.24 7.23 7.23 7.22 7.22 7.22 7.21 7.21 7.20 7.20 7.19 7.19 7.18 7.18 7.18 7.17 7.16 7.16
6.59
4a
10.02
2.00
13.0 12.5 12.0 11.5 11.0 10.5 10.0 9.5 9.0 8.5 8.0 7.5 7.0 6.5 6.0 5.5 5.0 4.5 4.0 3.5 3.0 2.5 2.0 1.5 1.0 0.5
f1 (ppm)

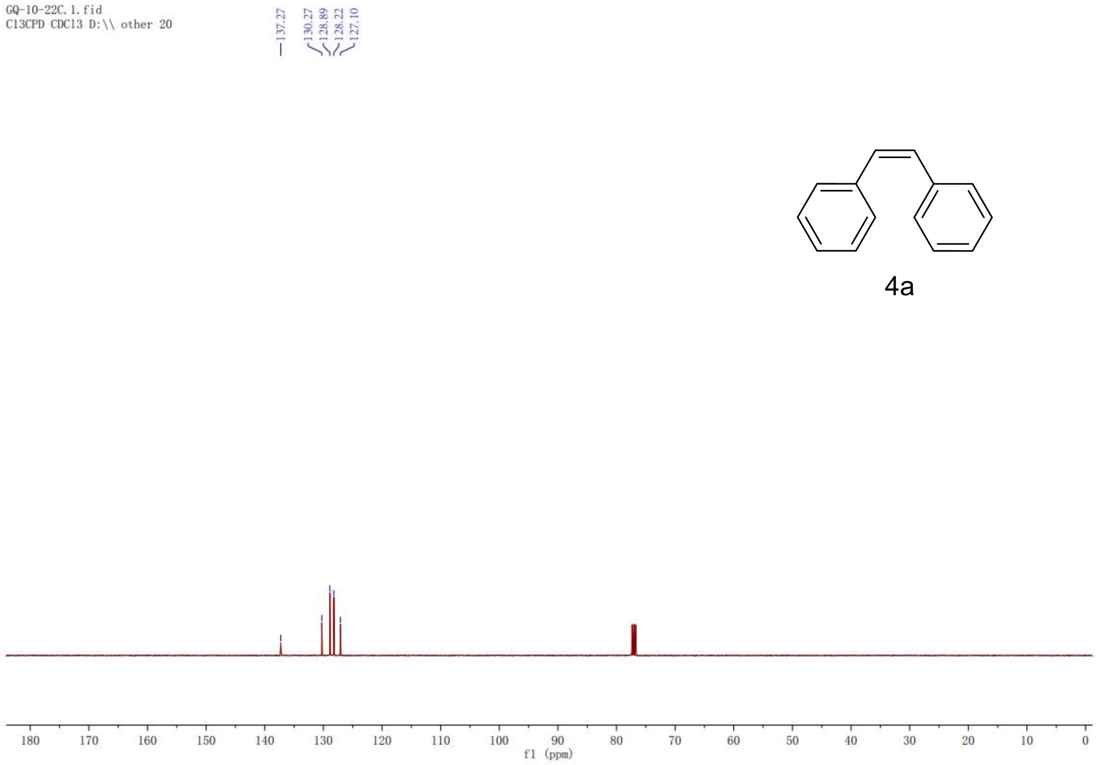
GQ-10-22C.1.fid
C13CPD CDCl3 D:\\ other 20
137.27
130.27
128.89
128.22
127.10
4a
180 170 160 150 140 130 120 110 100 90 80 70 60 50 40 30 20 10 0
f1 (ppm)

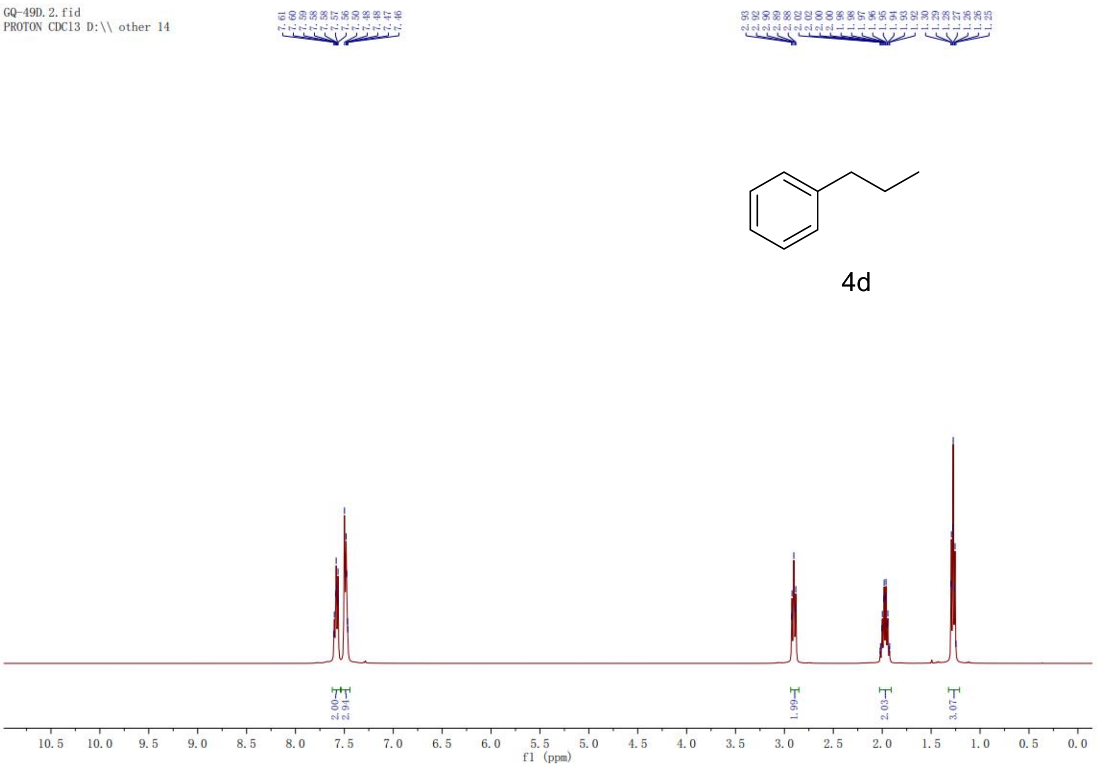
GQ-49D.2.fid
PROTON CDCl3 D:\\ other 14
4d
2.00
2.94
1.99
2.03
3.07
f1 (ppm)

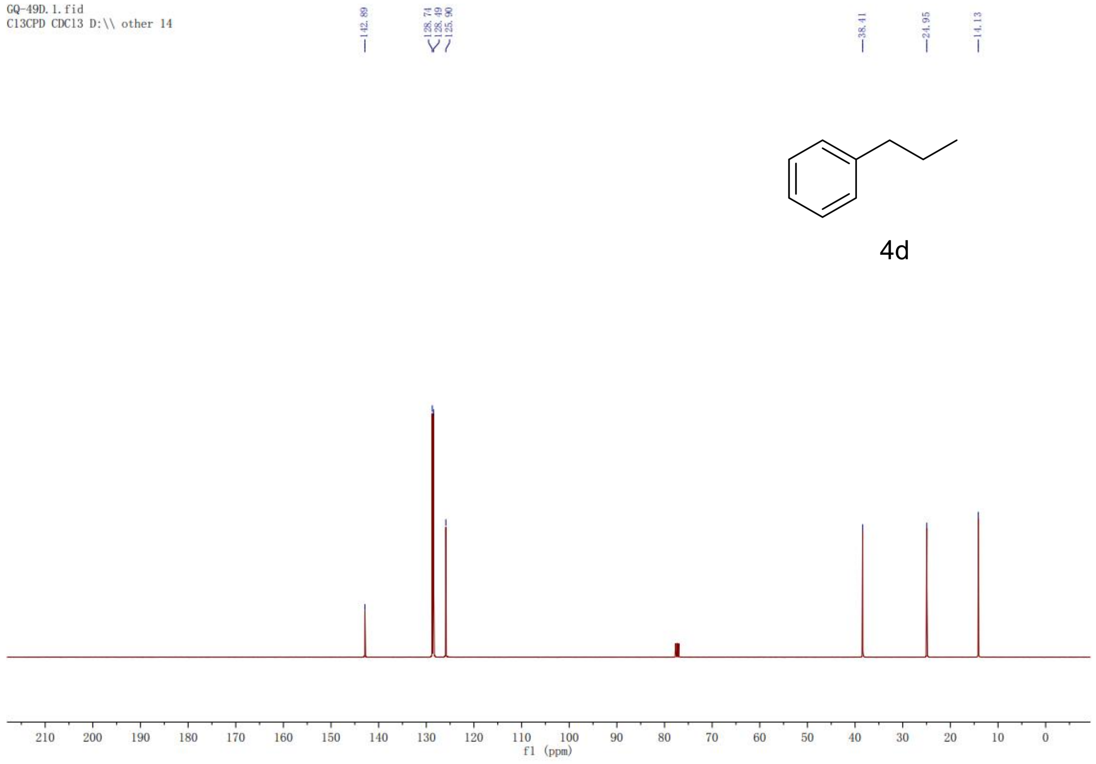
GQ-49D.1.fid
C13CPD CDCl3 D:\\ other 14
142.89
128.74
128.49
125.90
38.41
24.95
14.13
4d
f1 (ppm)

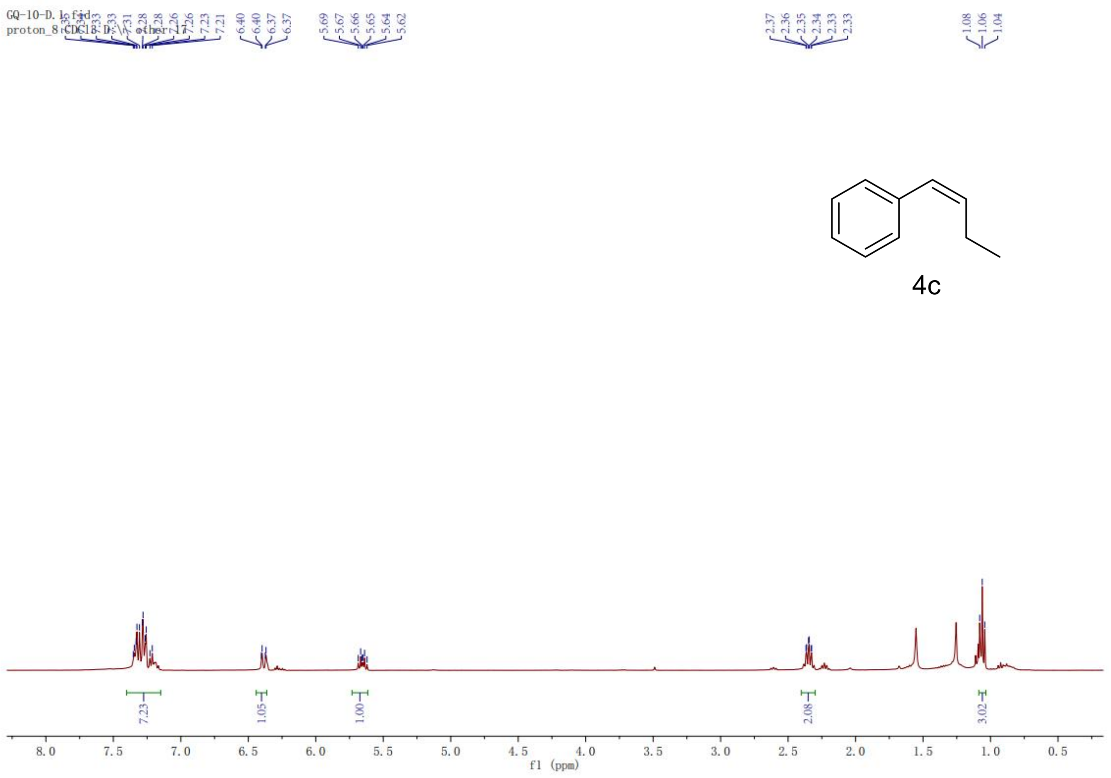
GQ-10-D.1.fid
proton_8 CDCl3 D:\\ other 17
7.31 7.28 7.28 7.26 7.26 7.23 7.21
6.40 6.40 6.37 6.37
5.69 5.67 5.66 5.65 5.64 5.62
2.37 2.36 2.35 2.34 2.33 2.33
1.08 1.06 1.04
7.23 1.05 1.00 2.08 3.02
4c
8.0 7.5 7.0 6.5 6.0 5.5 5.0 4.5 4.0 3.5 3.0 2.5 2.0 1.5 1.0 0.5
f1 (ppm)

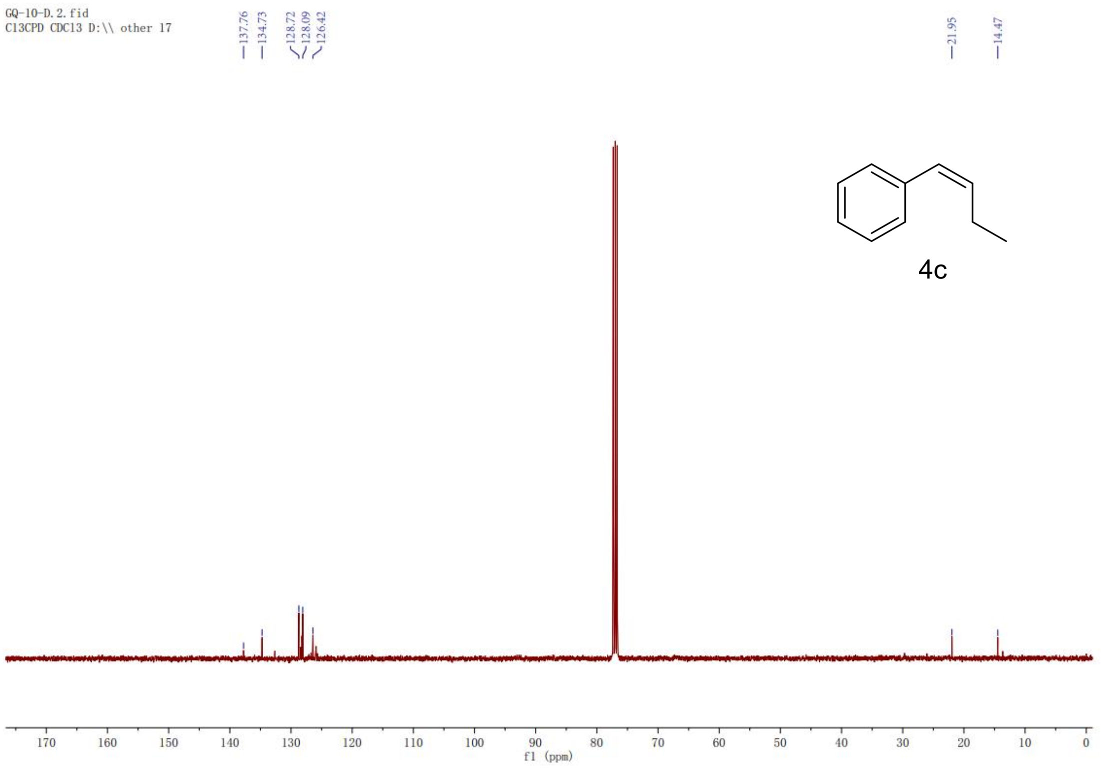
GQ-10-D.2.fid
C13CPD CDCl3 D:\\ other 17
137.76 134.73 128.72 128.09 126.42
21.95 14.47
4c
170 160 150 140 130 120 110 100 90 80 70 60 50 40 30 20 10 0
f1 (ppm)

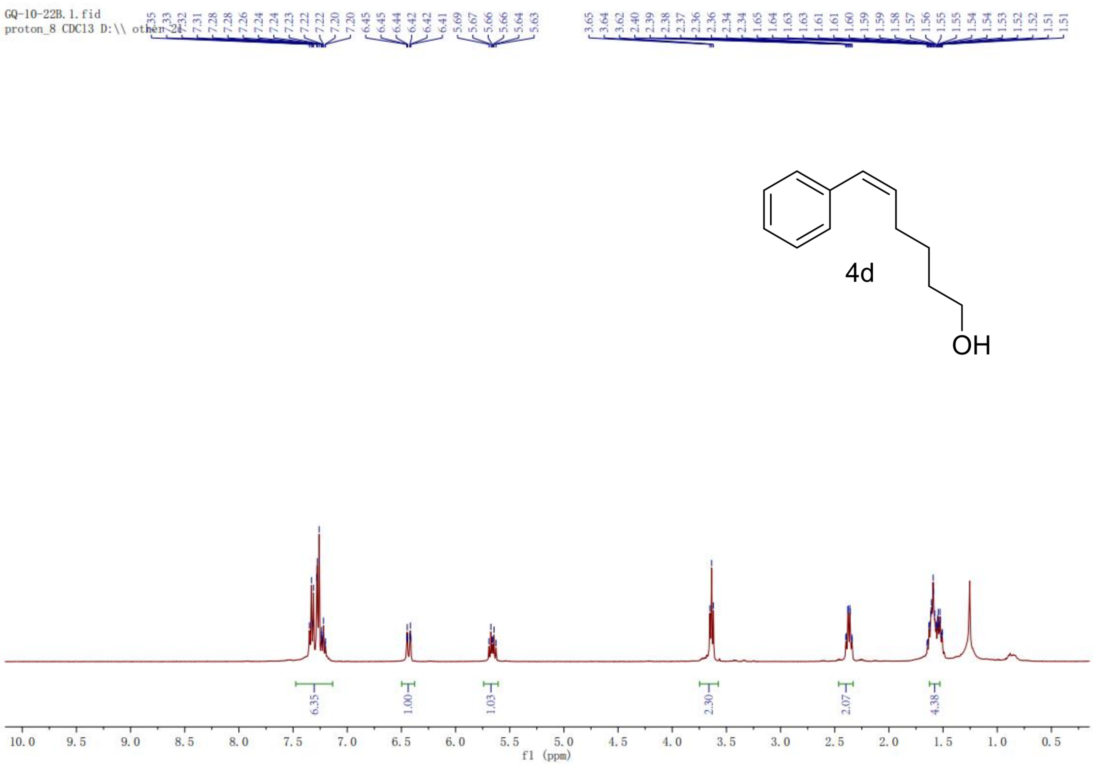
GQ-10-22B.1.fid
proton_8 CDCl3 D:\\ other 24
4d
OH
f1 (ppm)

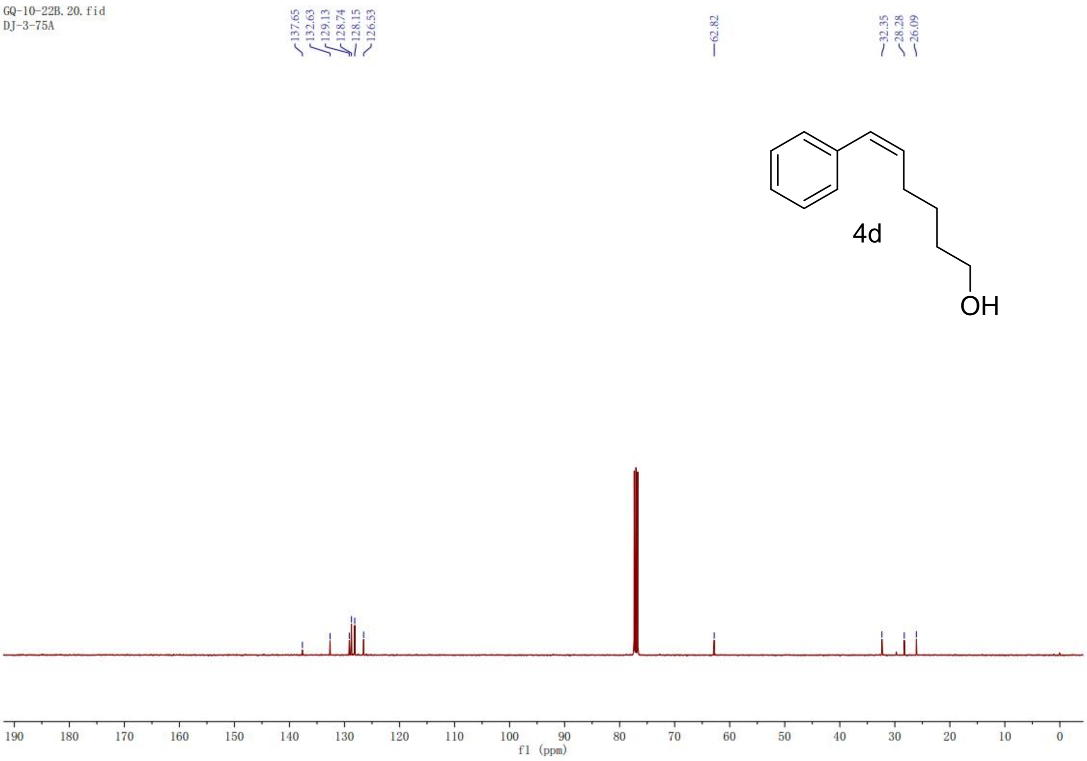
GQ-10-22B.20.fid
DJ-3-75A
137.65
132.63
129.13
128.74
128.15
126.53
62.82
32.35
28.28
26.09
4d
OH
f1 (ppm)

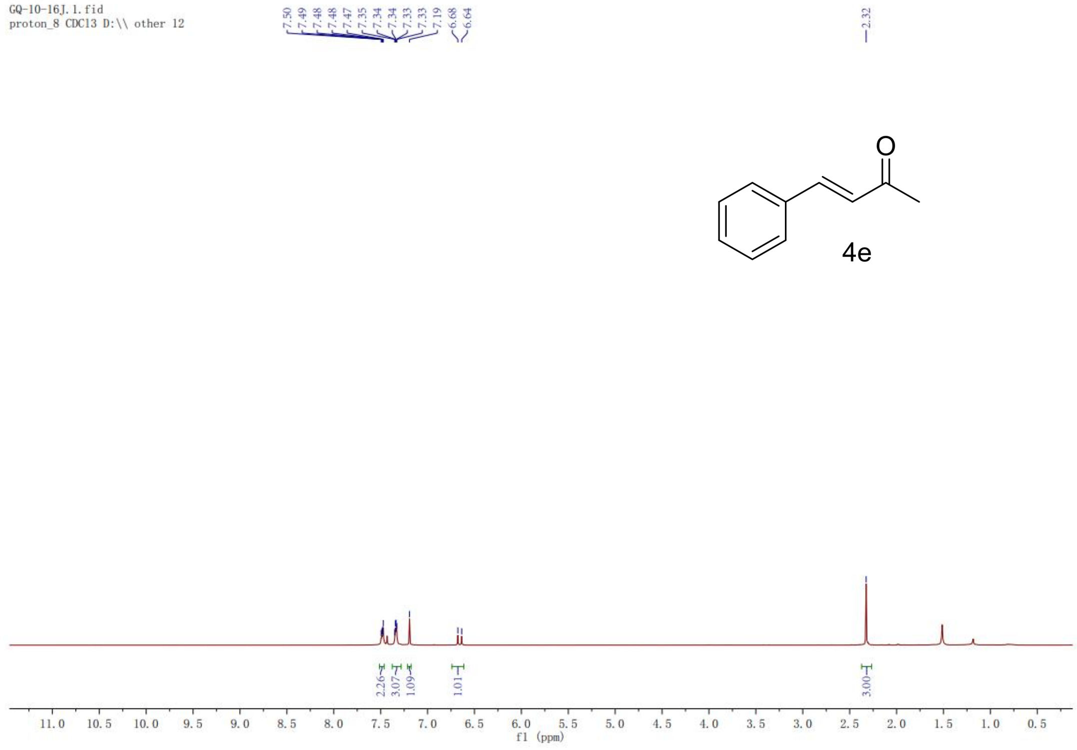
GQ-10-16J.1.fid
proton_8 CDCl3 D:\\ other 12
7.50
7.49
7.48
7.48
7.47
7.35
7.34
7.34
7.33
7.33
7.19
6.68
6.64
2.32
O
4e
2.26
3.07
1.09
1.01
3.00
11.0
10.5
10.0
9.5
9.0
8.5
8.0
7.5
7.0
6.5
6.0
5.5
5.0
4.5
4.0
3.5
3.0
2.5
2.0
1.5
1.0
0.5
f1 (ppm)

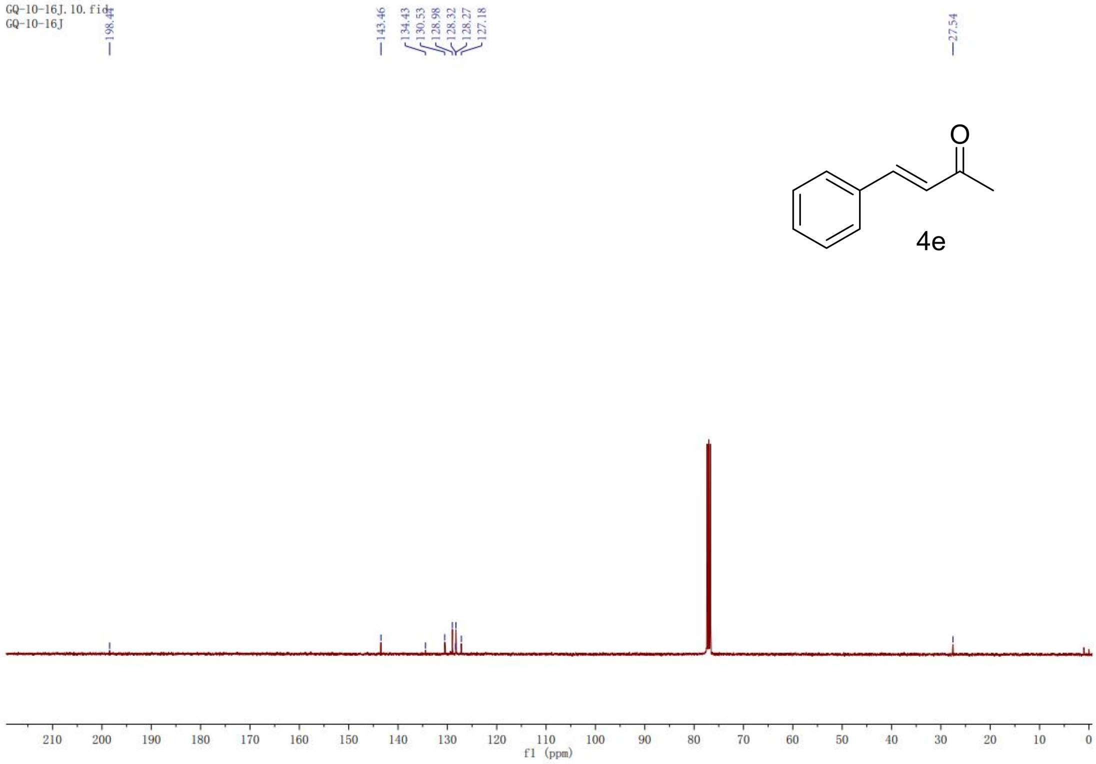
GQ-10-16J.10.fid
GQ-10-16J
198.44
143.46
134.43
130.53
128.98
128.32
128.27
127.18
27.54
O
4e
210
200
190
180
170
160
150
140
130
120
110
100
90
80
70
60
50
40
30
20
10
0
f1 (ppm)

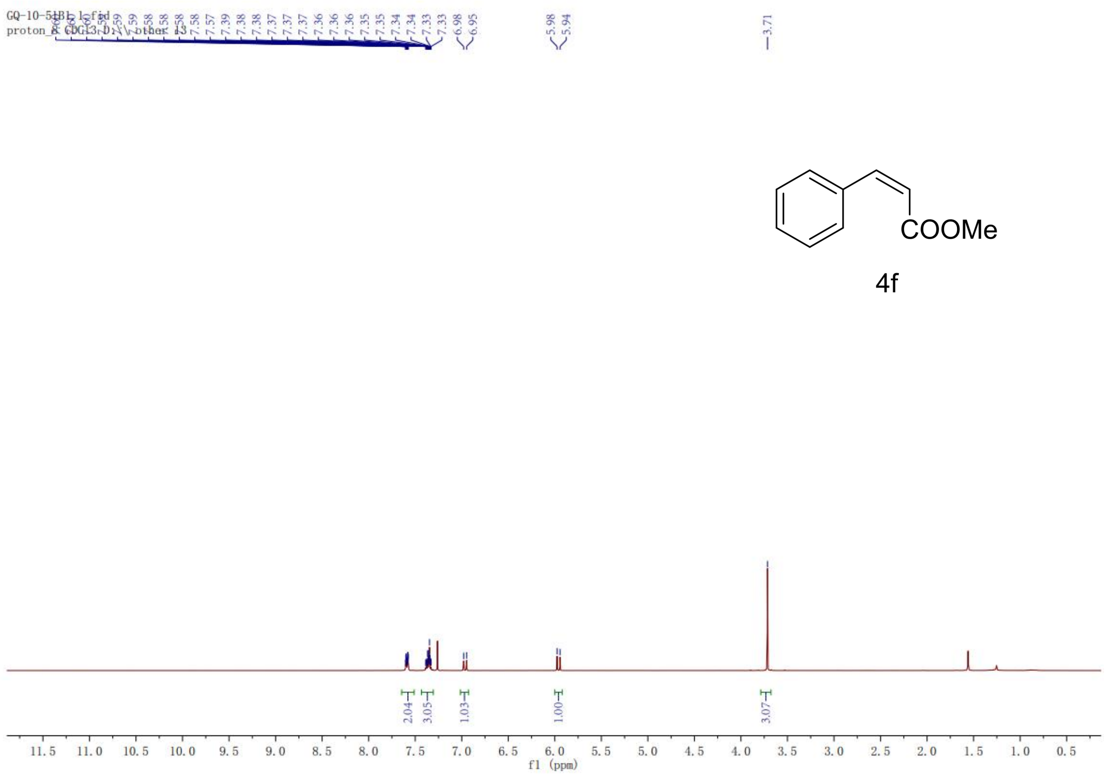
GQ-10-51B1.1.fid
proton_& CDCl3_D:\\ other 13
3.71
5.98
5.94
6.98
6.95
COOMe
4f
2.04
3.05
1.03
1.00
3.07
f1 (ppm)

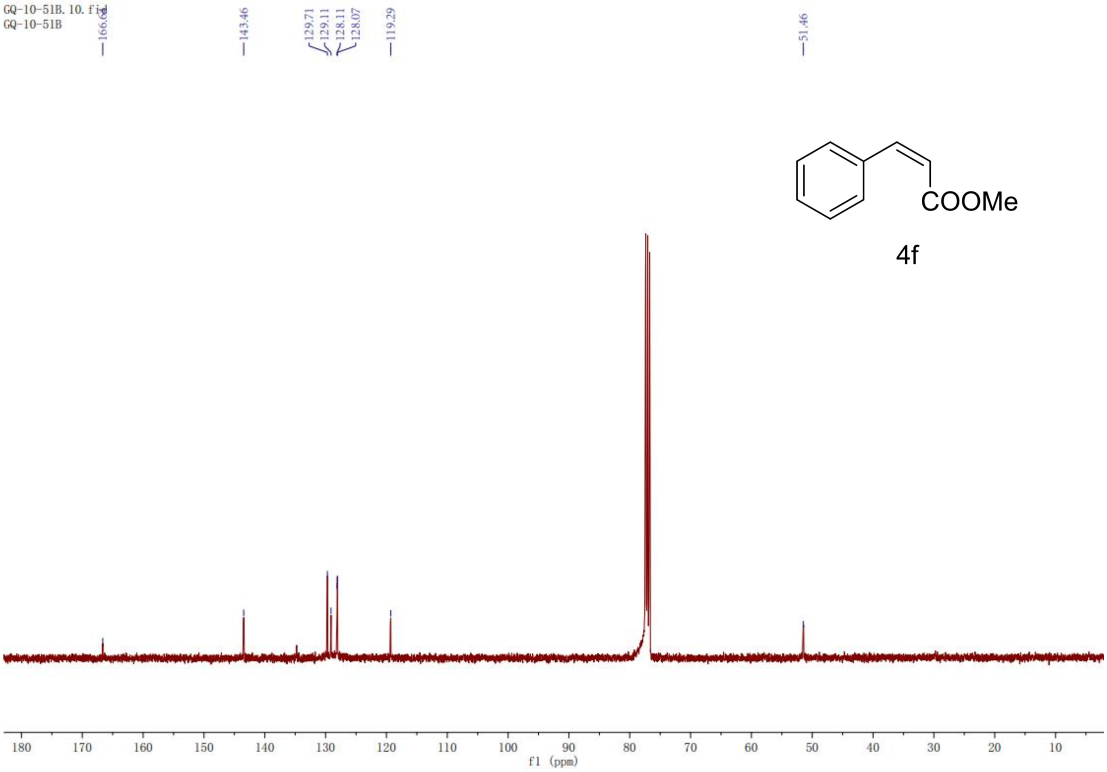
GQ-10-51B.10.fid
GQ-10-51B
166.6
143.46
129.71
129.11
128.11
128.07
119.29
51.46
COOMe
4f
f1 (ppm)

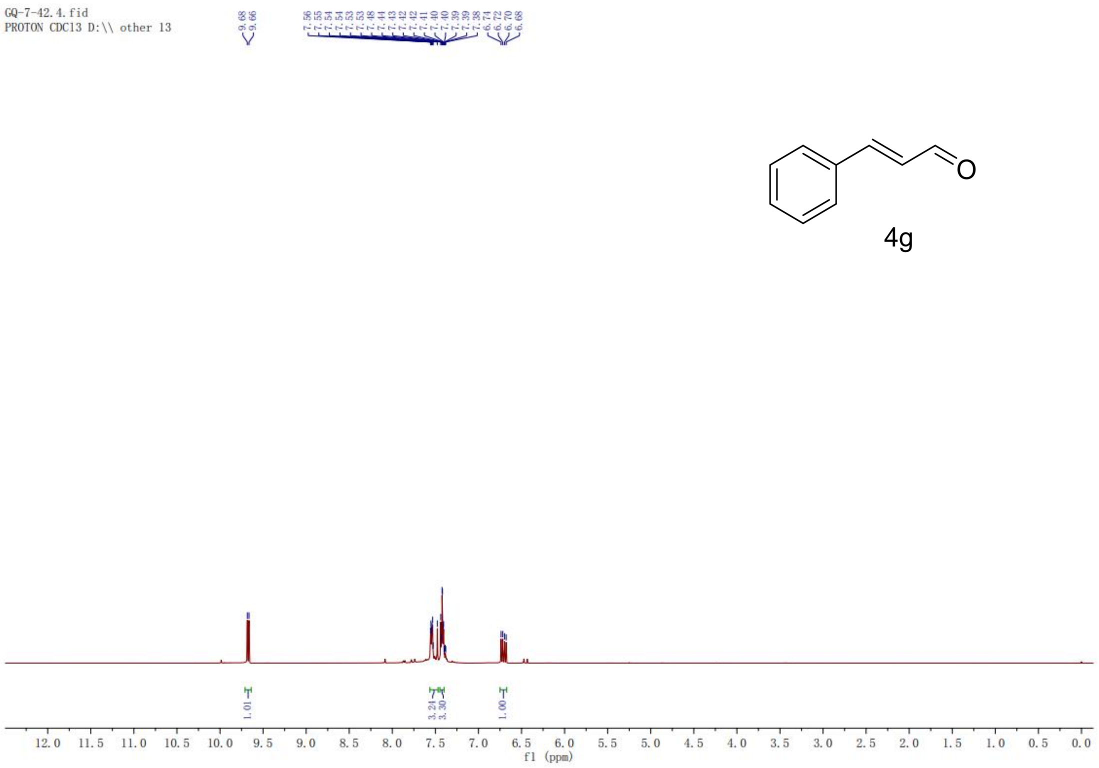
GQ-7-42.4.fid
PROTON CDCl3 D:\\ other 13
9.68
9.66
7.56
7.55
7.54
7.54
7.53
7.53
7.48
7.44
7.43
7.42
7.42
7.41
7.40
7.40
7.39
7.39
7.38
6.74
6.72
6.70
6.68
O
4g
1.01
3.24
3.30
1.00
12.0 11.5 11.0 10.5 10.0 9.5 9.0 8.5 8.0 7.5 7.0 6.5 6.0 5.5 5.0 4.5 4.0 3.5 3.0 2.5 2.0 1.5 1.0 0.5 0.0
f1 (ppm)

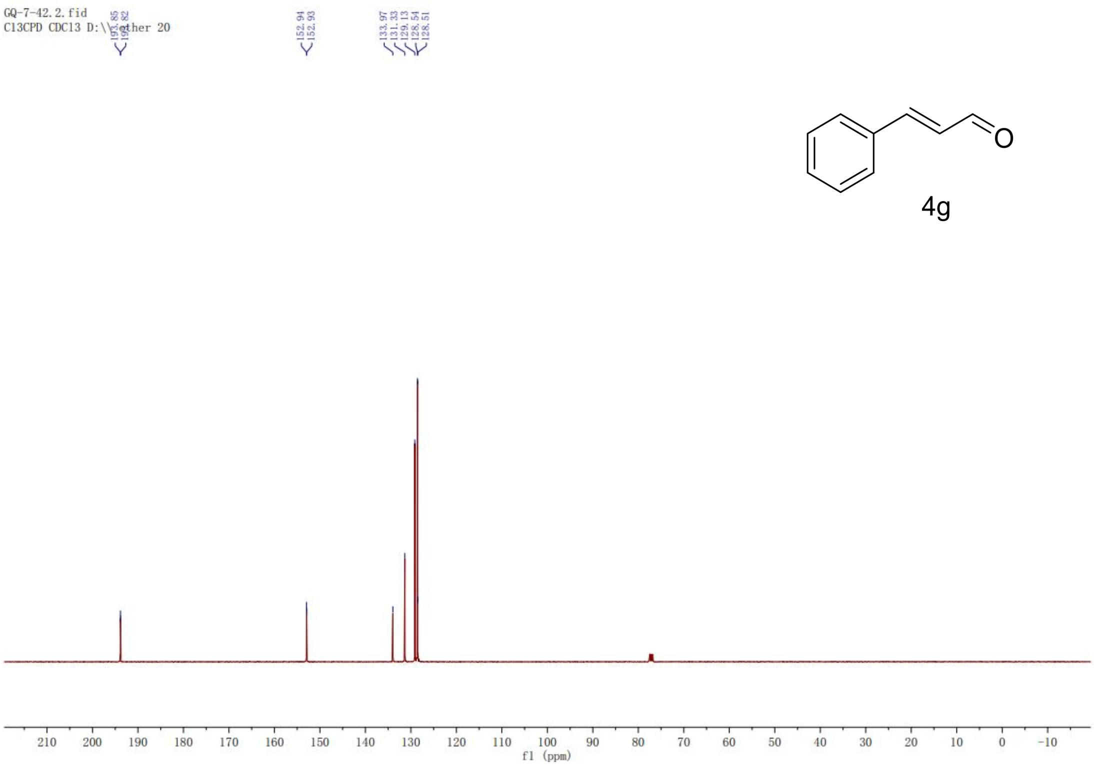
GQ-7-42.2.fid
C13CPD CDCl3 D:\\ other 20
193.85
193.82
152.94
152.93
133.97
131.33
129.13
128.54
128.51
O
4g
210 200 190 180 170 160 150 140 130 120 110 100 90 80 70 60 50 40 30 20 10 0 -10
f1 (ppm)

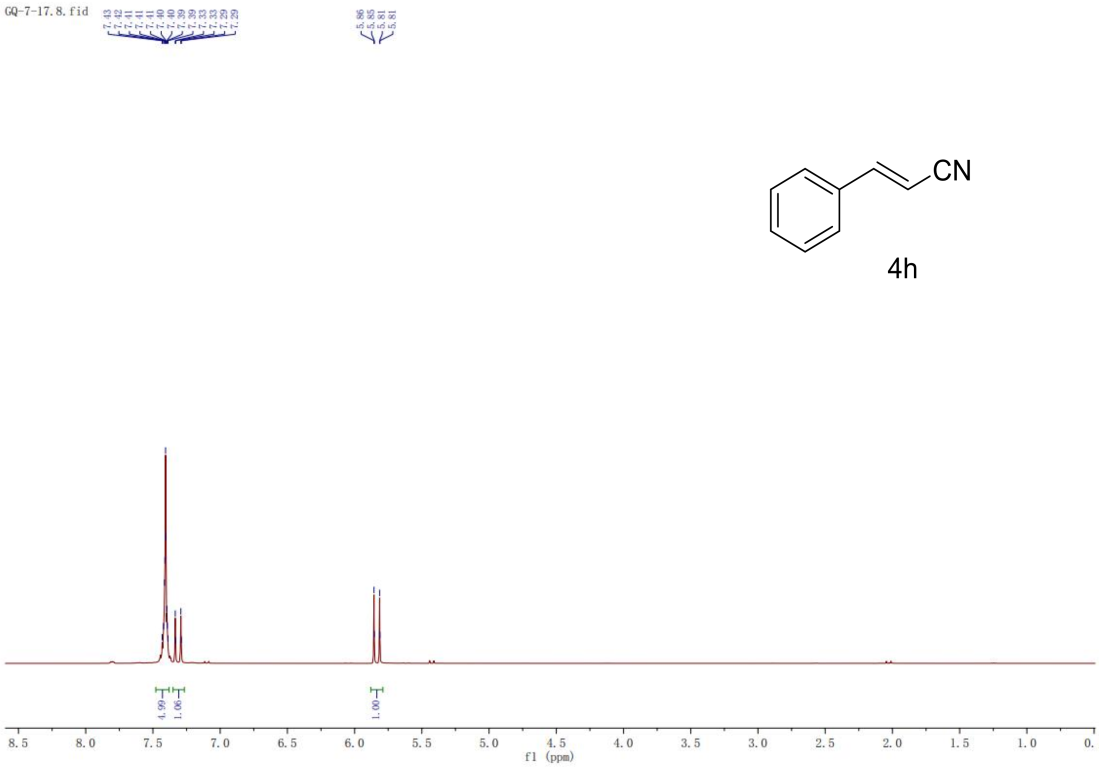
GQ-7-17.8.fid
7.43 7.42 7.41 7.41 7.41 7.40 7.40 7.39 7.39 7.33 7.33 7.29 7.29
5.86 5.85 5.81 5.81
CN
4.99 1.06 1.00
8.5 8.0 7.5 7.0 6.5 6.0 5.5 5.0 4.5 4.0 3.5 3.0 2.5 2.0 1.5 1.0 0.
f1 (ppm)

4h

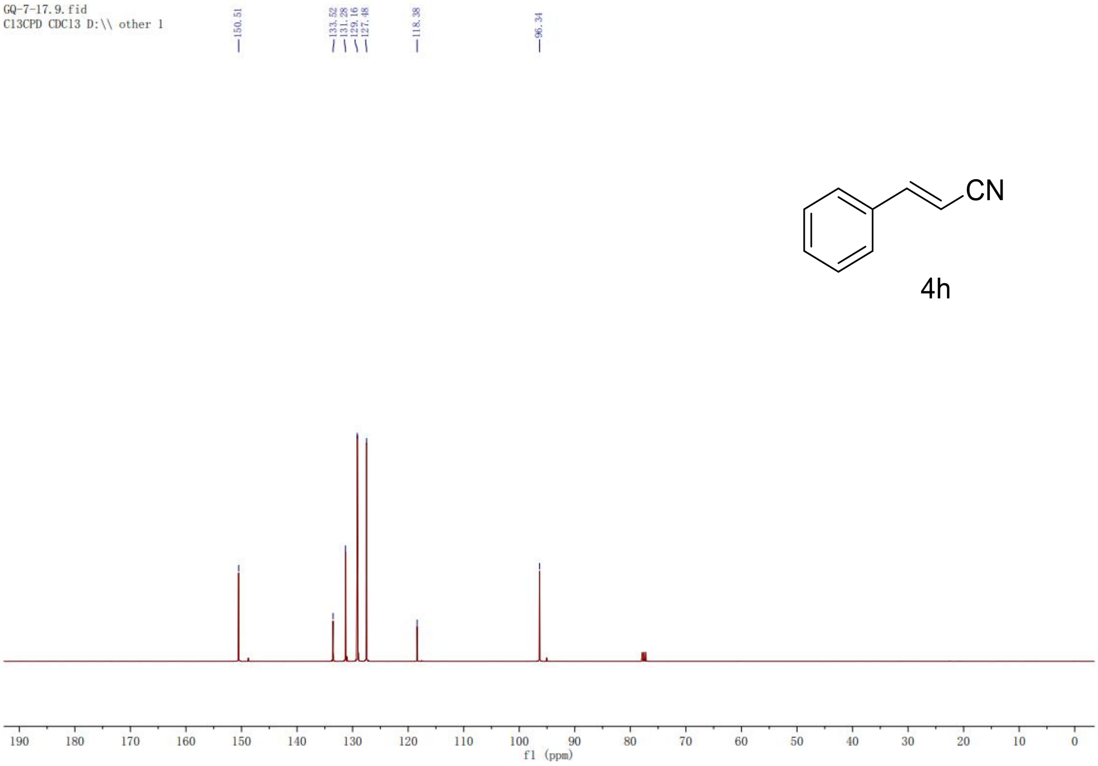
GQ-7-17.9.fid
C13CPD CDCl3 D:\\ other 1
150.51
133.52 131.28 129.16 127.48
118.38
96.34
CN
190 180 170 160 150 140 130 120 110 100 90 80 70 60 50 40 30 20 10 0
f1 (ppm)

4h

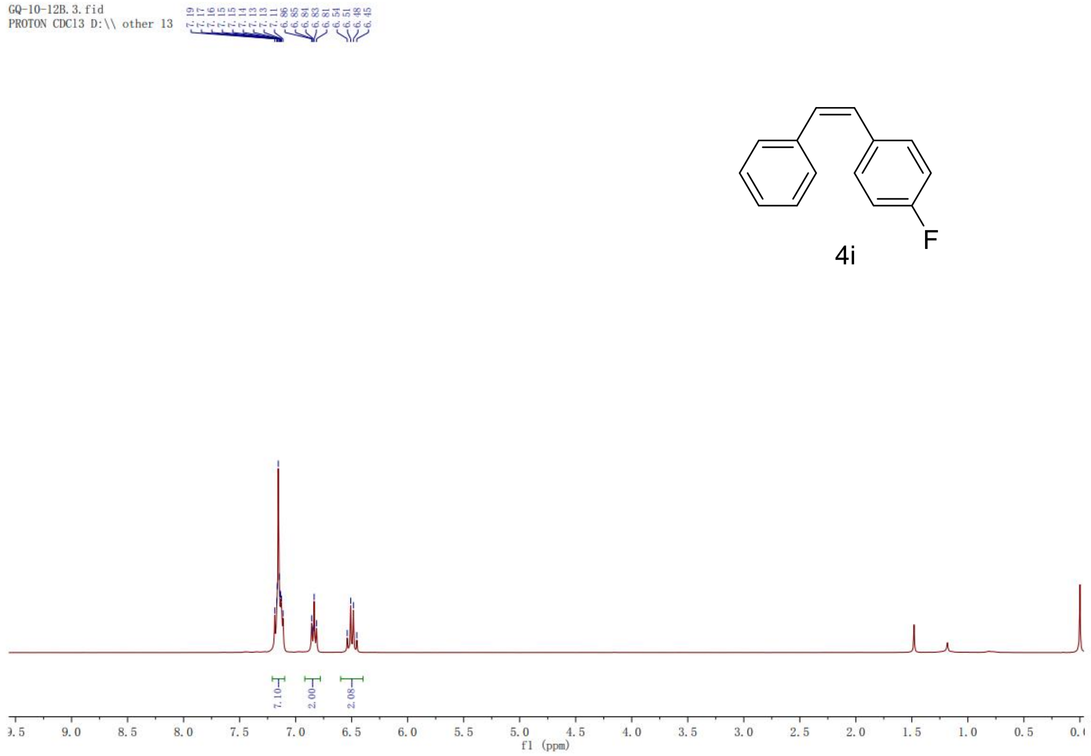
GQ-10-12B.3.fid
PROTON CDCl3 D:\\ other 13
7.19 7.17 7.16 7.15 7.15 7.14 7.13 7.13 7.11 6.86 6.85 6.84 6.83 6.81 6.54 6.51 6.48 6.45
F
4i
7.10
2.00
2.08
f1 (ppm)

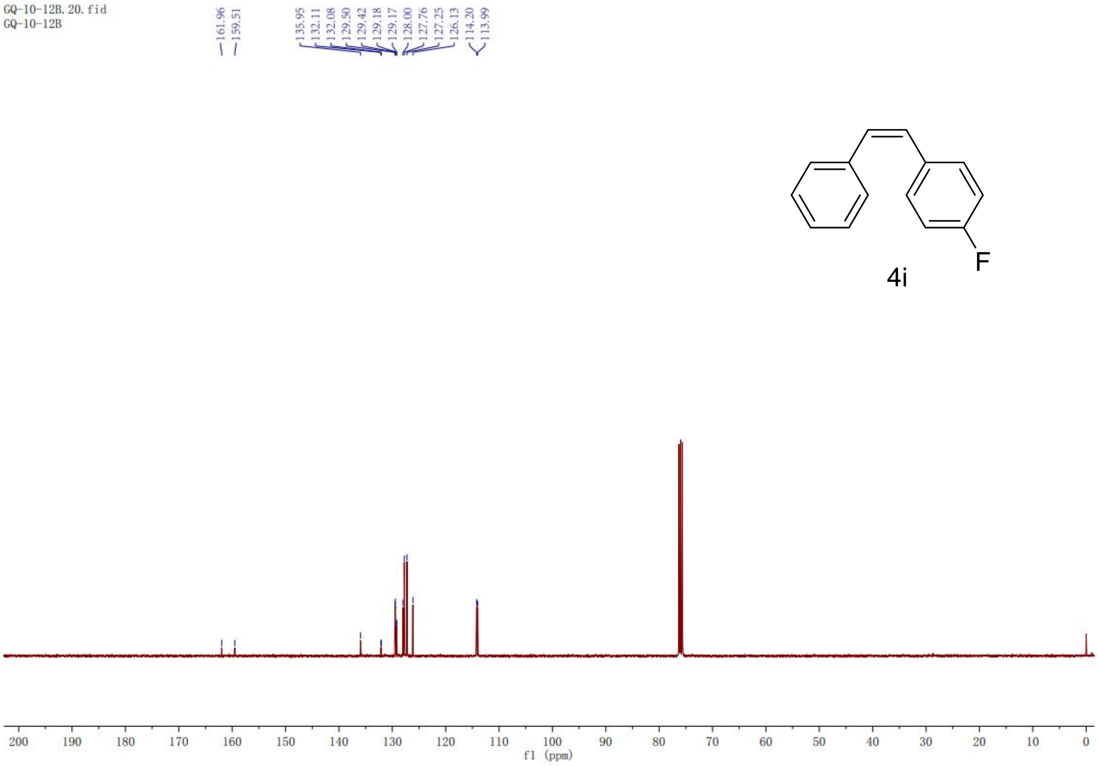
GQ-10-12B.20.fid
GQ-10-12B
161.96 159.51 135.95 132.11 132.08 129.50 129.42 129.18 129.17 128.00 127.76 127.25 126.13 114.20 113.99
F
4i
f1 (ppm)

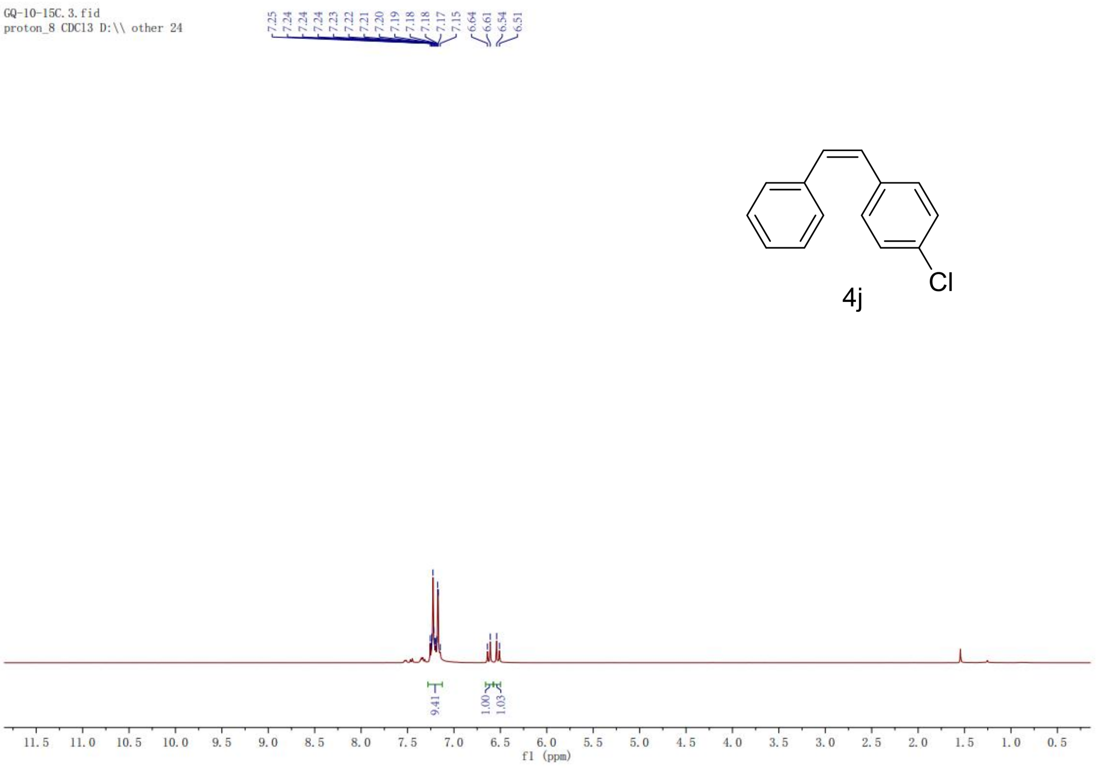
GQ-10-15C.3.fid
proton_8 CDCl3 D:\\ other 24
7.25
7.24
7.24
7.24
7.23
7.22
7.21
7.20
7.19
7.18
7.18
7.17
7.15
6.64
6.61
6.54
6.51
Cl
4j
9.41
1.00
1.03
11.5 11.0 10.5 10.0 9.5 9.0 8.5 8.0 7.5 7.0 6.5 6.0 5.5 5.0 4.5 4.0 3.5 3.0 2.5 2.0 1.5 1.0 0.5
f1 (ppm)

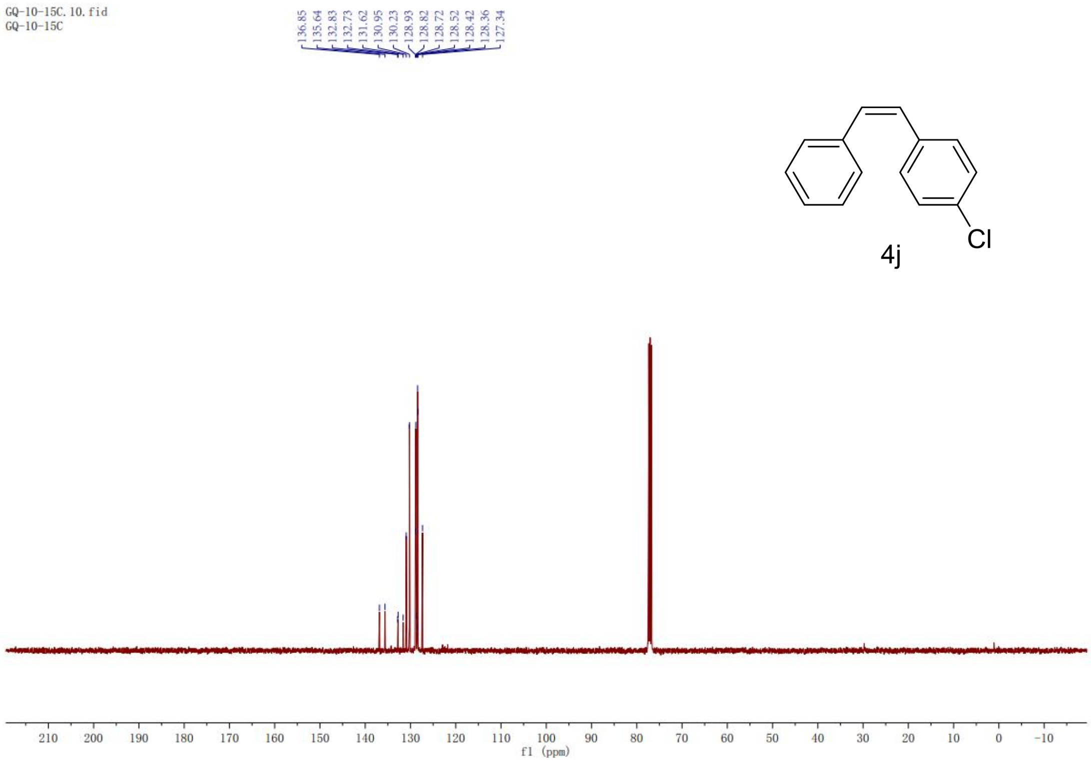
GQ-10-15C.10.fid
GQ-10-15C
136.85
135.64
132.83
132.73
131.62
130.95
130.23
128.93
128.82
128.72
128.52
128.42
128.36
127.34
Cl
4j
210 200 190 180 170 160 150 140 130 120 110 100 90 80 70 60 50 40 30 20 10 0 -10
f1 (ppm)

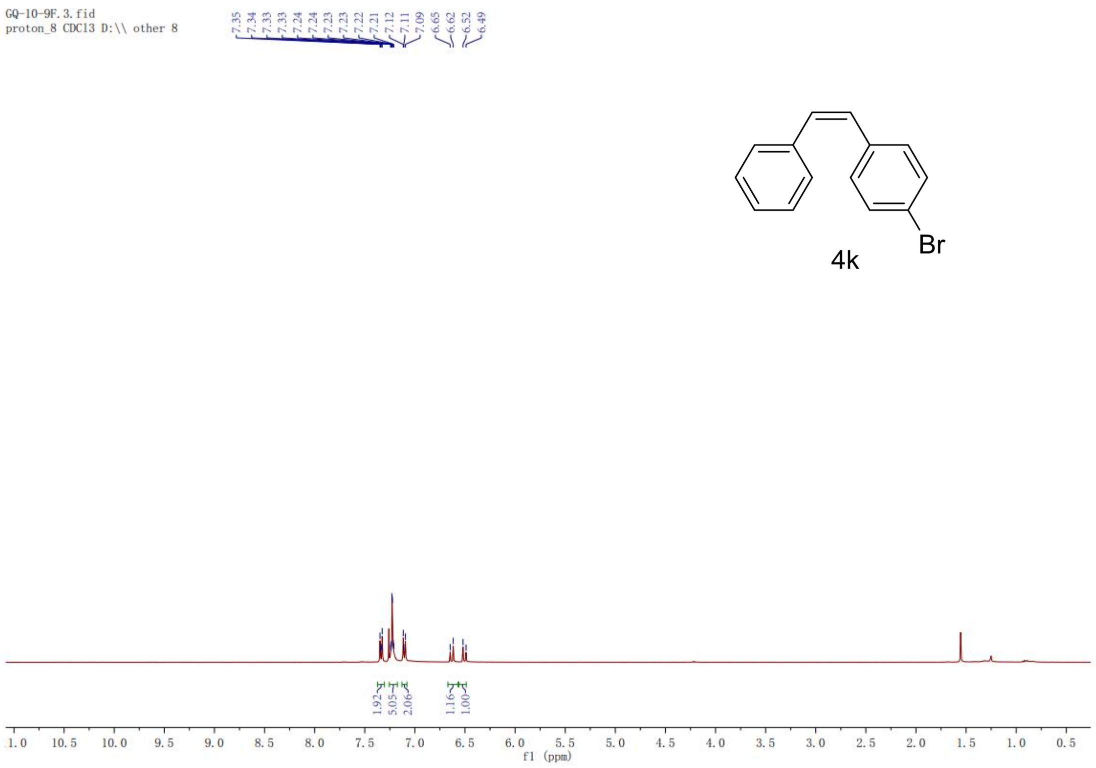
GQ-10-9F.3.fid
proton_8 CDCl3 D:\\ other 8
7.35
7.34
7.33
7.33
7.24
7.24
7.23
7.23
7.22
7.21
7.12
7.11
7.09
6.65
6.62
6.52
6.49
Br
4k
1.92
5.05
2.06
1.16
1.00
f1 (ppm)

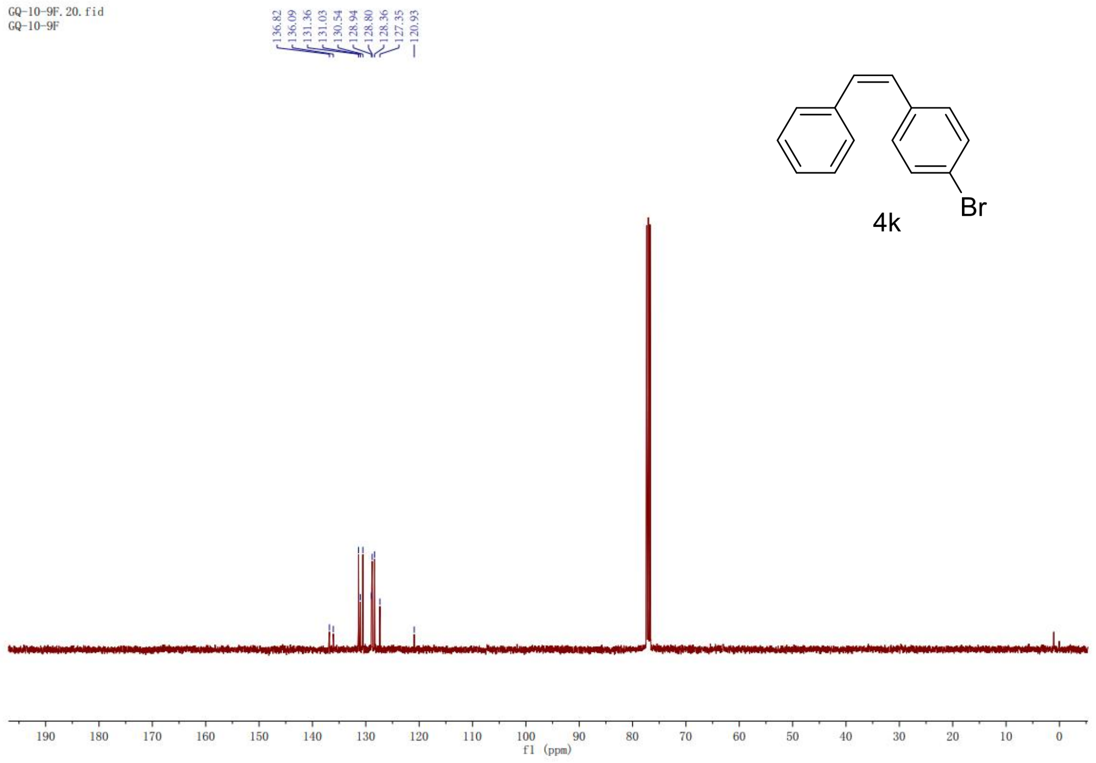
GQ-10-9F.20.fid
GQ-10-9F
136.82
136.09
131.36
131.03
130.54
128.94
128.80
128.36
127.35
120.93
Br
4k
f1 (ppm)

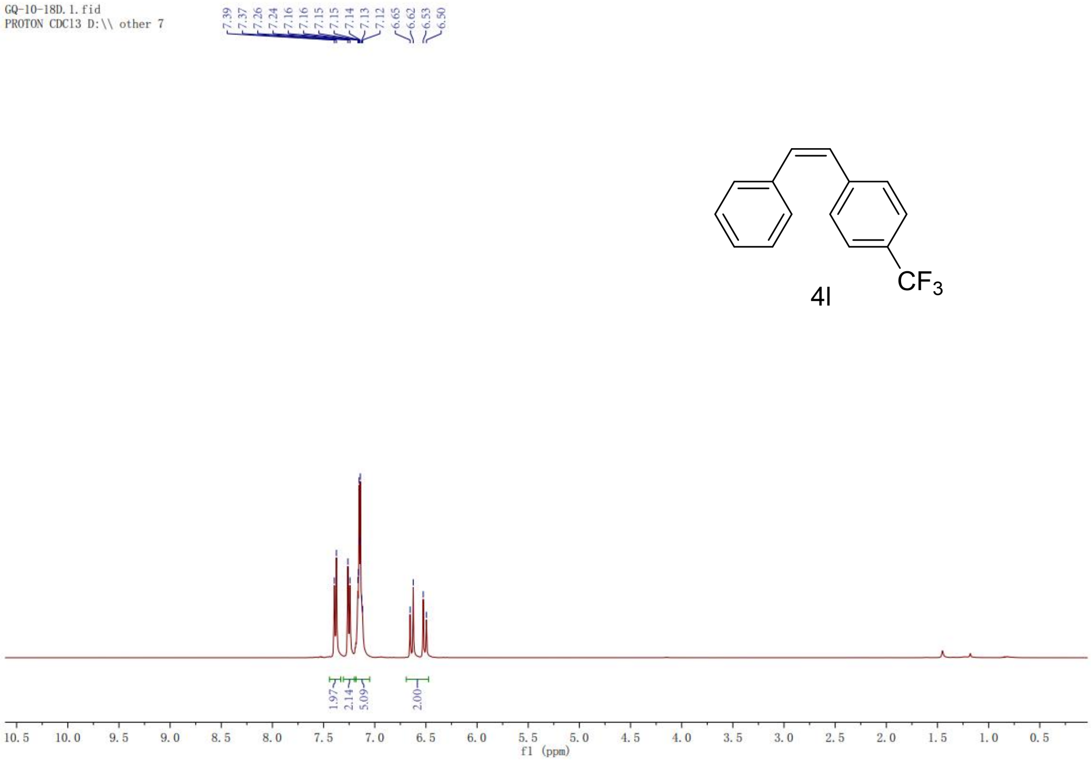
GQ-10-18D.1.fid
PROTON CDCl3 D:\\ other 7
7.39
7.37
7.26
7.24
7.16
7.16
7.15
7.15
7.14
7.13
7.12
6.65
6.62
6.53
6.50
4l
CF3
1.97
2.14
5.09
2.00
10.5 10.0 9.5 9.0 8.5 8.0 7.5 7.0 6.5 6.0 5.5 5.0 4.5 4.0 3.5 3.0 2.5 2.0 1.5 1.0 0.5
f1 (ppm)

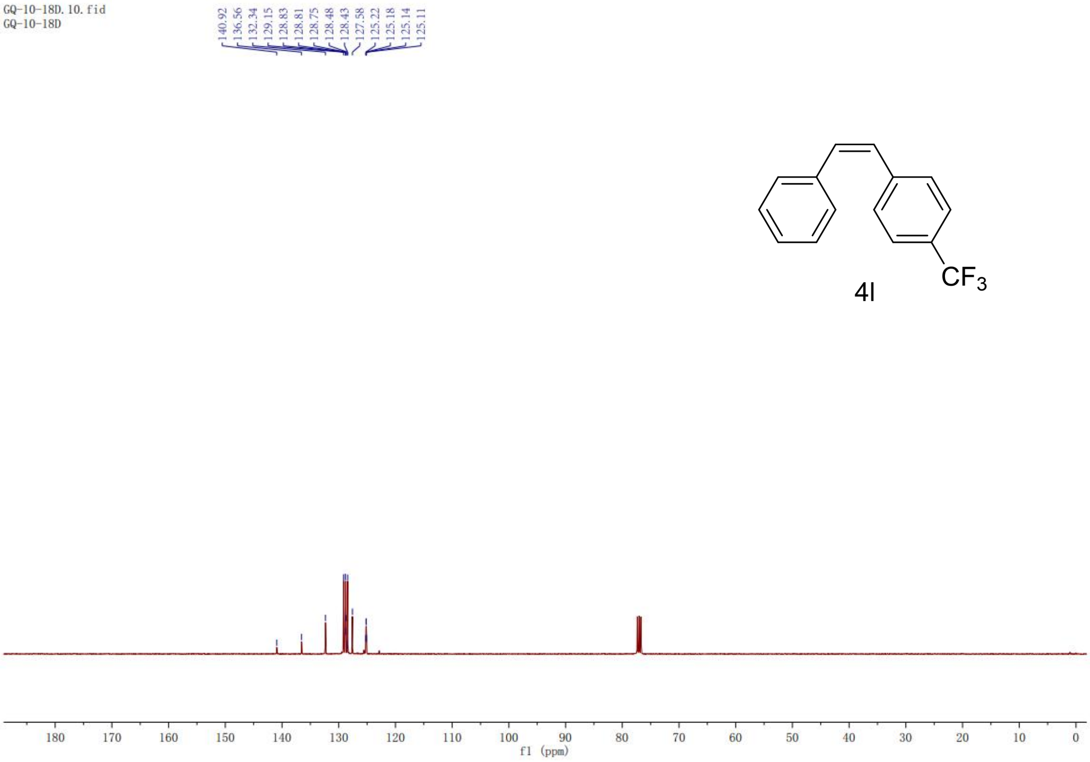
GQ-10-18D.10.fid
GQ-10-18D
140.92
136.56
132.34
129.15
128.83
128.81
128.75
128.48
128.43
127.58
125.22
125.18
125.14
125.11
4l
CF3
180 170 160 150 140 130 120 110 100 90 80 70 60 50 40 30 20 10 0
f1 (ppm)

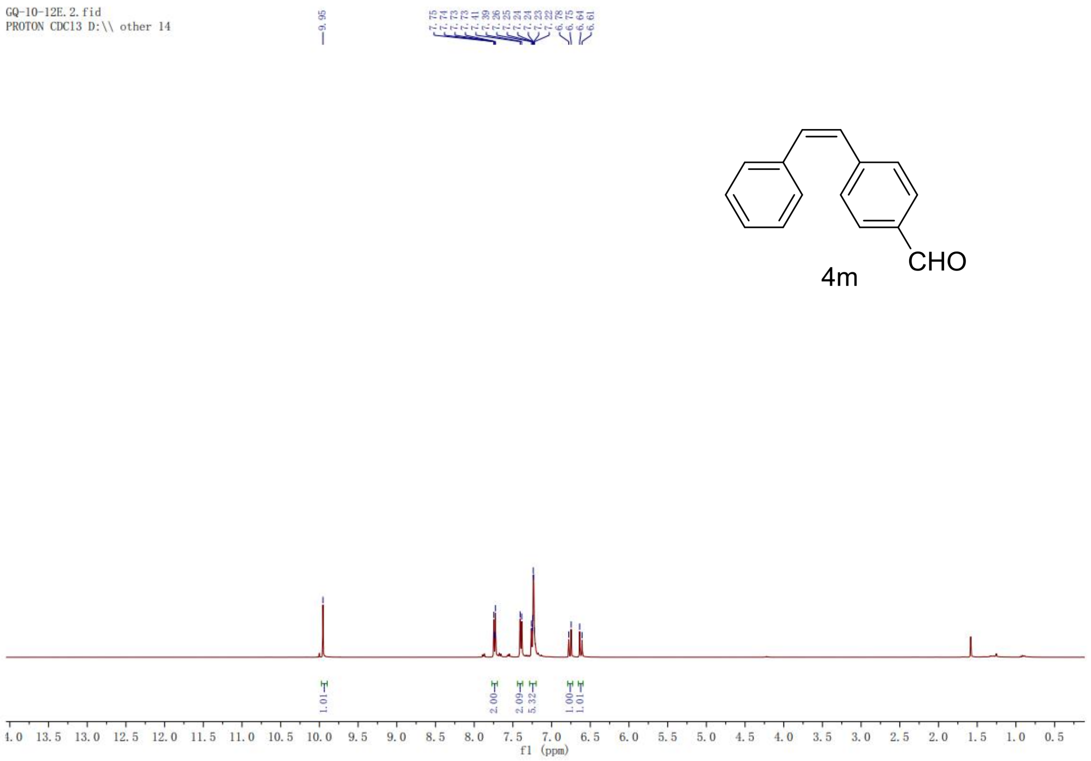
GQ-10-12E.2.fid
PROTON CDCl3 D:\\ other 14
9.95
7.75 7.74 7.73 7.73 7.41 7.39 7.26 7.25 7.24 7.24 7.24 7.23 7.22 6.78 6.75 6.64 6.61
4m
CHO
1.01
2.00 2.09 5.32
1.00 1.01
f1 (ppm)

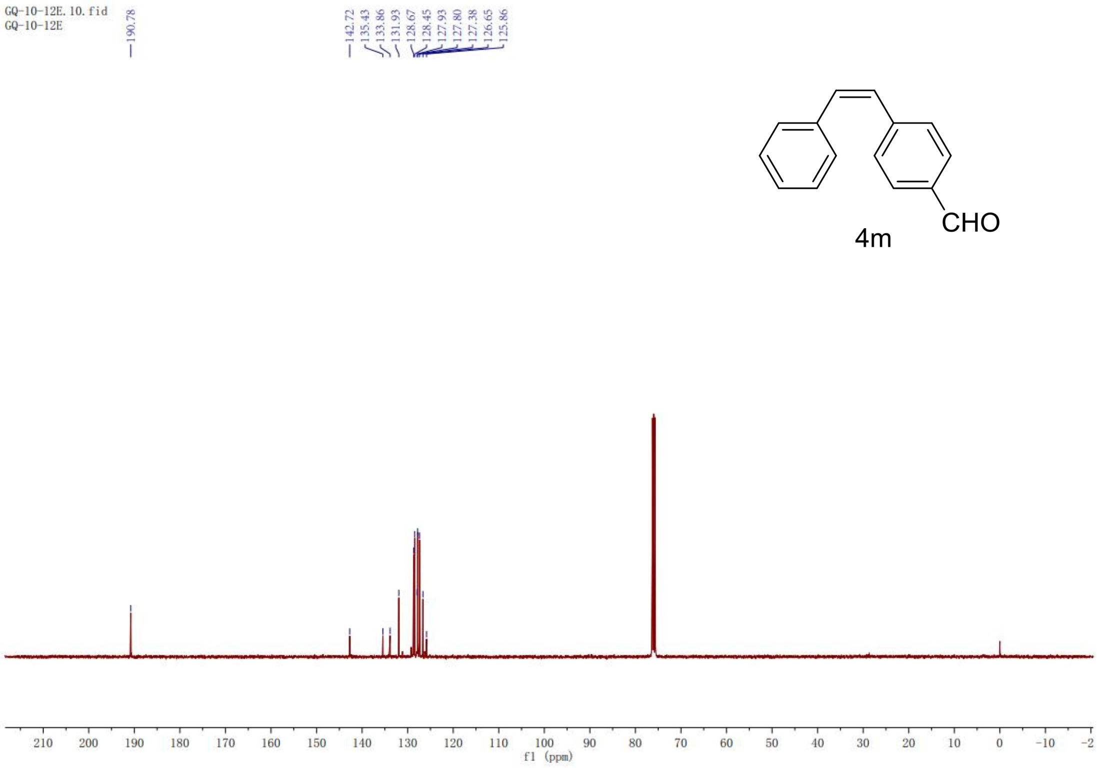
GQ-10-12E.10.fid
GQ-10-12E
190.78
142.72 135.43 133.86 131.93 128.67 128.45 127.93 127.80 127.38 126.65 125.86
4m
CHO
f1 (ppm)

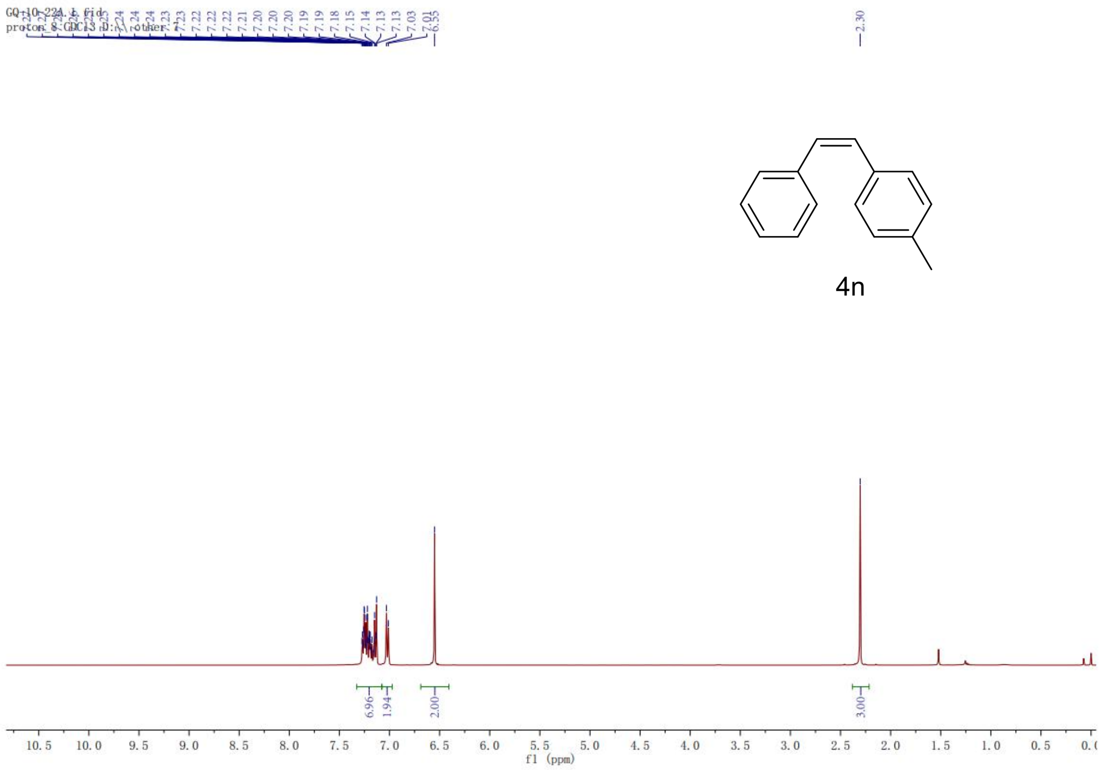
GQ-10-22A.1.fid
proton_8 CDCl3 D:\\ other 7
7.24
7.24
7.24
7.23
7.23
7.22
7.22
7.22
7.21
7.20
7.20
7.20
7.19
7.19
7.18
7.15
7.14
7.13
7.13
7.03
7.01
6.55
2.30
4n
6.96
1.94
2.00
3.00
f1 (ppm)

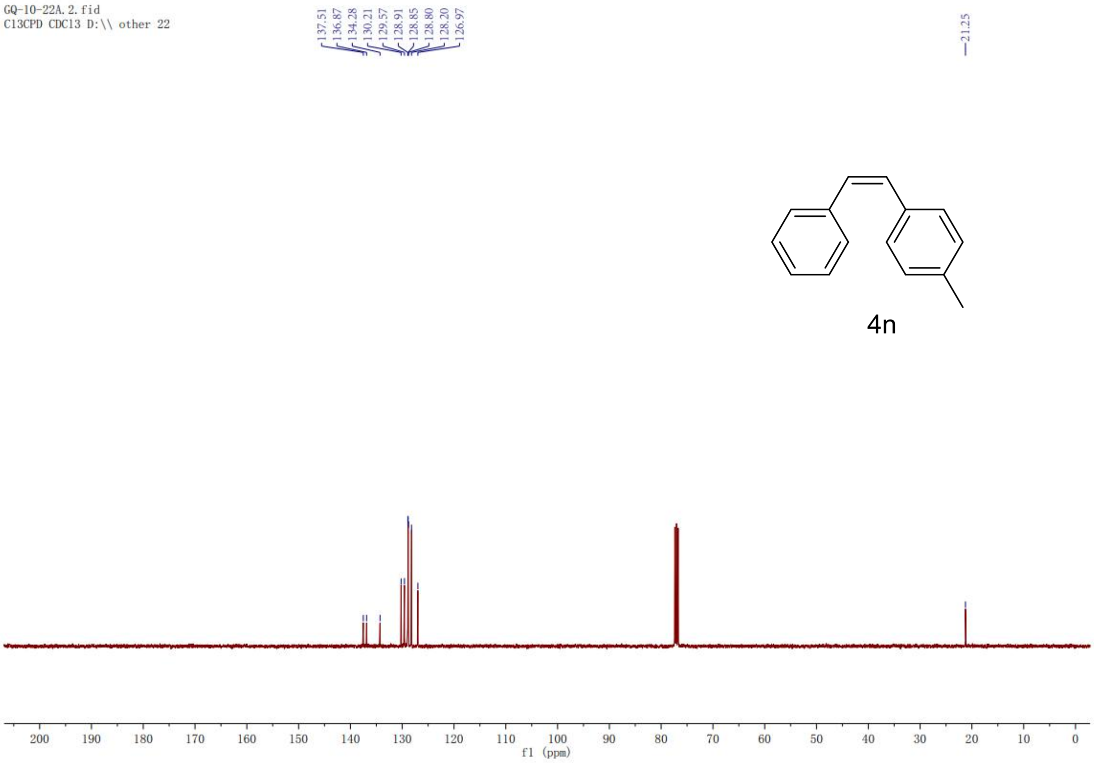
GQ-10-22A.2.fid
C13CPD CDCl3 D:\\ other 22
137.51
136.87
134.28
130.21
129.57
128.91
128.85
128.80
128.20
126.97
21.25
4n
f1 (ppm)

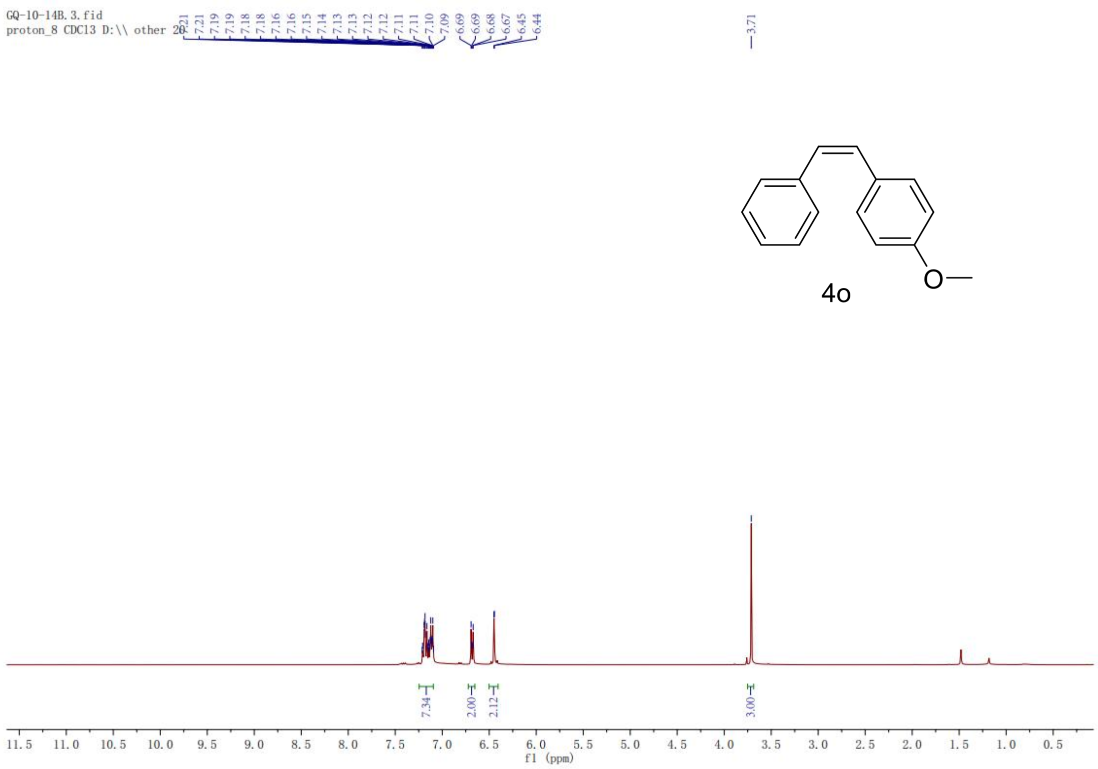
GQ-10-14B.3.fid
proton_8 CDCl3 D:\\ other 20
7.21 7.21 7.19 7.19 7.18 7.18 7.16 7.16 7.15 7.14 7.13 7.13 7.12 7.12 7.11 7.11 7.10 7.09 6.69 6.69 6.68 6.67 6.45 6.44
3.71
4o
7.34
2.00
2.12
3.00
11.5 11.0 10.5 10.0 9.5 9.0 8.5 8.0 7.5 7.0 6.5 6.0 5.5 5.0 4.5 4.0 3.5 3.0 2.5 2.0 1.5 1.0 0.5
f1 (ppm)

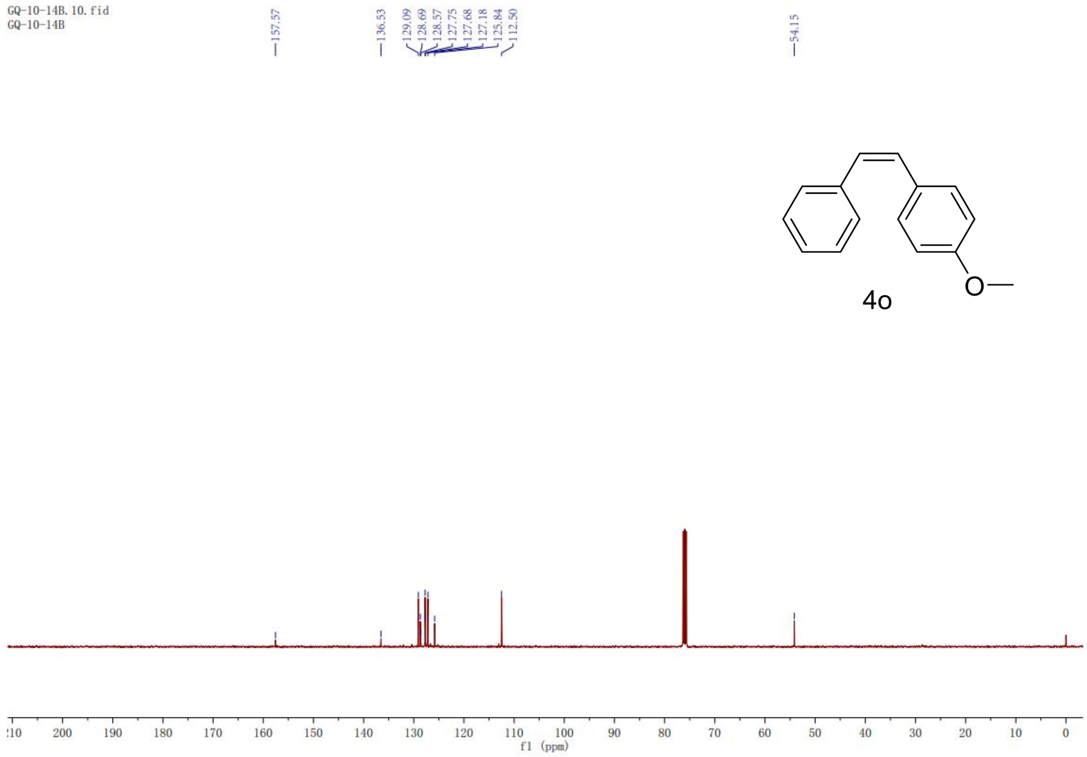
GQ-10-14B.10.fid
GQ-10-14B
157.57
136.53
129.09 128.69 128.57 127.75 127.68 127.18 125.84 112.50
54.15
4o
200 190 180 170 160 150 140 130 120 110 100 90 80 70 60 50 40 30 20 10 0
f1 (ppm)

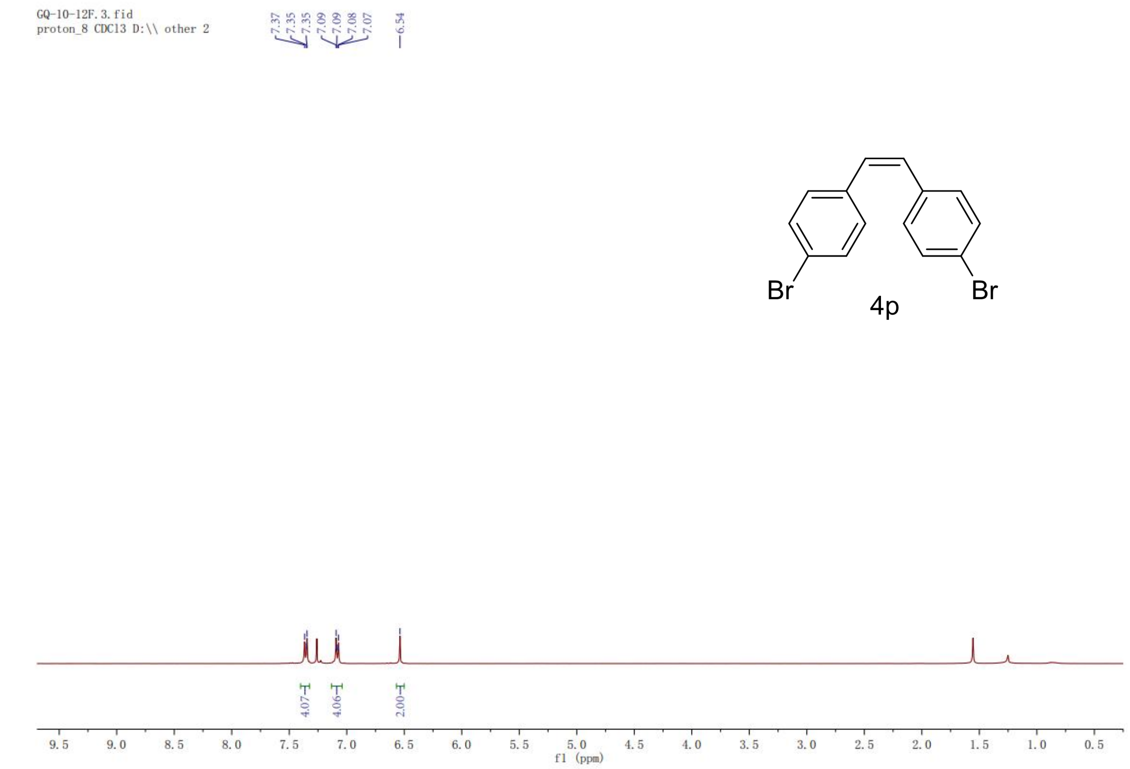
GQ-10-12F.3.fid
proton_8 CDCl3 D:\\ other 2
7.37
7.35
7.35
7.09
7.09
7.08
7.07
6.54
Br
Br
4p
4.07
4.06
2.00
9.5 9.0 8.5 8.0 7.5 7.0 6.5 6.0 5.5 5.0 4.5 4.0 3.5 3.0 2.5 2.0 1.5 1.0 0.5
f1 (ppm)

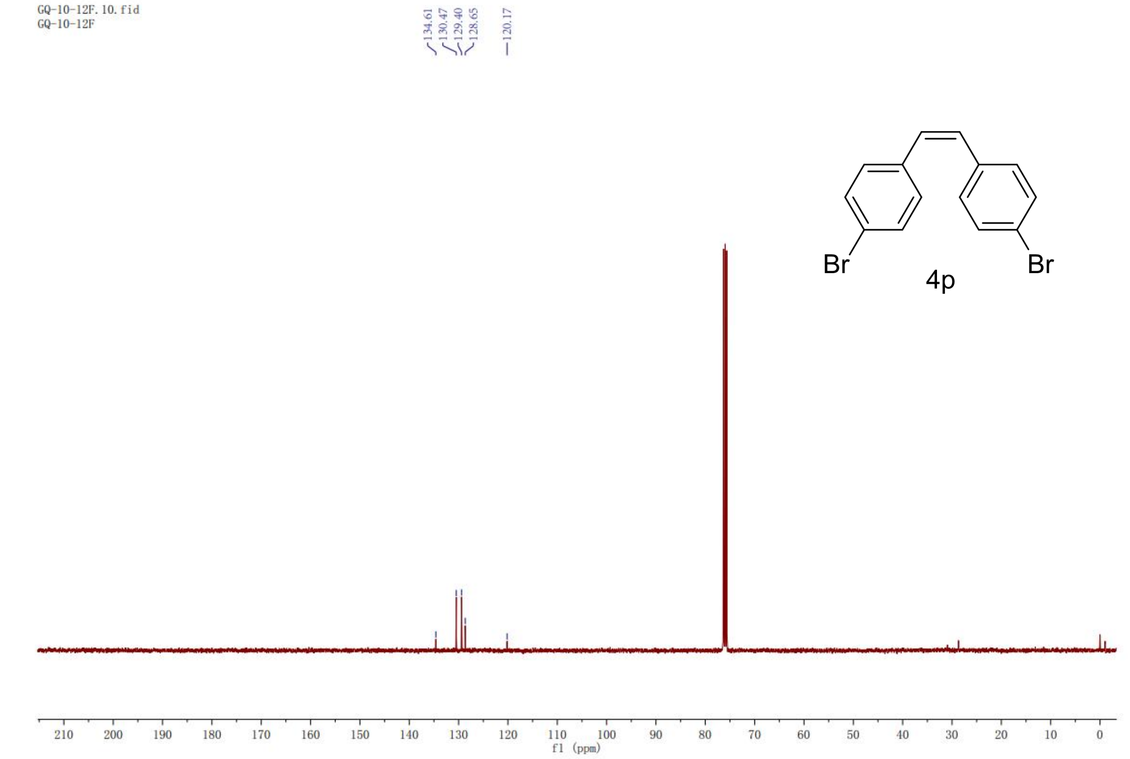
GQ-10-12F.10.fid
GQ-10-12F
134.61
130.47
129.40
128.65
120.17
Br
Br
4p
210 200 190 180 170 160 150 140 130 120 110 100 90 80 70 60 50 40 30 20 10 0
f1 (ppm)

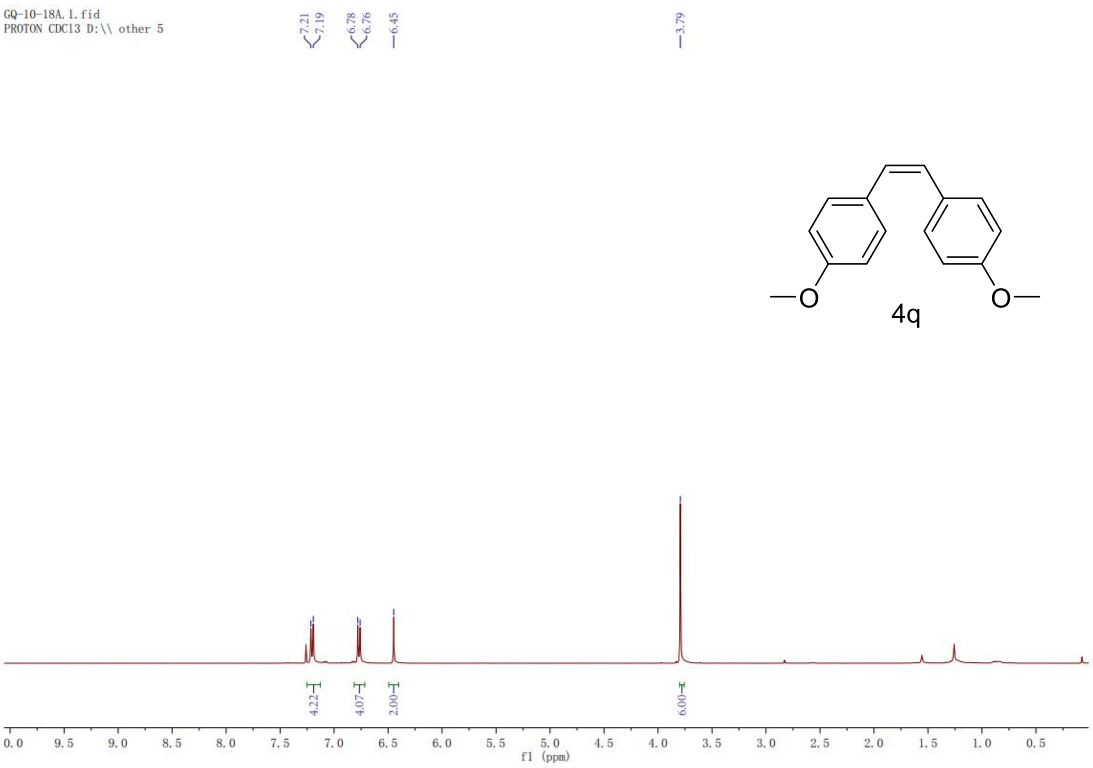
GQ-10-18A.1.fid
PROTON CDCl3 D:\\ other 5
7.21
7.19
6.78
6.76
6.45
3.79
4q
4.22
4.07
2.00
6.00
10.0 9.5 9.0 8.5 8.0 7.5 7.0 6.5 6.0 5.5 5.0 4.5 4.0 3.5 3.0 2.5 2.0 1.5 1.0 0.5
f1 (ppm)

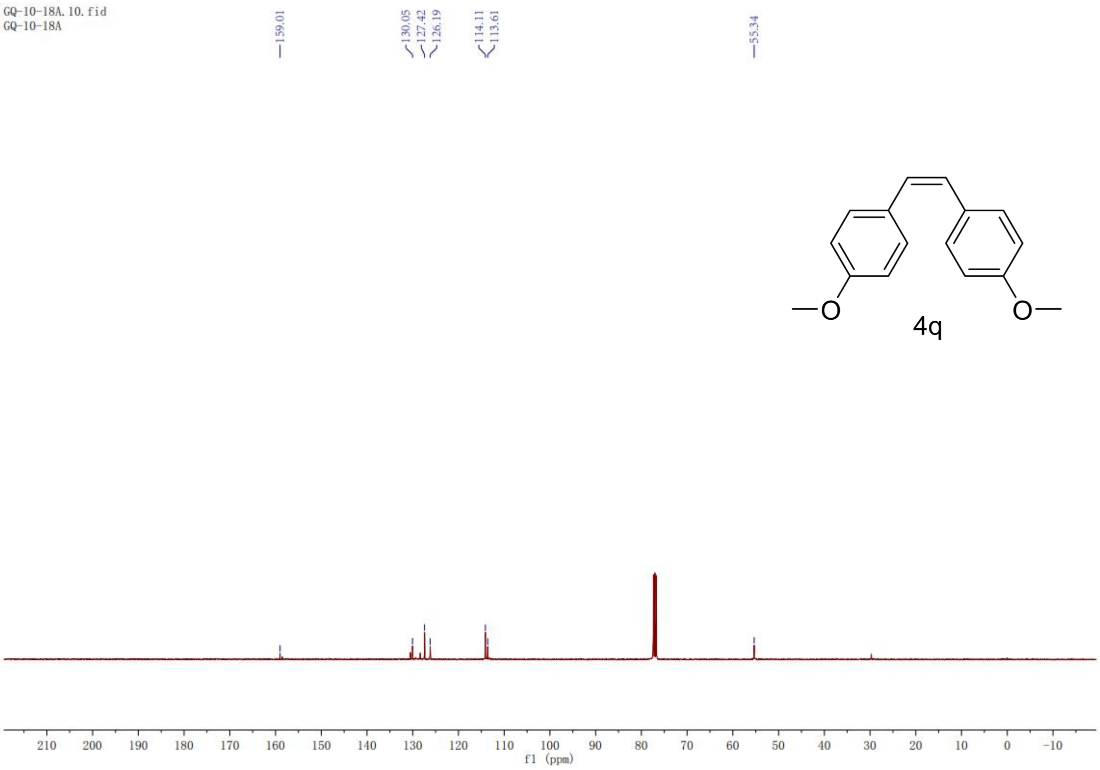
GQ-10-18A.10.fid
GQ-10-18A
159.01
130.05
127.42
126.19
114.11
113.61
55.34
4q
210 200 190 180 170 160 150 140 130 120 110 100 90 80 70 60 50 40 30 20 10 0 -10
f1 (ppm)

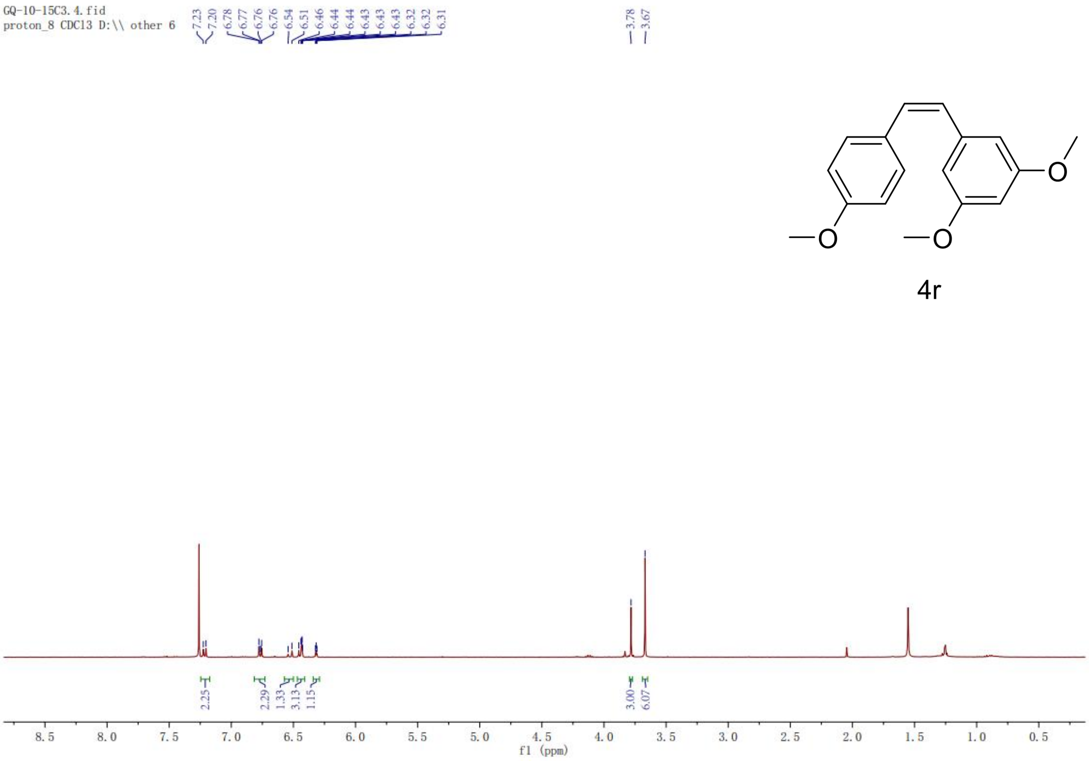
GQ-10-15C3.4.fid
proton_8 CDCl3 D:\\ other 6
7.23
7.20
6.78
6.77
6.76
6.76
6.54
6.51
6.46
6.44
6.44
6.43
6.43
6.43
6.32
6.32
6.31
3.78
3.67
2.25
2.29
1.33
3.13
1.15
3.00
6.07
8.5 8.0 7.5 7.0 6.5 6.0 5.5 5.0 4.5 4.0 3.5 3.0 2.5 2.0 1.5 1.0 0.5
f1 (ppm)

4r

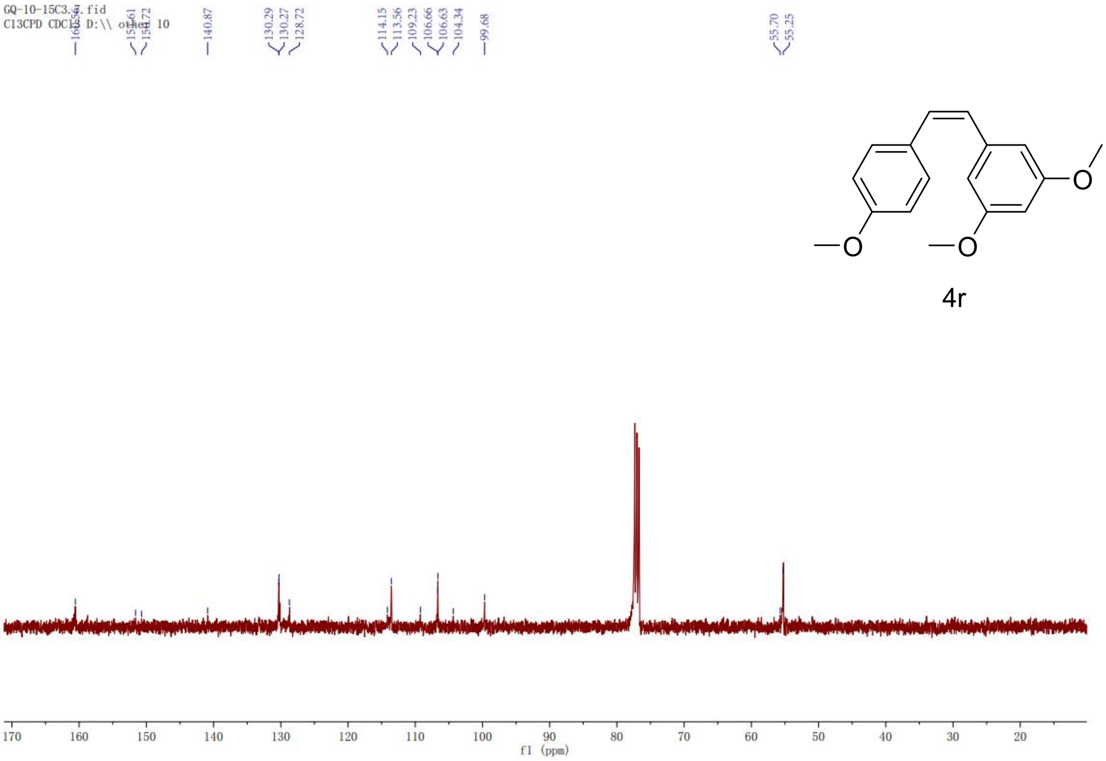
140.87
130.29
130.27
128.72
114.15
113.56
109.23
106.66
106.63
104.34
99.68
55.70
55.25
170 160 150 140 130 120 110 100 90 80 70 60 50 40 30 20
f1 (ppm)

4r

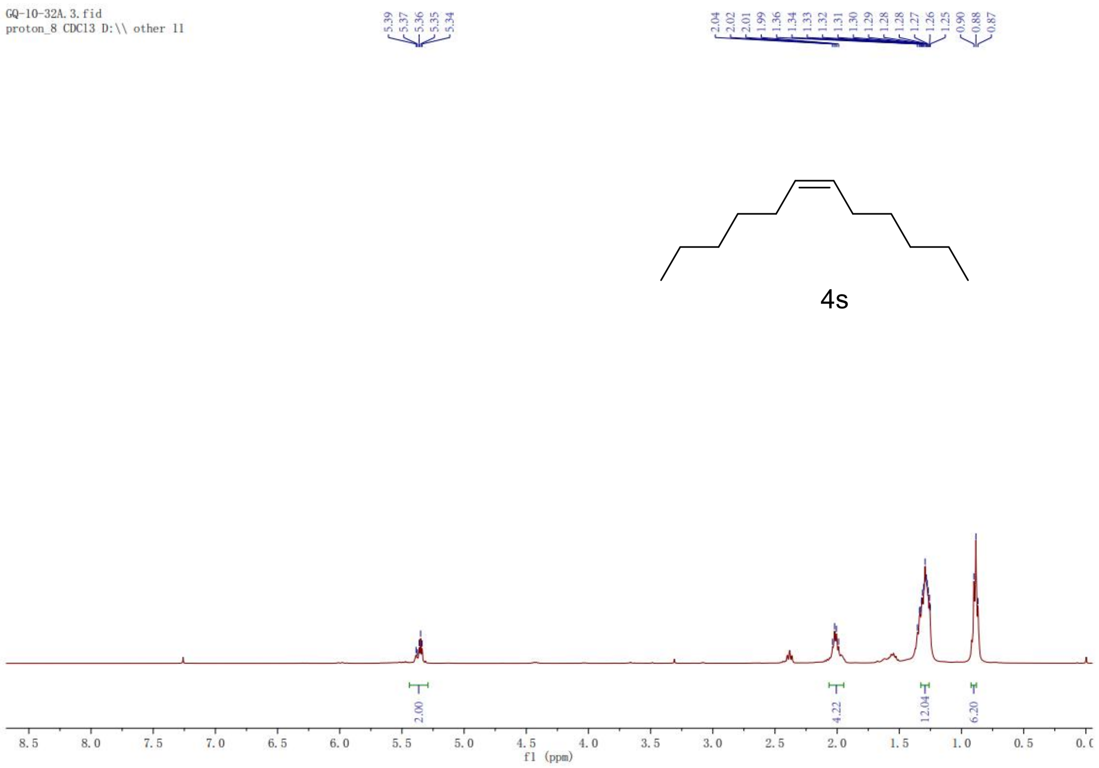
GQ-10-32A.3.fid
proton_8 CDCl3 D:\\ other 11
5.39
5.37
5.36
5.35
5.34
2.04
2.02
2.01
1.99
1.36
1.34
1.33
1.32
1.31
1.30
1.29
1.28
1.28
1.27
1.26
1.25
0.90
0.88
0.87
4s
2.00
4.22
12.04
6.20
f1 (ppm)

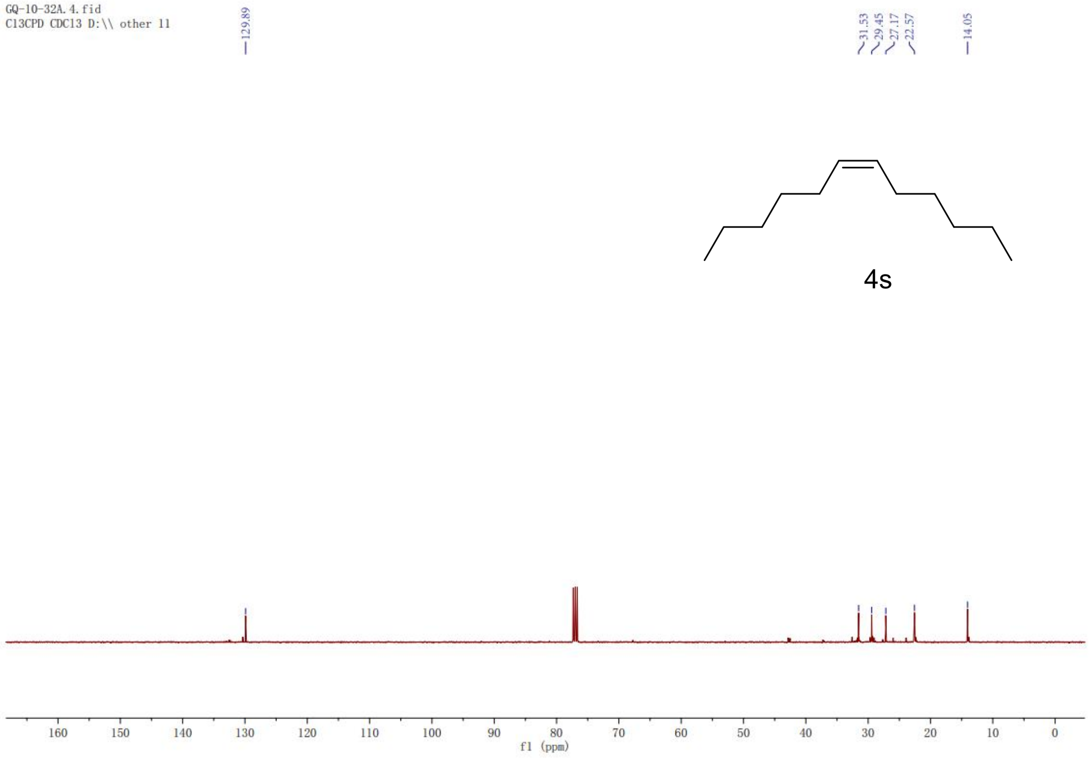
GQ-10-32A.4.fid
C13CPD CDCl3 D:\\ other 11
129.89
31.53
29.45
27.17
22.57
14.05
4s
f1 (ppm)

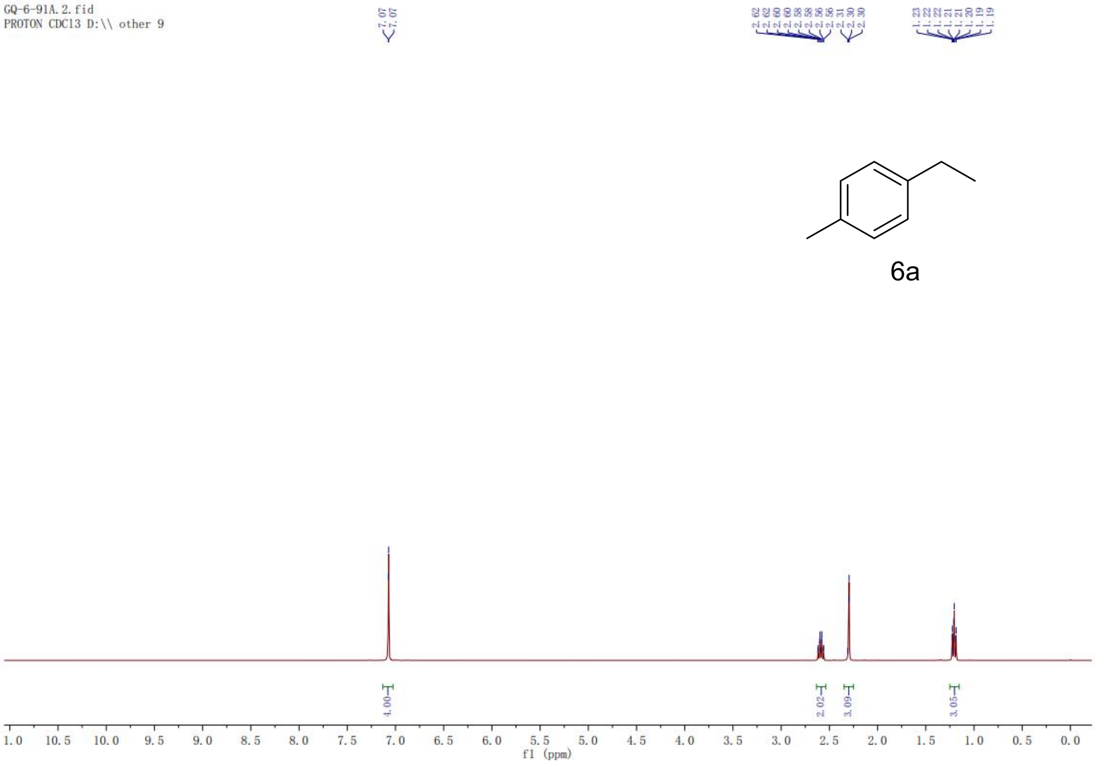
GQ-6-91A.2.fid
PROTON CDCl3 D:\\ other 9
7.07
7.07
2.62
2.62
2.60
2.60
2.58
2.58
2.56
2.56
2.31
2.30
2.30
1.23
1.22
1.22
1.21
1.21
1.20
1.19
1.19
4.00
2.02
3.09
3.05
f1 (ppm)

6a

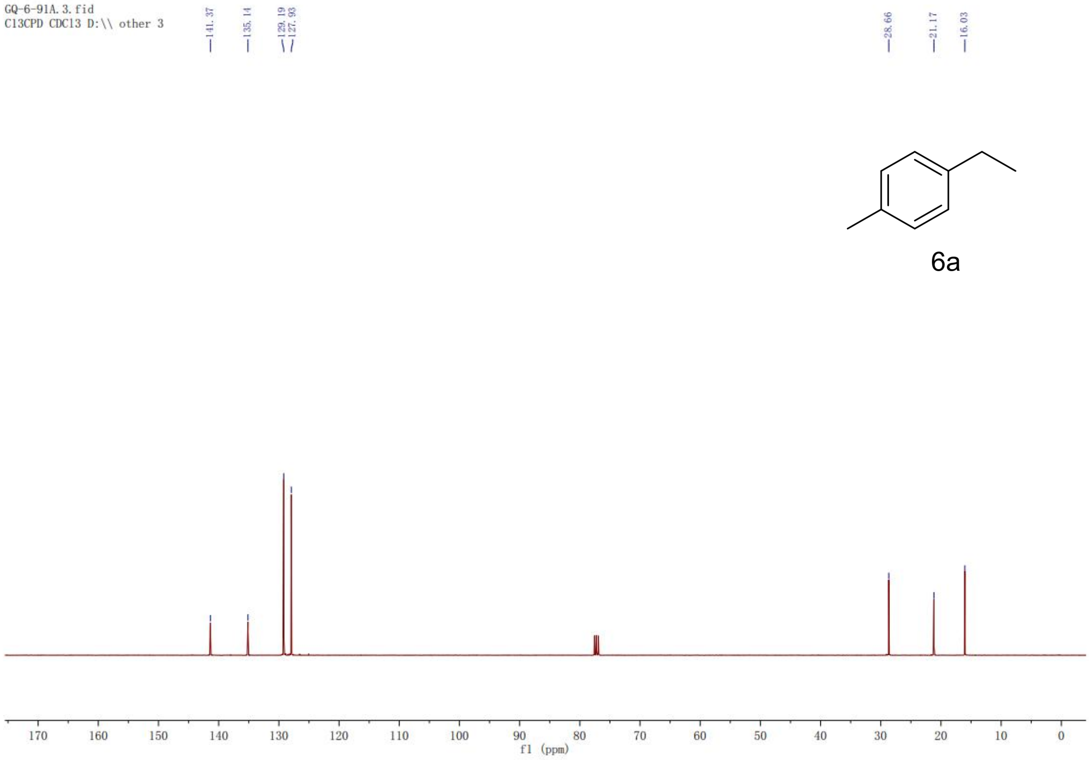
GQ-6-91A.3.fid
C13CPD CDCl3 D:\\ other 3
141.37
135.14
129.19
127.93
28.66
21.17
16.03
f1 (ppm)

6a

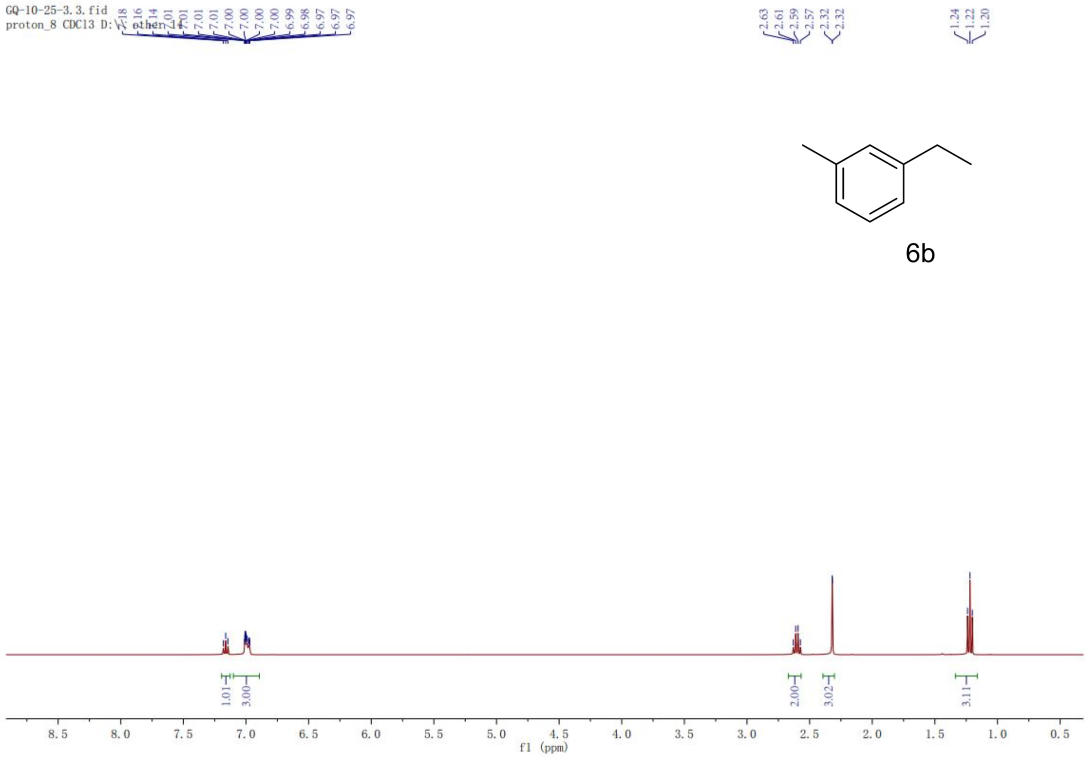
GQ-10-25-3.3.fid
proton_8 CDCl3 D:\\ other 11
7.18
7.16
7.14
7.01
7.01
7.01
7.01
7.00
7.00
7.00
7.00
6.99
6.98
6.97
6.97
6.97
2.63
2.61
2.59
2.57
2.32
2.32
1.24
1.22
1.20
6b
1.01
3.00
2.00
3.02
3.11
8.5
8.0
7.5
7.0
6.5
6.0
5.5
5.0
4.5
4.0
3.5
3.0
2.5
2.0
1.5
1.0
0.5
f1 (ppm)

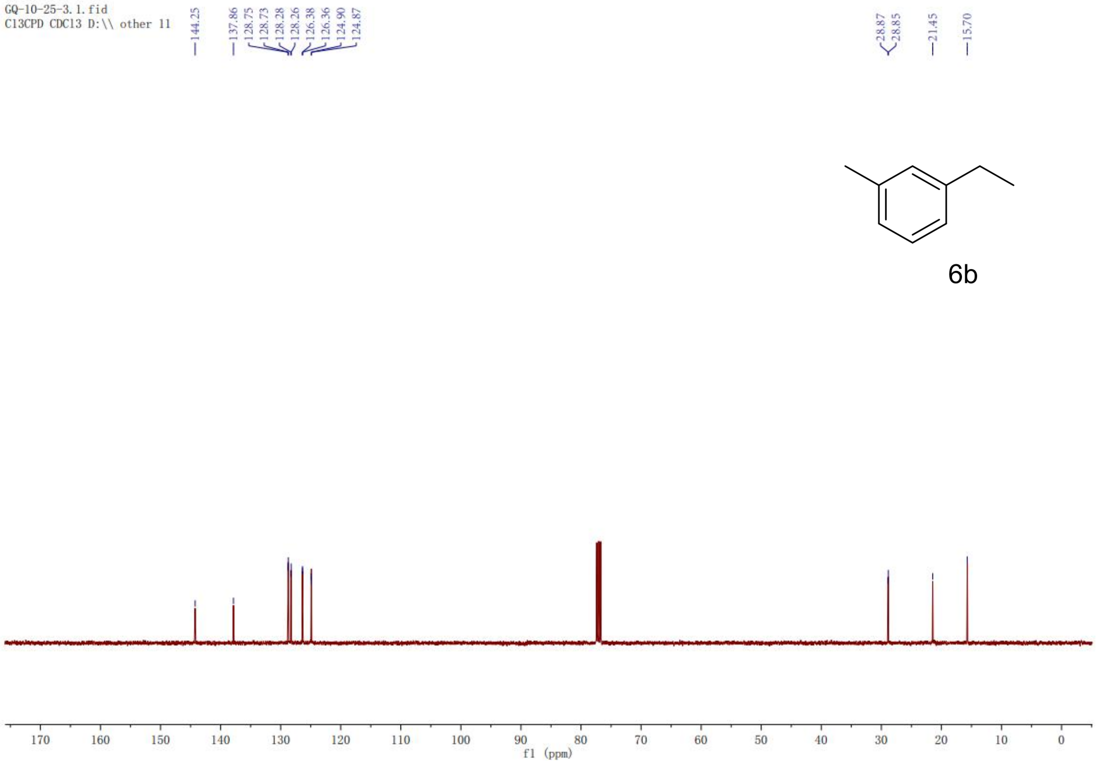
GQ-10-25-3.1.fid
C13CPD CDCl3 D:\\ other 11
144.25
137.86
128.75
128.73
128.28
128.26
126.38
126.36
124.90
124.87
28.87
28.85
21.45
15.70
6b
170
160
150
140
130
120
110
100
90
80
70
60
50
40
30
20
10
0
f1 (ppm)

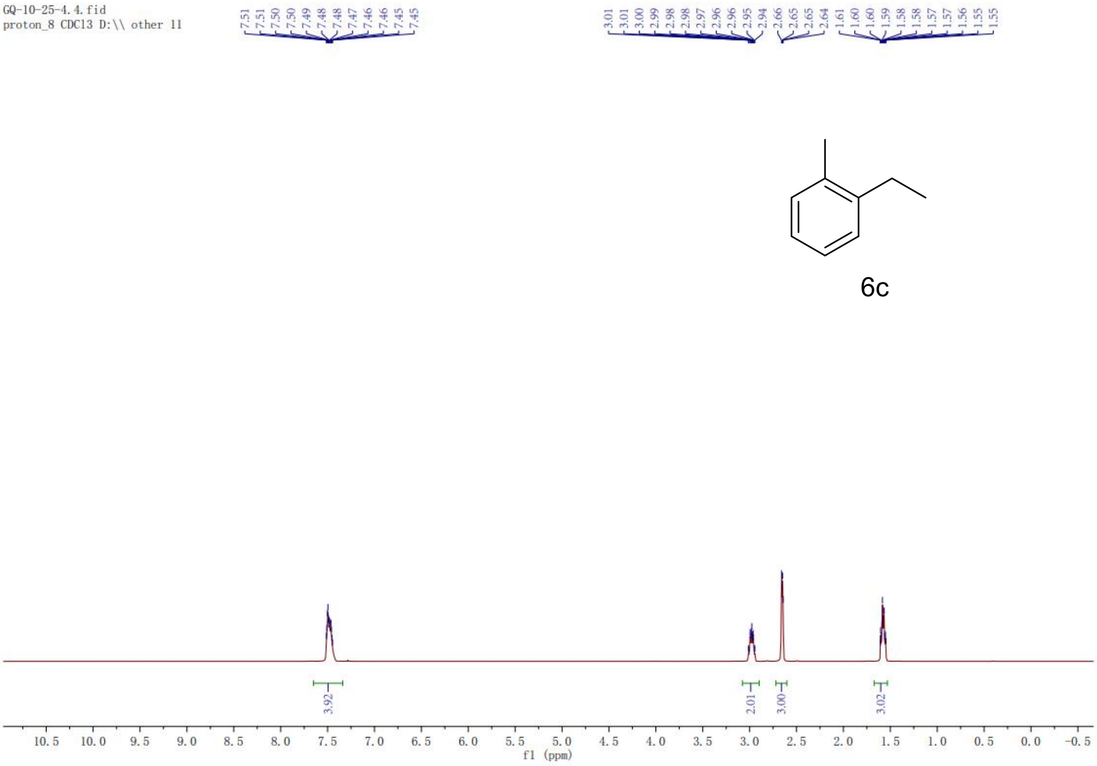
GQ-10-25-4.4.fid
proton_8 CDCl3 D:\\ other 11
7.51 7.51 7.50 7.50 7.49 7.48 7.48 7.47 7.46 7.45 7.45
3.01 3.01 3.00 2.99 2.98 2.98 2.97 2.96 2.96 2.95 2.94 2.66 2.65 2.65 2.64 1.61 1.60 1.60 1.59 1.58 1.58 1.57 1.57 1.56 1.55 1.55
6c
3.92
2.01
3.00
3.02
10.5 10.0 9.5 9.0 8.5 8.0 7.5 7.0 6.5 6.0 5.5 5.0 4.5 4.0 3.5 3.0 2.5 2.0 1.5 1.0 0.5 0.0 -0.5
f1 (ppm)

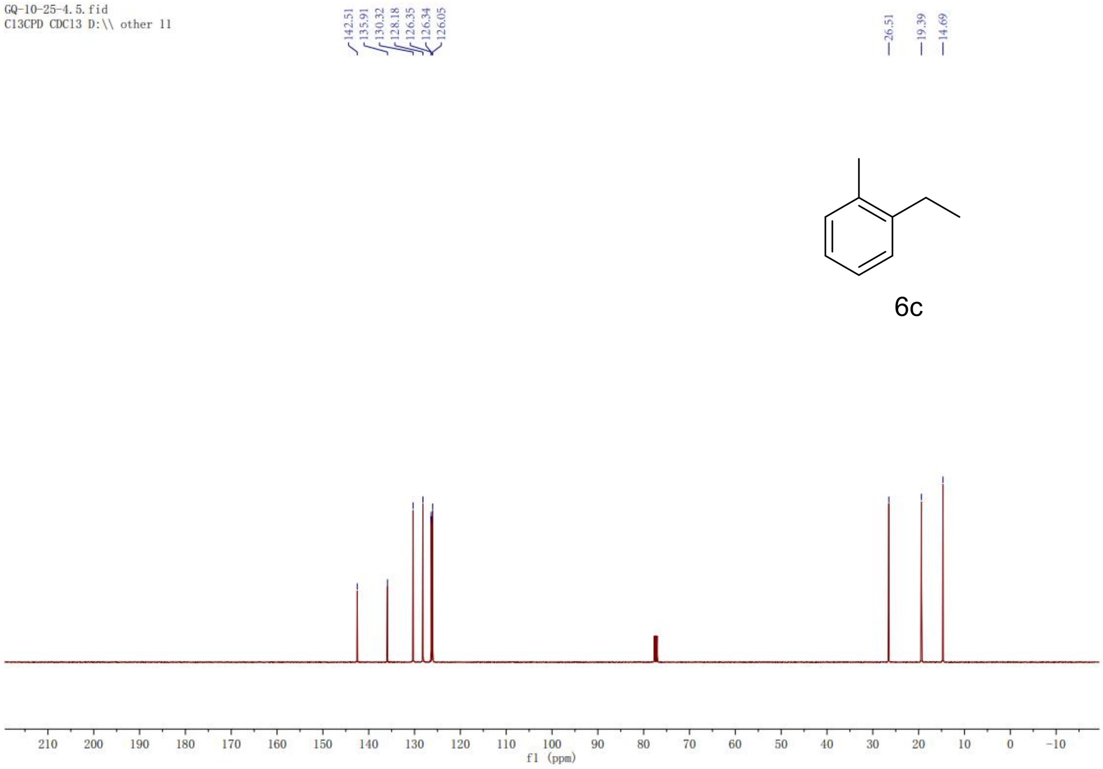
GQ-10-25-4.5.fid
C13CPD CDCl3 D:\\ other 11
142.51 135.91 130.32 128.18 126.35 126.34 126.05
26.51 19.39 14.69
6c
210 200 190 180 170 160 150 140 130 120 110 100 90 80 70 60 50 40 30 20 10 0 -10
f1 (ppm)

GQ-7-61B.1.fid
PROTON CDCl3 D:\\ other 20

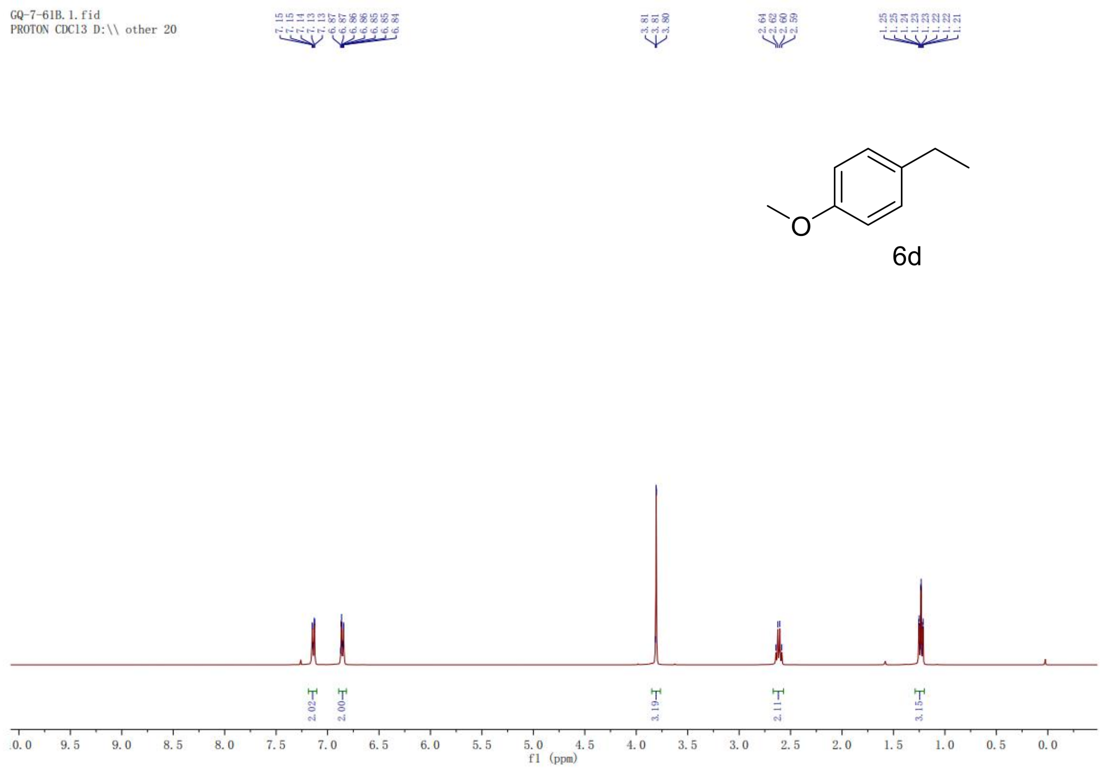


GQ-7-61B.2.fid
C13CPD CDCl3 D:\\ other 20

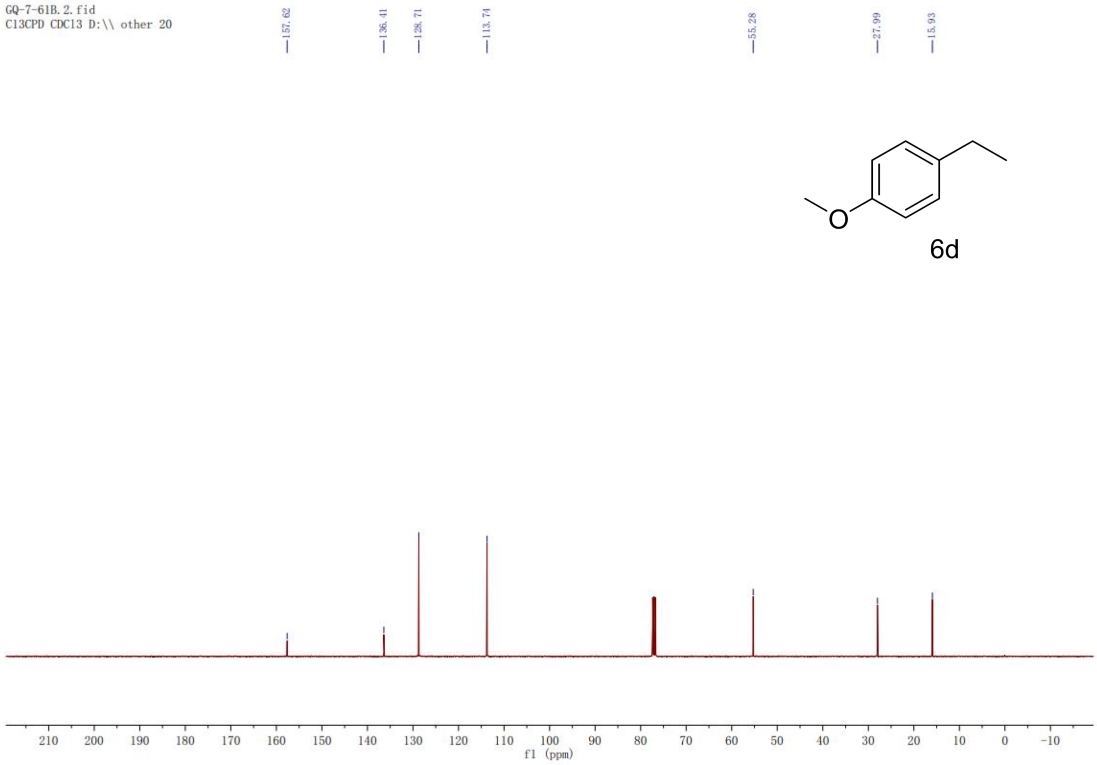

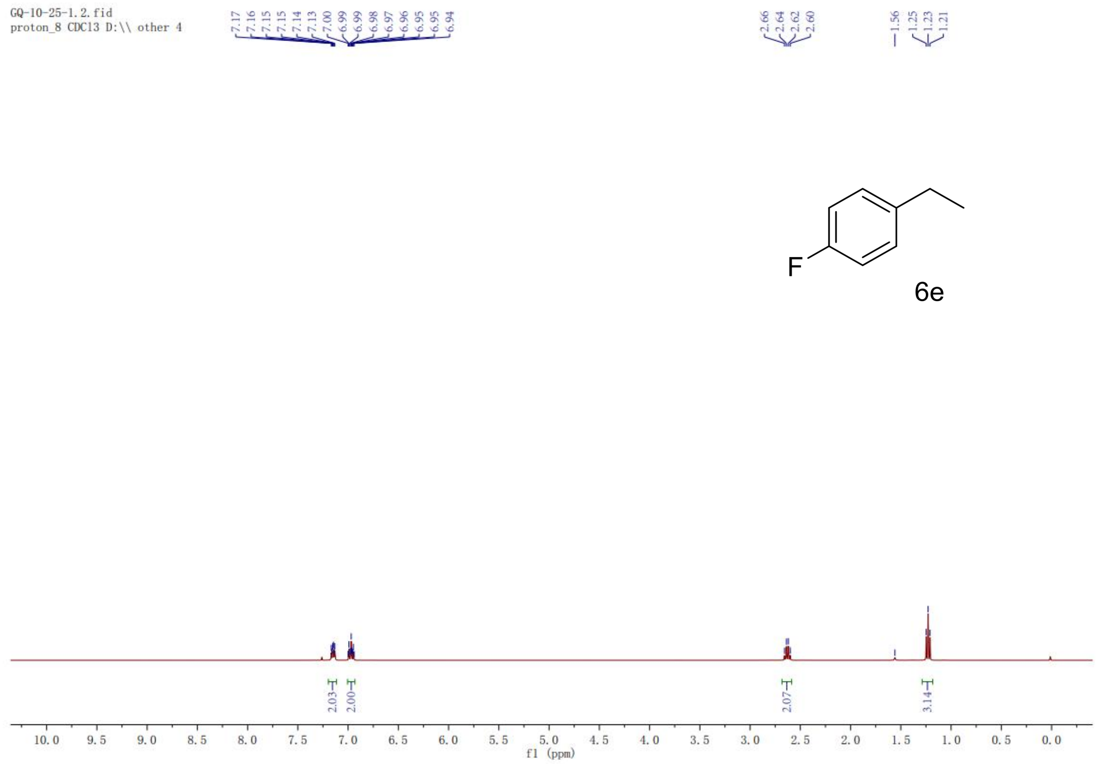
GQ-10-25-1.2.fid
proton_8 CDCl3 D:\\ other 4
7.17 7.16 7.15 7.15 7.14 7.13 7.00 6.99 6.99 6.98 6.97 6.96 6.95 6.95 6.94
2.66 2.64 2.62 2.60
1.56 1.25 1.23 1.21
F
6e
2.03 2.00 2.07 3.14
10.0 9.5 9.0 8.5 8.0 7.5 7.0 6.5 6.0 5.5 5.0 4.5 4.0 3.5 3.0 2.5 2.0 1.5 1.0 0.5 0.0
f1 (ppm)

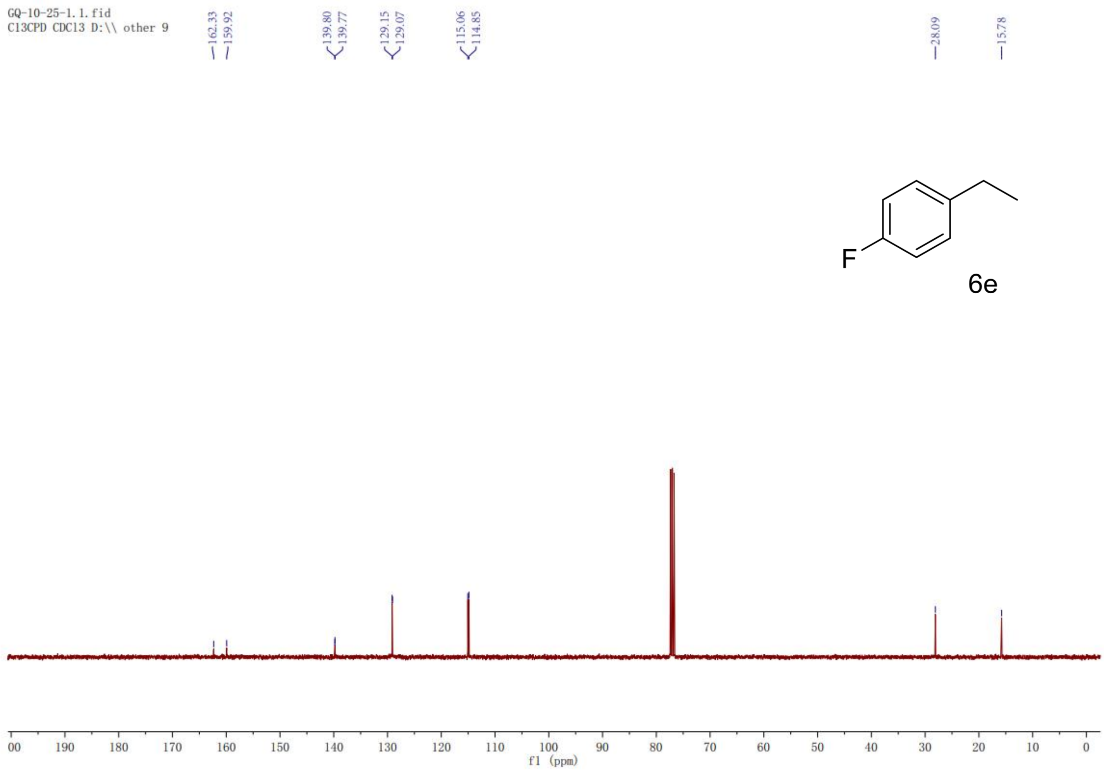
GQ-10-25-1.1.fid
C13CPD CDCl3 D:\\ other 9
162.33 159.92
139.80 139.77
129.15 129.07
115.06 114.85
28.09
15.78
F
6e
00 190 180 170 160 150 140 130 120 110 100 90 80 70 60 50 40 30 20 10 0
f1 (ppm)

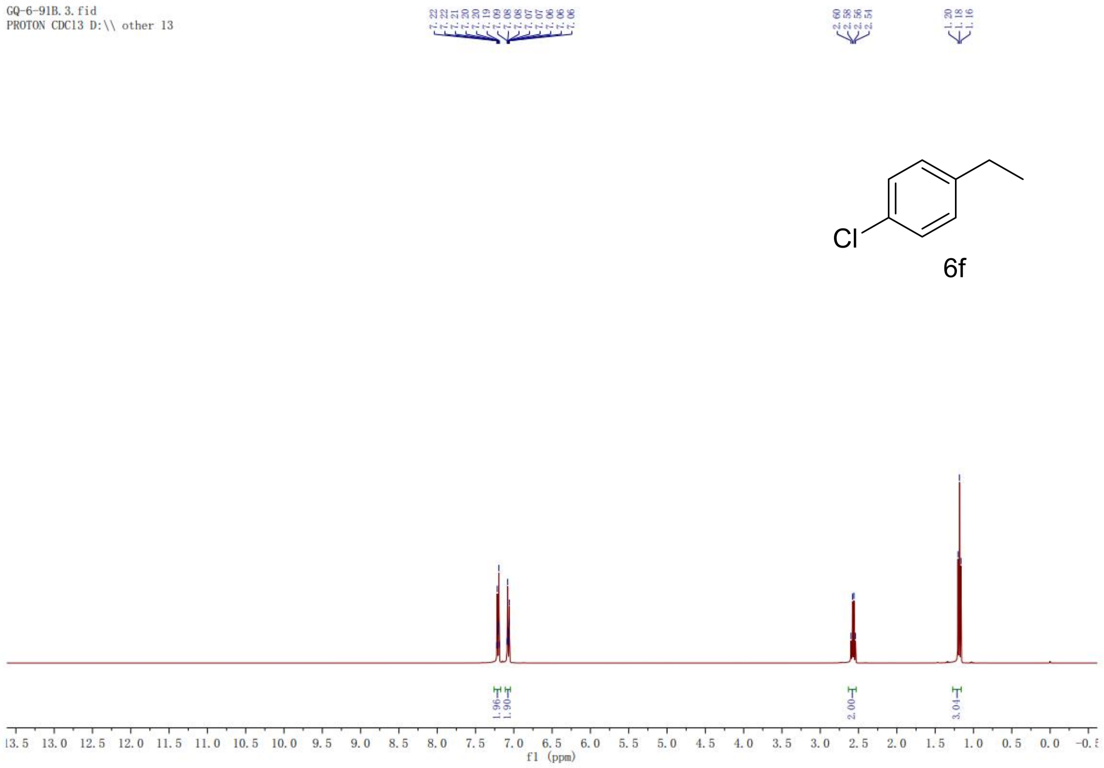
GQ-6-91B.3.fid
PROTON CDCl3 D:\\ other 13
7.22 7.22 7.21 7.20 7.20 7.19 7.09 7.08 7.08 7.07 7.07 7.06 7.06 7.06
2.60 2.58 2.56 2.54
1.20 1.18 1.16
Cl
6f
1.96 1.90
2.00
3.04
13.5 13.0 12.5 12.0 11.5 11.0 10.5 10.0 9.5 9.0 8.5 8.0 7.5 7.0 6.5 6.0 5.5 5.0 4.5 4.0 3.5 3.0 2.5 2.0 1.5 1.0 0.5 0.0
f1 (ppm)

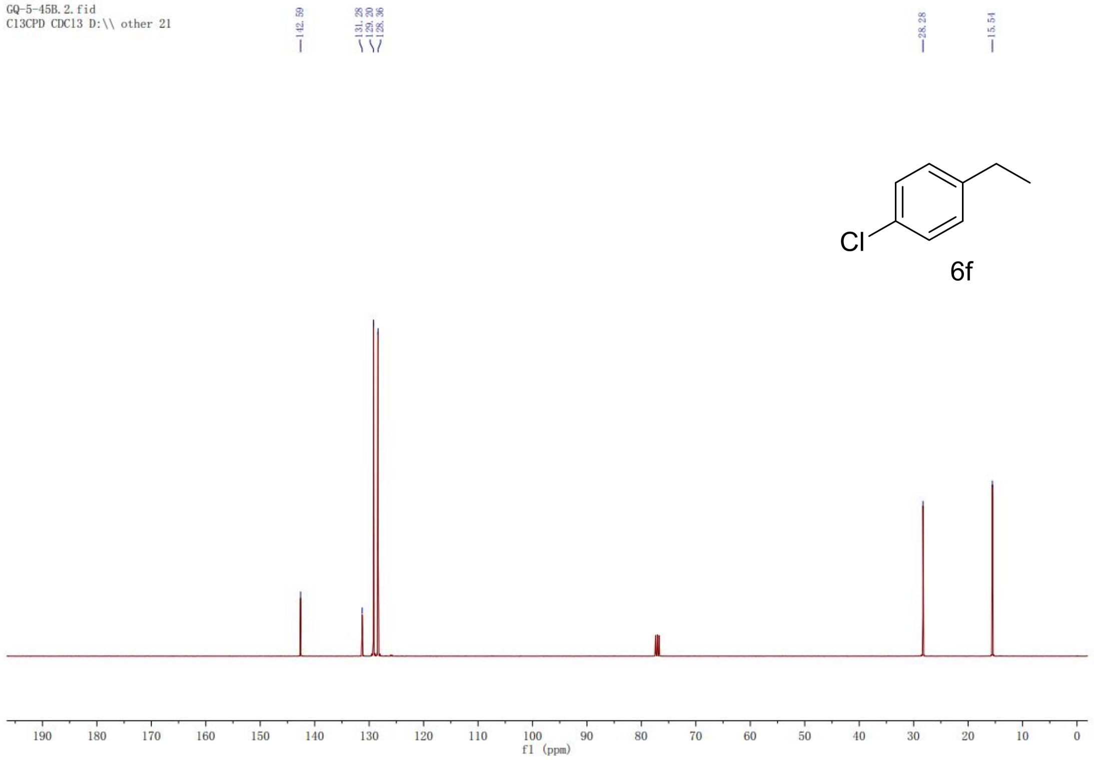
GQ-5-45B.2.fid
C13CPD CDCl3 D:\\ other 21
142.59
131.28 129.20 128.36
28.28
15.54
Cl
6f
190 180 170 160 150 140 130 120 110 100 90 80 70 60 50 40 30 20 10 0
f1 (ppm)

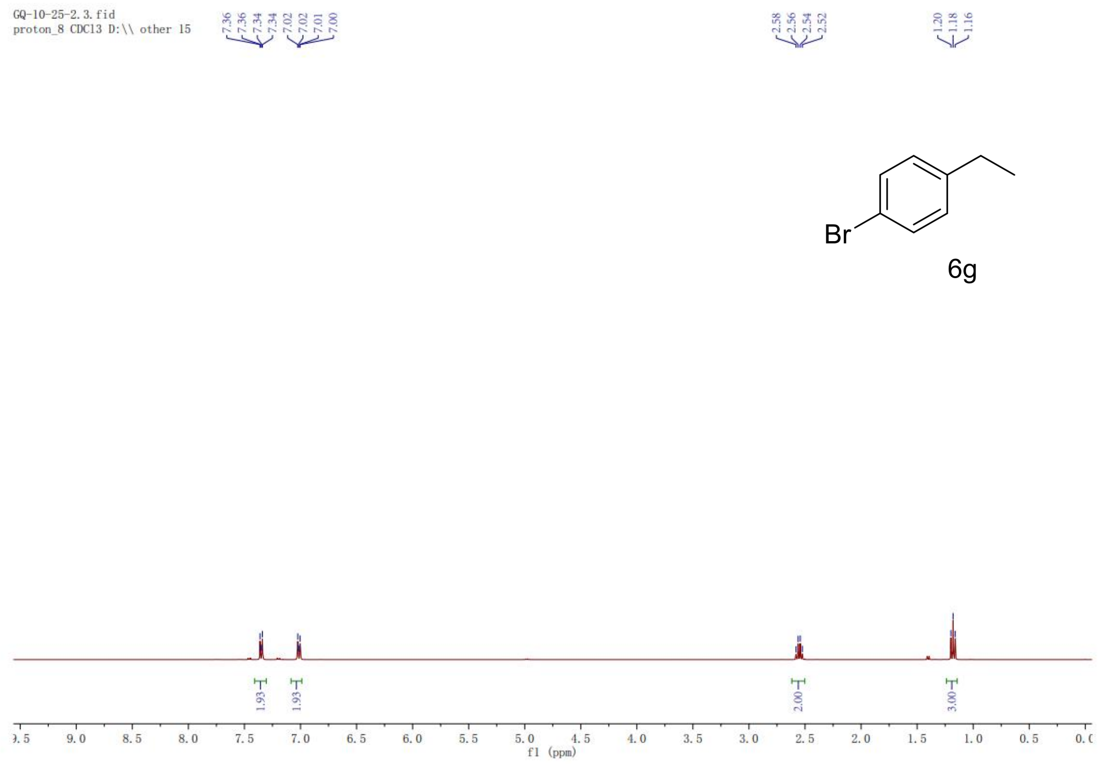
GQ-10-25-2.3.fid
proton_8 CDCl3 D:\\ other 15
7.36
7.36
7.34
7.34
7.02
7.02
7.01
7.00
2.58
2.56
2.54
2.52
1.20
1.18
1.16
Br
6g
1.93
1.93
2.00
3.00
9.0
8.5
8.0
7.5
7.0
6.5
6.0
5.5
5.0
4.5
4.0
3.5
3.0
2.5
2.0
1.5
1.0
0.5
f1 (ppm)

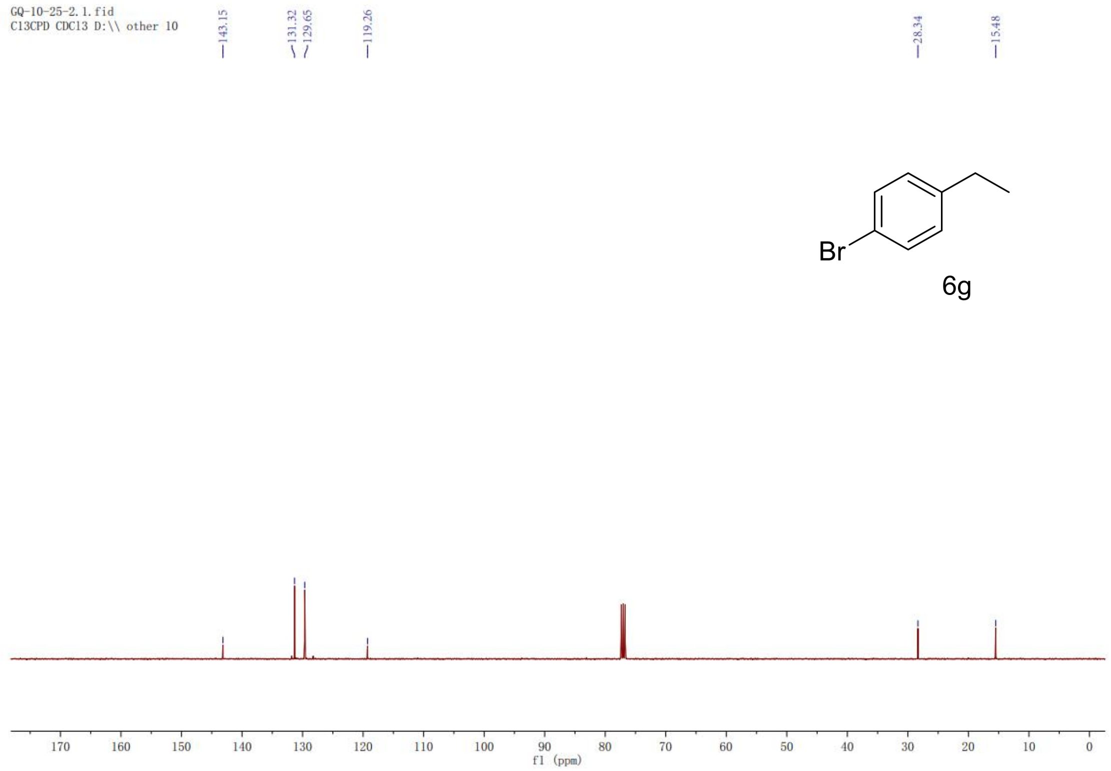
GQ-10-25-2.1.fid
C13CPD CDCl3 D:\\ other 10
143.15
131.32
129.65
119.26
28.34
15.48
Br
6g
170
160
150
140
130
120
110
100
90
80
70
60
50
40
30
20
10
0
f1 (ppm)

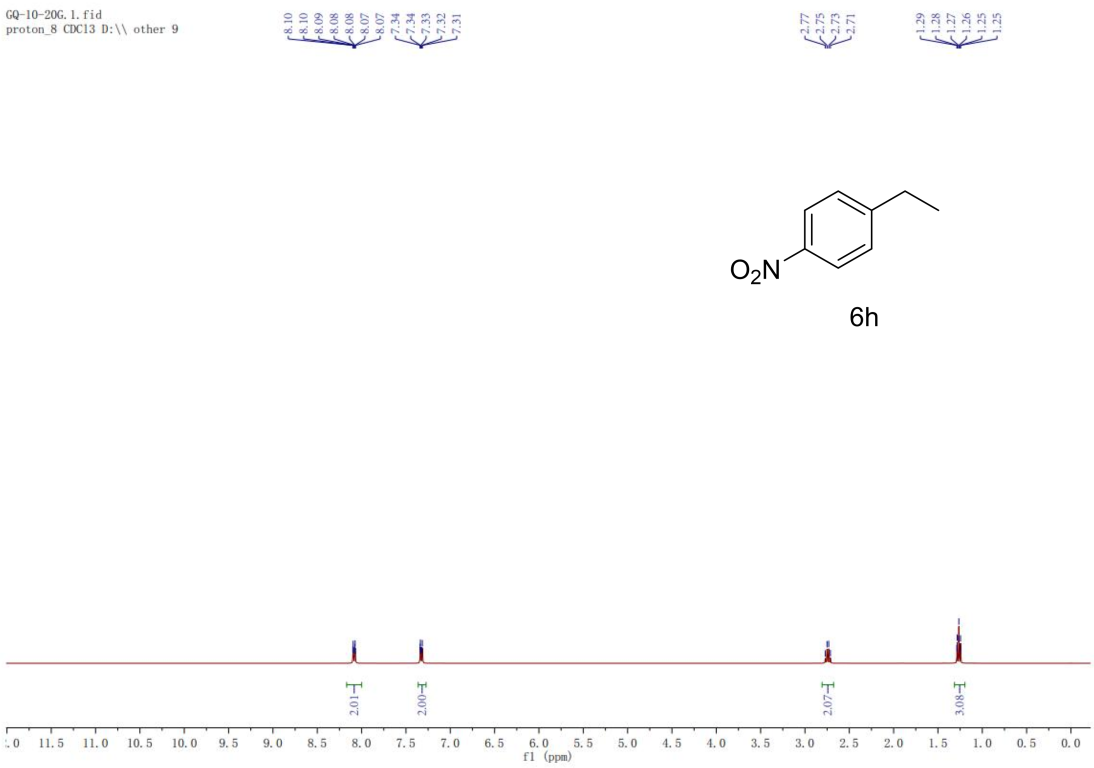
GQ-10-20G.1.fid
proton_8 CDCl3 D:\\ other 9
8.10
8.10
8.09
8.08
8.08
8.07
8.07
7.34
7.34
7.33
7.32
7.31
2.77
2.75
2.73
2.71
1.29
1.28
1.27
1.26
1.25
1.25
$O_2N$
6h
2.01
2.00
2.07
3.08
f1 (ppm)

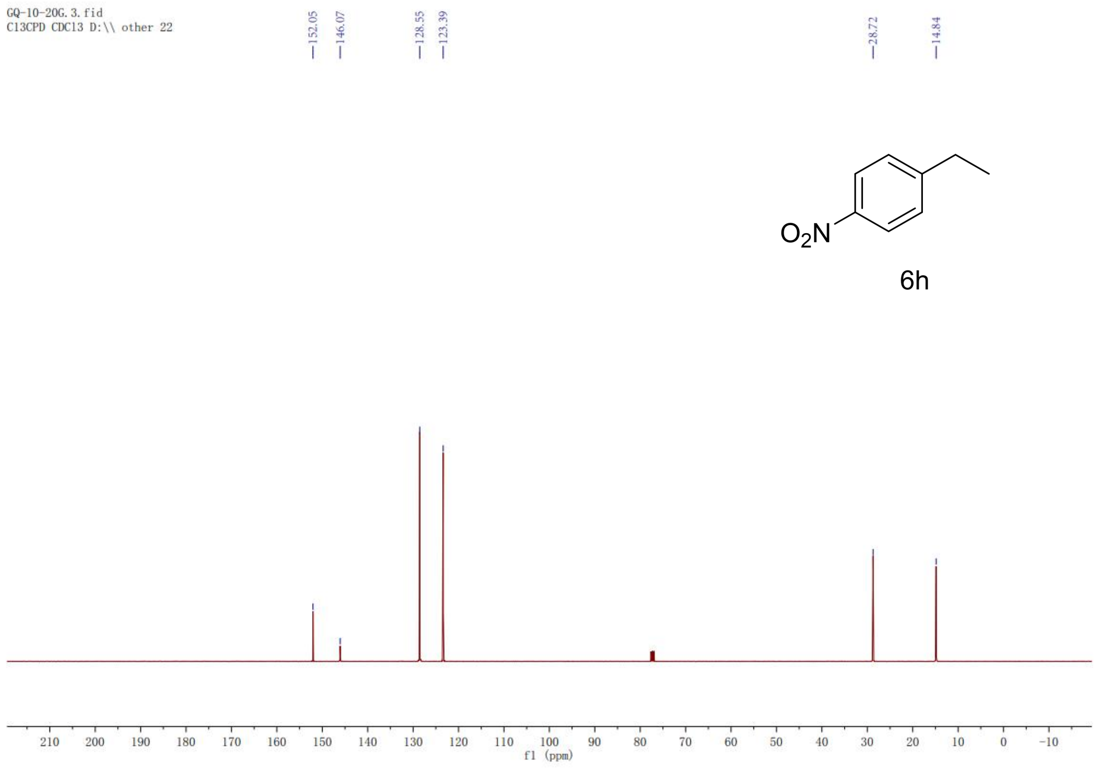
GQ-10-20G.3.fid
C13CPD CDCl3 D:\\ other 22
152.05
146.07
128.55
123.39
28.72
14.84
$O_2N$
6h
f1 (ppm)

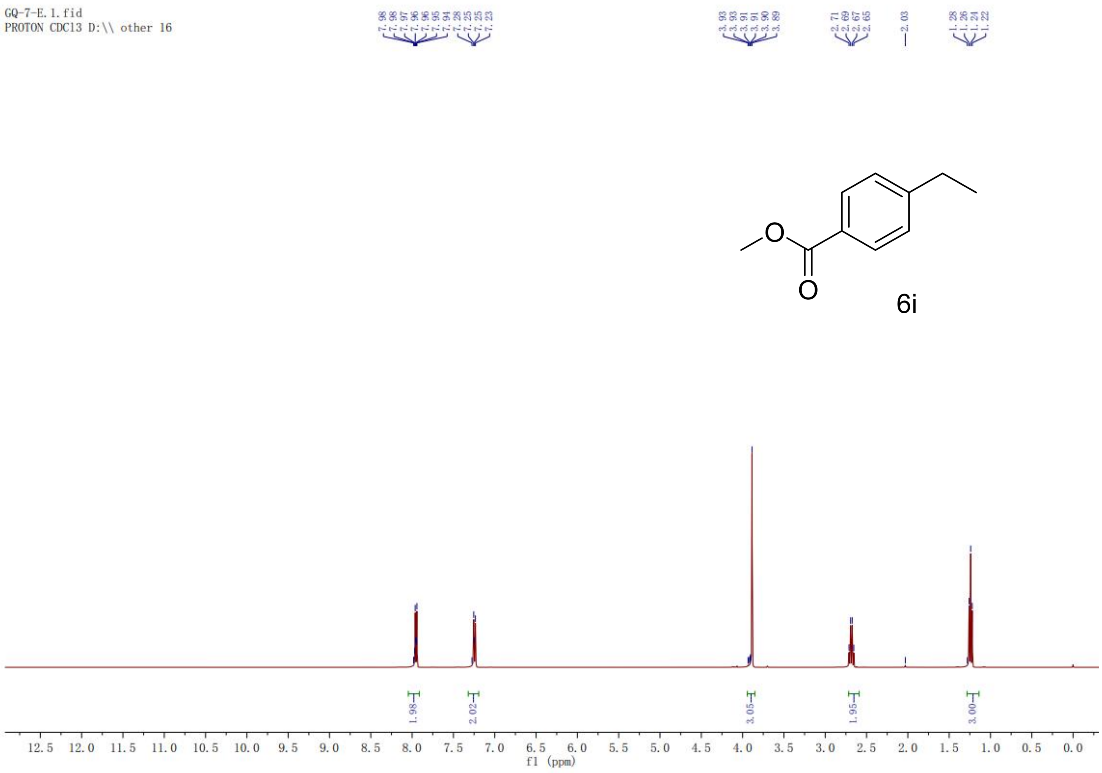
GQ-7-E.1.fid
PROTON CDCl3 D:\\ other 16
3.93
3.93
3.91
3.91
3.90
3.89
2.71
2.69
2.67
2.65
2.03
1.28
1.26
1.24
1.22
O
O
6i
1.98
2.02
3.05
1.95
3.00
12.5 12.0 11.5 11.0 10.5 10.0 9.5 9.0 8.5 8.0 7.5 7.0 6.5 6.0 5.5 5.0 4.5 4.0 3.5 3.0 2.5 2.0 1.5 1.0 0.5 0.0
f1 (ppm)

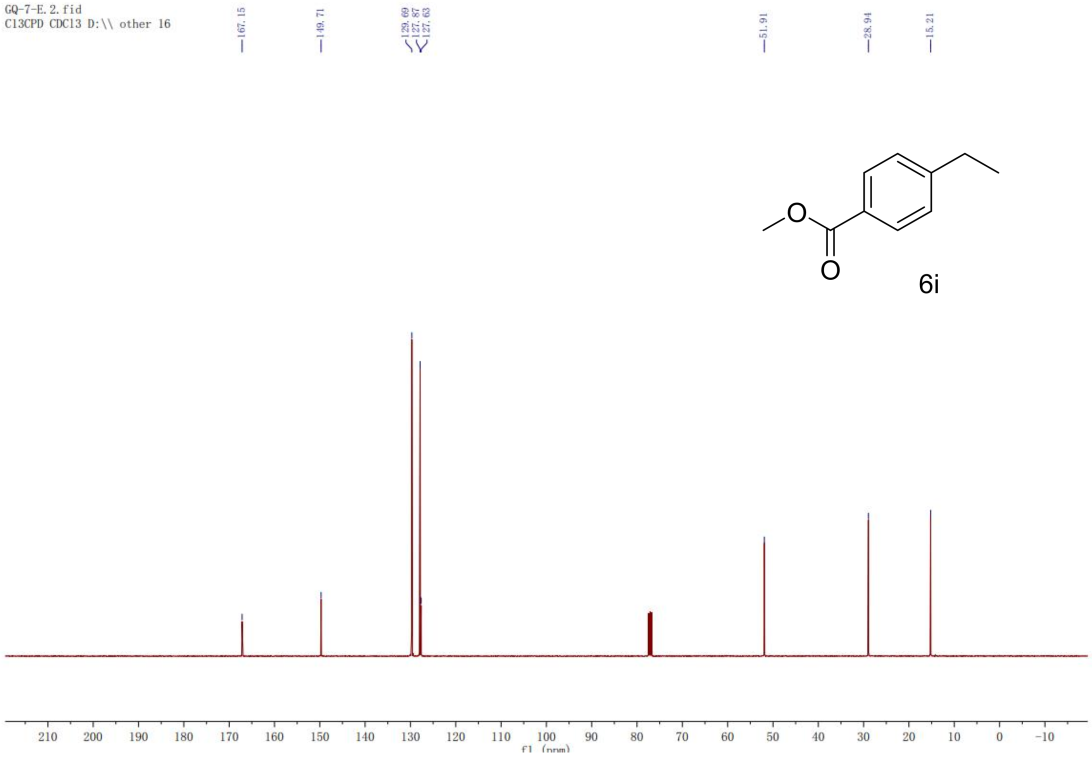
GQ-7-E.2.fid
C13CPD CDCl3 D:\\ other 16
167.15
149.71
129.69
127.87
127.63
51.91
28.94
15.21
O
O
6i
210 200 190 180 170 160 150 140 130 120 110 100 90 80 70 60 50 40 30 20 10 0 -10
f1 (ppm)

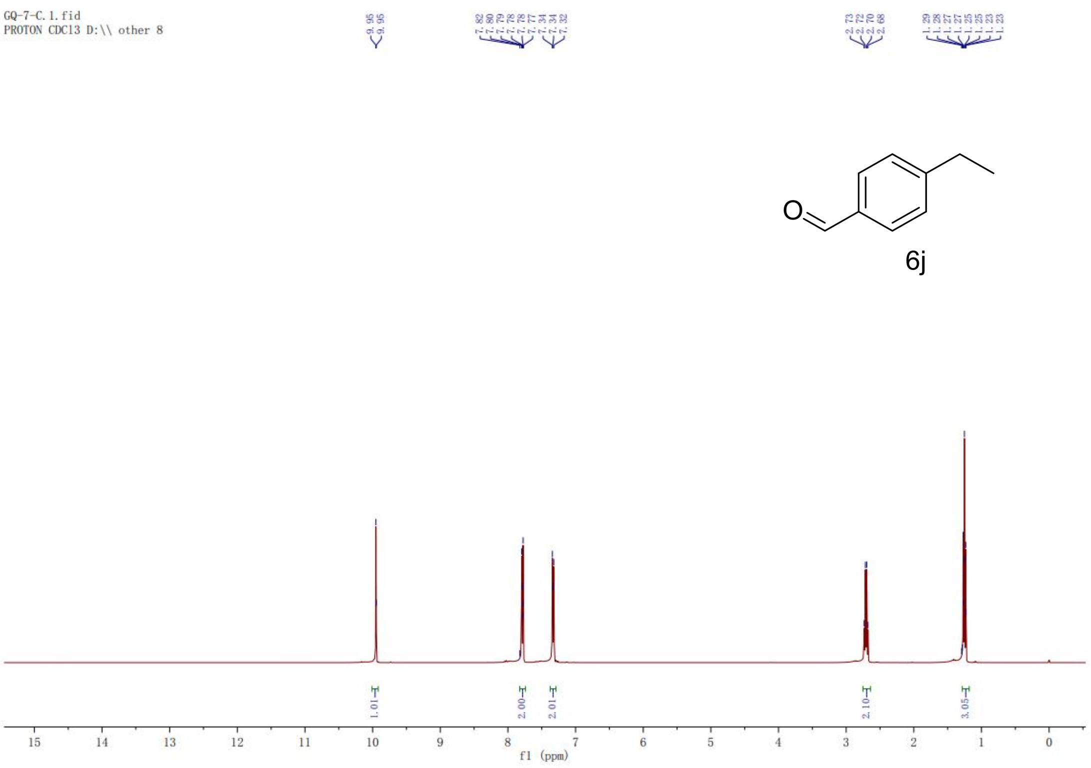
GQ-7-C.1.fid
PROTON CDC13 D:\\ other 8
6j
1.01
2.00
2.01
2.10
3.05
f1 (ppm)

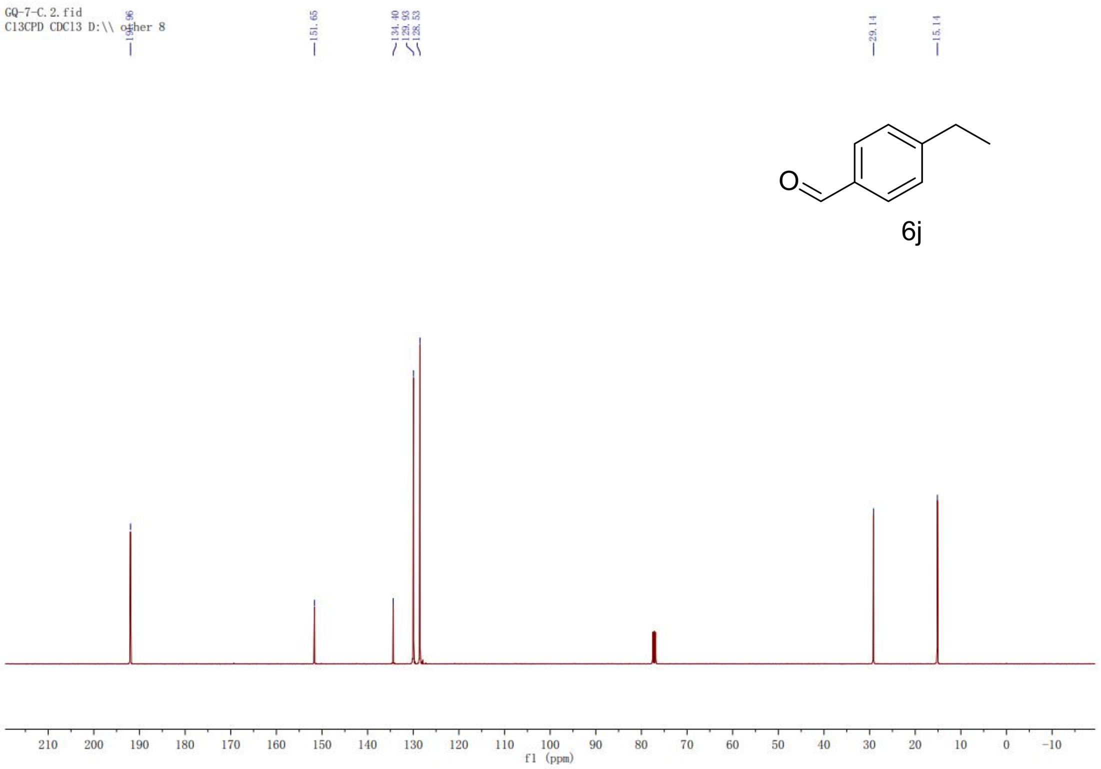
GQ-7-C.2.fid
C13CPD CDC13 D:\\ other 8
191.96
151.65
134.40
129.93
128.53
29.14
15.14
6j
f1 (ppm)

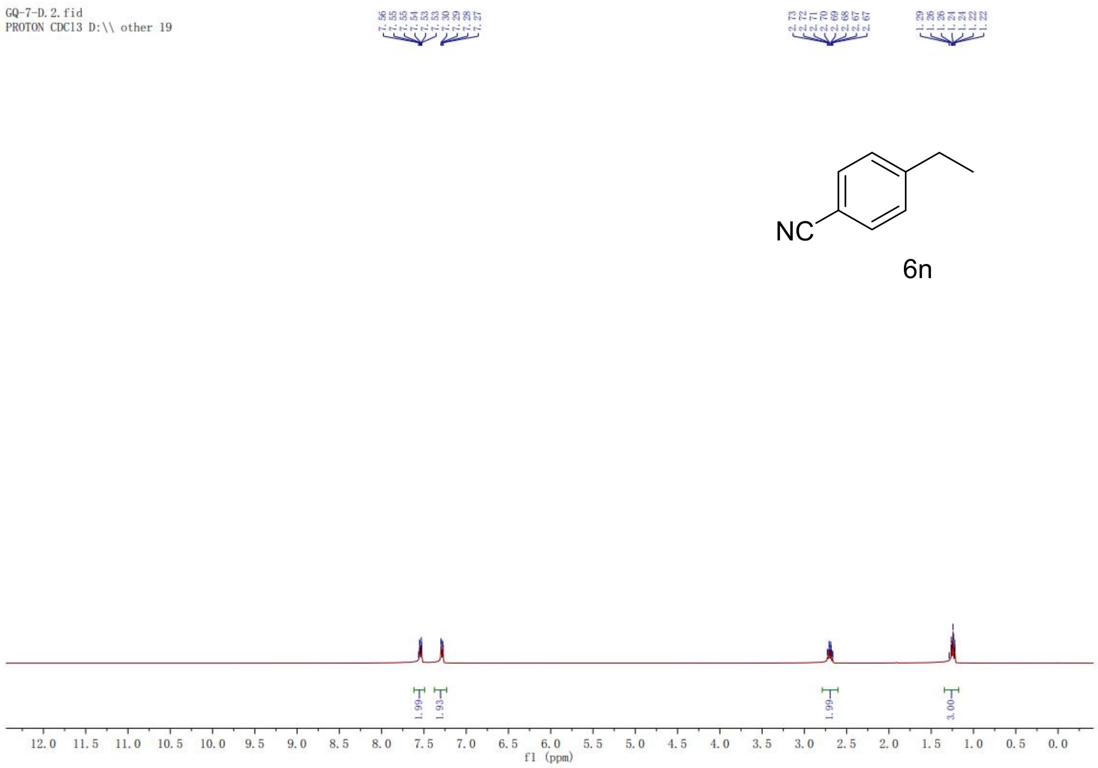
GQ-7-D.2.fid
PROTON CDCl3 D:\\ other 19
7.56 7.55 7.55 7.54 7.53 7.53 7.30 7.29 7.28 7.27
2.73 2.72 2.71 2.70 2.69 2.68 2.67 2.67
1.29 1.26 1.26 1.24 1.24 1.22 1.22
NC
6n
1.99 1.93 1.99 3.00
f1 (ppm)

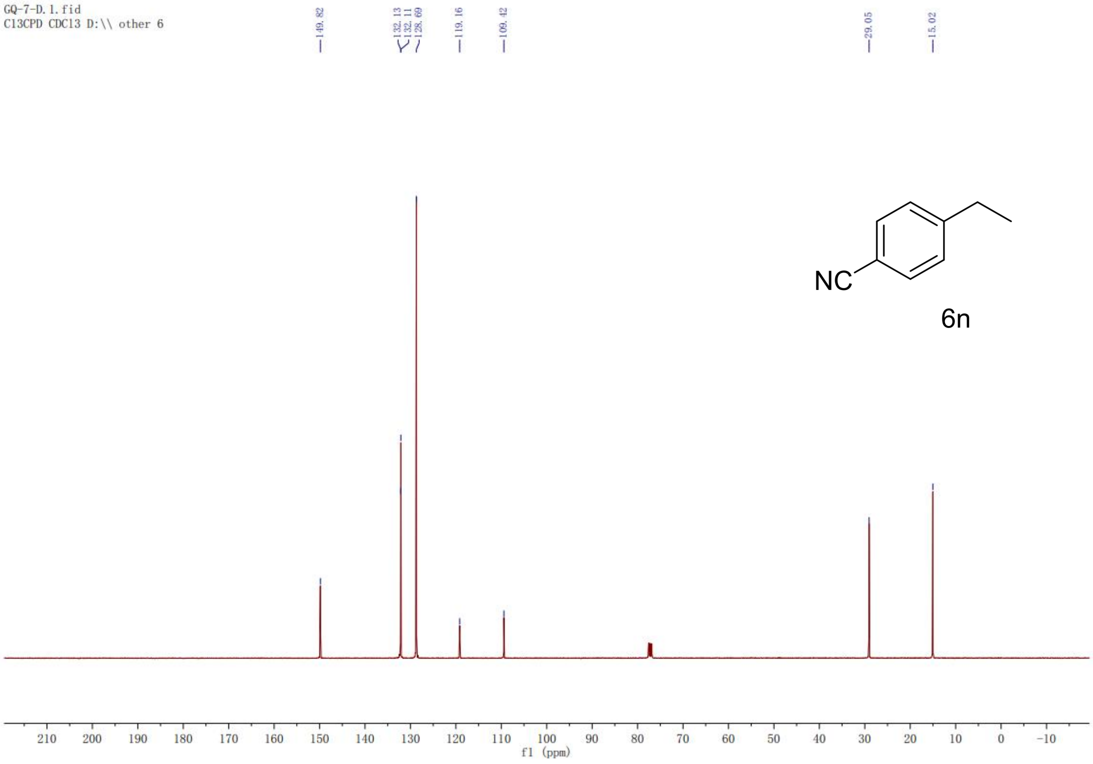
GQ-7-D.1.fid
C13CPD CDCl3 D:\\ other 6
149.82
132.13 132.11 128.69
119.16
109.42
29.05
15.02
NC
6n
f1 (ppm)

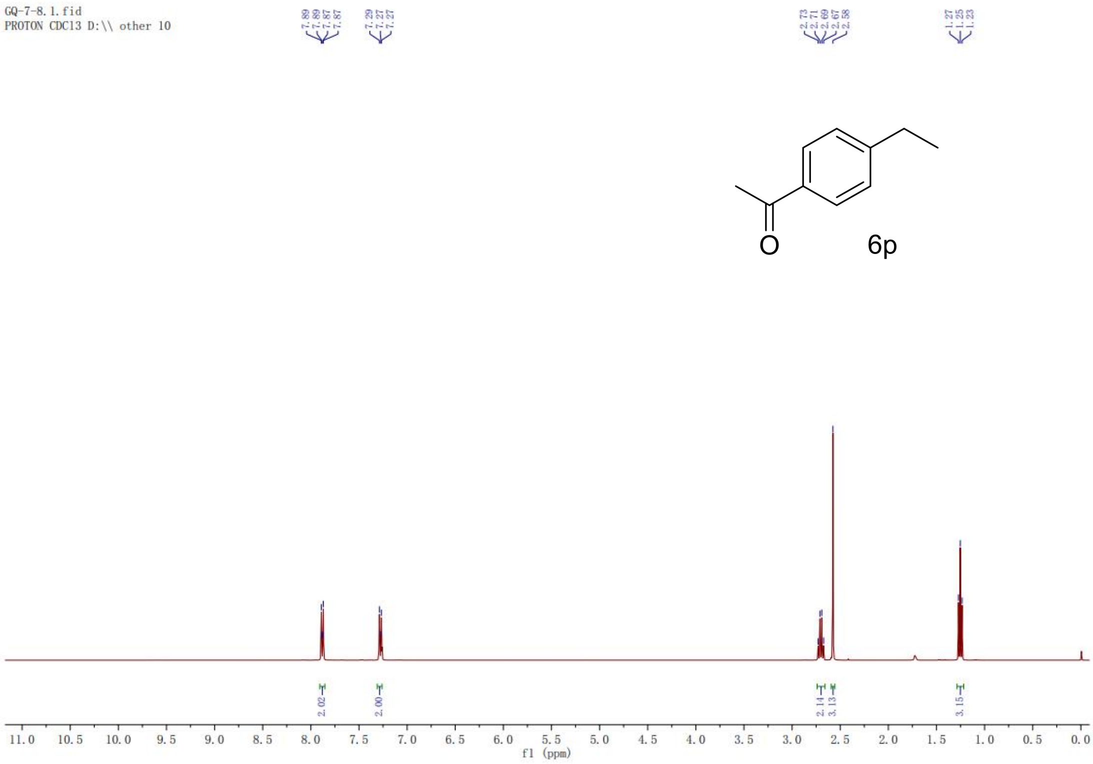
GQ-7-8.1.fid
PROTON CDCl3 D:\\ other 10
7.89
7.89
7.87
7.87
7.29
7.27
7.27
2.73
2.71
2.69
2.67
2.58
1.27
1.25
1.23
O
6p
2.02
2.00
2.14
3.13
3.15
f1 (ppm)

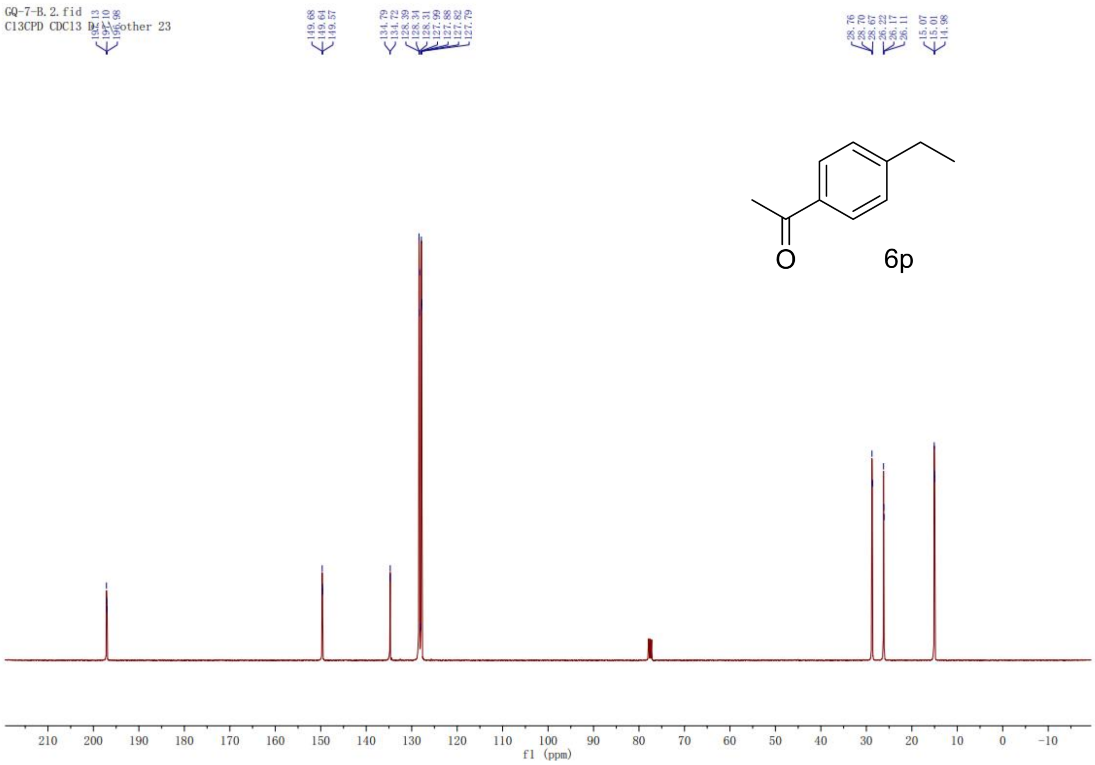
GQ-7-B.2.fid
C13CPD CDCl3 D:\\ other 23
149.68
149.64
149.57
134.79
134.72
128.39
128.34
128.31
127.99
127.88
127.82
127.79
28.76
28.70
28.67
26.22
26.17
26.11
15.07
15.01
14.98
O
6p
f1 (ppm)

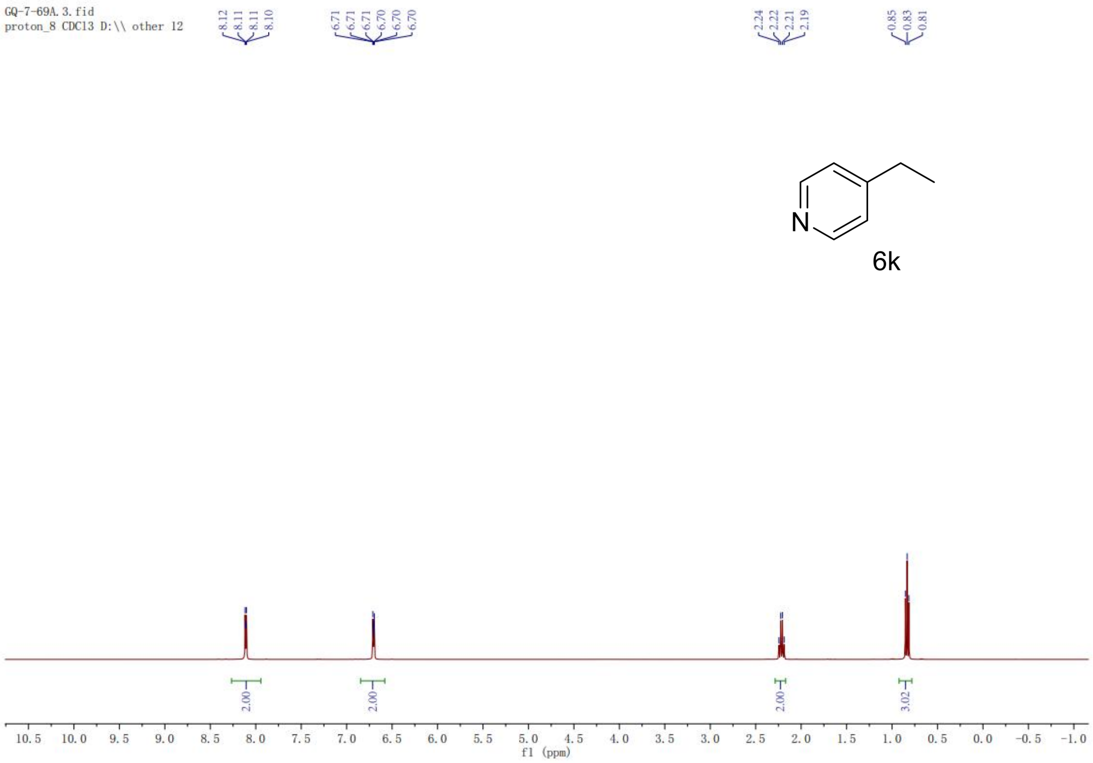
GQ-7-69A.3.fid
proton_8 CDCl3 D:\\ other 12
8.12
8.11
8.11
8.10
6.71
6.71
6.71
6.70
6.70
6.70
2.24
2.22
2.21
2.19
0.85
0.83
0.81
N
6k
2.00
2.00
2.00
3.02
f1 (ppm)

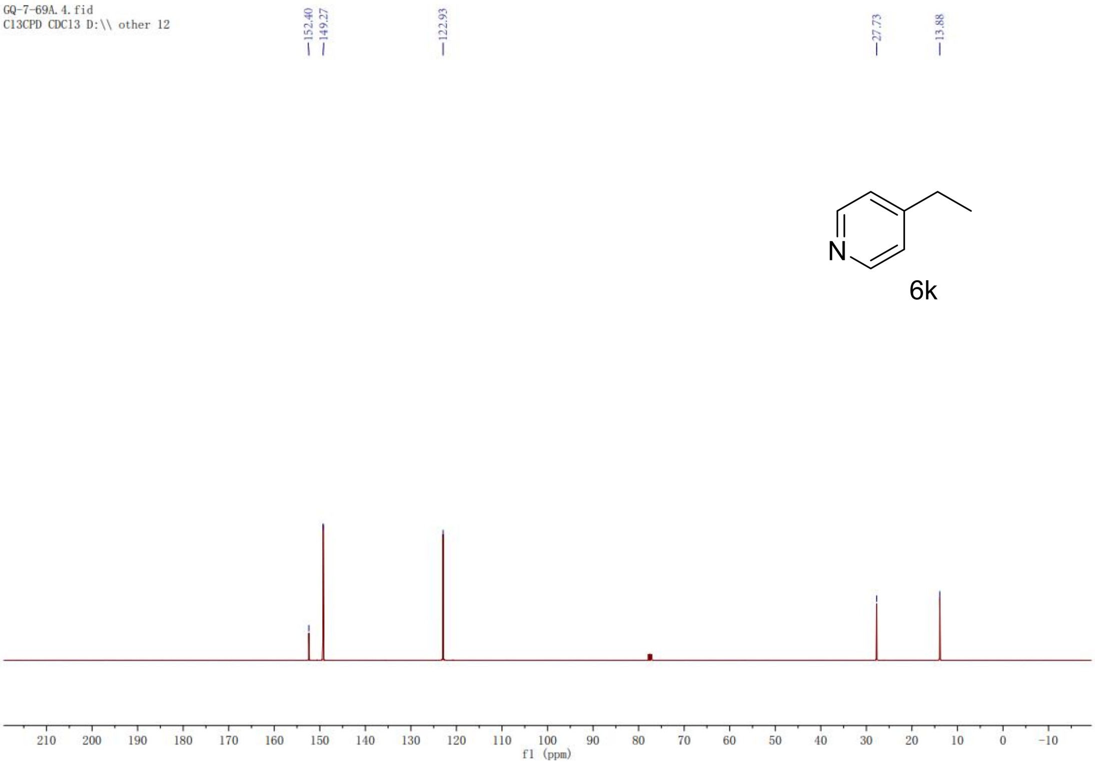
GQ-7-69A.4.fid
C13CPD CDCl3 D:\\ other 12
152.40
149.27
122.93
27.73
13.88
N
6k
f1 (ppm)

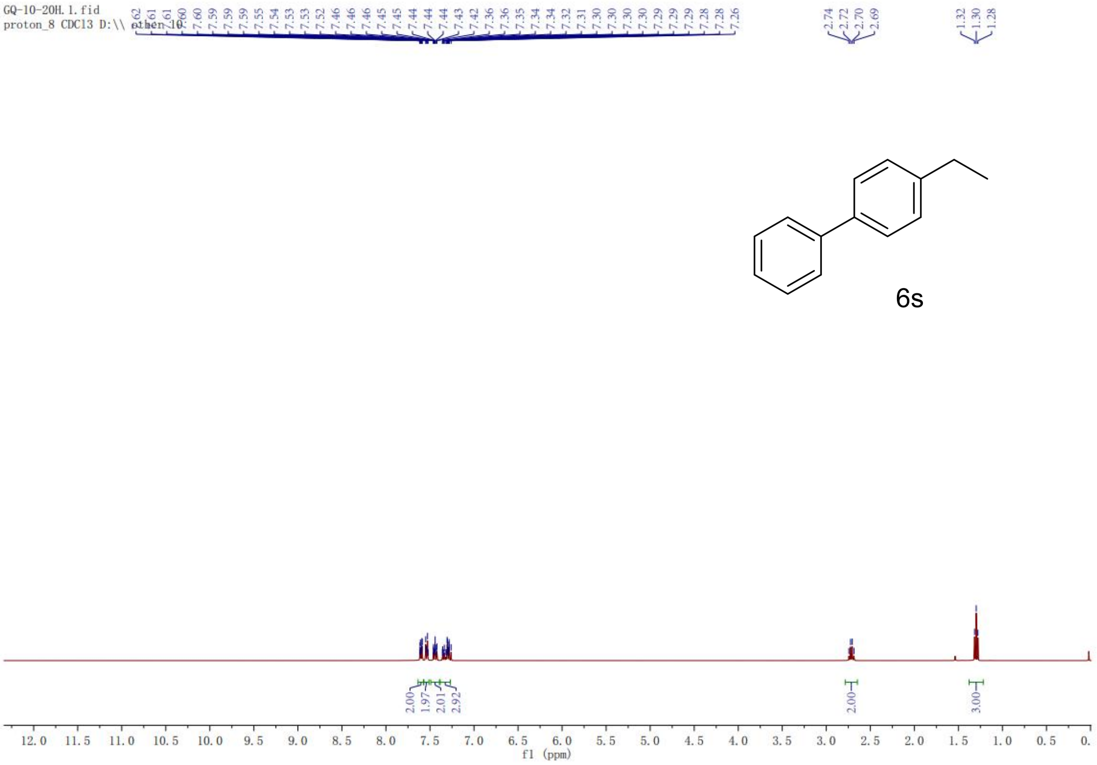
GQ-10-20H.1.fid
proton_8 CDCl3 D:\\ other 10
7.62
7.61
7.61
7.60
7.60
7.59
7.59
7.59
7.55
7.54
7.53
7.53
7.52
7.46
7.46
7.46
7.45
7.45
7.44
7.44
7.44
7.43
7.42
7.36
7.36
7.35
7.34
7.34
7.32
7.31
7.30
7.30
7.30
7.30
7.29
7.29
7.29
7.28
7.28
7.26
2.74
2.72
2.70
2.69
1.32
1.30
1.28
6s
2.00
1.97
2.01
2.92
2.00
3.00
12.0
11.5
11.0
10.5
10.0
9.5
9.0
8.5
8.0
7.5
7.0
6.5
6.0
5.5
5.0
4.5
4.0
3.5
3.0
2.5
2.0
1.5
1.0
0.5
0.
f1 (ppm)

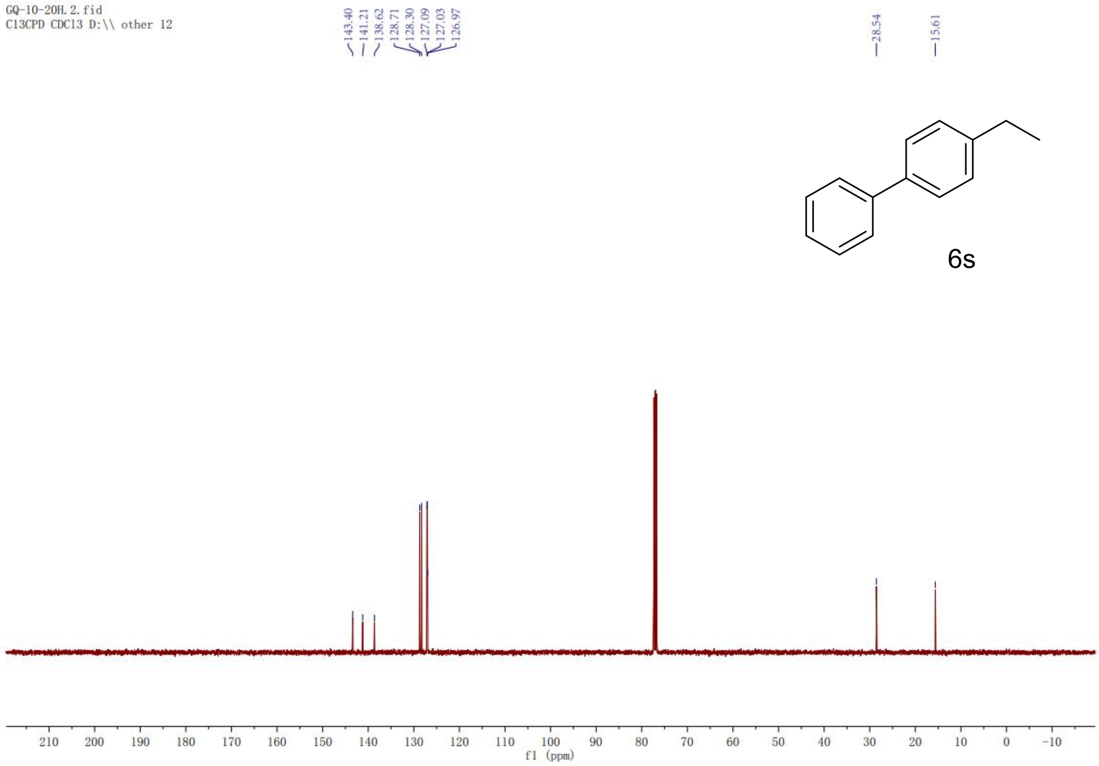
GQ-10-20H.2.fid
C13CPD CDCl3 D:\\ other 12
143.40
141.21
138.62
128.71
128.30
127.09
127.03
126.97
28.54
15.61
6s
210
200
190
180
170
160
150
140
130
120
110
100
90
80
70
60
50
40
30
20
10
0
-10
f1 (ppm)

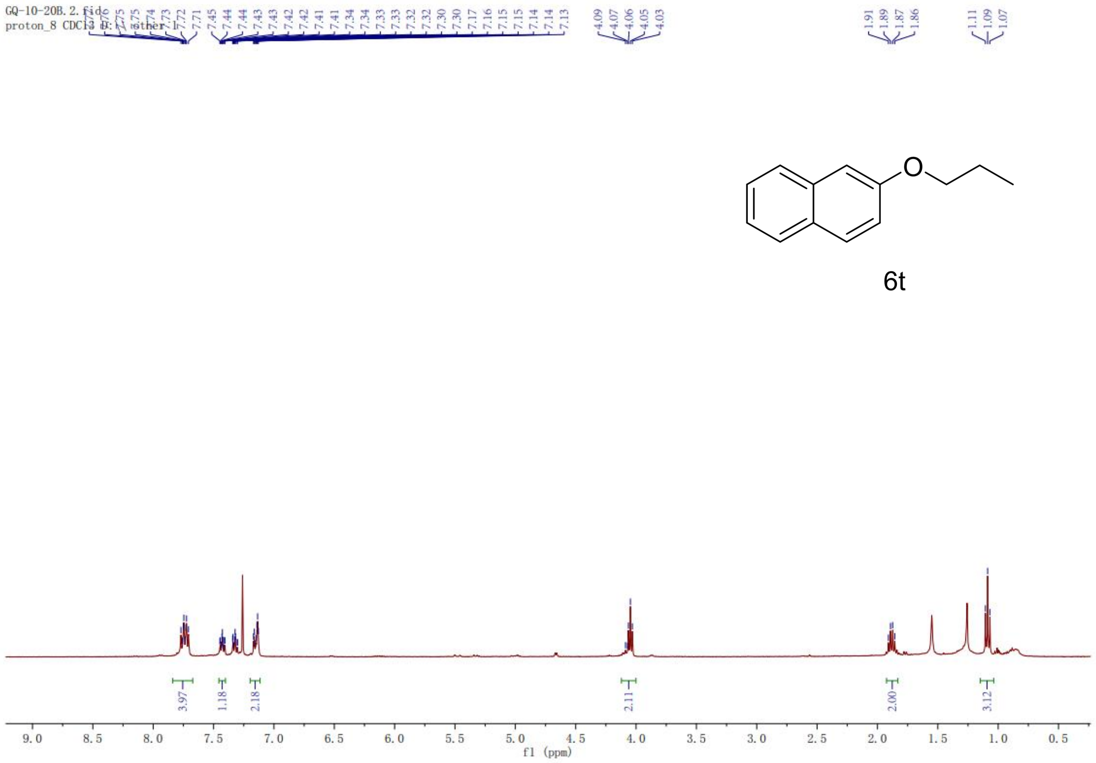
GQ-10-20B.2.fid
proton_8 CDCl3 D:\\ other 1
6t
9.0
8.5
8.0
7.5
7.0
6.5
6.0
5.5
5.0
4.5
4.0
3.5
3.0
2.5
2.0
1.5
1.0
0.5
f1 (ppm)

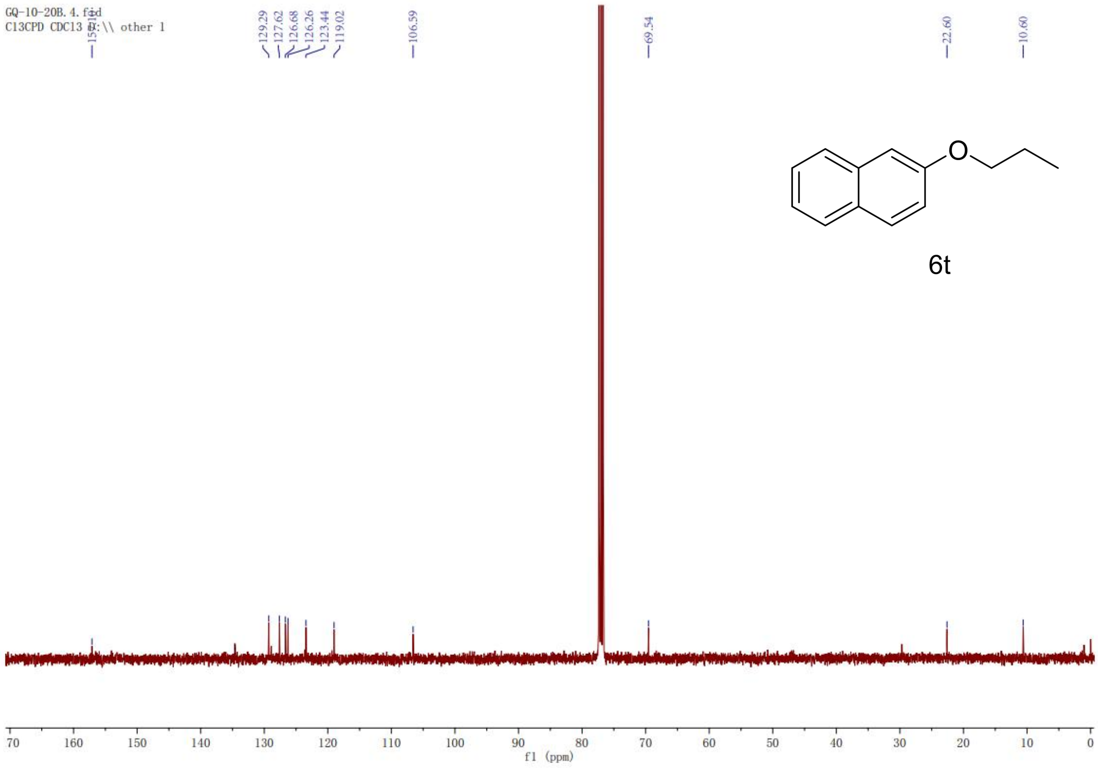
GQ-10-20B.4.fid
C13CPD CDCl3 D:\\ other 1
129.29
127.62
126.68
126.26
123.44
119.02
106.59
69.54
22.60
10.60
6t
f1 (ppm)

## 11. References


[1] Y. S. Wagh, N. Asao, *J. Org. Chem.* **2015**, *80*, 847-851.

[2] S. Liang, G. B. Hammond, B. Xu, *Chem. Commun.* **2016**, *52*, 6013-6016.

[3] K. Okuma, O. Sakai, K. Shioji, *B. Chem. Soc. Jpn*. **2003**, *76*, 1675-1676.

[4] G. A. Molander, A. R. Brown, *J. Org. Chem.* **2006**, *71*, 9681-9686.

[5] G. Shen, H. Liu, J. Chen, Z. He, Y. Zhou, L. Wang, L. Luo, Z. Su, B. Fan, *Org. Biomol. Chem*. **2021**, *19*, 3601-3610.

[6] Y. Yasu, T. Koike, M. Akita, *Org. Lett.* **2013**, *15*, 2136-2139.

[7] J. C. Roberts, J. A. Pincock, *J. Org. Chem.* **2006**, *71*, 1480-1492.

[8] S. E. Denmark, C. R. Butler, *J. Am. Chem. Soc.* **2008**, *130*, 3690-3704.

[9] F. Cong, Y. Wei, P. Tang, *Chem. Commun.* **2018**, *54*, 4473-4476.

[10] P. Ye, Y. Shao, X. Ye, F. Zhang, R. Li, J. Sun, B. Xu, J. Chen, *Org. Lett.* **2020**, *22*, 1306-1310.

[11] E. Arceo, J. A. Ellman, R. G. Bergman, *J. Am. Chem. Soc.* **2010**, *132*, 11408-11409.

[12] T. Mitsudome, T. Urayama, K. Yamazaki, Y. Maehara, J. Yamasaki, K. Gohara, Z. Maeno, T. Mizugaki, K. Jitsukawa, K. Kaneda, *ACS Catal.* **2016**, *6*, 666-670.

[13] X. Huo, M. Quan, G. Yang, X. Zhao, D. Liu, Y. Liu, W. Zhang, *Org. Lett.* **2014**, *16*, 1570-1573.

[14] X. Gu, J. Ying, R. S. Agnes, E. Navratilova, P. Davis, G. Stahl, F. Porreca, H. I. Yamamura, V. J. Hruby, *Org. Lett.* **2004**, *6*, 3285-3288.

[15] K. Li, R. Khan, X. Zhang, Y. Gao, Y. Zhou, H. Tan, J. Chen, B. Fan, *Chem. Commun.* **2019**, *55*, 5663-5666.

[16] L. Bettucci, C. Bianchini, C. Claver, E. J. Garcia Suarez, A. Ruiz, A. Melia, W. Oberhauser, *Dalton Trans*, **2007**, 5590-5602.

[17] D. van der Waals, A. Pettman, J. M. J. Williams, *RSC Adv*. **2014**, *4*, 51845-51849.

[18] J. Petrignet, A. Boudhar, G. Blond, J. Suffert, *Angew. Chem. Int. Ed.* **2011**, *50*, 3285-3289.

[19] B. M. Pierce, B. F. Simpson, K. H. Ferguson, R. E. Whittaker, *Org. Biomol. Chem*. **2018**, *16*, 6659-6662.

[20] K. M. Lambert, J. M. Bobbitt, S. A. Eldirany, L. E. Kissane, R. K. Sheridan, Z. D. Stempel, F. H. Sternberg, W. F. Bailey, *Chem. Eur. J.* **2016**, *22*, 5156-5159.

[21] T. Bosanac, C. S. Wilcox, *Org. Lett.* **2004**, *6*, 2321-2324.

[22] S. Natour S, R. Abu-Reziq, *RSC Adv*. **2014**, *4*, 48299-48309.

[23] V. Polshettiwar, M. N. Nadagouda, R. S. Varma, *Chem. Commun.* **2008**, 6318-6320.

[24] L. S. Bennie, C. J. Fraser, S. Irvine, W. J. Kerr, S. Andersson, G. N. Nilsson, *Chem. Commun.* **2011**, *47*, 11653-11655.

[25] D. Zhang, T. Iwai, M. Sawamura, *Org. Lett.* **2019**, *21*, 5867-5872.

[26] L. J. Goossen, K. Ghosh, *Chem. Commun.* **2002**, 836-837.

[27] T. Osako, K. Torii, A. Tazawa, Y. Uozumi, *RSC Adv.* **2015**, *5*, 45760-45766.

[28] W. Wang, A. Zheng, P. Zhao, C. Xia, F. Li, *ACS Catal.* **2014**, *4*, 321-327.

[29] A. Modvig, T. L. Andersen, R. H. Taaning, A. T. Lindhardt, T. Skrydstrupet, *J. Org. Chem.* **2014**, *79*, 5861-5868.

[30] B. Han, C. Ren, L. Wu, *Organometallics* **2023**, *42*, 1248-1253.
